# Exploring barriers and facilitators of implementing an at-home SARS-CoV-2 antigen self-testing intervention: The Rapid Acceleration of Diagnostics—Underserved Populations (RADx-UP) initiatives

**DOI:** 10.1371/journal.pone.0294458

**Published:** 2023-11-16

**Authors:** Lisa Maria Cross, Amelia DeFosset, Bola Yusuf, Donaldson Conserve, Rakiah Anderson, Christina Carilli, Warren Kibbe, Michael Cohen-Wolkowiez, Alan Richmond, Giselle Corbie, Gaurav Dave

**Affiliations:** 1 Department of Social Medicine, University of North Carolina at Chapel Hill, Chapel Hill, North Carolina, United States of America; 2 Department of Prevention and Community Health, Milken Institute School of Public Health, The George Washington University, Washington, D.C, United States of America; 3 Department of Maternal and Child Health, University of North Carolina at Chapel Hill, Chapel Hill, North Carolina, United States of America; 4 Duke Clinical Research Institute, Duke University, Durham, North Carolina, United States of America; 5 Community-Campus Partnerships for Health, Raleigh, North Carolina, United States of America; Independent Consultant, UNITED STATES

## Abstract

**Background:**

Evaluating community-based programs provides value to researchers, funding entities, and community stakeholders involved in program implementation, and can increase program impact and sustainability. To understand factors related to program implementation, we aimed to capture the perspective of community partners engaged in organizing and executing community-engaged programs to distribute COVID-19 at-home tests in underserved communities.

**Methods:**

We conducted semi-structured interviews and focus groups with community-based stakeholders informed by the Outcomes for Implementation Research framework.

**Results:**

Findings describe how community-engaged communication and dissemination strategies drove program adoption among grassroots stakeholders. Establishing and sustaining trusted relationships was vital to engaging partners with aligned values and capacity. Respondents characterized the programs as generally feasible and appropriate, and community partners felt capable of delivering the program successfully. However, they also described an increased burden on their workforce and desired more significant support. Respondents recognized the programs’ community engagement practices as a critical facilitator of acceptability and impact.

**Discussion:**

Implementation evaluation aims to inform current and future community outreach and engagement efforts with best practices. As we continue to inform and advance community-engaged disaster response practice, a parallel reimagining of public health funding mechanisms and timelines could provide a foundation for trust, collaboration, and community resiliency that endures beyond a given crisis.

## Introduction

Communities of color were disproportionately impacted by the COVID-19 pandemic, seeing higher morbidity and mortality from the virus [1, 2]. Several efforts have been launched at the federal, state, and local levels to address testing barriers specifically, including online screening for COVID symptoms and drive-through testing centers to increase testing accessibility [3]. Other efforts to decrease COVID-19-related health disparities in minority populations included culturally sensitive education programs [4, 5] and various programs distributing at-home test kits to underserved populations around the country [6, 7].

Mounting evidence supports that partnering with communities to execute public health programs is important for impact and sustainability [8–10]. Among historically underserved or marginalized communities, meaningful engagement in public health programs is especially critical [8]. Shared decision-making around program design, implementation, and research has resulted in more tailored, acceptable, and ultimately impactful interventions within communities that have been historically left out of, or actively harmed by, public health efforts [11]. Community-engaged interventions that center on unique community strengths and preferences are essential to reduce disparities in COVID-19 impacts. Looking beyond the present crisis, the risk of large-scale emergencies, including pandemics and natural disasters, is slated to rise over the coming decades, with impacts likely to accrue disproportionately among historically marginalized groups [12, 13]. Explicating both promising intervention models and the community engagement strategies that gave rise to them is a vital and timely need. Our study fills a gap in the literature by capturing perspectives of community partners engaged in organizing and executing community-engaged programs to distribute COVID-19 at-home tests in underserved communities, and reporting on lessons learned through partnering with communities to develop and evaluate two COVID-19 self-testing interventions. These qualitative findings provide crucial insight that researchers and practitioners can use to build community resilience in future public health emergencies [14].

### *Say Yes*! *COVID Test* and *You and Me COVID Free*

The *Say Yes COVID Test (SYCT)* and the related *You and Me COVID Free (YMCF)* initiatives were disseminated and implemented as part of the Rapid Acceleration of Diagnostics in Underserved Populations (RADx-UP) program [15]. Early in the course of the pandemic, the SYCT and YMCF initiatives provided underserved populations with at-home tests to reduce the spread of COVID-19 in high-risk communities and increase community members’ knowledge of the virus [16, 17]. Before at-home tests were widely available, the programs were implemented as a public-private partnership between state and local health departments, Amazon, and community partners in counties with high rates of community transmission and social vulnerability. For SYCT, 24,582 test kits (25 tests per kit = 614,550 tests) were distributed in Pitt County, North Carolina (NC), and 38,933 kits (25 tests per kit = 973,325 tests) in Hamilton County, Tennessee (TN), through the health department and in collaboration with community partners between April and June 2021. The impact of the intervention was then assessed using publicly available data including COVID-19 testing, hospitalization, vaccination, and mobility data as well as wastewater COVID-19 viral load. The details of the SYCT study design and protocol can be found in Ciccone et al. [16]. The YMCF program was piloted in Merced County, California (CA), and used a similar community-partnered implementation approach to SYCT, with the addition of social marketing principles to promote COVID-19 testing and vaccination. In November and December 2021, 200,000 test kits were distributed through the health department and other community partners (2 tests per kit = 400,000 tests). All communities have a high rate of underserved community members.

Both programs relied on community partnerships to create a meaningful program implementation [18]. They engaged community-based organizations, such as schools, churches, and community centers, to facilitate outreach and test distribution [19] to build trust [20] and increase program impact. The programs were grounded in community engagement principles [21], providing the guiding framework and core implementation strategy. Both programs leveraged established networks and partnerships to operationalize a community-specific implementation plan, harness community expertise and assets, and maximize the program’s acceptability, uptake, efficiency, and impact. In the two SYCT communities, the RADx-UP team used a community advisory board model, wherein Community-Campus Partnerships for Health (CCPH) built on existing contacts to identify community leaders (organizations and individuals) to participate on the board and then worked with them to engage others in their networks to join as board members or distribution partners. Advisory boards then defined a local implementation strategy to coordinate the specific distribution partners, sites, and methods for distributing kits. Distribution partners were community organizations involved in executing their local strategy, with guidance from the board. The RADx-UP team further guided these efforts by developing and implementing a promotional campaign, with the input of community members, that included social media, flyers, etc. [16]. Instead of an advisory board, the YMCF program partnered with one widely known and respected community organization, United Way, to coordinate the distribution of test kits by community distribution partners in the county. Otherwise, the programs were the same.

Our goal in this study was to understand the implementation of the SYCT and YMCF initiatives from the perspective of community partners engaged in organizing and executing the programs in their communities. We used a qualitative, descriptive approach, informed by implementation science frameworks, to examine community participants’ program experiences. Evaluating community-based programs benefits researchers, funding entities, and community stakeholders involved in the program implementation, and can increase the impact and sustainability of a program [22]. A secondary goal for this study was to use insights gained to generate hypothesis for further quantitative evaluation of our implementation outcomes [23].

This study was designed to generate actionable information about program strengths and weaknesses that could inform the development of large-scale, community-engaged efforts to respond to emerging public health crises, an as-yet underexamined area with urgent implications for both population health and health equity.

## Methods

### Background and setting

The core intervention components of SYCT and YMCF were the distribution of free rapid at-home testing kits to those disproportionately impacted by COVID-19; mainly communities with a larger African American and Hispanic population. To select intervention communities, the RADx- UP Coordination and Data Collection Center (CDCC) worked with Datarobot, Inc. to model disease transmission based on county-level data, including COVID-19 disease burden, population size, vaccination status/estimated immunity, and polymerase chain reaction testing rates [16]. They also considered data availability for modeling and other RADx-UP activities that would allow them to quickly tap into existing community relationships and networks [16], ultimately selecting counties where the program was likely to have the most impact and most favorable implementation context; meaning those communities which were most likely to succeed in the distribution of COVID-19 test kits to their community members. This study focuses specifically on three of these counties. This study was approved by the UNC Chapel Hill Institutional Review Board (protocol # 21–2872).

### Study team positionality

The core study team, responsible for the design, execution, and reporting, were authors LK, AD, DC, BY. All are credentialed public health researchers (PhD and Masters-level) with formal training and experience conducting qualitative research and evaluation, including community-engaged initiatives (4–10 years of experience). LK is a white woman, originally from Germany with a focus in exercise and sports science. AD is a white woman, originally from North Carolina with experience in chronic disease disparities in the rural south and in California. DC is a Black man originally from Haiti with a focus in dissemination of HIV programs, BY is a Black woman originally from Nigeria with a background in clinical medicine. LK, AD, BY are staff of the external firm, within UNC’s School of Medicine, who conducted the evaluation. DC is an Adjunct Assistant Professor with UNC’s School of Public Health, with primary appointment at George Washington University. All had no prior relationships with study participants.

### Evaluation guiding framework

This study sought to capture the perspectives of individuals engaged in organizing and executing SYCT and YMCF in their communities to generate practice-relevant information on the strengths and weaknesses of the community-engaged implementation strategy. The Outcomes for Implementation Research, a heuristic taxonomy developed through a rigorous review of implementation science literature [24], guided the development of data collection and analysis tools and processes. This taxonomy was selected because it draws on proven and foundational evaluation [e.g., Glosgow’s RE-AIM framework; 25] and implementation science models [e.g., Rogers’ Diffusion of Innovations theory; 26], grounded in an understanding of how components are being applied in practice. Critically though, it provides a distilled set of eight implementation outcomes that the evaluation team felt were both feasible and important to examine. Given the goal of harvesting practical lessons in a highly dynamic public health crisis, the eight implementation outcomes are: Acceptability, Adoption, Appropriateness, Feasibility, Fidelity, Implementation Cost, Penetration, and Sustainability [26]. See S1 Table for a detailed description of our analysis approach.

Several adaptations were made to the framework to further focus attention on dimensions and constructs: 1) that could be productively explored through qualitative inquiry, and 2) had the most immediate implications for practitioners and decision-makers seeking to replicate the program model, including factors that motivated community members to participate in distributing test kits (communication/engagement, adoption), their expectations and reflections on what was required for them to implement the program (appropriateness and feasibility), and what they felt worked well and what didn’t about the community-engaged program (acceptability, barriers/facilitators, recommendations). We did not measure fidelity, implementation cost, and penetration, as these constructs were more suited to quantitative measurement [24] and were measured through other parts of the study. We added questions/codes around communication to examine dynamics between community-based implementation teams and program planners, given the centrality of community engagement practices to the SYCT program [27]. We also combined appropriateness and feasibility in analysis as respondents tended to discuss these constructs in tandem or in a way that was difficult to parse while remaining ‘close’ to the data. We examined the construct of sustainability through two dimensions: barriers and facilitators and satisfaction/recommended adaptations. As the program was inherently term-limited, the team felt participant reflections around what helped or hindered program success during active implementation and what they would do differently in the future would provide helpful insights into the potential for program sustainability.

### Data collection

#### Sampling strategy

We used criterion-i sampling to recruit individuals with firsthand experience in implementing the programs as either advisory board members or community leaders developing their county’s implementation strategy or as distribution partners executing on-the-ground activities [28]. These roles were not mutually exclusive, although understanding differences in perspectives based on a participant’s role was of interest. All organizations participating in the SYCT or YMCF program were eligible for data collection. We identified specific representatives to invite within each organization by reviewing SYCT/YMCF community engagement plans and activities. Representatives were eligible to participate if they were at least 18 years old at the time of data collection and were comfortable completing an interview/focus group (FG) with project staff in English or Spanish.

Organizational representatives whose primary role was as distribution stakeholders were invited to participate in FGs, given the comparative strength of this approach in generating rich or layered qualitative data through group discussion [29–31]. Our expectation was that interactions between participants would elicit details and insights germane to our research goals, given the collaborative nature of the intervention. To allow for open discussion among this group regarding the whole program experience, including interaction with advisory boards or implementation leaders in their county, representatives from organizations that served in these roles were omitted from FGs. Instead, advisory board members were invited to participate in individual semi-structured interviews. FGs were organized by county to enable the exploration of county-specific contexts and experiences. We aimed for balance in our sample for both data collection methods, conducting equal numbers of interviews and FGs across the three counties. Given the limited number of advisory/leadership organizations, we aimed to interview all organizations that participated in this capacity (8 advisory board members). We aimed for two FGs for each of the three counties (8–12 participants each), in keeping with best practices [29], expectations around the level of data needed to reach thematic saturation [32], and the rapid qualitative analysis approach guiding the evaluation [33–39].

#### Recruitment approach

We designed our recruitment approach to be both community-partnered and community-centered. Recruitment for this evaluation study occurred in August 2022. We worked with partners in each community who had established the initial programmatic connections to invite organizations to participate in interviews/FGs. We asked partners to develop a contact list of potential organizations/representatives for each community. Partners contacted the identified organizations/representatives to introduce them to the study and announce researcher outreach. The research team had access to participant names and email addresses.

The research team then sent emails via REDCap that contained a link to complete a brief recruitment survey which screened for eligibility and collected written informed consent, describing the basic procedures, risks, and incentives associated with participating (a $100 gift card for interviews, $50 gift card for FGs). As needed to complete the recruitment process, individuals received up to three email reminders or two phone calls. Individuals who did not complete the survey after the maximum number of contact attempts, were screened as ineligible, or did not consent to participate, did not receive any further contact from the study team.

#### Data collection procedures

Participant demographics and information about their role within their community and in SYCT/YMCF were captured via a 25-item (advisory board members) or 6-item (distribution stakeholders) REDCap survey. Surveys were pilot tested in REDCap by the research team. All interviews and FGs were conducted between August and October 2022. All participants agreed to use a virtual conferencing platform that allowed for video recording, and all participants chose to participate in English. In confirmation emails, the research team encouraged participants to join from a private location free from distractions. We shared that research staff involved in data collection would participate from a private location and via a secure internet connection approved by UNC’s security and research oversight bodies. Two research team members were involved in each interview, one as a facilitator and one as a notetaker. The facilitators (AD, DC) had formal training and extensive prior experience in qualitative data collection. Notetakers received a one-hour training in general qualitative data collection methods and principles. They were also trained in project-specific methods during group working meetings led by the facilitators, which focused on practicing data collection tools and protocol before use. Interview and focus group guides were pilot tested by the research team during these sessions.

At the beginning of each data collection session, participants were asked if they consented to have the session recorded, and all agreed. Even so, notetakers attempted to capture the conversation as close to verbatim as possible. The facilitator remained on video for the session and encouraged participants to do the same, especially during the FGs, where facilitating participant interactions was a goal. As feasible during interviews or FGs, the facilitator made high-level notes regarding body language or other noteworthy aspects of the interview.

Individual interviews were scheduled for one hour. Before beginning, participants were informed that their responses would be kept confidential, data would be de-identified during transcription, there were no right or wrong answers, and that it was okay to skip any questions or end the interview at any time if they choose, per the informed consent they signed during recruitment. The facilitator then moved through the interview guide, reading questions and probes as written, and in order, to the extent possible. Real-time modifications were made to respond to the flow of the conversation (e.g., skip questions that were addressed already) or to provide clarity when prompted by respondents (e.g., requests to reword a question).

FGs were scheduled for 90 minutes. As with the interviews, participants were reminded of the contents of the informed consent, with the facilitator additionally encouraging the group members to keep the FG content confidential. To further ensure confidentiality within the group, we replaced the names that automatically appear on the screen when participants join the video conference with a randomly assigned number; research staff used these numbers to refer to participants throughout the evaluation. As with the interview process, the facilitator worked through the guide in order and as written while making real-time adjustments. Facilitators encouraged and responded dynamically to discussions and interactions between group members, adjusting the interview guide accordingly. For both the interviews and FGs, the guide included questions on (1) the counties’ response to COVID-19, (2) the participants’ knowledge and understanding of their county’s health needs, health priorities, available resources, and gaps in resources, and (3) the impact and success of SYCT/YMCF.

Following each data collection session, the facilitator and notetaker used a structured debrief process to refine understanding and capture preliminary impressions and insights. For instance, team members noted answers to questions they did not fully understand and sought to generate clarity or consensus through group discussion. Recorded interviews were transcribed verbatim, de-identified, and packaged with associated notes for analysis.

### Data analysis

#### Quantitative demographic information

We used basic descriptive statistics to describe the self-reported demographic characteristics of participants, as collected via brief recruitment surveys.

#### Qualitative interview and focus group data

We used a primarily deductive rapid content analysis approach modeled after Gale et al. [34] and informed by other practical applications of rapid qualitative inquiry [36, 39]. The method was designed to enable rapid generation and dissemination of qualitative insight to drive implementation or translation decisions, which was an explicit goal of the evaluation. Gale et al. [34] found results using this method to be consistent with those of a parallel comparative in-depth qualitative analysis and concluded this is an appropriate method for producing valid and actionable findings in a short timeframe. The analysis team consisted of three research team members: the two facilitators, and one notetaker who participated in data collection. One of the facilitators had also been involved in the design and planning of both the SYCT/YMCF program and evaluation.

Before data collection, the analysis team developed a set of structured analytic tools grounded in the study’s guiding framework (described above), including a codebook with apriori defined codes and sub-codes, a structured template for organizing data from each session, and a rapid analysis matrix (in Excel) for sorting and comparing data across all sessions. We made minor refinements to the tools during data collection based on observations and reflections generated during structured debriefs. When one interview and FG transcript became available, the team piloted the tools and process for coding each transcript in parallel and then met to discuss the results. Additional minor modifications were made based on the discussion, for instance, adding codes or sub-codes, refining definitions, or logging coding rules and norms. Because changes were minor, the coding of transcripts used in piloting was considered final and used in the second-level coding [40]. As preliminary reflections during data collection and pilot analysis did not highlight analysis-relevant differences between FG and interview data and given the potential for overlap between these two groups, the team decided to analyze these data together.

The team completed the remainder of the analysis between November and December 2022, concurrent with data collection. They met weekly to complete first-level coding; before each hour-long analysis meeting, one primary coder assigned codes and sub-codes by sorting content from transcripts into the analysis matrix. Primary coders copied portions of text from transcripts directly into the matrix to maintain the original wording and sentiment while generating a summary statement to facilitate the identification of patterns or themes in the second-level analysis [40]. The two other team members reviewed the transcript and associated notes, as needed, before the meeting. During each meeting, the primary coder walked through their coding decisions in the matrix and the summary statements they included. The group affirmed or adjusted coding based on discussion and consensus. The team allowed new codes to emerge from the data during this process, adding rows to the matrix and modifying the codebook and coding rules accordingly. The team explicitly reflected on the adequacy of codes and definitions as more data were added to the matrix, enabling comparison and identification of patterns across interview/FG discussions, and made modifications where needed. Likewise, as analysis was concurrent with data collection, as new codes, subcodes, and themes emerged, these were explored with probes in subsequent interviews and focus groups. When coding definitions and rules evolved, they were applied retrospectively, i.e., coding was changed where required. Thematic saturation was reached after six transcripts, after which time no new codes emerged.

Once the team completed first-level coding on all transcripts, one analysis team member (AD) conducted second-level analysis, identifying themes within each conceptual dimension by grouping codes and sub-codes and prioritizing insights or sentiments expressed across discussions. However, unique or outlier codes/sub-codes were also examined and retained in the results. The other two team members reviewed second-level analysis output, including descriptions of themes and component codes, selected illustrative quotes, densities, and narrative descriptions of the results. The group then met to review and discuss any suggested changes or areas needing clarification, after which the results were finalized.

## Results

### Participants

Five advisory board members (4 female and one male) participated in in-depth interviews; three did not respond to our outreach attempts. Fifteen distribution stakeholders (10 female, five male) participated in FGs, of 138 who were invited. Participants were between 40 and 70 years old and represented all three counties (NC 2, TN 2, CA 1). The majority of the interview participants were Black/African American. Focus group participants were between 25 and 70 years old and were White (7) or Black/African American (7); one participant identified as Hispanic/Latinx.

### Conceptualization of findings

A synthesis of our findings and representative quotes is shown in Table 1.

**Table 1:** Synthesis of content analysis findings on implementation outcomes.

Construct	Dimension and Themes (density)	Illustrative Quote
**Communication/engagement**- How respondents were approached or engaged around the project, experiences with communicating with the SYCT team.	*Initial contact*: through a known/trusted partner or network (7) through a community organization (1) *Level of needs or issues needing response from SYCT program team*: Few, if any (4) Minor needs/issues (3) Major needs/issues (1) *Impressions of communication in response to needs/issues*, *or in general*: Positive (7) Somewhat or significantly negative (2)	[Initial contact-through a known/trusted partners or network] *I’m real busy and sometimes you’ve got to pick and choose the things that you can do and not do*. *But like I said this was one*, *this project was one that was real[sic] important*. *So*, *yeah*, *I think by number one*, *I knew her anyway*. *We worked together on many things so*, *again*, *it’s all coming down to trust and relationship*. *So*, *I had that with her*, *so*, *when she called it was kind of hard to say no*. *And then when I saw what they were doing*, *I said yes*. *So*, *yeah*, *I think that was a good way to go*. *(Interview Participant (I)*.*3)*.
**Adoption**- why respondents chose to participate in the program. Individual and organizational motivation to engage initially.	Mission alignment (8) Potential for impact on pandemic (3) Community collaboration (2)	[Mission alignment] *Because we do everything*. *Our ministry is more of helping in the community*. *We help*. *We’re a community that helps*.*—We work with men and women who comes [sic] out from jail*. *We work with people who are homeless*. *We help pay people’s rents*, *light bills*. *We’ll community network*. *We have feeding programs*. *We just believe in helping*, *and we have disaster sites in different locations*. *We’re just a network of folks that come together and help in the community (I*.*6)*. [Potential for impact on pandemic]*—I do tend to work more with the African American community*. *So*, *again that made it easier because they did not have the information and information*, *good information that you can share*, *is really important*. *So*, *it was easy again for me as well people that were working with me to buy into this project*. *To want to do something because it benefited the community*. *It benefitted the African American community because they were really dying from Covid*, *so—and if they weren’t dying*, *they were hospitalized and they were in the hospital for long periods of time*. *So*, *anything that I and my group could do was really important*. *So*, *we did*. *We embraced it*. *(I*.*3)*
**Appropriateness and feasibility**- How closely matched the intervention was to the organization’s mission, normal scope and existing resources, how difficult it was to implement, and additional resources needed to do so.	*Organizational fit* Reach or location in priority communities (5) Perceive mission alignment (4) Access to needed infrastructure (1) *Extent to which program tasks aligned with normal work*. Highly aligned (5) Somewhat aligned (1) Departure- not aligned (2) *Level of difficulty*: Simple or easy- not difficult (5) Very difficult (1) *Resources or support needed*: Very minimal needs, if any (2) Additional person power (5) Capital investments (2) Marketing and promotional materials (1)	[Resources or support needed- additional person power] *I don’t know if we came ahead*, *honestly*, *financially*, *even with that stipend that we got*, *which again*, *for a small nonprofit is always a difficult thing*, *and I think*, *sometimes*, *government agencies don’t realize that*, *when they give us these jobs to do*, *that there is a ton of work and a ton of administrative stuff that needs to happen that either takes us away from what we’re already supposed to be doing or just becomes a financial burden for us*. *Right*? *So*, *and again*, *I would do it in a heartbeat again if need to be*.*—I think there needs to be more of an awareness about it*, *and I think it would’ve helped*, *even my staff*, *knowing that there was going to be a little bit of extra compensation or something would’ve made it much easier for them in times when their own families were at risk*. *Right*? *So*, *I think recognizing that our staff members are all single parents*, *and so*, *they had to make a decision- do we serve the community*, *or do we protect our children*? *(FG CA 9*.*29)*.
**Acceptability-** Extent to which respondents felt the intervention met the needs of their community, was satisfactory, or important.	*Extent to which program met community’s COVID 19 needs*: Succeeded in meeting an important need (8) Did not succeed in meeting a need (1) *Extent to which program addressed barriers to testing*: Addressed major testing barriers (8) Did not (1)	[Extent to which program addressed barriers to testing- addressed major testing barrier] *I think so*, *because some of the places when they had events—now*, *like I said*, *I did a lot of distributing tests to people one-on-one or whatever*, *but many times they were set up sites and many times they would be at the grocery store and places where people were constantly frequenting [sic]*. *So*, *therefore they were able to come get kits and do what they needed to do*, *and I thought that was good*. *I thought that was real strategic [sic]*, *that where people were hanging out*, *that’s where you would find folks distributing those kits*. *Those were kind of what I would call the events and then others were like on a more personal level passing them out to people when they call needing the kits (I*.*3)*
**Recommended adaptations-** How satisfied respondents were with the program and recommendations for changes or adaptations to improve the program.	Extend the duration of the program (5) Make kits available earlier, when need first arose (1) Ensuring program remain targeted on those most vulnerable to poor COVID 19 outcomes (1) More streamlined logistics (1) Including community voices in all areas of program design, including evaluation and research (1) Processes for communicating/coordinating across on-the-ground groups defined before program launch (1)	[Extend the duration of the program] *About three weeks after we finished the project here in [county] we had a significant spike in Covid cases and everybody was looking for test kits and there were none available*, *so I mean*, *I wish we could have had run the test a little longer here and had more kits but that just wasn’t feasible*.*” (I*.*7)*

#### Communication

Most evaluation participants (7) described being contacted initially through a known or trusted colleague or community partner/network, with many reflecting on personal outreach as key to them joining the effort.

Respondents were also asked about the questions or needs requiring communication across program teams (e.g., academic funders and community leaders). Respondents generally reported having almost no questions (4) or only minor needs requiring input (3); they felt the program was straightforward or simple to understand/execute. Participants reflected on significant issues in one FG, describing communication breakdowns that caused ‘stress’ and ‘conflict’ among those on the ground. Overall, however, most respondents (7) described communication during the program as positive, expressing appreciation for the SYCT program teams’ willingness to communicate through their preferred method or channel (e.g., phone vs. email, one-on-one discussion in addition to group meeting), and be proactive and responsive in providing needed information. In two instances, one FG and one interview from the same community, communication was described as somewhat or majorly flawed. These participants commented explicitly on communication with the academic lead organizations as challenging, convoluted, and even condescending, saying ‘[*we] felt like the stepchild*, *[and] almost walked out’ (I*.*8)*.

#### Adoption

All respondents that outlined their reasons for participating (8) described a sense of ‘shared mission’ or ‘mission alignment’ with the SYCT program. Respondents talked about participating in the program as a ‘no brainer’ because it would help their community, and helping their community was at the heart of their work.

Three respondents also discussed the potential impact of the program’s strategy (increasing testing accessibility) on community transmission as a prime motivator. FG participants were explicitly asked how the idea of community collaboration, i.e., working on the project with a coalition of local organizations/stakeholders, impacted their decision to participate. In two discussions, FG participants reported the program’s collaborative nature had been their deciding factor. Although participants were split in one of these FGs, some said their participation was more influenced by their trust in the lead organization than the idea of working with a coalition.

#### Appropriateness and feasibility

When asked why they were a good fit for the program, respondents described their organization’s reach or location in communities prioritized for test kit distribution (3), the perceived alignment of their organization’s mission or goals with that of the program (4), and their access to needed infrastructure, specifically warehouse space (1). Five respondents described SYCT tasks as strongly aligned with their organizations’ normal scope or capacities, saying ‘—*Just being involved in the community already providing information and things like that*. *It fit in perfect with the*, *with the Covid—it was a natural fit to us’ (NC FG 8*.*25)*. Although some noted initial, but temporary, challenges as they defined and implemented new or modified processes. Five respondents described SYCT program tasks as easy for their organization to execute and integrate with their normal work. Surprisingly, one respondent for whom tasks were highly aligned, felt they were nonetheless very difficult to accomplish, citing the intense fear around COVID-19 mitigation measures. Respondents were asked what additional resources their organization required to execute SYCT program tasks. Most reported needed very minimal support, if any. The most common need was additional person power (5); organizations marshaled additional staff time, volunteers, or new partnerships to meet program requirements and made investments to train this new workforce. Being able to leverage their existing networks and communication channels was discussed as key. A few of this group expressed the need for support from SYCT planners to compensate staff/partners for their efforts. Other support needs included capital investments to develop needed infrastructure (2) (e.g., storage or equipment, insurance, new IT tools), or support developing and distributing marketing and promotional materials.

#### Acceptability

Respondents were asked to describe their county’s needs around COVID-19 broadly. Seven respondents indeed focused on the need for more, and more convenient/accessible, tests, citing major barriers to accessing in-person testing which were perceived to be heightened in their communities (e.g., limited public transportation, few open testing centers, more workers without paid time off). Other needs mentioned included education around testing to help overcome misconceptions and hesitation (3), linguistically inclusive services and supports (2), and broader issues that predated, but intersected with, the COVID-19 pandemic such as homelessness, food insecurity, and limited internet access (1). Reflecting on the extent to which the program addressed these needs, most (7) agreed that it at least partially had, with one noting the need to provide education/marketing to help community members accept the intervention, once available. They explicitly attributed this success to the community-engaged dissemination approach. The program being planned and implemented by community stakeholders enabled them to harvest local wisdom to tailor intervention elements AND engendered trust among resident, One FG participant said the program did not meet the needs of their community, citing the confusion around online data collection activities and expressing frustration at the assumption that everyone in their community would have a smartphone or internet access. They also felt that the program had failed to fully consider and accommodate the needs of specific vulnerable groups including non-English speakers and the visually impaired. Focusing just on testing barriers, seven respondents said the program had effectively addressed their county’s barriers to testing.

#### Recommendations

Seven discussions included explicit recommendations. The most common recommendation for program planners, mentioned five times, was to extend the program. From the respondents’ perspective, the program ended before the need for test kits ended, with some even noting an increase in need just as the kits became unavailable. One respondent also commented that they would have preferred to have kits earlier. Like with the program ending, the beginning of kit availability did not correspond with the beginning of the need for kits, and by the time they were available, the perception was that COVID-19 had “already gotten out of control”. Other recommendations for improvement included keeping future program efforts targeted to those most vulnerable to testing barriers (1), more streamlined or professional logistics (i.e., having dedicated warehouse space) (1), emphasizing or including community voices in all areas of program design (1), and clearly defined processes for coordinating/communicating across on-the-ground community groups from the outset (1).

## Discussion

Our findings support and provide insight into how community-engaged program dissemination strategies can catalyze grassroots mobilization in public health disasters. To facilitate program adoption, SYCT/YMCF used communication and engagement strategies that centered and leveraged existing community relationships [41, 42]. When respondents reflected on why they joined the SYCT/YMCF effort, being approached by a known or trusted community leader as opposed to a governmental organization was the most commonly cited reason. Lack of trust in governmental organizations is a documented barrier to community engagement in health programs and research. Relatedly, respondents discussed participating as a ‘no brainer’, because they felt the effort was philosophically aligned with their values, even when tasks were unfamiliar or challenging [43]. This finding speaks to two related program strengths. First, it engaged local wisdom to identify and facilitate targeted partnerships. Second, it tapped into the community’s intrinsic values and motivations to drive action, lending credence to a core tenant of asset-based models being explored in public health [44].

Generally, findings support the feasibility and appropriateness of SYCT/YMCF implementation strategies, both which are established drivers of success in implementation science studies [8]. Respondents felt they were well situated to reach intended audiences and most described program tasks as closely aligned with their existing capacity and competencies-, as expected, -having the right partners at the table led to efficiencies and impact [8–10, 45]. However, respondents described needing extra investments to execute program tasks, even when aligned with their existing scope, especially in person power [46]. While tapping into existing partner- or volunteer networks helped them quickly respond to this challenge, at least one respondent described feeling that this added burden was under-addressed by academic program leaders. More proactive strategies for building human resource capacity among partner organizations could have mitigated this challenge, brought the program further in-line with community engagement best practices and promoted long-term sustainability [47].

Overall, respondents felt like they were able to implement the program in a way that overcame barriers to testing in their communities, while noting opportunities to strengthen future efforts. The strongest recommendation for improvement was the need to better align implementation timelines to community need, both beginning efforts earlier, when the need first arose, and extending efforts until community members felt the need was resolved. Respondents described facing complex external barriers, including widespread misinformation and fear of vaccines and testing. Overcoming this barrier required building trust over time and having tests available and accessible when community members were ready to accept them [48]. Many respondents then expressed regret that the program ended on a seemingly arbitrary timeline. This finding speaks to a major operational challenge for public health–the field is often trying to ‘hit a moving target’ in resource landscapes that are neither proactive nor agile, and where sustainability is often not sufficiently addressed [18, 49]. A substantial body of literature supports the importance of considering sustainability in particular when implementing community projects [48]. Our finding adds to this literature by highlighting how misaligned timelines can contribute to mistrust, especially in historically marginalized communities where trust is already justifiably low. To optimize impact, we must reimagine public health disaster response and funding models to center and evaluate community trust as a key outcome. Optimizing community-engaged disaster response practice will require a broader reimagining of public health systems, especially in how we fund our work. Although daunting, this effort would provide a critical foundation for trust, collaboration, and community resiliency that endures beyond a given health threat or response. Rohlmann et al. [50].

### Limitations

Despite its strengths, our study has a number of limitations. Due to the time lapse from the implementation of the study to its evaluation, we recognize the likelihood of participants’ recall bias that might impact the plausibility of the evaluation results. The number of participants recruited for this study was smaller than the initial recruitment goal due to changes in stakeholders’ affiliation with program organizations. It was not feasible to pilot the instruments and data collection processes prior to their implementation. In analysis, we ultimately combined acceptability and feasibility because, although conceptually distinct, the questions and probes used did not elicit data that enable us to explore them separately, as intended. Piloting the instrument could have prevented this limitation. Although our sampling approach engaged key players in the program implementation, findings represent the specific perspective of those who chose to partner on the project and were perhaps more favorably inclined. Other voices could have yielded different results. Even though the evaluation was conducted by an external group, and steps were taken to minimize any power differentials between participants and data collectors and protect privacy so respondents felt comfortable being candid, it is possible participants perceived the UNC evaluation team to be the same as the UNC program implementation team, opening the possibility of social desirability bias [51]. Finally, our rapid, deductive approach may not have allowed for the full expression or exploration of insights or themes related to participant experiences, although it enabled useful findings to emerge sooner.

## Conclusion

Our findings provide insight into how community-engaged communication and dissemination strategies drive program implementation among grassroots stakeholders. Leveraging trusted relationships is key to engaging partners with aligned values and capacity. The purpose of an implementation evaluation is to inform current and future community outreach and engagement efforts with best practices. A parallel reimagining of public health funding mechanisms and timelines could support the advancement and optimization of community-engaged disaster response practice, which could provide a foundation for trust, collaboration, and community resiliency that endures beyond a given crisis.

## Supporting information

S1 Table Data analysis methodology. (DOCX)

S1 Dataset (ZIP)

## References

[1] Doherty IA, Pilkington W, Brown L, Billings V, Hoffler U, Paulin L, et al. COVID-19 Vaccine Hesitancy in Underserved Communities of North Carolina. medRxiv. 2021. 10.1371/journal.pone.0248542 PMC8559933 34723973

[2] Miller S, Wherry LR, Mazumder B. Estimated Mortality Increases During The COVID-19 Pandemic By Socioeconomic Status, Race, And Ethnicity. Health Affairs. 2021;40(8):1252–60. doi: 10.1377/hlthaff.2021.00414 34288698

[3] Priem JS, Krinner LM, Constantine ST, McCurdy L. Diversification of COVID-19 Testing Resources to Decrease Racial/Ethnic Disparities: Comparative Use of Adaptive Approaches to Community Testing Across an Integrated Healthcare System. Dialogues in Health. 2022;1:100017. Epub 2022 May 27. doi: 10.1016/j.dialog.2022.100017 36942315 PMC9135493

[4] Anda SD, Budd EL, Halvorson S, Mauricio AM, McWhirter EH, Cioffi CC, et al. Effects of a Health Education Intervention for COVID-19 Prevention in Latinx Communities: A Cluster-Randomized Controlled Trial. American Journal of Public Health. 2022;112(S9):S923–S7. doi: 10.2105/AJPH.2022.307129 36446063 PMC9707712

[5] Gillard CJ, Al-Dahir S, Earls M, Singleton B. A Culturally Competent Vaccine Hesitancy Educational Model for Community Pharmacists to Increase Vaccine Uptake, Louisiana, 2021–2022. American Journal of Public Health. 2022;112(S9):S900–S3. doi: 10.2105/AJPH.2022.307070 36446056 PMC9707709

[6] Juarez R, Phankitnirundorn K, Ramirez A, Peres R, Maunakea AK, Okihiro M. Vaccine-Associated Shifts in SARS-CoV-2 Infectivity Among the Native Hawaiian and Other Pacific Islander Population in Hawaii. American Journal of Public Health. 2022;112(S9):S896–S9. doi: 10.2105/AJPH.2022.306973 36108254 PMC9707710

[7] Whanger S, Davis SK, Kemper E, Heath-Granger J, Hodder SL. Novel Strategies to Increase COVID-19 Testing Among Underserved and Vulnerable Populations in West Virginia. American Journal of Public Health. 2022;112(S9):S892–S5. doi: 10.2105/AJPH.2022.307004 36265093 PMC9707708

[8] Cyril S, Smith BJ, Possamai-Inesedy A, Renzaho AM. Exploring the role of community engagement in improving the health of disadvantaged populations: a systematic review. Global health action. 2015;8(1):29842. doi: 10.3402/gha.v8.29842 26689460 PMC4685976

[9] D’Agostino EM, Corbie G, Kibbe WA, Hornik CP, Richmond A, Dunston A, et al. Increasing access and uptake of SARS-CoV-2 at-home tests using a community-engaged approach. Prev Med Rep. 2022;29:101967. doi: 10.1016/j.pmedr.2022.101967 36061814 PMC9424120

[10] Williams NJ, Gill E, Punter MA, Reiss J, Goodman M, Shelley D, et al. Rapid Community Engagement in Response to SARS-CoV-2 Funding Opportunities: New York City, 2020‒2021. American Journal of Public Health. 2022;112(S9):S904–S8. 36446061 10.2105/AJPH.2022.307072 PMC9707719

[11] Drahota A, Meza RD, Brikho B, Naaf M, Estabillo JA, Gomez ED, et al. Community-Academic Partnerships: A Systematic Review of the State of the Literature and Recommendations for Future Research. Milbank Q. 2016;94(1):163–214. doi: 10.1111/1468-0009.12184 26994713 PMC4941973

[12] Marani M, Katul GG, Pan WK, Parolari AJ. Intensity and frequency of extreme novel epidemics. Proceedings of the National Academy of Sciences. 2021;118(35):e2105482118. doi: 10.1073/pnas.2105482118 34426498 PMC8536331

[13] Davidson TM, Price M, McCauley JL, Ruggiero KJ. Disaster impact across cultural groups: comparison of Whites, African Americans, and Latinos. Am J Community Psychol. 2013;52(1–2):97–105. doi: 10.1007/s10464-013-9579-1 23709270 PMC4386718

[14] Lane-Barlow C, Thomas I, Horter L, Fleurence R, Green J, Juluru K, et al. Experiences of Health Departments on Community Engagement and Implementation of a COVID-19 Self-testing Program. Journal of Public Health Management and Practice. 9900. doi: 10.1097/PHH.0000000000001688 36729971 PMC10198798

[15] Webb Hooper M, Compton WM, Walsh ER, Hodes RJ, Pérez-Stable EJ. Harnessing the Power of Community-Engaged Science to Facilitate Access and Uptake of COVID-19 Testing: RADx-UP. American Journal of Public Health. 2022;112(S9):S854–S7. doi: 10.2105/AJPH.2022.307105 36446064 PMC9707707

[16] Ciccone EJ, Conserve DF, Dave G, Hornik CP, Kuhn ML, Herling JL, et al. At-home testing to mitigate community transmission of SARS-CoV-2: protocol for a public health intervention with a nested prospective cohort study. BMC Public Health. 2021;21(1):2209. doi: 10.1186/s12889-021-12007-w 34863144 PMC8642753

[17] Singler L, Uhlenbrauck G, Corbie-Smith G, Richmond A, Hattem A, Linney K, et al. Say Yes! COVID Test: A Health Communication Campaign to Encourage Use of Rapid, At-Home Antigen Testing in Underserved and Historically Marginalized Communities. INQUIRY: The Journal of Health Care Organization, Provision, and Financing. 2023;60:00469580221146046. doi: 10.1177/00469580221146046 36704996 PMC9903010

[18] D’Agostino EM, Dave G, Dyer C, Hill A, McCarty D, Melvin S, et al. Listening to Community Partners: Successes and Challenges in Fostering Authentic, Effective, and Trusting Partnerships in the RADx-UP Program. American Journal of Public Health. 2022;112(S9):S846–S9. doi: 10.2105/AJPH.2022.307104 36446065 PMC9707723

[19] Barrett ES, Andrews TR, Roy J, Greenberg P, Ferrante JM, Horton DB, et al. Community- Versus Health Care Organization–Based Approaches to Expanding At-Home COVID-19 Testing in Black and Latino Communities, New Jersey, 2021. American Journal of Public Health. 2022;112(S9):S918–S22. doi: 10.2105/AJPH.2022.306989 36265092 PMC9707722

[20] Cheng J, Tsoh JY, Guan A, Luu M, Nguyen IV, Tan R, et al. Engaging Asian American Communities During the COVID-19 Era Tainted With Anti-Asian Hate and Distrust. American Journal of Public Health. 2022;112(S9):S864–S8. doi: 10.2105/AJPH.2022.306952 36108257 PMC9707711

[21] Pérez-Stable EJ, Hodes RJ, Schwetz TA. An NIH Response to COVID-19 That Engages Communities and Scientists. American Journal of Public Health. 2022;112(S9):S844–S. doi: 10.2105/AJPH.2022.307118 36446062 PMC9707713

[22] Judd J, Frankish CJ, Moulton G. Setting standards in the evaluation of community-based health promotion programmes—a unifying approach. Health promotion international. 2001;16(4):367–80. doi: 10.1093/heapro/16.4.367 11733455

[23] Barroga E, Matanguihan GJ. A Practical Guide to Writing Quantitative and Qualitative Research Questions and Hypotheses in Scholarly Articles. J Korean Med Sci. 2022;37(16):e121. doi: 10.3346/jkms.2022.37.e121 35470596 PMC9039193

[24] Proctor E, Silmere H, Raghavan R, Hovmand P, Aarons G, Bunger A, et al. Outcomes for implementation research: conceptual distinctions, measurement challenges, and research agenda. Adm Policy Ment Health. 2011;38(2):65–76. doi: 10.1007/s10488-010-0319-7 20957426 PMC3068522

[25] Glasgow RE, Vogt TM, Boles SM. Evaluating the public health impact of health promotion interventions: the RE-AIM framework. American Journal of Public Health. 1999;89(9):1322–7. doi: 10.2105/ajph.89.9.1322 10474547 PMC1508772

[26] Rogers EM, Singhal A, Quinlan MM. Diffusion of innovations. An integrated approach to communication theory and research: Routledge; 2014. p. 432–48.

[27] Taylor M, Dunston A, Richmond A, Conserve D, Corbie G, Dave G, et al. Using Community Organizing Practices to Advance Community Engagement in the Rapid Distribution of COVID-19 Test kits. PloS One. submitted.

[28] Palinkas LA, Horwitz SM, Green CA, Wisdom JP, Duan N, Hoagwood K. Purposeful Sampling for Qualitative Data Collection and Analysis in Mixed Method Implementation Research. Administration and Policy in Mental Health and Mental Health Services Research. 2015;42(5):533–44. doi: 10.1007/s10488-013-0528-y 24193818 PMC4012002

[29] Bender DE, Ewbank D. The focus group as a tool for health research: issues in design and analysis. Health Transit Rev. 1994;4(1):63–80. 10147164

[30] Lambert SD, Loiselle CG. Combining individual interviews and focus groups to enhance data richness. Journal of Advanced Nursing. 2008;62(2):228–37. doi: 10.1111/j.1365-2648.2007.04559.x 18394035

[31] Roller M, Lavarkas P. Strenghts of the Focus Group Method: An Overview. Applied Qualitative Research Design: A Total Quality Framework Approach 2015. p. 111–2.

[32] Guest G, Namey E, Chen M. A simple method to assess and report thematic saturation in qualitative research. PLoS One. 2020;15(5):e0232076. doi: 10.1371/journal.pone.0232076 32369511 PMC7200005

[33] Damschroder LJ, Lowery JC. Evaluation of a large-scale weight management program using the consolidated framework for implementation research (CFIR). Implementation Science. 2013;8(1):51. doi: 10.1186/1748-5908-8-51 23663819 PMC3656778

[34] Gale RC, Wu J, Erhardt T, Bounthavong M, Reardon CM, Damschroder LJ, et al. Comparison of rapid vs in-depth qualitative analytic methods from a process evaluation of academic detailing in the Veterans Health Administration. Implementation Science. 2019;14(1):11. doi: 10.1186/s13012-019-0853-y 30709368 PMC6359833

[35] Keith RE, Crosson JC, O’Malley AS, Cromp D, Taylor EF. Using the Consolidated Framework for Implementation Research (CFIR) to produce actionable findings: a rapid-cycle evaluation approach to improving implementation. Implementation Science. 2017;12(1):15. doi: 10.1186/s13012-017-0550-7 28187747 PMC5303301

[36] McMullen CK, Ash JS, Sittig DF, Bunce A, Guappone K, Dykstra R, et al. Rapid assessment of clinical information systems in the healthcare setting: an efficient method for time-pressed evaluation. Methods Inf Med. 2011;50(4):299–307. doi: 10.3414/ME10-01-0042 21170469 PMC3746487

[37] Ash JS, Sittig DF, McMullen CK, Guappone K, Dykstra R, Carpenter J. A rapid assessment process for clinical informatics interventions. AMIA Annu Symp Proc. 2008;2008:26–30. 18999075 PMC2656056

[38] Averill JB. Matrix analysis as a complementary analytic strategy in qualitative inquiry. Qual Health Res. 2002;12(6):855–66. doi: 10.1177/104973230201200611 12109729

[39] McNall M, Foster-Fishman PG. Methods of Rapid Evaluation, Assessment, and Appraisal. American Journal of Evaluation. 2007;28(2):151–68.

[40] Elliott V. Thinking about the Coding Process in Qualitative Data Analysis. The Qualitative Report. 2018;23(11):2850–61.

[41] Zuh C. Community Engagement: A summary of theoretical concepts. 2011.

[42] Griffith DM, Allen JO, DeLoney EH, Robinson K, Lewis EY, Campbell B, et al. Community-based organizational capacity building as a strategy to reduce racial health disparities. J Prim Prev. 2010;31(1–2):31–9. doi: 10.1007/s10935-010-0202-z 20127281

[43] Harrison R, Blickem C, Lamb J, Kirk S, Vassilev I. Asset-Based Community Development: Narratives, Practice, and Conditions of Possibility—A Qualitative Study With Community Practitioners. SAGE Open. 2019;9(1):2158244018823081.

[44] von Hippel C. A Next Generation Assets-Based Public Health Intervention Development Model: The Public as Innovators. Front Public Health. 2018;6:248. doi: 10.3389/fpubh.2018.00248 30234092 PMC6131659

[45] Bamberger M. The importance of community participation. Public Administration and Development. 1991;11(3):281–4.

[46] Andrulis DP, Siddiqui NJ, Purtle JP. Integrating racially and ethnically diverse communities into planning for disasters: the California experience. Disaster Med Public Health Prep. 2011;5(3):227–34. doi: 10.1001/dmp.2011.72 22003140

[47] Ramsbottom A, O’Brien E, Ciotti L, Takacs J. Enablers and Barriers to Community Engagement in Public Health Emergency Preparedness: A Literature Review. J Community Health. 2018;43(2):412–20. doi: 10.1007/s10900-017-0415-7 28840421 PMC5830497

[48] Gamboa-Maldonado T, Marshak HH, Sinclair R, Montgomery S, Dyjack DT. Building capacity for community disaster preparedness: a call for collaboration between public environmental health and emergency preparedness and response programs. J Environ Health. 2012;75(2):24–9. 22984732 PMC4651206

[49] Walugembe DR, Sibbald S, Le Ber MJ, Kothari A. Sustainability of public health interventions: where are the gaps? Health Research Policy and Systems. 2019;17(1):8. doi: 10.1186/s12961-018-0405-y 30646911 PMC6334403

[50] Rohlman D, Samon S, Allan S, Barton M, Dixon H, Ghetu C, et al. Designing Equitable, Transparent, Community-engaged Disaster Research. Citizen Science: Theory and Practice. 2022;7(1)(22). doi: 10.5334/cstp.443 36909292 PMC9997484

[51] Althubaiti A. Information bias in health research: definition, pitfalls, and adjustment methods. Journal of multidisciplinary healthcare. 2016:211–7. doi: 10.2147/JMDH.S104807 27217764 PMC4862344

