# Supplementary material for: Exploring barriers and facilitators of implementing an at-home SARS-CoV-2 antigen self-testing intervention: The Rapid Acceleration of Diagnostics—Underserved Populations (RADx-UP) initiatives
Source: PLoS One. 2023 Nov 16;18(11):e0294458. doi: 10.1371/journal.pone.0294458 (PMC10653400; doi:10.1371/journal.pone.0294458)
Supplement: S1 Table — (DOCX) [file pone.0294458.s001.docx]

Supplement Table 1. Data Analysis Methodology

| **Steps** | **Process** | **Timeline** |
| --- | --- | --- |
| 1. Pilot/refine/calibrate analytic tools:   – Summary template  – Analysis matrix | - Draft tools, and assess face validity via full analysis team review (i.e., do these tools capture what we’re trying to capture?). - 2 primary analysts work in tandem to populate tools using 1st interview transcript, note key decisions and points of disagreement, edit tools as needed - The same analysts work in parallel to populate modified tools using a 2^nd^ transcript, then meet to review results, resolve discrepancies, generate final tools and analytic definitions, rules, and guidelines for populating them. | After 2 interviews are complete and transcribed |
| 1. Pilot/refine/calibrate analytic process | - Primary analysts tandem code 1 new transcript (not used in step 1) using the final tools, discussing and taking notes on decisions being made throughout. - Then the same analysts parallel code a 2^nd^ transcript (also not used in step 1) and meet to review with the third analyst. - The third analyst determines the need for more parallel coding and calibration (minimal discrepancies=no, more than minimal=yes). | Once 4 interviews are complete and transcribed |
| 1. Analyze interviews | - Once tools and processes are final, all transcripts, including the ones used in steps 1-2, are prepped for analysis. - The two primary coders are each assigned two transcripts, they code in parallel (i.e., populate the template and then the matrix). - The full analytic team meets to confirm and finalize the matrices, by 1) reviewing each matrix for alignment with analytic definitions, rules, and guidelines to finalize, 2) resolving any discrepancies through discussion with a third analyst (act as tie-breaker), and 3) consolidating the matrices, grouping like responses within each cell to develop sub-codes (predefined and emerging). - As new sub-codes emerge, the team will develop definitions, rules, and guidelines for how to apply them and continually review prior coding decisions to confirm they align with this emerging understanding. - Repeat rounds of coding and consolidation (~3, 1 per week) until all transcripts are analyzed. - The full analytic team meets to review the final matrix, including final sub-code groupings and definitions. Once the matrix is confirmed final, the team will develop descriptive statements to interpret and summarize the themes represented in each matrix category and select 1-2 quotes illustrating each key message or concept. These elements will form the basis of an internal report and other analysis products. | Once all interviews are complete (expected n=7) |

Note: Interview recordings and notes were referenced during any step as needed (e.g., to resolve discrepancies), but will not be formally or holistically reviewed in addition to the transcripts. A slightly modified version of the above was used for focus group analysis.
