# Supplementary material for: Exploring barriers and facilitators of implementing an at-home SARS-CoV-2 antigen self-testing intervention: The Rapid Acceleration of Diagnostics—Underserved Populations (RADx-UP) initiatives
Source: PLoS One. 2023 Nov 16;18(11):e0294458. doi: 10.1371/journal.pone.0294458 (PMC10653400; doi:10.1371/journal.pone.0294458)
Supplement: S1 Dataset — (ZIP) [file pone.0294458.s002.zip › CA-FG_09_29_POST-INTERVIEW REFLECTION MEMO.docx]

**CA FG -POST-INTERVIEW REFLECTION MEMO**

**Interviewer(s) Name**: Amelia Defossset

**Date of Interview**: 9/29/202

**Memo Date (if different from interview date)**: 9/30/2022

*** * ***

**REFLECTION QUESTIONS:**

*The purpose of this document is to serve as a reflection guide. You can answer the questions that resonate most with your interview experience, you do not need to answer every question. The research team may use your responses to refine the interview protocol, interview process, and/or inform data analysis.*

***Please report any urgent reflections or concerns to your research team immediately, as compliant with the IRB.*** *(e.g., protocol deviation that harmed a subject or placed subject at risk of harm, breach of confidentiality, unintended effects, unresolved respondent complaint, etc.).*

| **INTERVIEW PROCESS**   1. **How did you feel about the interview?** It went well overall. Participants were engaged and had a lot to say 2. **What went well?:** Participants knew each other 3. **Any frustrations?** It was hard to keep participants on track due to familiarity with one another. 4. **Anything unexpected that may have impacted the interview?** No |
| --- |

| **TAKEAWAYS**   1. **Did you obtain the kind of information you expected?** No 2. **What were your key takeaways from the interview?**     1. Frustrations with academic partners around communications, study design (trying to get participants fill out surveys) |
| --- |
| - 1. **Conversation emphasized trust in known community network** |

| **INTERACTION**   1. **What was your interaction like with the interview participant? (Your/the participant’s behavior, body language, mood, or disposition).** Participants were engaged. One participant was really frustrated with the academic partners than others. 2. **Was the person you interviewed cooperative and helpful, or something else?** Most participants were cooperative although one seems a little hesitant to provide further explanation on a thought. |
| --- |

| **UNANSWERED QUESTIONS**   1. **Did you get to all the interview questions? If not, which questions did you not get to, why?** No, participants were talking to each other a lot and some questions were already addressed in previous answers. 2. **Any responses from the participant that were incomplete or weren’t as clear as you wanted them to be? Please describe.**     1. Some responses were not clear as participants were not clear when they speak about the program versus their overall experience with COVID. 3. **Any questions that the participant did not understand? Did not feel comfortable answering? Needed rephrasing?** No 4. **Any questions that you wished you asked? These might be questions that were not in the interview guide.** No |
| --- |

| **PATTERNS/CONNECTIONS**   1. **If you’ve conducted other interviews, are you noticing any emergent patterns or insightful connections?**     1. Mission alignment: organizations were already serving the community, and this aligned with the project goals.    2. Trust: organizations being approached by someone they trusted and already know. |
| --- |

| **ASSUMPTIONS/BIASES**   1. **Were any of your personal assumptions/biases about the research, the participant, or some other aspect challenged as part of this interview?** The notion that academics conduct studies for their own research purposes and not for the benefits of the community. |
| --- |

| **OTHER REFLECTIONS**   1. **Use this space for anything else you’d like to share as part of this reflection.** |
| --- |

**Thank you!**
