# Supplementary material for: Exploring barriers and facilitators of implementing an at-home SARS-CoV-2 antigen self-testing intervention: The Rapid Acceleration of Diagnostics—Underserved Populations (RADx-UP) initiatives
Source: PLoS One. 2023 Nov 16;18(11):e0294458. doi: 10.1371/journal.pone.0294458 (PMC10653400; doi:10.1371/journal.pone.0294458)
Supplement: S1 Dataset — (ZIP) [file pone.0294458.s002.zip › CAFG-Notes (09.03.22).docx.docx]

**CA FG Notes**

4.Merced County had higher level of focus on the situation at hand, and paid attention to the public’s health.

5. California Department of Health, University of California, San Francisco and UC Merced

6. One participant mentioned that they couldn’t judge but the organization follows legal and state mandates

11. Challenges or Barriers

Capacity: Stipends to for local community partners to increase participation

Distribution

Time frame

12. School nursing staff helped organized, distribute and inform parents about the process

Network of community health workers in the county were helpful: voluntary work

Patient referral: Readiness of the community and collaboration with community members.

13. Goal: To test families as fast as possible

To bring needed resources for people to know their status to mitigate the virus.

Provide access to testing.

Participants believed that the project was successful but for a short period of time.

14. People needed to know to

Parent wanting to get quick results.

15. Interest to know their status

Staff members and parent were concerned about other family members and wanted to know to protect them.

Anxiety

Convenience

Exposure to a positive person

16. Ensuring resources including capacity and language services were provided by partnering with other organizations.

Distribution to every child

Available numbers to call

Communication

17. Provided more attention to COVID-19

18. Unusual: needed planning and some thoughts.

Not an organization that provided direct services but need to be part of the solution

20. Results of the survey related to the test kits should be shared.

23. Having test kits for all child served

24. Access and restocking of the test kits.

26. Participants answered affirmatively saying “absolutely’. Reasons stated were related to access and equity.

Debriefing

1. Overall participants were positive, responsive and engaging.

3. Not all participants showed up.

4. No

5. Yes

6. Participants seemed passionate about their work, one of the reasons they were happy to join the projects. Participants mentioned the short time frame of the project.

7. Participants were attentive and engaging

8. Participants were cooperative and helpful

9. Yes

10. No

11. No

12. No

13. Short time frame of project, Distribution problem.
