# Supplementary material for: Exploring barriers and facilitators of implementing an at-home SARS-CoV-2 antigen self-testing intervention: The Rapid Acceleration of Diagnostics—Underserved Populations (RADx-UP) initiatives
Source: PLoS One. 2023 Nov 16;18(11):e0294458. doi: 10.1371/journal.pone.0294458 (PMC10653400; doi:10.1371/journal.pone.0294458)
Supplement: S1 Dataset — (ZIP) [file pone.0294458.s002.zip › CAFG-Notes (09.29.2022).docx.docx]

Notes

1. **Things liked about Merced County**:

Positive- Weather but on the downside can be very hot with high temperatures.

-Good county collaboration

1. **Things disliked about Merced County**: Healthcare: not a lot of healthcare specialists (just two ophthalmologists). Bad transportation.
2. **Response to COVID-19**

Vaccination, testing outreach was provided.

Mobile testing in multiple location

Testing availability

County reached out to COVID programs that enhanced testing.

1. **Initial communication**

Different connections in the community

Through United Way

1. **Immediate thoughts**

Wanted to make people do the tests and get vaccinated

Aligns with what they were doing before (already doing)

It was an additional task to the ongoing task (one more thing to the plate)

Concerns about finance

1. **How if at all, did the process or idea of working with a community group that brought together several community leaders influence your decision to participate.**

Already involved in meeting the needs of the community, knowing the community, and the contact.

1. **Meet your county COVID needs:**

Did not meet the county’s needs: -one participant agreed to this.

Client was not able to complete survey. Spanish material came late and were inconsistent. Another participant agreed to this saying that surveys had to be printed. Also, a lot of steps were not available for the blinds-They had to do sessions on using test kits.

1. **Barriers to implementing program:**

Storage: Two participants agreed that they did not have a storage.

Families go to different sites and sell test kits (another participant agreed with this).

1. **What helped accomplish project task**

Volunteers

Connection to state programs that gave out masks and sanitizers

Food distribution that was ongoing at the same time

Mobile van -was great for advertisement

1. **Was project successful?**

Distributed a lot of kits

Success in getting kits

Creating awareness

1. **Factors that drove participants to pick up test kits**

Frequent use of public transportation

People did not want to infect others

Seeing others do it

Protect families-common in Latino families with big families

For paid time off

1. **For gatherings How did your tasks for this project compare to your normal activities with the organization that you are presenting?**

One participant said it was routine-not harder or easier. Another participant said it wasn’t as there are not a direct service provider and it was out of the norm.

Level of difficulty

Participant with a larger organization said it was not that difficult. Another participant said it was a little bit easier, but they had some financial concerns.

1. **Perception on communication**

Communication from the community partner was good but was not from Duke. It was overwhelming from Duke and a premeeting with community partners would have been helpful. There was confusion as to what to do.

1. **Recommendations/thoughts about the project**

The lead team lacked insight on how things worked in the community; a community advisor should be on the lead team.

There were no explanations as to why Duke instead of the University in the state is the lead for the project: all participants nods in agreement.
