# Supplementary material for: Exploring barriers and facilitators of implementing an at-home SARS-CoV-2 antigen self-testing intervention: The Rapid Acceleration of Diagnostics—Underserved Populations (RADx-UP) initiatives
Source: PLoS One. 2023 Nov 16;18(11):e0294458. doi: 10.1371/journal.pone.0294458 (PMC10653400; doi:10.1371/journal.pone.0294458)
Supplement: S1 Dataset — (ZIP) [file pone.0294458.s002.zip › Interview reflection questions.docx]

**POST-INTERVIEW REFLECTION MEMO**

**REFLECTION QUESTIONS:**

*The purpose of this document is to serve as a reflection guide. You can answer the questions that resonate most with your interview experience, you do not need to answer every question. The research team may use your responses to refine the interview protocol, interview process, and/or inform data analysis.*

***Please report any urgent reflections or concerns to your research team immediately, as compliant with the IRB.*** *(e.g., protocol deviation that harmed a subject or placed subject at risk of harm, breach of confidentiality, unintended effects, unresolved respondent complaint, etc.).*

| **INTERVIEW PROCESS**   1. **How did you feel about the interview?** 2. **What went well?** 3. **Any frustrations?** 4. **Anything unexpected that may have impacted the interview?** |
| --- |

| **TAKEAWAYS**   1. **Did you obtain the kind of information you expected?** 2. **What were your key takeaways from the interview?** |
| --- |

| **INTERACTION**   1. **What was your interaction like with the interview participant? (Your/the participant’s behavior, body language, mood, or disposition).** 2. **Was the person you interviewed cooperative and helpful, or something else?** |
| --- |
| **UNANSWERED QUESTIONS**   1. **Did you get to all the interview questions? If not, which questions did you not get to, why?** 2. **Any responses from the participant that were incomplete or weren’t as clear as you wanted them to be? Please describe.** 3. **Any questions that the participant did not understand? Did not feel comfortable answering? Needed rephrasing?** 4. **Any questions that you wished you asked? These might be questions that were not in the interview guide.** |

| **PATTERNS/CONNECTIONS**   1. **If you’ve conducted other interviews, are you noticing any emergent patterns or insightful connections?** |
| --- |

| **ASSUMPTIONS/BIASES**   1. **Were any of your personal assumptions/biases about the research, the participant, or some other aspect challenged as part of this interview?** |
| --- |

| **OTHER REFLECTIONS**   1. **Use this space for anything else you’d like to share as part of this reflection.** |
| --- |

**Thank you!**
