# Supplementary material for: Exploring barriers and facilitators of implementing an at-home SARS-CoV-2 antigen self-testing intervention: The Rapid Acceleration of Diagnostics—Underserved Populations (RADx-UP) initiatives
Source: PLoS One. 2023 Nov 16;18(11):e0294458. doi: 10.1371/journal.pone.0294458 (PMC10653400; doi:10.1371/journal.pone.0294458)
Supplement: S1 Dataset — (ZIP) [file pone.0294458.s002.zip › NC.FG-Notes 09.21.docx.docx]

#2 Focus Group

1. Access to university, medical schools, livable income from employers. Largest county in eastern NC, looked up to on the outside. Work with other counties that may not have the same services. Urban area, accessible resources
2. Always room for improvement, improve the school systems that are struggling.
3. Strong partnerships, logistically everything worked and made sure they were accessible to the community. Advocated for the rural area to help the entire county
4. Other counties didn’t have the same relationships and sacrifices from partnerships. Had a strong participation and commitment to the goal. Shutting down the county and some other places stayed open
5. Trusted the person who reached out and felt his would be a good opportunity to be engaged. Positive, excited, led by a professional. A way to help people directly. Had a strong background in public health and felt like they could help effectively
6. Get the community involved and reach out to resourceful and like-minded
7. Gave the community the most information in a fast manner. Slow start, once it got started it went well. People came around and decided they wanted to get the tests when before they didn’t
8. Before program ended, some people still didn’t trust but did after it ended. Sometimes trusted too late because then they didn’t have the kits
9. Delivery issues at first. Post office, fedex, delivery times aren’t reliable or reasonable
10. Social media – used facebook to show that people were getting and using the kits. Advertise kits on certain platforms, cover as many different entities as they could. Food trucks were successful.
11. Hesitant about participant in this (community members) so she told them that the health director is supporting it
12. A lot of people started getting covid, people were getting educated and found out how serious it could be
13. Wanted to be safe when around family and coworkers, learned more about the kits
14. Concerned that they wouldn’t hear the outcome of the tests kits
15. Did not totally because she is retired and worked with every aspect of public health, so she already had those resources
16. Enhanced and took to new level of supporting the community. Changes in the community and helping people to stay alive and healthy. Activities helped increase trust between facilities and community partners
17. Couldn’t have been any better with communication
18. Wish that it could’ve lasted a little longer. Drug stores ran out of tests and people wanted tests from them. Cost of kits were expensive and abusing the system. Liked the kits from SYCT better than the ones from government
19. Mildred was very influential in getting people involved
20. Helpful in set up, needing signs and marketing materials
21. Learned a lot about covid 19 and enhanced her knowledge and skill set.
22. Yes, prevention is worth it. Need in the community which was met.

Debrief

- Not understanding the questions entirely
- Forgetting the other half of the question
- Gave good information about specific areas
- Spoke a lot about their backgrounds
- Challenging to keep on track somewhat
