# Supplementary material for: Exploring barriers and facilitators of implementing an at-home SARS-CoV-2 antigen self-testing intervention: The Rapid Acceleration of Diagnostics—Underserved Populations (RADx-UP) initiatives
Source: PLoS One. 2023 Nov 16;18(11):e0294458. doi: 10.1371/journal.pone.0294458 (PMC10653400; doi:10.1371/journal.pone.0294458)
Supplement: S1 Dataset — (ZIP) [file pone.0294458.s002.zip › NCFG-NotesNotes (8.25.22).docx [Christina].docx]

NC Focus Group

1. Born and raised in North Carolina – friendliness, experience of all 4 seasons, the wages vs the cost of living. Very active and can go to the mountains and the city – easy access to cultural thing. NC has more access than VA. The weather is nice and overall variety. Lives in a small town and enjoys it. Proximity to Raleigh. Like the climate and loves the people and feels safe. Can be active in the community because it a smaller community vs a big city
2. The school systems may not be strong enough for the teachers and students to do well. Teachers don’t feel supported. NC doesn’t get the national recognition it deserves. The economic role – manufacturing base to service base. Lack of manufacturing and opportunities for people not going to college.
3. By the time they got the resources she felt it was almost over. Education around covid and lack thereof, reflected the tests being taken. Educate before passing out the test. Push back of wearing masks was disappointing.
4. From a healthcare perspective, we were “heroes” and supported. 2^nd^ year people pushed back harder with mandatory things such as testing, felt different from the start and second wave of it. Not happy about pushing more resources onto them. Ended up not feeling supported.
   1. Testing options – drive thru site and test distribution. Relatively easy to get tested and available. Above average.
5. Does own research/reading of articles and listen to other health professionals. Daily updates from government office in those broadcasts. Reads newspaper to get information. Med- twitter. Infectious control committee at ECU. Chat between health directors and committees at the schools across the state
6. Data, officials speaking about it (Participant 33), Conflict in opinions concerning the news (Participant 36), governing bodies and the information they share out and use. Data from other schools that are similar in size and geographical. Trends in similar areas.
7. Excited to be part of the project. Encourage people to be testing and trying to help the community. Wish they still had the tests to distribute. Distribution went well.
8. Helped with what they needed at the time. Only took 15 minutes at home vs. an hour+ of time to go to the testing site and be tested. Met the needs of the participants well.
9. Couldn’t get enough tests. Distribution of the tests. Getting the tests from where it they were stored. Transition and methods for distribution made it hard to get tests.
10. Kits available to community events. Relationships with other community members and organization to distribute test kits to.
11. Early identification of people with covid – preventative measure so it doesn’t spread
12. Very effective. Indication from other people. Having the test its distributed and taken/used
13. Easy and free. People dying who they knew. Fear, not understanding how symptoms varied. Convenience.
14. Testing before seeing elderly people. Healthcare fields. Not wanting to be the person who spread it. Higher risk individuals. Theater students who go off campus more/don’t wear masks during performances.
15. Advertisement for free tests. Events passing out free tests. Good use of media.
16. Aligned with daily tasks as a community official. Involved in the community and providing information already.
17. Didn’t have a choice but would’ve done it willingly. Advocate for people who cannot speak and connecting people who don’t have connections. Educate and provide for the people we live around
18. Severity of the problem (covid) people dying. Affecting the community, kids out of school and people not working. Willing to grab onto anything that may be helpful.
19. Clear communication and willing to use the platforms that she was comfortable with.
20. If the coordinators got in contact sooner and there wasn’t a leadership transition. Change in leadership made things a little harder
21. Would participate going forward and participate again. Always looking to increase access and preventative measures. Always being asked if they have more test kits for all types of diseases.
22. Advocacy, increasing access and less barriers
23. More programs like this in the future, increases access to anything that will help the community.
24. Was told the shot affects women’s fertility

Debrief

- Only 3 people showed up
- All the participants seemed to have similar answers/agreement
- Academic slightly different perspective
- Felt a little rushed at the end
- Didn’t ask as many questions about communication because of time
- Skipped question that were kind of repetitive
- Question 1-6, aren’t too relevant don’t need that much time spent
- Disruption going from his house to his car and made us feel less like we wanted to engage him
- Notetaker change their name/ID
- Check about IRB again if we said we won’t use people’s name
- Gotten richer data if they talked to each other more and built off each other responses
