# Supplementary material for: Exploring barriers and facilitators of implementing an at-home SARS-CoV-2 antigen self-testing intervention: The Rapid Acceleration of Diagnostics—Underserved Populations (RADx-UP) initiatives
Source: PLoS One. 2023 Nov 16;18(11):e0294458. doi: 10.1371/journal.pone.0294458 (PMC10653400; doi:10.1371/journal.pone.0294458)
Supplement: S1 Dataset — (ZIP) [file pone.0294458.s002.zip › PID(3)-Notes (8_16_22).docx [Christina].docx]

1. Retired from health department, non-profit, serves on many boards in the community, department of social services, mental health and addiction. Mission of her organization is to help women and girls.
   1. President of the organization – advocacy, non-profit and 501c
2. Didn’t go willingly into the project, asked to represent church and ended up staying with the project to distribute kits, touched the smaller groups of people rather than larger corporations
3. Extremely successful because they were at the grassroot level, and most of the pople involved in the project had connections. They had the kits and were trustworthy, so people were more likely to accept the tests. “blacks used as guinea pigs for new drugs and kits” so they felt safe taking from the community who distributed the tests and look like them.
   1. We were able to help the county as a whole
4. Members of the church involved, asked Minerva to collect the kits and her community was calling to receive them
5. Communication method was best approach. Prioritized this project and trusted Mildred.
6. Minerva works with the whole community, but tends to work more with the African American community. Easy to buy into this project because it benefitted the community and specifically the African American people
   1. Mildred reached out because of her church
   2. National council for negro women
   3. More than one way she got involved because of connections
7. Aligned very well. Easy to pull groups together to collaborate.
8. The kits were at a certain location, Minerva called and requested a certain amount of kits and went to the site to pick them up without getting out of the car. Food boxes were given out while distributing kits. Combined all resources together to help the community
9. Do as much for people as possible. Test kits distributed as grocery stores and could also get a vaccine distributed by a nurse there. Nurses at different sites was simple and helpful
10. No questions, everything was self-explanatory. Felt that is she had questions she could have them answered. Health director was involved. Comprehensive approach to give out masks, test kits, education and different layers of people.
11. Everyone in their county was working together because they had the same goal. County level – information got out about appointments, etc.
    1. *LEVEL OF TRUST AND IDENTITY BETWEEN COMMUNITY*
12. Prior to covid, housing issues. During covid, it may have gotten better because they could house more people and fund hotel rooms. (Community shelter). Internet improved as well
13. They were afraid and suspicious of the shot. Transportation issues. Misunderstanding of the vaccine is safe and good.
14. Yes, because some of the places had events. Sites were set up where people were constantly going.
15. Doesn’t know if she can really answer that. Get people to use the kit. End of project – value and wanted them
16. Not difficult, not on her agenda to do but wanted to after she picked up the kits
17. Saw the value in the kits and wanted them
18. Just not having the info but once the kits were distributed people saw the value
19. Not at first aware of online version
    1. Picking them up preferred

Debriefing:

- Sometimes answers were not related to the questions
- Answers were consistent
- Focused throughout the interview and was engaged
- Overall, a good interview and reliable
