# Supplementary material for: Exploring barriers and facilitators of implementing an at-home SARS-CoV-2 antigen self-testing intervention: The Rapid Acceleration of Diagnostics—Underserved Populations (RADx-UP) initiatives
Source: PLoS One. 2023 Nov 16;18(11):e0294458. doi: 10.1371/journal.pone.0294458 (PMC10653400; doi:10.1371/journal.pone.0294458)
Supplement: S1 Dataset — (ZIP) [file pone.0294458.s002.zip › PID6 interview notes.docx]

**Participant 6 In depth Interview (notes from responses to questions in the interview guide)**

General appearance: Participant appears to be attentive, engaged, and present with no distractions

Question 1: resource connection

Question 2: Founder of the organization

Question 3: *clarifies question*The project goal aligns with organization’s previous work

Question 4: Overall positive response

Question5: Response to question was unclear. Positive response about communication across project. *Participant nodded head and smile while mentioning this*

Question 6: Participant clarified question and mentioned the relationship with people implementing the project made his organization participate.

Question 7: Participant affirms that task aligns with organization’s usual activities of helping people*participant smiles when listing the work, they do*. Interviewer confirmed response.

Question 8*participant clarified questions*, mentioned that they already had resources which made the task easy.

Question 9: participants mentioned that the only questions/problems they had was with test kits delivery as there was no amazon delivery in their location. He mentioned that overall project team was responsive to both needs and questions.

Question 10: *clarifies question* *sounds impressed* stated that “that is what is needed” emphasizes “long lines to get tested” and says that getting the test kit is “very expensive in a high tone”

Question 11: As regards barriers to COVID -19 testing, stated that the test kits were explanatory and talked about increase spread after the project stopped.

Question 12: Participant mentioned that he was asked to maintain the test kit warehouse and that the task was “very simple” with emphasis and says it was “like ABC”

Question 13: Participants went on to explain his own preference for instore pick ups

Question 14: Participants reiterated the need for project continuation*serious and concerned demeanor*. The need for one warehouse for test kits that they can pick up test kits instead of deliveries.

**Debrief (using the debrief template)**

Both interviewer and note taker agreed on the following:

**INTERVIEW PROCESS**

-Participant was present and engaged

- The participant seems to have been very involved in the project and was able to provide valuable insight

-Question 13 feels confusing, there might need to be an introduction texts about the different ordering methods in the organization. Participant was talking about his own preference for orders

-There were no frustrations during the interview, though participant may have thought interviewer and note taker were part of the project implementation team hence the reason for reiterating project continuation

**TAKEAWAYS**

- **Project tasks were in line with what organizations do.**
- **Things organizations do normally have better processes**
- **Organizational versus individual tasks: We might not be getting the organizational experience if people were asked to do different things within the same organization (possibly probe about roles)**
- **Challenges: No amazon delivery**
- **Timing: Project should have continued.**

**INTERACTION**

Participant was willing to share his experience, positive with occasional laughter.

Cooperative and helpful

**UNANSWERED QUESTIONS**

-Question 13 on the interview guide might need clarification so that participant can provide appropriate response.

-Participant clarified a few questions (questions 3, 8, 10)

-Participant did not provide appropriate response for the question on “initial communication method”. Maybe helpful to add probes with options (email, phone etc)

**PATTERNS/CONNECTIONS**

-Organization’s mission aligning with project task
