# Supplementary material for: Exploring barriers and facilitators of implementing an at-home SARS-CoV-2 antigen self-testing intervention: The Rapid Acceleration of Diagnostics—Underserved Populations (RADx-UP) initiatives
Source: PLoS One. 2023 Nov 16;18(11):e0294458. doi: 10.1371/journal.pone.0294458 (PMC10653400; doi:10.1371/journal.pone.0294458)
Supplement: S1 Dataset — (ZIP) [file pone.0294458.s002.zip › PID8 interview notes.docx]

Interview #1 (participant 8?), Amelia notes 8.1.22, 2-3pm

Interviewee was sitting close to the camera and slightly skewed in the frame throughout the interview. At first, she seemed a bit distracted, e.g., not always looking at the screen as she was talking. As the interview went on, she seemed to focus her attention more on the exchange and was almost always looking into the screen and seemed engaged.

The intro prompt indicated the conversation would last for about 30 minutes and several times throughout she said she was trying to stay on track in terms of time. I reminded her we had scheduled an hour and so she didn’t need to be mindful of time in crafting her responses.

She was quick to respond to questions and largely didn’t ask for clarification.
