# Supplementary material for: Exploring barriers and facilitators of implementing an at-home SARS-CoV-2 antigen self-testing intervention: The Rapid Acceleration of Diagnostics—Underserved Populations (RADx-UP) initiatives
Source: PLoS One. 2023 Nov 16;18(11):e0294458. doi: 10.1371/journal.pone.0294458 (PMC10653400; doi:10.1371/journal.pone.0294458)
Supplement: S1 Dataset — (ZIP) [file pone.0294458.s002.zip › PID8 interview notes.docx]

1. United Way several types of funding – community health
2. Run the program at United Way
3. Local non-profit, she has a lot of community involvement, Merced county is well connected with each other “melting pot” working with different cultures
4. The test kits helped when there was a shortage and was “extremely helpful”, saw the lack of knowledge while running the program
5. The United Way – unknown, brought in after recruitment, community had farmers and people of Asian descent?
   1. Issues with “playing telephone” with Duke Clinical and CCPH
   2. Lack of communication in beginning of project, felt like “no one was listening”, kits coming in when events were cancelled
   3. The meetings were at an inconvenient time due to their schedule
6. Anything that is available to help the community they will do, smaller community/county that takes care of each other
   1. Community outreach, map out plan/model
7. The united way normally does funding of projects but this project made them use community partners who already use community outreach to make the project happen, “boots on the ground”
8. Take/acquire storage facility, people to operate equipment of pallets, school system let them use employees and facilities, part-time employees to help arrange pictures, social-media, purchases insurance for drive thru’s, offer incentives for non-profits, volunteers for covid vaccine clinics
   1. LED light truck “You & Me, COVID Free” – used in parade to promote, provided with distribution truck (food truck) – used more for advertising, supplied flags but they were stolen, advertising
9. No question – more clarification
   1. Duke good at getting back to her with question
   2. CCPH took a few days to respond
10. Yes – county was able to track everything and where the tests went
    1. Set up test distribution events in different spots/territories
    2. Education and tests kits in that specific county was needed
11. A lot of farm workers, meat and produce packers. Workers can’t take off and will continue to go to work if they don’t know for sure they have it, worried they will be replaced/lose job if they got sick and didn’t work
    1. Yes, the non-profits hand were hand-picked because of being “heroes” – looked up to in their culture/community not government person
12. Matched perfectly, scope of work loose, get 200,000 kits out before the date and saturate the county which they did
    1. Tasks were not difficult at all
13. People were still getting together but wanted to take precautions and tests before getting together, highest rates in CA
    1. Anti-vaccine people were using the kits eventually
    2. Online ordering just started
14. No recommendations – issues were resolved in the moment and felt heard
15. “You and Me Healthy” went live, getting info out to the community, through CCPH and Duke Clinical, next step after Y&MCF project. Provide info to the public for healthier living
    1. Ex: monkey pox info, diabetes, etc.

Debrief:

- Interviewee seemed confused about Say Yes Covid Test and You & Me Covid Free. There was confusion about if those projects are the same or totally different
- Note taker and facilitator think that creating a FAQ about both programs (YMCV and SYCT) may be helpful to provide to the interviewee if they are confused or have questions
- There was confusion about Ordered kits vs in-person distribution. Seemed like Janet didn’t know about the ordering part of it until recently so that wasn’t an option when they were distributing the kits
  - Find out if that was an option first because she seemed confused
- Facilitator is Assuming there is documentation about each program the communities developed and is wondering if we need to ask that because it took up a bit of time and doesn’t need to be repeated?
- Janet’s responses regarding the communication between each part was contradictory. At first said communication wasn’t strong but at the end said the communication lines were good.
- ***Facilitator and note-taker agree on all of these points***
