# Supplementary material for: Exploring barriers and facilitators of implementing an at-home SARS-CoV-2 antigen self-testing intervention: The Rapid Acceleration of Diagnostics—Underserved Populations (RADx-UP) initiatives
Source: PLoS One. 2023 Nov 16;18(11):e0294458. doi: 10.1371/journal.pone.0294458 (PMC10653400; doi:10.1371/journal.pone.0294458)
Supplement: S1 Dataset — (ZIP) [file pone.0294458.s002.zip › PID8 interview transcript.docx]

9

00:02:33.120 --> 00:02:33.810

Interviewer: hi there.

10

00:02:34.770 --> 00:02:37.170

Interviewer: Hello hi how are ya.

11

00:02:38.190 --> 00:02:40.710

Participant: i'm here that's about all I can say.

12

00:02:42.690 --> 00:02:51.540

Interviewer: Well, glad to have you here with us today i'll just quickly introduce the folks on the call, so my name is amelia and i'll be facilitating the

13

00:02:51.990 --> 00:03:11.910

Interviewer: interview today i'm and i'm joined by Christina who's going to be taking notes, I wanted to let you know that a colleague of ours Donaldson might also join the call um he just will also be facilitating interviews and when it to listen in to shadow the process um but if it's.

14

00:03:13.590 --> 00:03:17.070

Interviewer: I guess that's just based on your permission and comfort level if you're.

15

00:03:17.490 --> 00:03:18.570

Participant: It doesn't matter to me.

16

00:03:19.200 --> 00:03:26.100

Interviewer: Okay, great so if you're okay with that he will be joining shortly, but we can go ahead and get started.

17

00:03:27.090 --> 00:03:34.740

Interviewer: um so just a reminder today's interview will be a little bit more formal than a regular conversation.

18

00:03:35.310 --> 00:03:46.140

Interviewer: So I have a series of questions to ask you and i'll need your help to stay on course, so that we can get through all of them were scheduled for an hour, I think that will be okay to stay within that time.

19

00:03:47.550 --> 00:03:55.320

Interviewer: we're talking to are interviewing individuals of various positions and professions that are part of the planning Community advisory team.

20

00:03:56.130 --> 00:04:01.140

Interviewer: and your role in recruiting and securing Community organizations was a primary function of this project.

21

00:04:02.070 --> 00:04:14.550

Interviewer: So, so therefore it's really important that I asked you each of the questions as written, of course, if you don't understand a question, or if you need me to repeat it, or you have any need for clarification just let me know.

22

00:04:15.720 --> 00:04:21.150

Interviewer: feel free to jump in anytime and as a reminder to for your time you'll receive a $100 gift card.

23

00:04:23.640 --> 00:04:33.090

Interviewer: So again, thanks for agreeing to talk with us today, this interview will last approximately 30 minutes so again more scheduled for an hour, but anticipating 30 minutes.

24

00:04:33.900 --> 00:04:48.870

Interviewer: i'll ask you questions about yourself your knowledge and understanding of your counties health needs health priorities available resources and gaps and resources or other access related issues there aren't any right or wrong answers today.

25

00:04:49.980 --> 00:04:55.590

Interviewer: If there are any questions that you'd rather not answer feel free to decline to answer, and we can just move on.

26

00:04:57.150 --> 00:05:04.920

Interviewer: And all of your responses will be kept confidential so, meaning that only the research team will know what you responded.

27

00:05:05.730 --> 00:05:16.440

Interviewer: And then, when we summarize the results and report them out we're going to report across all of the participants together, so no individual names of specific participants will be used without their permission.

28

00:05:17.700 --> 00:05:22.710

Interviewer: And then Lastly, as I mentioned, or as we mentioned in the informed consent.

29

00:05:24.120 --> 00:05:37.140

Interviewer: We would like to record this interview today and will keep the your private information, separate from the recording and will destroy the recording after they're transcribed so do we have your permission to record the interview.

30

00:05:37.890 --> 00:05:43.050

Interviewer: Yes, okay great um any questions before we jumped in.

31

00:05:43.980 --> 00:05:45.900

Participant: To exactly which program is this for.

32

00:05:46.500 --> 00:05:48.720

Interviewer: So this is for this say yes covid test.

33

00:05:49.740 --> 00:05:50.130

Participant: Okay.

34

00:05:52.200 --> 00:05:53.040

Participant: we've got a couple of.

35

00:05:53.130 --> 00:05:54.540

Going so it's kind of like.

36

00:05:56.100 --> 00:05:58.170

Interviewer: yeah no fair and thank you for.

37

00:05:58.410 --> 00:06:00.660

Participant: For us it was you and me covid free.

38

00:06:01.530 --> 00:06:11.790

Interviewer: Okay, all right i'm gonna i'm gonna say you say yes covid test throughout but um if that's helpful, I can I can try you me covid free.

39

00:06:12.660 --> 00:06:21.660

Participant: But you and me covid free there were just some changes made as far as advertisement and promotion stuff.

40

00:06:22.740 --> 00:06:25.170

Participant: That the others didn't have access to.

41

00:06:26.100 --> 00:06:27.360

Interviewer: Okay okay great.

42

00:06:28.470 --> 00:06:34.080

Interviewer: um so first i'm going to ask you some questions about your role at your organization.

43

00:06:34.920 --> 00:06:38.820

Interviewer: But before that can you just find the primary mission of your organization.

44

00:06:40.500 --> 00:06:55.890

Participant: So at that time I was working with the united way and the united way we do several different types of funding and one of them, which this program fell into was Community health.

45

00:06:58.020 --> 00:07:02.640

Interviewer: Okay, and um What was your position, at united way?

46

00:07:03.990 --> 00:07:07.620

Participant: The united way brought me in especially to run this program.

47

00:07:09.150 --> 00:07:09.600

Interviewer: Okay.

48

00:07:10.800 --> 00:07:17.730

Interviewer: um, and so the next set of questions about your organization and the say yes covid test project.

49

00:07:19.260 --> 00:07:25.110

Interviewer: So, in your opinion, what made you or your organization, a good partner for the project.

50

00:07:27.210 --> 00:07:34.590

Participant: Okay, the united way, with it being a local nonprofit we're downtown have a lot of.

51

00:07:35.640 --> 00:07:52.110

Participant: People agencies other nonprofits are well aware of the united way also they brought me on to run the project, because I have got a lot of Community involvement.

52

00:07:54.780 --> 00:07:57.600

Participant: That was to remind me, so I would not miss this appointment.

53

00:07:58.050 --> 00:07:58.440

Okay.

54

00:08:00.210 --> 00:08:05.040

Participant: So I yeah I hopefully I turned off now so anyhow.

55

00:08:06.750 --> 00:08:13.140

Participant: But it was because Merced, we are so we're very well connected with each other.

56

00:08:14.040 --> 00:08:30.720

Participant: And we have got we're basically a huge melting pot, and we work with the different organizations within the different Community breakdowns of, be it through culture i'm going to say culture, not so much race, but culture.

57

00:08:33.000 --> 00:08:33.450

Interviewer: Okay.

58

00:08:34.710 --> 00:08:42.480

Interviewer: um and how do you feel about the say yes covid test projects ability to address your county's covid 19 needs.

59

00:08:44.910 --> 00:08:54.330

Participant: Well, for us it was super super cool because the test kits we the entire country was out of test kits.

60

00:08:55.230 --> 00:09:00.480

Participant: People were getting ready to go on break from school for thanksgiving and Christmas.

61

00:09:01.050 --> 00:09:09.960

Participant: And those kits came in at the perfect time, not only because of the holidays, but because of the shortage country wide on the test.

62

00:09:10.500 --> 00:09:22.350

Participant: So it was extremely helpful we even got to a point where people were calling us and asking us, I have a positive test now do I have to report it to somebody at it, we just we saw the lack of knowledge.

63

00:09:23.370 --> 00:09:25.050

Participant: While running the Program.

64

00:09:25.890 --> 00:09:28.860

Interviewer: Right great um OK.

65

00:09:30.450 --> 00:09:40.380

Interviewer: And now i'm going to ask a couple questions about the say yes covid test program specifically so, can you describe how you were initially contacted about this program.

66

00:09:42.210 --> 00:09:54.900

Participant: At the united way I don't know exactly how that came about, I was brought in, after the fact, approximately two days, but I was told that because of the.

67

00:09:56.160 --> 00:10:05.280

Participant: We have got a lot of Farmworkers up in our Community, as well as a lot of immigrants, a lot of Southeast Asian immigrants.

68

00:10:05.940 --> 00:10:14.310

Participant: And that was and our numbers were really high we were the highest in the state of California, as far as our positivity rates.

69

00:10:15.120 --> 00:10:27.900

Participant: And when the opportunity came up for the program I know that the executive director for united way he went ahead and put us forward saying hey we'd be glad to go ahead and do this.

70

00:10:28.830 --> 00:10:29.280

Interviewer: uh huh.

71

00:10:31.980 --> 00:10:36.510

Interviewer: what are your thoughts about communication across the project or during the project in general.

72

00:10:38.760 --> 00:10:42.900

Participant: There was some issues, there were there were some issues.

73

00:10:45.150 --> 00:10:57.000

Participant: Sometimes it felt like we were playing telephone, because you had to go we'd have an issue we need to address with duke clinical but we had to go through CCPH

74

00:10:57.870 --> 00:11:09.480

Participant: So if they could ask the question, so it could go back to them, so it could come back to us and the things got lost in translation, as you know it normally does when there are so many passed through.

75

00:11:10.560 --> 00:11:18.780

Participant: And so that wasn't the best, but it wasn't terrible i've had worse, I worked for the government so i've had worse.

76

00:11:20.940 --> 00:11:36.750

Participant: We also there was a lack of communication in the beginning of the project we submitted what our plan was the plan was approved and then midway through, we had to pivot because we weren't getting the kits in.

77

00:11:38.160 --> 00:11:49.530

Participant: And we were on track as far as our distribution to our nonprofits and other agencies that were assisting with distribution.

78

00:11:50.250 --> 00:12:00.000

Participant: When we first signed on it was going to be 20,000 kits no problem that's you know easy peasy then it went to 100,000 kits still easy peasy.

79

00:12:00.750 --> 00:12:15.090

Participant: And then, a week later, with 200,000 kits not so easy peasy but we're, we have to pivot a little bit more made that plan and then all of a sudden, it was we're not going to send you those other hundred thousand kits until you do this and.

80

00:12:16.140 --> 00:12:20.430

Participant: And so it was kind of like you felt like a stepchild parts of the time.

81

00:12:21.120 --> 00:12:36.780

Participant: And we almost walked out in the middle of the project and said don't send the other kits were we're not going to do it because we had made the plan, I had gotten the insurance paid for insurances for our drive through events.

82

00:12:37.890 --> 00:12:47.130

Participant: had other agencies coming on our across the county to pick up test kits so they could set up, they pick them up like four or five days early.

83

00:12:47.400 --> 00:12:49.170

Participant: To set up for their function.

84

00:12:50.190 --> 00:12:59.970

Participant: And then, yet i'm being told they'll get those back well they haven't had their function, yet we picked him up that it was just it felt as though nobody was listening.

85

00:13:00.930 --> 00:13:12.300

Participant: And then pretty soon when we basically I said out that's Okay, I need some time off anyway, and at that point, the kits started coming in, when we started canceling events.

86

00:13:13.770 --> 00:13:21.840

Participant: And people started calling the numbers that we're not ours, so they were calling CCPH and.

87

00:13:21.900 --> 00:13:22.680

Duke clinical.

88

00:13:23.910 --> 00:13:35.100

Participant: Saying hey what's going on how come there's there's nobody here How come we can't get kits and then all of a sudden, we were able to get the rest of the kits and and we completed the job.

89

00:13:35.790 --> 00:13:46.410

Participant: and completed the task and handed out 200,000 and we mapped everything, so we know that it where they went, and that we covered the entire county.

90

00:13:47.220 --> 00:13:51.270

Interviewer: huh huh yeah, thank you for that um.

91

00:13:52.320 --> 00:13:57.120

Interviewer: It so I know that you said that you weren't privy to kind of the initial communication.

92

00:13:58.170 --> 00:14:07.470

Interviewer: And what you're describing is or if i'm understanding you correctly, that there were challenges in terms of there being a lot of kind of a long communication chain.

93

00:14:07.920 --> 00:14:11.430

Participant: So I wasn't brought in until day three.

94

00:14:12.150 --> 00:14:12.660

Interviewer: mm hmm.

95

00:14:12.960 --> 00:14:17.190

Participant: Okay, so we didn't have the contract, we didn't have product yet.

96

00:14:18.930 --> 00:14:35.010

Participant: We were ready on our side, it took me approximately maybe three or four days to start getting my side setup for us to find a warehouse that was temperature controlled where we could store the kits.

97

00:14:36.480 --> 00:14:37.680

Participant: We had everything set.

98

00:14:37.680 --> 00:14:49.830

Participant: up, it was just the communication afterwards wasn't the best and we knew and when i say we it's me and there's one other person from the united way.

99

00:14:51.420 --> 00:15:09.750

Participant: We knew we were on track, we knew that it was going to happen, the way we wanted, I had just come off of managing all the vaccination clinics in the county so we kind of already had a plan set and the united way is going to use my plan

100

00:15:10.800 --> 00:15:12.570

Participant: So does that make any more sense.

101

00:15:13.260 --> 00:15:20.280

Interviewer: yeah it does, I guess, I just want to clarify what was kind of the major like the method of communication was it.

102

00:15:21.300 --> 00:15:24.240

Interviewer: primarily via email or their meetings or.

103

00:15:24.450 --> 00:15:24.840

Interviewer: There were.

104

00:15:26.100 --> 00:15:36.990

Participant: There were meetings once a week and then there was emails and in the meetings they would have it at a very inconvenient time.

105

00:15:37.860 --> 00:15:44.040

Participant: For us, because they would want to do them at the same time that we had maybe a drive thru event going.

106

00:15:44.940 --> 00:16:02.070

Participant: And so it was you could take and turn on your phone and continue to get the kits out, you know what what was more important at the time, and so we had one of us, myself or rob would sit in on the meeting and the other person would be doing the work.

107

00:16:04.080 --> 00:16:04.770

Participant: So he ran.

108

00:16:04.920 --> 00:16:09.720

Participant: Their House I ran all the I took care of all the nonprofits in the Community.

109

00:16:12.270 --> 00:16:14.970

Interviewer: got it got it Thank you um.

110

00:16:16.500 --> 00:16:17.130

Interviewer: And then.

111

00:16:18.180 --> 00:16:24.270

Interviewer: Switching to the next question now what makes you or your organization decide to participate in the project.

112

00:16:25.860 --> 00:16:37.680

Participant: Because anything that comes into or is available for our Community we're going to try and jump in there if nobody else wants to take lead we're going to take the lead and.

113

00:16:38.190 --> 00:16:51.120

Participant: Really it's anything to help our neighbors our family members, we are a smaller Community the county we are about 184,000 for the entire county.

114

00:16:52.440 --> 00:16:57.300

Participant: But a lot of us, we were born and raised here, I guess, I didn't get that turned off

115

00:17:00.690 --> 00:17:07.620

Participant: We were born and raised here our grandparents are born and raised here and we consider each other, you know basically family.

116

00:17:08.760 --> 00:17:11.490

Participant: So our Community does take care of each other.

117

00:17:12.750 --> 00:17:15.210

Interviewer: yeah that's great um.

118

00:17:16.710 --> 00:17:19.380

Interviewer: And what were you asked to do for the project.

119

00:17:23.070 --> 00:17:24.930

Participant: I was asked do it.

120

00:17:25.470 --> 00:17:26.430

Participant: Just do it.

121

00:17:28.650 --> 00:17:39.090

Participant: i'm used to I retired out of the county and I was used to doing large projects and a lot of Community outreach.

122

00:17:39.870 --> 00:17:51.090

Participant: And so I was matter of fact, I was babysitting my grandchildren, one day, when I got the call my grandchildren they work their old one was 15 not able to drive herself to school, yet so.

123

00:17:52.170 --> 00:17:57.090

Participant: I got a call and asked to come talk to the united way and I said but i'm retired.

124

00:17:57.540 --> 00:18:12.510

Participant: well they said talk to us and I did, and it was immediate was like okay let's do this and that day I threw out a map, this is what we would do this is how we would do it.

125

00:18:13.140 --> 00:18:24.540

Participant: And they agree to it, we went ahead and sent that in is our model and we basically stuck to the same model, the entire way through.

126

00:18:26.280 --> 00:18:26.850

Interviewer: mm hmm.

127

00:18:27.900 --> 00:18:39.120

Interviewer: And what you just described in terms of your model and your plan how close were those activities to your regular tasks, or what united way typically does.

128

00:18:41.760 --> 00:18:44.430

Participant: Well you're talking about a.

129

00:18:45.510 --> 00:18:57.330

Participant: Very non typical couple of years, and so the united way they normally do a lot of funding of projects.

130

00:18:58.770 --> 00:19:05.820

Participant: But this one we took on we were you know boots on the ground, united way was out there, doing the work.

131

00:19:07.110 --> 00:19:09.480

Participant: For that reason, well.

132

00:19:10.710 --> 00:19:17.100

Participant: united way and, as you i'm sure it's the same on the east coast getting people hired is rough.

133

00:19:18.450 --> 00:19:30.720

Participant: getting them tired and trained is tough, but here we only had six weeks to do it in, and so, for that reason we chose the option A option of using Community partners who already had.

134

00:19:31.560 --> 00:19:48.780

Participant: Individuals that do Community outreach and then I took another piece of Community outreach and it was very meeting people shaking hands here's your stuff explaining things so we were very.

135

00:19:51.510 --> 00:19:54.480

Participant: We were basically boots on the ground, everybody knew who we were.

136

00:19:55.650 --> 00:19:57.600

Participant: But normally it's not that.

137

00:19:57.810 --> 00:20:00.720

Participant: Normally it depends on the project coming through.

138

00:20:02.790 --> 00:20:21.030

Participant: The united way right now they've asked me to come in and work on updating some databases, they run the two ones information system and things such as that except for right now they're all in Las Vegas at a conference and i'm here in merced at the city.

139

00:20:23.250 --> 00:20:23.700

Participant: Having.

140

00:20:24.360 --> 00:20:24.900

yeah.

141

00:20:27.390 --> 00:20:32.100

Interviewer: yeah that's tough, it is especially if you haven't gotten to travel anywhere, for the last couple years.

142

00:20:35.550 --> 00:20:44.970

Interviewer: um some what resources did your organization required to be able to complete these the tasks that you had to do as part of the project.

143

00:20:46.680 --> 00:20:47.640

Participant: Okay, so.

144

00:20:49.770 --> 00:20:54.600

Participant: We had to take and tell you know if i'm if i'm off track sent me back on.

145

00:20:55.050 --> 00:20:55.590

Participant: So we have.

146

00:20:55.620 --> 00:20:59.430

Participant: To take and acquire a storage facility.

147

00:21:00.780 --> 00:21:06.780

Participant: And then we needed to take and find people that could operate the equipment, since it's huge pallets.

148

00:21:06.960 --> 00:21:23.820

Participant: were coming in, because that's kind of not what we do well, and so we did have the school system allowed us to use one of their buildings, as well as their employees to take an assistant unloading the trucks that were coming in.

149

00:21:25.200 --> 00:21:29.310

Participant: We recruited a few part time people who do.

150

00:21:30.720 --> 00:21:33.450

Participant: Basically piece work for the united way.

151

00:21:34.470 --> 00:21:54.810

Participant: And so I pulled them in in some of them could only work, maybe three hours a week, but still three hours a week, to help us arrange things one of them was doing all of our pictures and posting things on you know social media and such another one.

152

00:21:56.010 --> 00:21:57.030

Participant: Let me think, then, what.

153

00:21:58.410 --> 00:22:02.340

Participant: We had to take in purchase the insurances because of all the drive thru's

154

00:22:06.540 --> 00:22:19.860

Participant: i'm trying to think if there was anything else we did offer incentives to all of the nonprofit's who were assisting us and those were paid out dependent upon the number of kits that they distributed, but none of them knew this ahead of time.

155

00:22:21.420 --> 00:22:33.360

Participant: They were made aware of it, after the program was over, because I felt as though I didn't want them to hoard kits knowing they were getting paid per kit.

156

00:22:33.930 --> 00:22:34.530

Participant: So it was.

157

00:22:34.800 --> 00:22:38.130

Participant: If I told them I said Oh, and now I have a blessing for you.

158

00:22:39.630 --> 00:22:42.960

Participant: Also, are utilized volunteers I run a.

159

00:22:44.100 --> 00:23:06.030

Participant: During the code vaccine clinics, I had recruited in close to 600 volunteers to assist me with the clinics, and so I called back on those that lived in the towns or cities that we were going to be having events that I thought had a hold of them and have them come back into assist.

160

00:23:07.770 --> 00:23:18.360

Participant: There was little basic things resources, I mean and we needed to have like safety vests and things such as that some tents to go over there, where the kits were going to be distributed.

161

00:23:21.690 --> 00:23:35.910

Participant: Other than that, that really sounds like about it, we did have some issues with the bags not coming in, so we had to take and figure something else out for that we did.

162

00:23:38.820 --> 00:23:40.290

Participant: But basically, that was it.

163

00:23:41.610 --> 00:23:41.820

Participant: I mean.

164

00:23:42.030 --> 00:23:43.080

Participant: You had six weeks.

165

00:23:43.110 --> 00:23:49.260

Participant: You have six weeks to get them all out so it was kind of what have we already got in place and let's go ahead and run.

166

00:23:49.950 --> 00:24:00.870

Interviewer: Right right that makes a lot of sense and what resources, would you require specifically from the say yes covid test team to complete the tasks.

167

00:24:01.440 --> 00:24:13.320

Participant: So what they did was it brought on, and this is where we different from the other programs, so they sent us a led light light truck.

168

00:24:14.340 --> 00:24:22.500

Participant: And it was like a box truck That was all lit up and said you and me covid free on the sides and telephone numbers and things.

169

00:24:23.160 --> 00:24:33.180

Participant: So I scheduled because it's close to all of our nighttime Christmas parades I went ahead and schedule that truck to be in.

170

00:24:33.660 --> 00:24:48.900

Participant: In the parade which we got some really cool pictures, especially when the you and me covid free truck is right in front of Santa Clause so it everybody was taking pictures and posting out those pictures online.

171

00:24:50.160 --> 00:25:03.360

Participant: And there, it was in every picture that was being posted so that was really cool and then we were provided with a distribution truck so it was a food truck with the kits in it.

172

00:25:06.720 --> 00:25:07.470

Participant: That one.

173

00:25:09.060 --> 00:25:20.280

Participant: It was really it was cool to be able to have that truck because was large enough to take out a lot of kits we did not distribute out of the truck.

174

00:25:20.910 --> 00:25:29.970

Participant: The truck we used it more for advertising, this is where we are and then we would set up, I had volunteers.

175

00:25:30.390 --> 00:25:42.210

Participant: That were runners basically with the kits finding out how many people needed and taking gather information and another person telling them, you know this is how use the test as we're getting them out of the parking lots.

176

00:25:43.680 --> 00:25:46.920

Participant: But that was really a cool thing they supplied the flags.

177

00:25:48.030 --> 00:26:04.770

Participant: Flags got stolen out of the truck the very first day the truck was there here in merced, it was funny because they stole the flags, but they didn't take any test kits with them, which they could have gotten some money for so it was kind of all right.

178

00:26:07.980 --> 00:26:30.270

Participant: Also, provided us with a lot of advertising a ton of advertising, so it was on social media, the radio stations TV stations, the little kiosks at the gas stations, so it had really hit everywhere we had people that were driving from bakersfield, which is about four hours away.

179

00:26:30.900 --> 00:26:34.890

Participant: To come up here to pretend they live in merced so we could give them test kits.

180

00:26:36.390 --> 00:26:39.330

Participant: So, because the word got so far.

181

00:26:40.500 --> 00:26:43.470

Participant: And so, those were the things you know they would.

182

00:26:44.580 --> 00:26:47.070

Participant: As well as the kits and you know.

183

00:26:48.120 --> 00:26:51.000

Participant: The bags and that kind of stuff is also what was supplied.

184

00:26:51.810 --> 00:26:53.040

Interviewer: Okay, great.

185

00:26:54.210 --> 00:27:00.900

Interviewer: um and during the course of the project did you have any questions for the team during your collaboration with them.

186

00:27:03.300 --> 00:27:06.120

Participant: not really questions.

187

00:27:07.680 --> 00:27:23.760

Participant: It was more clarifications, because you had different people, and it was exactly what is that you want, what is your vision and that's why I always ask what is it you're seeing what's The bottom line, what do you want to see in the end.

188

00:27:25.140 --> 00:27:39.540

Participant: We had issues with the computer system, the tracking system and the way they were having issues in the beginning and basically it doesn't work for.

189

00:27:41.250 --> 00:27:48.990

Participant: It wasn't working for us because you want to see on a daily basis to track where things are going, where they are.

190

00:27:50.700 --> 00:28:01.470

Participant: And where are we failing if there's a hole in the system if people are not tracking them why and who is it that isn't tracking because I know who has the kits.

191

00:28:01.830 --> 00:28:11.400

Participant: I know what events they have, and I know what zipcodes I should be having test kits being registered in if that isn't happening, let me nip it now.

192

00:28:12.000 --> 00:28:30.900

Participant: I not a week and a half later and try and figure out a mess, let me do it now, and so they did go ahead, approximately three weeks in or so open up the portal to where I was able to get closer to real time not real time but closer.

193

00:28:32.220 --> 00:28:40.380

Participant: Which assisted me with being able to report back without having to say i'll have to check into it get back to you later, which I hate that.

194

00:28:41.340 --> 00:28:42.210

Interviewer: Right right.

195

00:28:42.270 --> 00:28:46.500

Participant: i'd rather tell you what I know, but I need those tools to be able to give you that information.

196

00:28:47.610 --> 00:28:58.800

Interviewer: yeah that makes sense, and so you just mentioned one example of this, but i'm wonder if you could talk about how responsive you felt like the project team was to the questions that you raised.

197

00:28:59.880 --> 00:29:01.530

Interviewer: or I guess points of clarification.

198

00:29:02.670 --> 00:29:05.520

Participant: You know I I felt as though we I had.

199

00:29:06.630 --> 00:29:08.130

Participant: It was really good communication.

200

00:29:10.650 --> 00:29:14.730

Participant: it's because the other pieces it's brand new relationships.

201

00:29:15.810 --> 00:29:34.740

Participant: And anytime that you're dealing with brand new relationships you don't want to i'm one I see the finish line and i'm going to get there and i'm going to succeed that's my personality, but sometimes I run over people in the meantime, and so I try to stay in my lane and.

202

00:29:36.420 --> 00:29:44.490

Participant: People who know me well know, had my personality which Ellen has no she knows my personality so but.

203

00:29:45.600 --> 00:29:53.040

Participant: yeah it took a while to get that relationship to where you didn't want to hurt somebody's feelings, you know what i'm saying it's kind of.

204

00:29:54.270 --> 00:30:04.050

Participant: Your kind of try to be kind on everything you say and don't just say what you need, but they were very, very responsive.

205

00:30:05.490 --> 00:30:12.540

Participant: Normally, if somebody was at the office somebody else was picking up and sending me an email answering those questions for me.

206

00:30:13.500 --> 00:30:25.650

Interviewer: Okay, and if this is different, it might it might not be different, but how responsive, where the project TEAM members to responding to any needs that you raised.

207

00:30:26.550 --> 00:30:29.520

Participant: This is the duke clinical team correct.

208

00:30:30.450 --> 00:30:32.760

Interviewer: yeah they say yes covid test team.

209

00:30:33.450 --> 00:30:40.620

Participant: Well, because we had two different groups I as a CCPH people, and then I have duke clinical.

210

00:30:41.790 --> 00:30:52.260

Participant: Duke clinical was really good to get back to me and i'm going to say immediately, even with the time differences, they got back to me what I would say immediately.

211

00:30:53.460 --> 00:31:09.600

Participant: Also, the the person that was running the advertising would get back to me really quick the people that the contractor for the trucks got back to me really quick sometimes CCPH, it might take them a couple of days.

212

00:31:10.830 --> 00:31:17.580

Participant: But they were not as responsive, but they would eventually get back with you.

213

00:31:19.620 --> 00:31:21.090

Interviewer: Okay, thank you.

214

00:31:22.620 --> 00:31:29.250

Interviewer: And do you think that the project addressed a county need related to cover 19 health concerns.

215

00:31:30.750 --> 00:31:31.980

Participant: a county what?

216

00:31:32.550 --> 00:31:38.700

Interviewer: addressed a county level needs so essentially did the project address your counties needs around covid 19.

217

00:31:39.000 --> 00:31:40.500

Participant: Oh yeah, the first thing I did.

218

00:31:41.820 --> 00:31:45.750

Participant: In the first week was went to the county board of supervisors meeting.

219

00:31:46.770 --> 00:32:03.330

Participant: And Roche, the podium and said, this is what we're doing you this is who we are, this is what we're doing if you have any questions, let me know blah blah blah blah got my three minutes out there, and so we had the county on board with us and.

220

00:32:04.410 --> 00:32:08.190

Participant: So I mean the fire department showed up they run board with us.

221

00:32:09.240 --> 00:32:19.200

Participant: The health department, they didn't do anything they had one meeting with us, but they don't like to do much work at the health department here, and so they just kind of melted away.

222

00:32:21.120 --> 00:32:29.280

Participant: But as far as the county we were able to saturate, as I said earlier, the entire county we tracked everything by the zip code.

223

00:32:29.970 --> 00:32:39.540

Participant: And I mapped out how many individuals we had any to zip code, so that could see if I was missing like little bitty town of Stevenson.

224

00:32:40.290 --> 00:32:49.650

Participant: I know that I only have 200 people there, but i've only put out 10 kits, so I would call one of my nonprofits that services at areas a hey do you mind going to.

225

00:32:50.130 --> 00:32:55.770

Participant: You know the post offices, set up a table in front of the post office and we'll go ahead and get them the word out.

226

00:32:56.280 --> 00:33:04.770

Participant: Through the fire departments section we're going to be there and that nonprofits would take off and take that territory, and if we saw that somebody was.

227

00:33:05.280 --> 00:33:18.570

Participant: An area was getting too many and I have a nonprofit says, I want to go do something, and when I say ya know i'm already at a 75% there I need to pull back and can I send you to Selling.

228

00:33:19.650 --> 00:33:20.490

Participant: And that's what we did.

229

00:33:21.570 --> 00:33:29.340

Interviewer: yeah that's great and and what would you say the needs are the major means in your county were related to covid 19

230

00:33:32.760 --> 00:33:33.570

Participant: Education.

231

00:33:34.980 --> 00:33:36.330

Participant: Definitely, the test kits.

232

00:33:40.470 --> 00:33:42.210

Participant: At that it by the time.

233

00:33:43.860 --> 00:33:46.680

Participant: November, December rolled around we did the project.

234

00:33:49.470 --> 00:34:00.030

Participant: There were the vaccines were available here, we had a rough time in the beginning, getting them and so really I think was the education piece, as well as the kits.

235

00:34:00.630 --> 00:34:10.260

Participant: And even people I had one man, he told me the kits were horse manure and then he came back to me because I can imagine at a Bingo at the elks lodge.

236

00:34:10.890 --> 00:34:26.730

Participant: And then he comes back this you're almost out, and I said yes, I am and he says, can I get some for my family I said but I thought their horse manure and he says, I need some well his son nearly passed away of covid.

237

00:34:27.240 --> 00:34:44.130

Participant: And this man is in his 80s that's giving me hard time and i've known him for many, many years, but he almost lost his son, but he was one of those no no we're not getting vaccines, I know for a fact he was vaccinated because it came to one of my clinics I pretended I didn't see him.

238

00:34:45.720 --> 00:34:55.950

Participant: But we have a lot of those kind of folks that they were too embarrassed to say things have changed and now they've started believing what they were hearing.

239

00:34:58.140 --> 00:34:59.730

Interviewer: yeah that makes sense.

240

00:35:00.870 --> 00:35:08.160

Interviewer: And this is a related question, but what do you think are some of the biggest barriers to covid 19 testing in your county.

241

00:35:11.370 --> 00:35:18.960

Participant: The biggest barrier is the fact that we have a lot of farm workers and meat packing.

242

00:35:20.040 --> 00:35:23.670

Participant: produce packers and a lot of our.

243

00:35:24.870 --> 00:35:25.920

Participant: Our workers.

244

00:35:27.300 --> 00:35:36.480

Participant: The first they can't take off work they work, and if they test positive for covid they wouldn't be able to go to work so.

245

00:35:37.500 --> 00:35:54.780

Participant: If I don't know I have covid but if there's if it's not in black and white, then I don't have it and i'm going to continue to go to work and not realizing the harm that they could have taken home to their families also they were afraid that they would be replaced.

246

00:35:55.950 --> 00:36:06.150

Participant: Their field jobs or a bit the week God to foster farms out here and they were a fearful that they would lose their jobs.

247

00:36:06.690 --> 00:36:15.570

Participant: So that was the biggest thing once we told them, you know these tasks you're going to test at home and nobody else is going to see the results.

248

00:36:16.170 --> 00:36:26.070

Participant: and gave them some tips on how to stay healthy and you know we have a lot of them that we're going to go back to Mexico over the Christmas break.

249

00:36:26.400 --> 00:36:37.290

Participant: And so we went ahead and printed out some protocols and such for them, but the fact that we were giving them the free test kits it allowed us to open up that conversation a little bit.

250

00:36:38.010 --> 00:36:38.520

Interviewer: mm hmm.

251

00:36:39.840 --> 00:36:50.010

Interviewer: yeah and I guess building on what you're what you're just speaking about now do you think that this as covid test project responded well to the barriers to covid testing in your area.

252

00:36:50.970 --> 00:37:03.810

Participant: I think so, and the reason, again we go back to culture and the nonprofit's which we handpick I hand it over 100 nonprofits that I know that work.

253

00:37:04.470 --> 00:37:17.040

Participant: hands on with different communities within my county, and so I picked out the what I refer to this, the heroes, so they heroes are not your elected officials.

254

00:37:17.970 --> 00:37:30.810

Participant: It is the people who are looked up to within their culture and they're going to be the guy that is out there working pulling the weeds by the by the irrigation ditch.

255

00:37:31.680 --> 00:37:47.100

Participant: He may be that communities hero and that's why you get A hold of them first and that's what we did we worked through the heroes within the Community and that, because that person is like me.

256

00:37:48.540 --> 00:37:59.490

Participant: You know, and even when I was doing the vaccination clinics, one day, I had 1000 were supposed to be coming through, and it was 300 showed up in the morning.

257

00:38:00.420 --> 00:38:09.960

Participant: Because well and then, once they went back to work that evening I was slammed, but it was because the heroes went back to work.

258

00:38:10.500 --> 00:38:19.650

Participant: And they were still alive and so hey he did it so now i'm going to do it, and the same thing with the elders here.

259

00:38:20.340 --> 00:38:31.350

Participant: If they went ahead and had their family vaccinated or if they were handing out the test kits and saying yes we use them, then that brought in the other people that to start using them as well.

260

00:38:32.730 --> 00:38:34.530

Interviewer: yeah that that makes a lot of sense.

261

00:38:37.200 --> 00:38:39.660

Interviewer: Anything else to add before we switch gears a little bit.

262

00:38:40.350 --> 00:38:40.620

nope.

263

00:38:41.880 --> 00:38:42.210

Participant: I.

264

00:38:42.690 --> 00:38:44.340

Participant: asked them what i'm watching the clock.

265

00:38:44.940 --> 00:38:45.630

Interviewer: you're doing great.

266

00:38:47.340 --> 00:38:56.520

Interviewer: So, at the end of the project when everything was all wrapped up how close to your typical activities match to what you were asked to do for the say yes covid test project.

267

00:38:59.310 --> 00:39:01.740

Participant: I believe we we hit the nail on the head.

268

00:39:02.940 --> 00:39:07.860

Participant: I mean the the scope of work was very loose and.

269

00:39:08.880 --> 00:39:18.720

Participant: It basically it's get those get 200,000 test kits out before this date and make sure you saturate the entire county and that's what we did.

270

00:39:19.380 --> 00:39:38.940

Participant: And then we had the backup documentation because of the records, we were keeping that we're not required by us that we were able to go back and show what we've done and the reason that we did the additional paperwork or reporting was for our next projects that are coming up.

271

00:39:40.140 --> 00:39:45.150

Participant: So we don't will already be ramped up we just gotta jump in and take off.

272

00:39:45.750 --> 00:39:53.700

Interviewer: mm hmm makes a lot of sense and did you find the tasks that you were asked to do as part of the say yes covid test to be difficult.

273

00:39:55.290 --> 00:39:55.650

Participant: No.

274

00:39:56.550 --> 00:39:57.090

Participant: Not at all.

275

00:39:59.970 --> 00:40:04.680

Interviewer: Great and could you do you want to say a little bit more about that or would you be willing to.

276

00:40:06.390 --> 00:40:07.920

Participant: Just to stay on track i'm fine.

277

00:40:10.080 --> 00:40:28.740

Participant: No, it wasn't difficult at all, because you were with when you're giving back to your community and you're helping people understand, I mean it was scary people were afraid and i'm going to go back to my vaccination clinics best it means it kind of gives the mind my mindset.

278

00:40:30.000 --> 00:40:39.270

Participant: So when they were first starting to give out the vaccinations merced county we weren't getting a lot we blamed our share for fighting with our governor and that's why, but you know.

279

00:40:39.870 --> 00:40:49.860

Participant: We just like to give him a hard time, but what would happen is, we would have enough vaccinations the vaccine for 500 people.

280

00:40:50.850 --> 00:41:06.750

Participant: And they give be giving out the vaccinations and I have 8, 10 , 12 people in their 80s, with their walkers with their spouses standing there, hoping that there's something left over because they couldn't get an appointment.

281

00:41:07.770 --> 00:41:15.750

Participant: The appointments were cut off, and so a matter of fact, I kept putting off having the vaccination done because.

282

00:41:17.400 --> 00:41:31.680

Participant: i'm healthy and I can take that you know I can take precautions they're afraid, these people are in tears afraid of this virus and so, seeing that and knowing that fear that they had.

283

00:41:33.330 --> 00:41:54.840

Participant: This program it was a blessing to have this program to come to our Community, and all of us felt it was a blessing, and so you can't say to receive it was difficult because we were able to give back and to a community that are scared to death, their neighbors were dying.

284

00:41:56.220 --> 00:41:58.710

Interviewer: huh huh huh yeah.

285

00:42:02.730 --> 00:42:07.800

Interviewer: So what would you say, are some of the reasons individuals in your Community picked up tests

286

00:42:09.210 --> 00:42:11.130

Participant: Where they do pick up test kits.

287

00:42:11.400 --> 00:42:13.560

Interviewer: yeah why they ordered them yeah or pick them up.

288

00:42:14.160 --> 00:42:14.460

Okay.

289

00:42:15.840 --> 00:42:21.420

Participant: So I had one gentleman came up he says we're going to be having a party all of my grandkids are coming.

290

00:42:22.290 --> 00:42:33.270

Participant: up, we want to make sure everybody is safe, you know and so he went ahead and he took a little box to each of his kids and said you're going to test before you come over.

291

00:42:33.630 --> 00:42:47.580

Participant: And it was because his wife had been sick, we found out afterwards and he wanted to make sure everybody coming household this healthy our Community was well aware that our rates for the highest in this in the state of California.

292

00:42:49.320 --> 00:42:53.550

Participant: That that wasn't lost on us and so people.

293

00:42:54.900 --> 00:42:58.080

Participant: I mean we we do a drive through and run out.

294

00:42:59.160 --> 00:43:10.380

Participant: And we have to tell people you know this is going to be the next one, and I tell them show up 30 minutes early I promise all take care of you show up early and you know because they've already been there once.

295

00:43:14.010 --> 00:43:16.710

Participant: It was just a scary time.

296

00:43:17.820 --> 00:43:37.530

Participant: It was just scary we had like I mentioned earlier, the one gentleman who was an Anti vaccinator and everything else, and yet, can I please have a couple of kits for my family, you know so even people that didn't believe in taking getting the vaccination.

297

00:43:38.910 --> 00:43:48.270

Participant: They were the pit in we're taking the test kits and we know that they use them and there's a mere fact that one of the churches there's a pharmacist there that he swore.

298

00:43:49.200 --> 00:43:59.160

Participant: never, never to the vaccination, everything is everything is bad, and so the people in his church he's the hero in their church so they're looking up to him.

299

00:44:00.360 --> 00:44:14.040

Participant: And I show up one day with what five cases of test kits and I said okay I got the girls out of one of the Sunday school classes, the teens and I said you guys are going to help me.

300

00:44:14.790 --> 00:44:24.570

Participant: And I explained to them what it was, and we talked about it, and then, as soon as the parishioners started coming in, for their their holidays lunch.

301

00:44:25.710 --> 00:44:35.880

Participant: I tell you what even the guy that didn't agree with the test kits I mean the vaccinations or the kits he took some with him, he ended up having covid.

302

00:44:36.450 --> 00:44:45.210

Participant: Then that entire church came back and thanked me for distributing the test kits because what happened was they would test.

303

00:44:45.990 --> 00:44:59.430

Participant: If they've been anywhere prior to going to church, so if they stayed home for three weeks, they didn't test, but if they were going to go to church they tested before they went to church if they have even been let's say at walmart.

304

00:45:01.080 --> 00:45:11.790

Participant: Or if they weren't feeling well, which you know that was really kind of cool to have this group that totally didn't agree with it and I crashed the doors anyway, but.

305

00:45:13.080 --> 00:45:21.210

Participant: You know you pace when your father goes to that church and go dad I need a favor but so that was really.

306

00:45:22.710 --> 00:45:26.820

Participant: Why, I think a lot of them, they knew they knew.

307

00:45:28.170 --> 00:45:41.790

Participant: But there's so much propaganda on TV right now that you know you're fighting against it, but I was pleased i'm pleased to say that my antibiotics are picking up the kits and they have used them in have told me they've used him.

308

00:45:42.270 --> 00:45:51.630

Interviewer: huh yeah yeah that's great and and in your county to people have the option to order testing kits online using an online ordering option.

309

00:45:52.230 --> 00:45:53.370

Participant: That has just started.

310

00:45:53.880 --> 00:46:01.080

Participant: Okay, it was after these so you were able to well it's the same ones that the entire country was able to get.

311

00:46:01.380 --> 00:46:14.490

Participant: From the from the Federal Government, so they were able to get those and I know for myself, I just received a notice from express scripts, which is the mail order prescriptions that.

312

00:46:15.780 --> 00:46:20.400

Participant: Each person can order, a certain number of kits and get them in mail order now.

313

00:46:21.420 --> 00:46:23.610

Interviewer: But that wasn't an option specifically under this.

314

00:46:23.910 --> 00:46:28.740

Participant: Product absolutely no there were at there was just no kits anywhere.

315

00:46:30.270 --> 00:46:36.750

Participant: And that's back when the President was saying i'm getting kits i'm getting kits and that didn't happen to like February.

316

00:46:37.140 --> 00:46:48.960

Participant: Right in February yeah we were doing test kits started November 17 and ended December November November 17 through December 31.

317

00:46:49.830 --> 00:46:50.340

Interviewer: mm hmm.

318

00:46:50.580 --> 00:46:51.180

Participant: was our time.

319

00:46:52.170 --> 00:46:52.560

Okay.

320

00:46:55.020 --> 00:47:00.780

Interviewer: And do you have any recommendations for this as covid test project to improve their approach.

321

00:47:05.550 --> 00:47:09.720

Participant: No, it really i've been I think that anything that.

322

00:47:10.020 --> 00:47:10.380

Participant: The bear.

323

00:47:10.710 --> 00:47:26.460

Participant: That we ran into we were able to talk them out and explain them during our weekly meetings either have the meetings weekly, then they went bi weekly, I think, but we were able to have the discussion and say this isn't working, this is working and move right through.

324

00:47:28.590 --> 00:47:31.290

Participant: The end really things for smooth.

325

00:47:32.400 --> 00:47:39.450

Interviewer: And so you feel like i'm in those conversations that the recommendations that you all were making were incorporated.

326

00:47:40.320 --> 00:47:41.940

Participant: We feel as though we were heard.

327

00:47:42.840 --> 00:47:46.170

Interviewer: Okay, great um.

328

00:47:47.310 --> 00:47:56.250

Interviewer: And lastly, just a couple kind of wrap up questions um What would you like to see happen with your community and their health, moving forward.

329

00:47:57.180 --> 00:48:03.480

Participant: Well, we are going to be, I believe it was just the link was just went live for you and me healthy.

330

00:48:04.590 --> 00:48:19.290

Participant: So I am right now waiting for the social media postings and such to be sent to me, and we will start going ahead and getting that information out to the Community so that's our next Community outreach project.

331

00:48:19.800 --> 00:48:21.270

Interviewer: And what's the focus of that project.

332

00:48:22.920 --> 00:48:25.830

Participant: It I don't know a whole lot about it.

333

00:48:26.850 --> 00:48:39.240

Participant: But it is also through CCPH and Duke clinical and it is the next step after the unicode free We already have the database this APP.

334

00:48:40.290 --> 00:49:04.230

Participant: They know who a lot of our people are that took the test and did the surveys, and this is basically it's my understanding, because I haven't gone into the site, yet it is to provide information to the public, so it should be healthier living and what you what you need to do to live healthy.

335

00:49:05.310 --> 00:49:11.010

Participant: In let's say that I don't know because I haven't seen the platform yet, but.

336

00:49:12.120 --> 00:49:18.810

Participant: The monkey pox if somebody had questions regarding that I believe that that would be something somebody is having issues with.

337

00:49:21.300 --> 00:49:31.560

Participant: A going to say they diabetes, or something but they're supposed to be able to use that platform for medical.

338

00:49:33.870 --> 00:49:34.560

Participant: information.

339

00:49:36.690 --> 00:49:51.540

Participant: So i'm curious because it's just now opening up and i'm sure it's going to morph into other things, but yeah it was supposed to have gone live on the 26th and i'm meeting with them on Friday, I think.

340

00:49:52.680 --> 00:49:53.850

Participant: So we can actually see it.

341

00:49:54.810 --> 00:49:55.590

Interviewer: yeah very cool.

342

00:49:56.760 --> 00:50:03.810

Interviewer: And then, lastly, do you have any other questions or comments anything that we want to share that we didn't talk about today.

343

00:50:04.740 --> 00:50:08.880

Participant: No, I think we were they gave you the copy of our report correct.

344

00:50:10.020 --> 00:50:10.410

Interviewer: yeah.

345

00:50:10.860 --> 00:50:11.850

Interviewer: The program folks.

346

00:50:12.840 --> 00:50:16.410

Participant: Okay, so a lot of the stuff will be in there.

347

00:50:18.450 --> 00:50:24.150

Participant: No like I said we've debriefed with several people and.

348

00:50:25.620 --> 00:50:31.380

Participant: Now I i'm just thrilled with the project i'm thrilled with the way the project turned out.

349

00:50:32.760 --> 00:50:37.200

Participant: We feel in merced that the project was very successful here.

350

00:50:38.310 --> 00:50:40.470

Participant: And again, our Community.

351

00:50:41.550 --> 00:50:55.470

Participant: And i'm sure it has come through on a lot of the survey say did our Community felt very blessed to be a part of this project yeah they were very thankful, and I know that they express that in their comments.

352

00:50:57.180 --> 00:51:07.680

Interviewer: Good good to hear so Those are all of the questions that we have today, I want to thank you for your time and your help, and I think Christina is gonna close out with.

353

00:51:08.790 --> 00:51:25.260

Participant: Okay, well, if you get up needing clarification I think just give me a holler send me an email and i'll answer, because I know I plan to go off here, but i'm a story answer because there's you know 10 explains where what i'm looking at.

354

00:51:27.210 --> 00:51:34.110

Note taker: your answers are great so i'm just going to stop the recording now and then i'm just going to get your information for the.

355

00:51:34.650 --> 00:51:35.010

Participant: wise.

356

00:51:35.190 --> 00:51:36.690

Participant: grass on the wall behind you.

357

00:51:37.950 --> 00:51:39.270

Note taker: Oh, I thought it looked Q.

358

00:51:40.470 --> 00:51:41.700

Participant: All I see is the bottom.
