# Supplementary material for: Exploring barriers and facilitators of implementing an at-home SARS-CoV-2 antigen self-testing intervention: The Rapid Acceleration of Diagnostics—Underserved Populations (RADx-UP) initiatives
Source: PLoS One. 2023 Nov 16;18(11):e0294458. doi: 10.1371/journal.pone.0294458 (PMC10653400; doi:10.1371/journal.pone.0294458)
Supplement: S1 Dataset — (ZIP) [file pone.0294458.s002.zip › P'sID(7)-Notes (8.18.22).docx]

1. Pediatric Healthcare associates – serves underserved population in their community. 92% is on medicade. Serves minority patients – educating patients on covid and covid testing
2. Administrative manager
3. In an area that’s a food desert and in the middle of an underserved population area. Can get the kits out easier because there on a bus line and accessible to people
4. Initially doing okay, health department hindered them being able to serve the minority and underserved populations. Get the test kits out to everyone – missing the mark because the health department wasn’t targeting minorities due to where they were setting up
5. Daphne contacted her – Chattanooga potentially the location and asked if she could sit in on the advisory board. She signed them up as a community partner
6. Good communication Angela was continuously following up with them about he project
7. Sought the need to help their patients and make them realize that testing is safe and they are not getting covid by taking the test
8. Was asked to create a list of potential community partners to participate in the project
9. Health department had a different list than them
10. Creating the list wasn’t hard because she served on other non-profits and already had the list from other projects
    1. Matter of events being set up to distribute the test kits
11. Meet with Angela weekly, communication was strong. Not many questions and when she did have them, she was very responsive. Timely, easy to work with, etc.
12. Yes, but health department didn’t stick with guidelines. SYCT was helpful in the community
13. The school system didn’t have good measures in place for COVID testing, they were sending kids back to the doctor to keep getting tested. Worried about running out of test because of re-testing people frequently
14. The test kits would have been helpful to do at home and take a picture of the result then call the doctor and ask what to do.
15. Myth that covid testing gives you covid – especially in black community. Spending a lot of time educating people on covid and the testing.
16. Closely aligned with SYCT, didn’t align closely with health department. Health department didn’t target the underserved populations. Holding kits for their employees and specific people that weren’t in need or had access.
17. Tasks were not difficult because she has experience with healthcare thesis’ and running a study. Her background helped her complete these tasks
18. Some people initially wanted to make sure they could test themselves (40-60yrs old) and teachers wanted to make sure they knew how to test themselves and had the kits to test
19. 65+ years preferred to pick up, <65 years preferred to order online
20. All programs and departments follow the same protocols. Health department didn’t follow the targeted audience
21. Other physicians and health departments focus on minorities and underserved population
22. Enjoyed program and wants it to be brought back. People there needs continual education, and the growth of the city has made underserved populations live together with multi-generations

Debriefing

- Concise, had a clear understanding of what was being asked of her
- She stayed on topic and had valuable information
- She was focused and really would like the program to be brought back to her area
