# Supplementary material for: Exploring barriers and facilitators of implementing an at-home SARS-CoV-2 antigen self-testing intervention: The Rapid Acceleration of Diagnostics—Underserved Populations (RADx-UP) initiatives
Source: PLoS One. 2023 Nov 16;18(11):e0294458. doi: 10.1371/journal.pone.0294458 (PMC10653400; doi:10.1371/journal.pone.0294458)
Supplement: S1 Dataset — (ZIP) [file pone.0294458.s002.zip › P'sID(7)-Transcript (8.18.22) [Christina].docx]

WEBVTT

00:00:06.270 --> 00:00:11.040

Interviewer: If it's okay with you to begin unless you have any other question.

00:00:15.330 --> 00:00:16.199

Interviewer: No question.

00:00:18.539 --> 00:00:33.270

Interviewer: we'll start with some questions about your role at the participating organization, so the first question is, what is the primary mission of your organization that you are part of our part of doing this as a test project.

00:00:34.440 --> 00:00:39.180

Interview Participant 7: A pediatric healthcare associate, my primary role.

00:00:40.740 --> 00:00:52.230

Interview Participant 7: For the say yes covid test was to distribute you know kits to the underserved population our office serves underserved populations as a pediatric medical practice.

00:00:54.060 --> 00:01:09.120

Interview Participant 7: In our Community, we serve is 92% of our patient population is 10 care mean they're on medicaid, so we are serving a large mile and underserved.

00:01:09.120 --> 00:01:10.200

Interview Participant 7: populations.

00:01:10.380 --> 00:01:11.190

Interview Participant 7: We are the only.

00:01:11.250 --> 00:01:29.190

Interview Participant 7: Three practices accepts all of the tinker group, so we are serving a large majority minority patients in our office, so we spent a large top part of our time during say yes go with educating patients, not only on covid testing.

00:01:32.520 --> 00:01:33.060

Interviewer: Thank you.

11

00:01:34.080 --> 00:01:39.060

Interviewer: What is your position in the organization.

00:01:39.540 --> 00:01:55.950

Interview Participant 7: I was administrative operational manager I ran the pediatric practice but also oversaw our building because we own the large medical building that we run in space to other physicians or medical professionals they're winning those faces.

00:01:58.140 --> 00:01:58.740

Interviewer: Thank you.

00:02:00.060 --> 00:02:08.490

Interviewer: next question is about your organization and specifically the say yes covid test project which you shared a bit already about.

00:02:09.780 --> 00:02:15.330

Interviewer: In your opinion, what made you or your organization, a good partner from the same school good test project.

00:02:17.490 --> 00:02:25.320

Interview Participant 7: Probably because we sit in an area that number one is a food desert there are no grocery stores around for anybody.

00:02:26.400 --> 00:02:26.790

Interview Participant 7: We.

00:02:26.850 --> 00:02:30.390

Interview Participant 7: are in the middle of the minority community.

00:02:31.620 --> 00:02:35.340

Interview Participant 7: Our office is located in underserved population area.

00:02:37.620 --> 00:02:53.160

Interview Participant 7: So we're able to get those kits out to people a little bit easier because we wanted to be our office to be located, where people who did not have access to cars, we are on a bus line we're very easily accessible to people.

00:02:56.040 --> 00:02:57.300

Interviewer: Thank you, thank you.

00:02:58.350 --> 00:02:59.370

Interviewer: And how do you feel.

00:03:00.630 --> 00:03:04.170

Interviewer: about the say yes covid test projects ability to address.

00:03:05.850 --> 00:03:09.390

Interviewer: Your county's covid 19 needs.

00:03:09.570 --> 00:03:11.910

Interview Participant 7: When our organization became involved.

00:03:13.920 --> 00:03:23.070

Interview Participant 7: Initially we were doing Okay, I think the health department hindered us significant significantly in being able to serve.

00:03:23.820 --> 00:03:39.870

Interview Participant 7: The minority and underserved populations, because when they became involved the focus was not just on the minority and underserved it was getting the test out to anyone and I think that's where we kind of missed the mark a little bit.

00:03:45.240 --> 00:03:49.020

Interviewer: Okay, can you elaborate a bit on by missing the mark.

00:03:50.190 --> 00:03:59.760

Interview Participant 7: Well, initially the program target says we want to get these test kits out to minority underserved populations that was the goal.

00:04:00.570 --> 00:04:13.740

Interview Participant 7: when the health department came in in the Community partners, they worked with we're not targeting minority underserved populations, so we have a whole set of kits they were setting up.

00:04:14.100 --> 00:04:18.960

Interview Participant 7: tents and booths where minority and underserved populations could not even get to.

00:04:21.360 --> 00:04:29.790

Interview Participant 7: So it it those kits were not getting to anybody, but the majority population, so it was not hitting.

00:04:29.880 --> 00:04:31.590

Interview Participant 7: The target a group at all.

00:04:34.140 --> 00:04:35.970

Interview Participant 7: We had one side hitting.

00:04:36.300 --> 00:04:41.100

Interview Participant 7: The target a group in one side hitting that not targeted Group and

00:04:42.240 --> 00:04:44.190

Interview Participant 7: So we were working against each other.

00:04:45.450 --> 00:04:48.870

Interviewer: and expand, thank you for that information.

00:04:50.370 --> 00:05:01.290

Interviewer: Now I have a couple of questions about the communication that you receive for the say yes covid test program So the first one is.

00:05:02.430 --> 00:05:07.500

Interviewer: Can you describe how you were initially contacted about the say yes covid test program

00:05:09.360 --> 00:05:11.760

Interview Participant 7: Yes, I was initially contacted.

00:05:12.960 --> 00:05:21.540

Interview Participant 7: By Daphne from Oregon who actually worked with here in chattanooga at one point she was executive director of a CDC here.

00:05:22.410 --> 00:05:35.100

Interview Participant 7: And she said that chattanooga was potentially going to be the location for the say yes, took the test, and she asked if I could sit in on the Advisory Board since i've worked in.

00:05:35.100 --> 00:05:41.550

Interview Participant 7: Healthcare and I joined that meeting, and that day I signed our office my office.

00:05:44.370 --> 00:05:54.000

Interview Participant 7: to become a Community partner after the initial meeting and then signed up another organization that I participated with as well.

00:06:01.980 --> 00:06:02.370

Interviewer: Sorry.

00:06:05.220 --> 00:06:06.060

Interviewer: Can you.

00:06:09.120 --> 00:06:09.600

Interviewer: say hello.

00:06:14.010 --> 00:06:17.340

Interview Participant 7: i'm sorry the connection went out for a minute it's storming here.

00:06:19.620 --> 00:06:21.120

Can you repeat your question.

00:06:27.000 --> 00:06:27.630

Interviewer: Can you hear me.

00:06:28.620 --> 00:06:29.640

Interview Participant 7: Now I can.

00:06:31.590 --> 00:06:33.540

Interview Participant 7: Just the screen is frozen.

00:06:33.900 --> 00:06:34.350

Oh.

00:06:41.550 --> 00:06:47.010

Note Taker: I think what Donaldson was asking was talking about, what are the two.

00:06:48.120 --> 00:06:50.850

Note Taker: And Community boards that you're on are the two.

00:06:52.710 --> 00:06:53.970

Note Taker: Organizations you work with.

00:06:54.390 --> 00:06:57.990

Interview Participant 7: Yes, other organization was junior league of chattanooga.

00:06:58.680 --> 00:07:02.490

Interview Participant 7: Because we work on getting with other.

00:07:06.810 --> 00:07:15.120

Interview Participant 7: We partner with working throughout the Community, so I knew that they would have a hand and be able to get the test kits out to the entire Community.

00:07:18.540 --> 00:07:20.640

Interviewer: Come back, can you hear me now heather sorry.

00:07:20.670 --> 00:07:21.450

Yes.

00:07:22.470 --> 00:07:28.590

Interviewer: Sorry, the last question I asked was about you mentioned, you were contacted by Daphne.

00:07:28.980 --> 00:07:33.780

Interviewer: Yes, and I was wondering if you could share a little bit more about who, that is.

00:07:34.140 --> 00:07:40.260

Interview Participant 7: Oh yes, she works worked for me to development corporation in.

00:07:41.490 --> 00:07:49.260

Interview Participant 7: chattanooga before moving to North Carolina and she had worked without Richmond at CDC

00:07:50.340 --> 00:08:00.690

Interviewer: Okay Okay, thank you, thank you for the clarification and, in your opinion, was the communication strategy that definitely use the best approach.

00:08:01.320 --> 00:08:11.370

Interview Participant 7: Yes, probably because she lived in chattanooga for many years, so everybody that she brought to the table, was able to help with the project.

00:08:14.250 --> 00:08:15.360

Interviewer: Thank you, thank you.

00:08:16.560 --> 00:08:26.910

Interviewer: And the project can you share or what your thoughts are about the communication that occurred throughout the whole project overall.

00:08:27.720 --> 00:08:44.730

Interview Participant 7: Now, I think it was very good communication because Angela followed up with us very regularly throughout the project to check and see how many kits were kind of around with each organization what each organization was doing what kind of events, we were having.

00:08:47.700 --> 00:08:57.360

Interview Participant 7: You know I even you know, we had our website for back line you know so people could come by because I worked like pretty much every day and pick up kits.

00:08:59.700 --> 00:09:01.800

Interview Participant 7: So it really what function very well.

00:09:03.120 --> 00:09:10.260

Interviewer: Thank you, and what what made you or your organization decide to participate in this project.

00:09:12.690 --> 00:09:20.640

Interview Participant 7: We, I guess, being a healthcare facility ourselves as healthcare providers, we saw the need to have that ability.

00:09:21.930 --> 00:09:27.690

Interview Participant 7: For our patients at home, because we were in undated quite a few times.

00:09:29.100 --> 00:09:32.160

Interview Participant 7: with high covid testing and protocols.

00:09:33.300 --> 00:09:51.720

Interview Participant 7: You know the Ad home testing and showing people you're not getting covid with by testing number one and number two showing regular testing is OK, you know would be very beneficial to minority underserved populations just continual education.

00:09:54.150 --> 00:10:02.820

Interviewer: Thank you, thank you, I am the spin makes sense and after you were contacted for the project.

00:10:03.960 --> 00:10:07.110

Interviewer: work for us to do specifically if you.

00:10:10.710 --> 00:10:14.940

Interview Participant 7: Well we had to take a three week pause while they waited on.

00:10:17.190 --> 00:10:33.630

Interview Participant 7: Some decisions to come back from the CDC then we kind of pick back up and I kind of made a list of all the Community partners that I felt we should bring in and there were still some doctors offices, we had we brought in.

00:10:35.670 --> 00:10:50.370

Interview Participant 7: Especially Obgyn to took all 10 cares just because they were serving an underserved population and those women need to be tested regularly as well, so they could be educated.

00:10:51.300 --> 00:11:09.990

Interview Participant 7: It kind of made a list the health department had their own kind of list and they contacted the food bank and a couple of more organizations, so we had kind of two separate list of health department listed in the list that I had with all the Community partners.

00:11:11.580 --> 00:11:22.950

Interviewer: Sorry, essentially from, if I understand correctly you're asked to create a list of potential organizations that should be contacted for the project.

00:11:23.370 --> 00:11:27.270

Interview Participant 7: Right and I reached out to most of them.

00:11:30.090 --> 00:11:31.560

Interview Participant 7: For the testing.

00:11:33.360 --> 00:11:35.250

Interview Participant 7: process, I talked with them.

00:11:37.020 --> 00:11:42.960

Interview Participant 7: On our side and either I did or Dr hubbard if I wasn't available to talk with them.

00:11:43.920 --> 00:11:46.560

Interviewer: Thank you, thank you, sounds like.

00:11:47.610 --> 00:11:54.540

Interviewer: A very you are able to come up with a comprehensive list and also there was another list you mentioned that was from the health.

00:11:55.020 --> 00:11:57.060

Interview Participant 7: department right had a separate list.

00:11:57.870 --> 00:12:02.940

Interview Participant 7: Okay, and I don't know how, and we would never privy to how they got there.

93

00:12:04.200 --> 00:12:11.520

Interviewer: Okay, thank you, and what resources did you require to complete.

00:12:12.660 --> 00:12:21.450

Interviewer: You know this list that you are, you know you created then maybe other we tasks that your organization carried out for the project.

00:12:23.010 --> 00:12:40.710

Interview Participant 7: um it was not that bad for me to create the list because I served on other nonprofit boards and everything, so I could literally go to a spreadsheet and copy and paste because all of those organizations needed to be helping in a sense, with this project.

00:12:42.480 --> 00:12:48.030

Interview Participant 7: it's about getting all of the minority organizations involved because that's the work they're already doing.

00:12:50.220 --> 00:13:01.650

Interview Participant 7: And so it was a matter of setting them setting up the events aware they were going to pass out these kits and so that's kind of how we did it and.

00:13:02.370 --> 00:13:16.350

Interview Participant 7: You know, we set up some individual meetings for churches, who had a few additional questions, but other than that it was very kind of simple everybody setting up what day they were having their events.

00:13:18.090 --> 00:13:23.010

Interviewer: Thank you, thank you so once you made the contact with those organizations, they were able to then.

00:13:23.730 --> 00:13:24.570

Interview Participant 7: Our green.

00:13:25.140 --> 00:13:29.730

Interviewer: bay and then share information about how they would distribute the kits.

00:13:29.760 --> 00:13:30.030

Get.

00:13:31.980 --> 00:13:32.940

Interviewer: Thank you

00:13:34.140 --> 00:13:42.990

Interviewer: and during your time working in assisting with the project did you have any questions for the project team at all.

00:13:44.790 --> 00:14:00.420

Interview Participant 7: i'm not that many I mean I probably takes Angela here and there about other stuff that we were you know, had more questions about you know just making sure that everybody was getting their kits out that wasn't being.

00:14:01.470 --> 00:14:02.100

Interview Participant 7: You know.

00:14:03.330 --> 00:14:12.330

Interview Participant 7: One doctor was going through kits so quickly, it was almost hard to keep him with kits so you know it was just.

00:14:14.340 --> 00:14:23.370

Interview Participant 7: He was picking them up so regularly so it's just you know flowing into so you know just making sure that she knew the counts, every week and making sure.

00:14:24.120 --> 00:14:29.790

Interview Participant 7: You know that they're all information was related back to North Carolina timely fashion.

00:14:30.150 --> 00:14:45.720

Interview Participant 7: So I usually did every Thursday or Friday I tried to meet with Angela for at least you know 20 minutes to give her you know all the information so if they hadn't been the following Saturday or Sunday, she would know how many kits they were taking out to those have been.

00:14:48.720 --> 00:15:07.320

Interviewer: Thank you, thank you so essentially the questions you had were minimal you know, very few and when the when the when the when you were in the process of communicating with Angela about your questions, can you share how responsive she.

00:15:07.530 --> 00:15:16.890

Interview Participant 7: have been very, very responsive even still Angela and I still you know take to this day about you know little things here and there, I mean.

00:15:17.370 --> 00:15:28.710

Interview Participant 7: cuz she's very quick to respond, you know, even if you have to call her if she was busy she turns around and calls you right back, I mean she was very timely very easy to work with, so

00:15:29.550 --> 00:15:31.620

Interview Participant 7: we've never had a problem at all.

00:15:43.110 --> 00:15:44.100

Interviewer: Okay, great.

00:15:45.360 --> 00:15:48.870

Interviewer: i'm happy to hear that can you still hear me, yes, yes.

00:15:52.830 --> 00:16:03.450

Interviewer: Okay, thank you so The next question is, do you think the project address accounting needs related to covid 19 health concerns.

00:16:04.920 --> 00:16:10.980

Interview Participant 7: And it did, I just wish the health department kind of stuck with the guidelines for the project.

00:16:12.180 --> 00:16:15.480

Interview Participant 7: It did significantly it helps significantly.

00:16:16.830 --> 00:16:29.370

Interview Participant 7: About three weeks after we finished the project here in chattanooga we had a significant spike in covid cases and everybody was looking for test kits and there were none available, so I mean.

00:16:30.690 --> 00:16:37.140

Interview Participant 7: It I wish we could have had run the test a little longer here and had more kids but that just wasn't feasible.

00:16:39.300 --> 00:16:40.380

Interview Participant 7: I mean, because we had a.

00:16:40.380 --> 00:16:40.890

Interview Participant 7: spike.

00:16:40.950 --> 00:16:50.970

Interview Participant 7: That was so significant I mean we went from seeing you know 45 patients a day to 60 to 75 a day, and I mean.

00:16:52.110 --> 00:16:59.790

Interview Participant 7: At one point, we were seeing just 25 people have covid it was just cope with patients, I mean, so it was we were inundated.

00:17:00.060 --> 00:17:10.770

Interview Participant 7: So we would have been helpful for health care providers, you know tab parents, where they could regularly test their children right and then bringing them three times a week because I just had to be tested.

00:17:12.990 --> 00:17:23.970

Interviewer: Thank you, thank you so essentially based on your observations you can you elaborate a little bit more about what the needs were in your county related to covid 19 health.

00:17:25.980 --> 00:17:28.500

Interviewer: covid 19 in general, based on what you just said.

00:17:28.920 --> 00:17:29.460

Yes.

00:17:30.600 --> 00:17:35.730

Interview Participant 7: The school system did not have for let's say the.

00:17:39.210 --> 00:17:54.450

Interview Participant 7: school year did not have good measures in place for covid testing in so and, as far as what was needed for child to return to school so.

00:17:54.960 --> 00:18:05.310

Interview Participant 7: They were sending them back to the doctor's office for another covid test and then, if somebody was exposed in the classroom again, you had to be retested again and so.

00:18:05.550 --> 00:18:13.770

Interview Participant 7: You know, and some children desk three times in a week they're going to be there, and so it was just you know we were inundated with key is because.

00:18:14.220 --> 00:18:21.180

Interview Participant 7: Everybody was getting delta that point, and so it was just a massive amounts, and so we weren't really.

00:18:22.050 --> 00:18:35.100

Interview Participant 7: Having to call the school system to say we got to come up with a plan you know, having to call the health department, we need help to guide us, because we can't be over in undated we are going to run out of tests at some point.

00:18:35.790 --> 00:18:52.680

Interview Participant 7: And so it was the need, at that point for more structure and, like the CDC to step in and say you don't need to retest and unless you know you're symptomatic still and you've gone beyond 14 days like.

137

00:18:53.940 --> 00:18:59.640

Interview Participant 7: You know, almost like they're doing now, but they should have had that last August.

00:19:01.020 --> 00:19:09.750

Interviewer: Thank you, and based on this need that was there, how do you think the sales overnight thing project fit in.

00:19:11.100 --> 00:19:15.780

Interviewer: To help address in the if at all of those needs.

00:19:16.290 --> 00:19:22.080

Interview Participant 7: Because with the test kits saying that people needed to test three times a week the parents would have had.

00:19:22.470 --> 00:19:30.540

Interview Participant 7: Those test kits to test those children who are the appropriate age three times a week and they could take a picture of it.

00:19:31.170 --> 00:19:52.350

Interview Participant 7: And you know if it was positive, they get contact us and we could have told them what other medicines to take, we could say you know take username stay hydrated watch, for you know temperatures use ibuprofen and tylenol alternating you know, and we could have say you know some resources.

00:19:54.150 --> 00:20:04.080

Interview Participant 7: Because it started to be a lot harder to get testing the state get us off from getting rapid covid test from them, you know we weren't limited.

00:20:06.600 --> 00:20:16.290

Interview Participant 7: Until it became a hard hard to get the resources to help our patient population that we serve, which is the minority underserved population.

00:20:18.540 --> 00:20:26.520

Interviewer: Thank you, thank you very much for sharing that information and how the project was able to help some of the needs for the county.

00:20:28.020 --> 00:20:35.070

Interviewer: related to what you shared, what do you think are some of the biggest barriers to covid 19 testing.

00:20:36.210 --> 00:20:40.290

Interviewer: Now, and also before doing the earlier, the pilot.

00:20:41.310 --> 00:20:42.930

Interview Participant 7: it's you know, the net.

00:20:43.980 --> 00:20:48.450

Interview Participant 7: Especially in the black Community bit covid that team testing.

00:20:48.720 --> 00:20:50.070

Interview Participant 7: gives you cope it.

00:20:50.550 --> 00:20:51.270

um.

00:20:53.820 --> 00:20:54.960

Interview Participant 7: And so.

00:20:56.850 --> 00:20:58.890

Interview Participant 7: And then that plays into.

00:21:00.270 --> 00:21:15.060

Interview Participant 7: If you get the vaccine is going to give you a covid and so it's that saying is driving that and so people literally are fearful to get covid tested, and so we are spending.

00:21:15.600 --> 00:21:25.320

Interview Participant 7: 30 minutes educating patients on the importance of covid 19 testing and some parents will go to the emergency room.

00:21:26.160 --> 00:21:40.740

Interview Participant 7: and want to know if their child may have coded but refused covid testing, and so it is you know we are spending a lot of time and energy but there's not the lack of understanding.

00:21:41.520 --> 00:21:54.990

Interview Participant 7: Just because it has become this great thing that is spread in the Community from people not having true knowledge and medical understanding that coven 19 testing does not give you go.

00:21:57.120 --> 00:22:00.030

Interviewer: Thank you, thank you and.

00:22:01.140 --> 00:22:12.810

Interviewer: Based on what you just shared do you think this as a test project responded well to that specific myth or barriers to covid 19 testing.

00:22:13.980 --> 00:22:21.960

Interview Participant 7: think it did, because we spent so much time with the families and parents, really, explaining to them even more.

00:22:22.980 --> 00:22:39.000

Interview Participant 7: And so, when delta really peaked they really got to realize that maybe you know what the doctors are saying it's true they were still having vaccine hesitancy, but they were not having as much hesitancy with the testing.

00:22:39.360 --> 00:22:49.890

Interview Participant 7: They were more willing to allow their child to get tested versus saying no I don't want them to be tested, because those who refuse tested decide to be quarantined.

00:22:51.000 --> 00:22:52.080

Interview Participant 7: For 14 days.

00:22:54.150 --> 00:22:55.950

Interviewer: Thank you i'm very happy to hear that.

00:22:57.000 --> 00:22:59.430

Interviewer: The project was helpful in that regard.

00:23:03.000 --> 00:23:13.260

Interviewer: So, at the end of the project, how close, do you think the typical activities match what you were told in the beginning of the project.

00:23:15.120 --> 00:23:18.060

Interview Participant 7: On our side, they did.

00:23:19.440 --> 00:23:27.330

Interview Participant 7: I would say about 98% on the health department side, I would probably say there's may come in at 50%.

00:23:29.970 --> 00:23:33.570

Interviewer: Okay Okay, and can you elaborate a little bit about what.

00:23:33.630 --> 00:23:37.410

Interview Participant 7: Well it's because the health department did not.

00:23:38.460 --> 00:23:50.610

Interview Participant 7: was still not following we were supposed to be serving the minority underserved populations, which include you know Spanish the homeless, you know.

00:23:51.900 --> 00:24:03.210

Interview Participant 7: And they were not targeting those areas at all so and then you know they were holding kits for their employees and things like that.

00:24:03.900 --> 00:24:09.240

Interview Participant 7: Whereas we are getting those kits out to the Community to those who are in need.

00:24:09.960 --> 00:24:21.600

Interview Participant 7: who do not have access, you know the health department employees have access to rapid testing kits through their job and things like that, so it was a very big.

00:24:21.600 --> 00:24:25.260

Interview Participant 7: difference in one side or the other we're doing.

00:24:26.100 --> 00:24:27.060

Interviewer: Okay okay.

00:24:27.090 --> 00:24:28.350

Interview Participant 7: So it was almost like running.

00:24:28.620 --> 00:24:30.990

Interview Participant 7: Two separate studies, at the same time.

00:24:34.380 --> 00:24:43.110

Interviewer: And, did you find any of the task that was asked of you, by the say yes covid test project difficult.

00:24:44.400 --> 00:24:45.600

Interview Participant 7: Oh no not at all.

00:24:47.580 --> 00:24:48.360

00:24:49.800 --> 00:25:12.720

Interview Participant 7: Well, I guess, probably sociology prepared in college, and so you know I had to do thesis on healthcare stuff as to graduate and so this was just kind of like running My thesis all over again because I had to study this to graduate so it was almost like like me running a steady like.

00:25:15.240 --> 00:25:17.730

Interviewer: Your your experience came in very handy.

00:25:18.180 --> 00:25:33.960

Interview Participant 7: Yes, so it was just like that in I knew the data kind of like I had died I knew all of that was going to take place, and so I knew we had to preserve that patient population, and you know.

00:25:35.010 --> 00:25:40.530

Interview Participant 7: And that's what I was kind of looking at everybody's like what what is the scope, what are we going to do.

00:25:41.970 --> 00:25:47.640

Interview Participant 7: And so that's why on our side, we really tried to make sure we were getting those kits out to be stayed.

00:25:49.500 --> 00:26:00.180

Interviewer: Thank you, thank you, as good to is to understand your background and how it helped you in the in responding to the needs for the project.

00:26:01.320 --> 00:26:02.400

Interviewer: and also a community.

00:26:03.540 --> 00:26:17.430

Interviewer: The next question is what are some of the reasons individuals in your Community ordered the test kits and they touched on this a bit earlier, but for the day, what are the biggest reasons that motivated people.

00:26:18.210 --> 00:26:20.940

Interview Participant 7: I think some people mmm.

00:26:22.080 --> 00:26:41.280

Interview Participant 7: Initially it was older people, because they wanted to be a sure they could test themselves, it was a demographic but we about 40 to 60 they were very serious about testing themselves and then.

00:26:42.600 --> 00:26:49.320

Interview Participant 7: When school teachers realize how close it was come into school and they weren't going to be certain protocols.

00:26:50.100 --> 00:26:59.910

Interview Participant 7: They ordered started ordering and coming to get kit because they said we're going to need the tests regularly kids won't be wearing masks this year.

00:27:00.390 --> 00:27:10.440

Interview Participant 7: You know I need to protect myself, and so you know they started thinking long term I can't get cold but i'm only going to have so many days off work.

00:27:11.460 --> 00:27:32.430

Interview Participant 7: And so people really started thinking of the long term effects of you know what happens if I get covid and so those people kind of said, let me order kits online and if they were no more online, then they started calling and that's how we kind of progressed to getting you know here.

00:27:34.860 --> 00:27:39.930

Interviewer: Thank you, and by referring to the online option for the test kits in

00:27:40.860 --> 00:27:43.020

Interviewer: chattanooga Thank you.

00:27:43.380 --> 00:27:46.320

Interviewer: and which method, do you think was preferred.

00:27:46.890 --> 00:27:50.130

Interviewer: In terms for ordering online or the picking up.

00:27:51.420 --> 00:28:02.130

Interview Participant 7: For their people who were 65 and up they prefer to come pick it up for people under 65.

201

00:28:03.330 --> 00:28:10.800

Interview Participant 7: They like the option to order it online, but there is a population who.

00:28:13.980 --> 00:28:21.300

Interview Participant 7: If their income falls below a certain amount that they wanted to pick up those kits as well.

00:28:22.740 --> 00:28:23.130

Interviewer: Okay.

00:28:23.220 --> 00:28:25.350

Interview Participant 7: Because they had to be encouraged.

205

00:28:26.640 --> 00:28:38.190

Interview Participant 7: To understand what it was, and then they would be willing to get the kits because we walked around beauty shop Barber shop, I mean all of those types of things and they say Oh, this is what we need.

00:28:40.230 --> 00:28:48.930

Interviewer: Thank you, thank you that's very, very helpful to know about the difference in terms of the age groups and their preference.

00:28:50.820 --> 00:29:02.190

Interviewer: Thank you, and what, if any recommendations, do you have for the say yes covid test project on how to improve their approach.

00:29:03.960 --> 00:29:04.560

Interview Participant 7: um.

00:29:08.730 --> 00:29:16.950

Interview Participant 7: I would say, if the health department would work with the Community Partner lead a little bit more.

00:29:18.360 --> 00:29:32.280

Interview Participant 7: So, then they are following that direct those that would be you know so everybody is doing the same thing, instead of it really being like two separate research projects at the same time, because.

00:29:32.940 --> 00:29:50.040

Interview Participant 7: It would be helpful, so that they know let's pull these Community partners that are going to get these kits you know, yes, it was great to get the kits to firefighters and you know EMS workers, however, that was not necessarily be targeted.

00:29:51.600 --> 00:29:55.590

Interview Participant 7: audience, because in chattanooga you can go.

00:29:57.060 --> 00:29:58.230

Interview Participant 7: To the.

00:29:59.250 --> 00:30:01.860

Interview Participant 7: same employee help and get tested.

00:30:03.900 --> 00:30:07.200

Interviewer: Thank you, thank you, I think that's a great recommendation.

00:30:08.880 --> 00:30:18.180

Interviewer: And lastly, what would you like to see happen in your community in the health in general.

00:30:19.980 --> 00:30:30.930

Interview Participant 7: I would really like to see you know that other physician and the health department in general get.

00:30:31.440 --> 00:30:41.040

Interview Participant 7: More people focus on minority and underserved populations, because there are very few doctors that accept insurance for the minority and underserved.

00:30:41.460 --> 00:31:03.090

Interview Participant 7: You know the health department will see some of them, but they do not spend an abundance of time like on this project getting these things you know out to them when the when matters come to things like this, it seems like their focus is on the population who can get to them.

00:31:04.440 --> 00:31:07.920

Interview Participant 7: Which is not always the minority in underserved community.

00:31:09.120 --> 00:31:10.080

Interviewer: Yes, yes.

00:31:11.700 --> 00:31:12.630

Interviewer: Yes, kind of like.

00:31:13.830 --> 00:31:17.910

Interviewer: Maybe picking the foods that are.

00:31:18.090 --> 00:31:18.510

Interviewer: easier.

00:31:19.230 --> 00:31:20.940

Interview Participant 7: To reach and so.

00:31:21.960 --> 00:31:30.720

Interview Participant 7: you're going all the way to little while with just part of Hamilton county but I guarantee you none of the minority underserved populations can get to La.

00:31:31.980 --> 00:31:37.650

Interview Participant 7: So or absent, you know it may be handled it can feel it they can't get out that far.

00:31:38.550 --> 00:31:39.360

Interviewer: and stand.

00:31:39.420 --> 00:31:52.260

Interview Participant 7: They're right over here by my office or in the West side you all are going over there and then, if you send one of your nurses, they say I don't want to be over here it's too dangerous is the complaint i've always heard.

00:31:53.910 --> 00:31:57.240

Interviewer: Stan Stan Thank you, thank you.

00:31:58.500 --> 00:32:03.360

Interviewer: And lastly, do you have any additional questions or comments.

00:32:04.860 --> 00:32:12.720

Interview Participant 7: um I really enjoyed the project I gotta ask recently, for it to be brought back to chattanooga.

00:32:14.130 --> 00:32:18.900

Interview Participant 7: You know the black nurses association would love to get more kits here to get them out.

00:32:20.310 --> 00:32:33.630

Interview Participant 7: Especially since we're having another little spike you know these people here in chattanooga really need it, I think they need continual education, especially since chattanooga has grown as a tech savvy.

00:32:34.410 --> 00:32:46.980

Interview Participant 7: it's put the minority underserved population in much more cramped quarters as you would call it, it is caused an influx of being.

00:32:47.850 --> 00:33:06.930

Interview Participant 7: more of them are having to live in one House more people are sharing two bedroom apartments, so I would really love for it to come back because more families are living together more generational families, you see, three and four generations and one home now.

00:33:09.390 --> 00:33:12.000

Interviewer: wow, thank you for sharing that and I pray that.

00:33:13.260 --> 00:33:14.550

Interviewer: They bring it back and.

00:33:15.840 --> 00:33:19.020

Interviewer: help to continue to address the needs of the Community.

00:33:20.940 --> 00:33:22.770

Interview Participant 7: We would love to see it come back here.

00:33:24.780 --> 00:33:29.100

Interviewer: I hope the team will will bring it back and have the resources to bring it back.

00:33:31.020 --> 00:33:41.490

Interviewer: Thank you very much heather I really appreciate you making the time to meet and speak with me I don't have any more questions at this moment.

00:33:42.630 --> 00:33:44.640

Interviewer: And if you don't have any questions.
