# Supplementary material for: Exploring barriers and facilitators of implementing an at-home SARS-CoV-2 antigen self-testing intervention: The Rapid Acceleration of Diagnostics—Underserved Populations (RADx-UP) initiatives
Source: PLoS One. 2023 Nov 16;18(11):e0294458. doi: 10.1371/journal.pone.0294458 (PMC10653400; doi:10.1371/journal.pone.0294458)
Supplement: S1 Dataset — (ZIP) [file pone.0294458.s002.zip › P'sID(9)-Notes (08.17.22).docx]

1. Utilize her practice as a facilitator – pediatrician. Gain knowledge so she could encourage her patients to test. She is the owner of a primary care facility
2. Located in an urban area so they could share the information with
3. Good project n terms of disseminating the test kits. There was not a clear disconnect between the test and medication against covid. Didn’t make the connection that it was a screening test and not a vaccination
4. Chattanooga selected as a site – women who was reaching out to community leaders knew Willie Hubbard. Can’t recall which organization the woman was with who contacted her
   1. Email followed up with a phone call
   2. Appropriate way to call someone personally
5. Her position and career made her think it was pertinent that she participated
6. One of the sites to hold the kits for distribution
7. Very similar, encouraging people to take a box home and talk to patients about the kits/screeners. Intergrated into what she did every day.
8. HPV vaccine similarity and flu
   1. Mentions black community and additional hesitations
9. The time required to explain a test during the appointment and when they were walking out. Training staff and everyone in the practice
10. Very responsive to their questions when they did have some about clarification
11. Very responsive to their needs and were able to connect to people in the inner-city and numerous events
12. Self-explanatory and clear. Everything fell into place
13. Yes, participate in major health fairs to give away kits – part of organizing the event and used to doing projects like this
14. It did but when the kits were still there the marketing could have been improved. It was not enough before distributing the kits because people were not informed enough and were fearful of the disease itself so they didn’t want to be involved in anything – marketing it better would have made it better. All of the efforts worked but it didn’t make people comfortable because of the set period of time.
15. People understood that the kits were able to be used at home and not go to the doctor which made it simple to test.
16. COVID-19 has become very political. She is a firm believer in immunization but minorities in her practice won’t get vaccinated. People will come out with covid and not wear the mask. People are divided for political reasons and others are fearful they’re being tested on.
17. Supply of tests. If people must pay for the test, usage will decrease. Incentives to everyone who got a covid test. Fear and political differences. Continue to perfect the test for low cost or free, they will be utilized
18. Positive affect on the community. Retrospect and reflect how significant it is.
19. Once people became more knowledgeable, they trusted the test
20. Attitudes of the home testing kits became significant in the lives of everyone who participated. Towards the end everyone as calling for additional tests. Acceptance of the kits grew
21. Anyone with symptoms had to be tested for covid in the car and couldn’t come into the office
22. Tasks were difficult because it took so much time, and it was a problem getting people to accept the kits
23. Told them it was a safe thing to do. The news talked about test kits. Positive tests kits allowed people to stay home with pay.
24. Did not have the option to order test kits online initially, maybe towards the end
25. Those who are used to the computer online ordering was bad, large population needs to have it be convenient to pick it up
26. Advertising needs to be improved and establish positive relationships with the people and the facilities distributing them
27. 1-2 weeks before the event – not enough time to demonstrate the procedure and discuss the pros of the kits
28. Community providers need to be more supportive of high-risk populations who may not understand the importance of appointments. Provide more education overall and services tangible

Debriefing

- She stated that the tasks were difficult – could be because she is a physician and has her own group of patients to educate
- Question about online ordering – no one seems to know what that means or that it was an option
- She had clear answers and didn’t contradict herself
- She needed more rephrasing of the questions than other participants
- Overall understood what was being asked of her and focused
- Mostly spoke about distributing the test kits and then spoke about role of coordinating with other facilities
