# Supplementary material for: Exploring barriers and facilitators of implementing an at-home SARS-CoV-2 antigen self-testing intervention: The Rapid Acceleration of Diagnostics—Underserved Populations (RADx-UP) initiatives
Source: PLoS One. 2023 Nov 16;18(11):e0294458. doi: 10.1371/journal.pone.0294458 (PMC10653400; doi:10.1371/journal.pone.0294458)
Supplement: S1 Dataset — (ZIP) [file pone.0294458.s002.zip › SI 1.03_Semi-Structured_Interview_Guide.docx]

###### SAY YES! COVID-19 TEST

###### SEMI-STRUCTURED INTERVIEW

**1. Interview Date ___/___/___**

**1a. Interview Method On-Site: ______ Phone: _______**

**2. Interviewer ID _________**

**3. Individual Type: ________________________________________________**

[**Note to interviewer**: ALL ITALICIZED WORDS SHOULD BE SPOKEN. PLEASE FOLLOW ALL SKIP PATTERNS. MARK YOU ANSWER WITH AN **X** IN THE APPROPRIATE BOX.]

*Today’s interview will be more formal than a regular conversation. I have a series of questions to ask you, and I will need your help to stay on course and ask all of them.* ***We are talking to/interviewing individuals of various positions and professions that were part of the planning community advisory team. Your role in recruiting and securing community organizations was a primary function of this project.*** *Therefore, it is important that I ask you each question as it is written. Of course, if you do not understand a question, or if you need for me to repeat a question, please let me know. For your time you will receive $100 gift card.*

*Thank you for agreeing to talk with me today. This interview will last approximately 30 minutes. I will ask questions about you, your knowledge/understanding about your county’s health needs, health priorities, available resources, and gaps in resources or other access related issues. There are no right or wrong answers. If there are any questions that you would rather not answer, you are free to decline to answer them. All of your responses will be kept confidential – meaning that only the research team will know your responses.* *When we summarize the results, we will report them across participants in the project, and no individual names of participants will be used without their permission. Lastly, as mentioned in the informed consent, we would like to record this interview, we will keep your private information separate from the recording and will destroy the recording after they are transcribed. Do I have your permission to record this interview?*

*If it’s ok with you, let’s begin…*

#### START TIME: ______ _______:______ ______

| **LET’S BEGIN WIT**  **H SOME QUESTIONS ABOUT YOUR ROLE AT THE PARTICIPATING ORGANIZATION.** | |
| --- | --- |
|  | **What is the primary mission for your organization?** |
|  | **What is your position at your organization?** |
| **NEXT I HAVE SOME QUESTIONS ABOUT YOUR ORGANIZATION AND THE SYCT PROJECT.** | |
|  | **In your opinion, what made you or your organization a good partner for the SYCT project?** |
|  | **How do you feel about the SYCT project’s ability to address your county’s COVID-19 needs?** |
| **Now, I am going to ask you some questions about the SAYYES! COVID TEST Program** | |
|  | **Can you describe how you were initially contacted about this program?**  **PROBES:**   - **In your opinion, was this the best communication approach?** - **What are your thoughts about the communication across the project?** |
|  | **What made you or your organization decide to participate in this project?**  **PROBE:**   - **What were you asked to do for this project?** |
|  | **Based on your answer to Q7, how close were these activities to your regular tasks?** |
|  | **What resources did you require to complete these tasks by your organization?**  **PROBE:**   - **What resources did you require to complete these tasks by the SYCT team?** |
|  | **Did you have any questions for the project team during your collaboration with them?**  **PROBES:**   - **How responsive were the project team members to your questions?** - **How responsive were the project team members to your needs?** |
|  | **Do you think the project addressed a county need related to COVID-19health concerns?**  **PROBES**   - **What were the needs in your county related to COVID-19 health?** |
|  | **What do you think are the biggest barriers to COVID-19 testing?**  **PROBES:**   - **Do you think that the SYCT project responded well to barriers to COVID-19 testing?** - **Why or why not?** |
|  | **At the end of the project, how close did your typical activities match what you were asked to do for the SYCT project?**  **PROBES:**   - **Did you find the tasks asked of you by the SYCT project difficult?** |
|  | **What are some reasons individuals in your community ordered the test kits?**  **PROBES:**   - **What do you think is the biggest reason that people in your county picked up a testing kit?** - **What do you think is the biggest reason that people in your county ordered a testing kit using the online ordering options?** - **Which method was preferred?** |
|  | **Do you have any recommendations for the SYCT to improve their approach?** |
| **Lastly, please provide any thoughts you have about the following:** | |
|  | **What would you like to see happen with your community and their health?** |
|  | **Do you have any additional questions or comments?** |
| **THOSE ARE ALL OF THE QUESTIONS I HAVE. THANK YOU VERY MUCH FOR YOUR TIME AND HELP.** | |
