# Supplementary material for: Exploring barriers and facilitators of implementing an at-home SARS-CoV-2 antigen self-testing intervention: The Rapid Acceleration of Diagnostics—Underserved Populations (RADx-UP) initiatives
Source: PLoS One. 2023 Nov 16;18(11):e0294458. doi: 10.1371/journal.pone.0294458 (PMC10653400; doi:10.1371/journal.pone.0294458)
Supplement: S1 Dataset — (ZIP) [file pone.0294458.s002.zip › CAFG-Transcript (9.03.22).doc]

**INTERVIEWER:**  So, let’s start the discussion by talking about what makes your city a good place to live. What are some things that you enjoy about living in your city? Anyone can jump in.

**CA129:** I will start. I have children and grandchildren, and there are a lot of activities for kids, theatre activities, we have three different theatre organizations, a lot of sports, a lot of dance, music, art, so it’s a great place to live and raise a family because of all of those wonderful activities for children and adults.

**INTERVIEWER:** Thank you, and where do you live, exactly, if you don't mind sharing?

**CA129:** Oh, I live in Atwater, California, but we primarily interact with activities in Merced, which is 7 miles south from us. Atwater is a smaller community outside of Merced.

**INTERVIEWER:** Thank you, and --?

**CA97:** I don't know how could respond to this one, because I work in Merced County, work in Atwater, but I do not live in Merced County.

**INTERVIEWER:** You say you cannot live?

**CA97:** I do not live in Merced county, but I work in Merced county.

**INTERVIEWER:** Okay, okay.

**CA97:** I can respond to my experiences of working here.

**INTERVIEWER:** Yes, and also where you live, if you don't mind, you can do both.

**CA97:** Yeah. Well, mainly I’m going to speak on Merced County. I have been working here now for 16-17 years, although I don't live here, obviously, I spend quite a bit of time here, and I’ll kind of ditto what was shared earlier. I think it’s a great community in regards to very family oriented. I think in the years I’ve worked here, lots of friendly people, and you kind of look at wherever one lives, we also are looking at the social determinants of health, so I think the economy is growing here. They have the newest university at UC Merced that's here in this county. I think that is great for this community. It’s bringing a lot of new businesses, it’s bringing in a lot of people moving into the area, so you’re starting to see the economy continuing to improve. I think the other part too, although this week is going to be extremely hot, I think the climate, the weather here that allows for people to do a lot of outdoor activities. Whenever I travel to the east coast, they deal with often with such climate conditions of cold, snow, and you don't really get that here, and I think where we also live here, the proximity to the mountains, which is Yosemite, or the proximity to the ocean, over to Monterey or so forth, it’s all about two hours away, so we’re in the center, we’re centralized, and I think it’s a great place for people to live in. I think quality of life is really important for people when they determine where they want to live, so I think the quality of life, although we do, as many communities, have issues with crime, and there are crimes, but unfortunately they tend to be in certain pockets, and I don't think I could say it is affecting all. I think that fortunately, it will be in certain pockets, but you do see crime in certain areas, and there are some kind of gang activities that occur in this community, but I think that law enforcement is making every effort to mitigate that and really try to make this into a safe community, so I think it’s really important that we look at the parts that really make this into a good community to live in. Hopefully, that kind of helps, what I shared.

**INTERVIEWER:** Thank you, thank you for sharing, and as you noticed, may notice, your name has changed, so when I want to call you, I’ll call your participant ID number. Thank you, participant CA97. Participant CA101, is there anything you’d like to add?

**CA101:** Yes. Good morning. Thank you for inviting me, first of all, to be part of this focus group. So, I moved to Merced 22 years ago, with the intent of moving over to a bigger city to the Bay area, but I truly fell in love with the people that live in our community. I was introduced to the world of nonprofit, and I never knew that we could get paid for helping other people. We came from a family of helping other people, growing up in Los Palos, and so I really fell in love with the different organizations that serve and help our community. The other thing about Merced, it’s still small enough where everybody knows everybody and we’re very well connected. Traffic to us is like 14 cars, and that's a lot of traffic, so we can get from one side of the city to the other side of the city in like five minutes, but it still provides us with everything that we need, the basic necessities to thrive in our community. Like participant 97 said, there are some challenges, of course, as we’re growing with the university, but change is good and we’re just going to keep on thriving.

**INTERVIEWER:** Thank you, thank you, and the last point you mentioned is a good segue into the next question, which is what are some things that are not so good about the living in your city?

**CA101:** Well, I’ll jump on that. When we first moved here, the cost of living was one of the perks of living in Merced County. You could rent an apartment for $750, $800, but now today that same apartment is like $1,600 to $1,800, so the cost of living is a huge issue for a lot of community residents, especially low-income community residents. We are a community of agriculture, so a lot of the jobs here are agricultural, construction now, and then the heat is also a huge challenge to us. The drought is becoming a more serious issue in our community. Obviously, we can’t grow food if we don't have water. We’re anticipated to hit 113 degrees some time next week, so the heat is always a challenge during the summer. Then, unfortunately, we just don't have the jobs that truly support someone making three times the amount of rent that they’re asking us to make in the Central Valley, and so that's causing folks to have two families living in an apartment. Folks are leaving our community because they just can’t afford to live here anymore. Then the other challenge too is just the lack of access to medical care, especially the specialists that we need in our community. We still have to travel out of town to receive special care for those.

**INTERVIEWER:** Thank you, participant CA101. Participant CA97, is there anything you would like to add? Oh, participant CA129, I see your hand is up, please, go ahead.

**CA129:** I wanted to expand on the healthcare services. Previously, I was in education, and the biggest challenge we have in schools is mental health services, various tiers of mental health services in our community. We just don't have the specialist specialties. They are in Fresno, they’ll drive up, but they’re hard to get, we don't have enough of them. We don't have enough of even primary care physicians, those that we have are retiring and not taking on new patients, so we just really need a lot more support for healthcare, all levels, specialty, mental health, and just general healthcare in our community.

**INTERVIEWER:** Thank you very much for sharing, participant CA129.

**CA97:** I would just add, because I do live in a larger city, I think when I come here, it is one of the things that I identify is not the same level of resources, whether it in healthcare, whether it is from other government type agencies, to be able to access certain types of programs that we need, they are not always going to be available in smaller communities. I think they have done a fair job when it comes to transportation, but it’s something that obviously they need to continue to work on. The city or county plan needs to continue to work on how they can improve transportation. We are also starting to see, at least from my experience coming here, I’m starting to see kind of an ongoing growth within the homeless population, and I know that affects many communities, so we have to continue to address the homelessness population here and what can be done to be able to hopefully give them some type of assistance, but that is growing. I cannot say enough of what was said by both of the other participants about mental health, all medical services, but mental health I think is one of the largest ones,

**[00:10:00]**

however I will say that this county has done I think some really steps of getting funding that's been brought into this county to be able to bring in more healthcare providers to address the health issues, however it still continues to need to be addressed. I think the other part too – again, I don't live here, but from what I can see – is the opportunities to socialize, whether it is having enough parks for children and families, the activities such as sports, youth sports I believe that they can continue to develop, restaurants, places where people can socialize that brings in the community. They do have a movie theatre, but I think that they can continue to develop, and hopefully in the near future, as they move towards where the newest campus of UC Merced, I imagine there will be some large developers bringing in opportunities for people to be able to go out and socialize, whether it’s new restaurants, movie theatres, shopping malls. The shopping mall that they have here that I’ve been to is very limited with access to certain stores, that you don't see what you see in the larger cities where I live at. Obviously, there’s so much more that's available when it comes to shopping and being able to come out and go out and get your needs met, I think that's something that they continue to work on, but it is a smaller community, so it does take time for the continued growth that will occur here over the next few years.

**INTERVIEWER:** Thank you, thank you. For the sake of time, I’m going to move to the next question. I see participant CA129, you wanted to add something real quick?

**CA129:** Nope, sorry, I didn't take my hand down. Let me do that.

**INTERVIEWER:** Oh, okay, no problem. Thank you very much for this rich contribution so far. I’m learning a lot about your community or the community you work in. Now I would like you to think about how the community responded to COVID-19, and thinking now about your area’s local response to COVID, were you satisfied with the response to COVID, and if you were, why were you satisfied, and if you were not, why were you not satisfied?

**CA97:** It may appear that I’ll be the first one. I may be the first one that takes it. Well, with COVID, it was a challenge throughout this country, so most of it was around information and communication, so I believe my frustrations that I experienced was the lack of resources that we were able to receive initially when we learned about COVID and it started affecting the community. We were big advocates for this county to be able to get vaccines or testing, supplies, and it seemed to be quite challenging. It took some time for us to have, I believe, an ongoing predictable flow of the vaccine coming into this county. We were quite challenged. I will give some credit to the county and to the leaders of this county, as they really worked together and they formed coalitions. Phone calls had a cadence that was every week, it seemed like, there was a call that was established, so we were able to share information and keep everybody informed and updated, so I think that was great. You have an individual that’s on this call, which is participant 101, she was amazing in this community to assemble a group that was very active in this community to be involved around vaccination, testing, and educating the community, but we were limited to resources outside of Merced County, came out and said, well, let’s come out and help Merced County. It seemed like many resources were going to the larger counties, but this county did not receive many resources that came from the California Department of Public Health, that came from others, whether it was federal agencies. We were very limited and had a lack of supplies, so we have had to become very resourceful, and I think I could say I’m very proud of the community here, that we did become resourceful to work together and there was a lot of community collaboration during that time, so we collaborated, many of us, as partners, and I think that says a lot and it speaks so much for this community, that we worked together, but I think lacking the resources that came in from the state level was very frustrating, at least in my experience that I had.

**INTERVIEWER:** Thank you, thank you very much, participant CA97.

**CA101:** I’ll jump on participant CA’s comment,

**[00:15:00]**

so access was nonexistent, I guess I’d just say that it was nonexistent. When the pandemic first hit, we did help organize with other organizations, vaccine clinics, and we had 900 people, 1,000 people lined up out the door, but unfortunately, a lot of our community residents, like I mentioned, are farm workers, construction workers, where they didn't have the liberty to take a whole day off to go stand in line and get vaccinated, right, and then they didn't have time to take off. If they took a day off, that meant that there was no pay for that entire day, and hopefully they didn't lose their job because they took a day off to go stand in line to get vaccinated. Then testing was a huge issue in our community. We didn't have access to rapid tests until almost a year-and-three-months later, I mean literally. I have the privilege now of just going next door and picking these up and swabbing whenever we need to, but we didn't have access to rapid testing, and people had to pay over $100 to get rapid tested at like local pharmacies and the small rural unincorporated communities, so access to preventative services was a huge issue in our community. Then as always, language access is a big issue in our community. If we don't speak the English language, if we only speak for example Spanish, navigating a lot through the system in our community is very difficult, and so that kind of prevented a lot of families to actually go and try and access services for themselves and for their families, but I do have to say that once rapid testing was available to us, our schools did an amazing job of providing access to testing for kids and their families, and they still continue to do that to this day, and so that was really just a great example of how schools are so important in our community, because they really played a huge part in making sure that kids were being tested regularly once we had access to testing. My personal story, if it wasn’t for the agency of participant CA97, when I was there volunteering, I literally got the leftovers of a vaccine dose because that's how limited we were, so I hung out until there was a vial that had enough to give me my first vaccine. I can speak English and I can speak Spanish and I know how to navigate, so just not being able to speak the language and navigate the systems, I can only imagine how difficult it was for a lot of people to get the vaccine, so as soon as we could organize, we started going out to places where we knew that people were that didn't speak Spanish, that didn't have documents, that were intimidated by any government group, and so we started vaccinating out in the community.

**INTERVIEWER:** Thank you, participant CA101. Go ahead, participant CA129.

**CA129:** I’m going to share my experience through the education lens. I worked for the County Office of Education, and the County Superintendent took the lead for all the school districts. We had weekly phone calls with Public Health, and all the superintendents participated in those phone calls, and their related staff, and that was key because we all experienced that about 50% of the parents and 50% of the staff believed there was an issue, and 50% didn't, and information on a regular basis was critical to keep everybody informed, to help those naysayers understand why kids were being sent home, why we were doing kids on Zoom, and then when we were bringing kids back, help those families who were fearful and worried about bringing kids back, just constantly giving them information about the status of the disease and what schools were doing. We purchased a lot of masks, desk dividers. We took furniture out of the room to make sure we could get 6 feet apart from kids. We did a lot of things to help mitigate the concerns. We got pushback from those that didn't believe,

**[00:20:00]**

we’d get nasty emails, they’d go visit us, they’d call us, but we just held the course. We said, “These are the state laws, these are the health guidance, this is what we’ve got to do, we understand your opinion, but this is what we have to do.” Things finally did settle down, but it was a good year, year-and-a-half before more of the people were comfortable with what was happening. Still a lot of controversy about the vaccines, we still had a third of our staff that wouldn't get vaccinated, so were testing them on a regular basis, and that caused rumbling. Eventually, more did get vaccinated, so that number has dropped, so we survived, and I think we did the best we could with education for kids. It’s hard to teach kids with significant special needs on Zoom, and so there’s opportunities to provide extra services now to try to make up for the lost instructional time.

**INTERVIEWER:** Thank you, thank you, for sharing that. There’s a lot of issues that I guess occurred in so many communities, and it sounds like there was a lot of collaboration between organizations in the beginning to do the best that could be done with the limited resources that were available in the community. There was also the point about testing and how expensive it was in the beginning, and this is definitely relevant to this project and the questions. The next question is related to when you were contacted for this project. What did you think about that initial communication, based on how you were contacted? Can you recall any immediate thoughts, positive, negative, or neutral, given the context of what you just shared?

**CA97:** I guess I’ll start again. When we were contacted, knowing that we were very limited on resources, and as participant 101 said earlier, the cost behind it, I think the biggest challenge that I could say, being in a health center, was not only we addressed issues about vaccine, but when it came to testing, the long lines that we were dealing with, because the initial PCR test, when we were inundated with PCR tests, I think we had two main laboratories in this country, which is going to be Labcorp and Quest, they were overwhelmed by the amount of tests that were being done, so a PCR test, which was initially maybe 4 or 5 days, went all the way up to 10 days plus to get a result back, so not being able to get a result back for 10 days, how are you able to address the quarantine issues and to make sure that that individual, when they’re going home, they understand if they’re positive or negative, so now they’re out there socializing. We would talk to patients, do a PCR test, they would make a comment, they’re going to go directly right to the local market here called Food 4 Less and they’re going to go to the store, when we’re telling them to quarantine. So the length of time that it took to get a result was really difficult in the healthcare community, when we were communicated about having the opportunity to have some antigen tests and be able to get a rapid test available for patients, that to us was I would say it was a gamechanger, because it’s significant, although, obviously, there’s been concerns with the accuracy of the test, and also educating patients how the test would work. Not everybody understands it. If it’s someone in healthcare, yes, we do it every day, but the average person, local community member may not fully understand how the antigen test works, so although as I was very excited and pleased to know that there will be more resources available to address the issues going on around COVID, I was concerned about the lack of education that was provided, and it required, again, internal local community resources to educate the community, but it was so important, primarily because of what I’ve stated. It was taking us 10 days to get a PCR test, and it was so frustrating, so the antigen test was a gamechanger for us, at least to let people know, get that in their hands, make sure that they get tested before they went out into the public with family members, and they went to a social event, they could at least test themselves and know whether they were positive or negative, so that was really important for us to have.

**INTERVIEWER:** Thank you, thank you, and who would like to go next? Participant CA101, I see your hand is up. What did you think about the communication about the project specifically?

**[00:25:00]**

**CA101:** Yeah, so we were very excited, just to know that we were going to have an abundant supply of rapid testing, right. The only concern that we had is the self-reporting piece, because that was going to be a challenge for a lot of our community residents. The other challenge that we had, because I was actually housed at the organization that ran the program locally, so I got to sere firsthand a lot of the challenges that we had to face in storing the supply, because now that we had all of the supply, we didn't have warehouses large enough to store them, and so we had to literally travel 10 miles up the road to go pick up orders every time that we needed them, but when we would do our little popup events out in the community, the testing would just fly. We would just pop up at a corner where we knew that a lot of traffic was driving by, and people would just flip a U-turn and drive by and pick up their testing kit. It was great to have. Unfortunately, it was late in the pandemic, but I know that we were able to help a lot of people, because we were handing them out right before the holidays, right before Thanksgiving and Christmas, and so I know that we were able to help a lot of people get tested and make sure that everybody was safe before they started to gather, and then, of course, just the timeline of the grants. Obviously, 90 days or 120 days wasn’t long enough. Ideally, we would have loved to continue to have access to supplies, but we have another resource now and we continue to receive rapid testing and we continue to distribute them to folks most in need.

**INTERVIEWER:** Thank you, thank you.

**CA129:** Schools respectively were very appreciative of the supply, and we were sending them home with kids, families, and they were extremely appreciative, because they were expensive, hard to find, and so it was a welcome resource at the strategic time that we needed it, so thank you.

**INTERVIEWER:** Thank you, so overall, what were your thoughts about the communication approach when you first were contacted about the project? It sounds like you were all appreciative of the resources the project provided, and that's very good, and I guess for the purpose of this question, we are interested in knowing what were your thoughts about the communication or the approach strategies that were used to engage you into the project? Was it positive? Were you hesitant? I mean of course, the resources I guess were --

**CA129:** My communication was positive. We didn't have any concerns.

**INTERVIEWER:** Okay, thank you, thank you, and the other two participants’ communication, was how for you?

**CA101:** I don't know if it – I mean it was just, you know, “We have access to testing, do you want to participate in our program to help distribute them within this timeline,” and so of course, our response was “yes, absolutely,” but there wasn’t a lot of communication, I have to say that, and I was actually in the building, so there wasn’t like a lot of communication back and forth from the fiscal agency, which is good, because that means that they trusted us, right, that they trusted to do what we were tasked to do, which was distribute the supplies. Then the turnaround time, that was a little challenging, because every time we had to restock, it did take us like three to four days to get restocked, again, just because of the warehouse issue, but I guess overall, it was a positive experience.

**INTERVIEWER:** Thank you, thank you.

**CA97:** I would say that the communication, it was brief, but I think we were already in the mindset of wanting to do the antigen test, so although it was brief, it was already something that we were already trying to access. It was just great to know that this came to Merced County, and the partnership that was established for us, it was very welcome and it was well-received that this was going to become available and for us to provide to the community, so it was all very positive.

**INTERVIEWER:** Thank you. Now how, if at all, did the process or idea of working with a community group

**[00:30:00]**

that brought together several community leaders influence your decision to participate in any way, if at all? I can repeat the question.

**CA129:** This is Susan or 129, like I said before, this community is extremely collaborative, more so at the beginning and more so as it progressed, so we didn't hesitate. We were happy to partner with the folks that were involved in the project, because we all know each other, believe each other, trust each other, and talk to each other about any bumps in the road or challenges, so it didn't stop us at all. We were pleased to do it.

**INTERVIEWER:** Wonderful, wonderful. Now how, if at all, was this project responsive to the needs in your county related to COVID-19? – And I’m only ask these questions because we have them, but I also know you also responded and included some topics related to the answers for this question. If there’s anything else you would like to add, feel free, and if not, I’m happy to go to the next question.

**CA101:** Can you repeat the question, please?

**INTERVIEWER:** This question was how was the project responsive to the needs in your community related to COVID-19?

**CA101:** Well, obviously, we didn't have the supplies at the moment, so it fulfilled a huge, huge gap of just having resources to distribute, where people didn't have to pay for rapid tests, and then just knowing that you have tests at home and you can do it in the privacy of your own home. You’re not risking having to go out and expose other people, if you were positive, that was a gamechanger to us. The only negative I would have to say, again, is just that it was just such a short time span of supply of testing, because like I said, to this day we could have still kept distributing them.

**INTERVIEWER:** I understand, thank you. What were some of the challenges, I guess the 90-day time span could potentially be one of them, or barriers that you experienced during the program, during your participation in this project?

**CA101:** Capacity is always a challenge, right. We are a very small nonprofit organization, at that time, I think we only had two people, but our promotoras, which is a network of community health workers, stepped up to the plate, like every time we would be popped up somewhere, I would have the support of the ladies – six, seven, eight at a time – which I couldn't have done it if it wasn’t for the group of promotoras that we have, and they did it because they knew that a lot of other families needed to have access to testing, but capacity-wise, it would have been nice to be able to provide like stipends for our promotoras, continued support, obviously, for a longer period of time, because we only have like one other staff person, so obviously we can only be at one place at one time per day, and so we would have loved to have more people and more popup tents, especially in like the unincorporated communities of our city, of our county, where people don't have access to these kind of resources.

**INTERVIEWER:** Thank you. Anything else?

**CA97:** Yeah, I can. Well, so I can make sure that I don't say the same thing, can you repeat the question?

**INTERVIEWER:** Yes, the question is what are some of the challenges or barriers that you experienced during your time of participation in the project?

**CA97:** I think the main barriers was just where it was being distributed, again, it was not as convenient. During this time, we were dealing with a lot of staffing challenges, so just trying to get resources to get supplies, and I understand it was a [PH] 90-day, it was limited resources, probably on their end also, but it wasn’t something that was being mailed to us where it would have UPS or someone that would drop off. We’d have to go and pick it up, so that was part of it, the timeframe in it, and probably the quantity. Because I do work at a medical center, we provide a test

**[00:35:00]**

and a vaccine seven days a week, so because of that, we were having huge lines of people coming in for testing and vaccination, so we were making every effort to get those out, so we were giving out a substantial amount of tests on a daily basis as people would come in, so we definitely could have utilized more supplies, but we also knew that there was an allocation that had to be distributed amongst many organizations throughout the county, so the supply I knew was limited, was part of it, and I think also the time of 90 days was also a challenge.

**INTERVIEWER:** Thank you, thank you for sharing that. 129, nothing to add?

**CA129:** I don't have anything to add. They covered it.

**INTERVIEWER:** Okay, thank you. Next question is what, if anything, helped you accomplish the task that you were given as part of this project?

**CA129:** I was able to use my school nursing staff and health aids to organize, distribute, deliver, inform parents, answer questions for parents about the test and what to expect and what to do, so I had resources that I could lean on to provide the support to make the process go smoothly.

**INTERVIEWER:** Thank you.

**CA101:** Repeat the question one more time, please?

**INTERVIEWER:** Yes. What, if anything, helped you accomplish the task that you were given for the project?

**CA101:** Yeah, like I mentioned earlier, our network of promotoras, which are our community health workers throughout the county of Merced, truly stepped up to the plate, and they still continue to help support us at the vaccine clinics that we do once a month, but they went over and beyond, and knowing that it was all volunteering for them just speaks volumes, but again, if it wasn’t for them and the support of that network, we couldn't have been able to impact as many people as we did.

**INTERVIEWER:** Thank you.

**CA97:** Just to add briefly, I think what helped us accomplish that is very similar to what participant 101 is saying, is that we had already built-in collaboration and partnerships, so when entities learned that this had become available, not all were participating, so we were able to have them refer patients over to us or we collaborated with them to be able to know that we could distribute the tests out, so I just go back to just knowing that there was a community that was ready, and I think it was ongoing collaboration and participation that we had with a lot of community members, so it really helped us to get the word out and get the tests into people’s hands. That was the most important part, because again, what we were seeing at our health centers, people were not coming in to the health centers, they were not coming in, so we were able to work with partners to get those out into their homes and deliver them directly to communities.

**INTERVIEWER:** Thank you. What do you think the overall goal of the project was?

**CA129:** For me, it was to get as many tests in the hands of families as we possibly could, as quickly as we could, in that short period of time.

**INTERVIEWER:** Thank you, and in your opinion, do you think it was successful?

**CA129:** Yes. Like the other participant had said, too short a window, would have loved to have a longer window to provide more resources, but we appreciated what we did get.

**INTERVIEWER:** Thank you. Um, participant 97?

**CA97:**  What was the question?

**INTERVIEWER:** Oh, the question was what do you think the goal of the project was, the overall goal of the project?

**CA97:** Oh, I look at it, the overall project goal was to bring in a really much-needed resource, which is a rapid test, get people to learn

**[00:40:00]**

and identify if they are positive so we could help mitigate the spread of the virus, and I think that's been the concern we had early on. We didn't have the tools, the PCRs were taking so long, and antigen tests were a resource, a tool that we had to help people learn if they were positive, and if they were positive, they could quarantine, they could isolate themselves, they could let family members know, get more people tested, so I think that goal of the project was important, but as what's been stated, that short period of time, and you know, this was not a three-month project, we’ve been at this for two years now. so even at the present time, we're limited to tests and to be able to get antigen tests now. so now we have to direct people to the market, where they're going to have to spend $25 to $35, depending on what the cost of tests are, so that's been an ongoing issue, but it did help us to assist patients to be able to get tested, as what was stated earlier, in a safe environment at home, where they could be tested, and if they had symptoms, they weren't going out to a location where there may be long lines to get tested and limited resources to begin with already in the community. That's what we have identified, there was such limited resources to be able to test. Our own staff were getting sick and they were getting out, so we were short with staff, so it was great to have this testing that we could send to people to do it at home.

**INTERVIEWER:** Thank you, thank you, and do you think the project was successful in achieving that goal?

**CA97:** Yeah, again, I think the project was successful in those 90 days, it was a great resource to have, it was a great tool to share with the community, but I think as what has been stated, this has been a two-year time that we’ve been in, so it’s great that we did have this, but as what occurred for our organization, once we started seeing that the resources were limited where this project ended, then the county was having some challenges with accessing tests and so forth, then we started having some challenges, which we continue now to be able to get the tests into people’s hands. We have to, unfortunately, direct them to either have the test done here in our facility where we do it for free for them, but if not, then they have to go to the store and pay out of their pocket, and that could be substantial for some families to pay that out of their pocket.

**INTERVIEWER:** Thank you. Participant 101, would you like me to repeat the question?

**CA101:** Please, yes.

**INTERVIEWER:** The question was what do you think was the overall goal of the project?

**CA101:** Well, I think the overall goal of the project was access, right, and ensuring that folks had access to testing. I think what we achieved once again is a perfect demonstration of how all of our nonprofit organizations, our school systems, out community partners, worked together to ensure that families had access to testing. I mean when I was watching people in action and I would see the photos that would come up, it was very innovative and inspiring just to see what organizations were doing to make sure that families had access to testing, I mean they were putting them in like the food distribution boxes and just making sure that those especially hardest-to-reach populations had access to testing, so it proved, once again, how our community truly mobilizes to help our residents here in Merced County.

**INTERVIEWER:** Thank you, thank you. Next question is what do you think was the biggest factor driving county residents to pick up the COVID-19 testing kit? And I know you have mentioned at different times the need for those test kits in our community and the expense for others who had to pay out of pocket before the project was implemented, but if you have any additional ideas you think you could share related to what was the driving factor for the residents picking up those kits quickly, as you mentioned.

**CA101:** If I can start with that question, I think people needed to know, I mean if you had a runny nose, everyone was panicking, right, because we still deal with allergies in the Central Valley, and we didn't know if it was allergies,

**[00:45:00]**

if it was a cold, or if it was COVID, so just having this tool right at hand and being able just to know, to protect yourself, to protect your family, to protect others, to protect your community was priceless, and to have that on hand where we didn't have to drive out to a clinic, wait in line. I mean sometimes these waits for rapid testing were three hours at a time, so that was a very powerful piece, just having the tool where we can know, and the peace of mind, knowing that I have a test kit at home, that's priceless.

**INTERVIEWER:** Thank you, thank you. Go ahead, 129.

**CA129:** From the perspective, it was the peace of mind, and parents wanted to keep their kids in school, because we did have a lot of concern about every sniffle, every runny nose, because kids get those things, and so we were not wanting to keep kids out but still not wanting to stick kids at school, so they were highly motivated to get quick results so they could get their kids in school, or if they needed to keep them home, they could find out quickly before we would expose the rest of the classroom and the staff, because we didn't have to close classrooms because it was spreading to other kids, and unfortunately, we had to close classrooms down for 10 days or more at a time.

**INTERVIEWER:** Thank you. Participant 97?

**CA97:** The only thing I can really add to it, part of that is the fact that again, PCR tests, what 101 said, is the long lines that were still there, and I also think that for a period of time, we started getting a little bit of a stigma for some folks wanting to come out and they were being tested, because we now were really emphasizing the guidelines. We were being educated on those from the CDC and CDPH, so now I think it was part of that, the fact that it could be confidential and done at their home, and they could get a fast result, I think that was important, and I know what you stated earlier, which has already been addressed, is the cost factor, but I think just the fact that it provided a way for it to be done in a confidential manner and it allowed them to do it from home. The convenience I think was a really important part. We came across so many people that just were so appreciative that they were handed out, so it was a really important project.

**INTERVIEWER:** Thank you, thank you. The next question is kind of related to the first, in a way, but what do you think was the biggest reason that county residents were interested in knowing their COVID-19 status, if there are any additional reasons that we have not shared? 129, go ahead.

**CA129:** I was just going to share that staff members and parents were concerned about other family members that had health challenges, so they were wanting to make sure they protect their other family members by monitoring closely their own health, because people were dying and so they were frightened, so it was important to them to have that access and resource, not just for them but for the other ones that they loved in their family.

**INTERVIEWER:** Thank you.

**CA97:** We came across a lot of people that were very anxious, so there was a high level of anxiety, what was just stated. You were hearing people that were going into the hospital and in patient care that were in intensive care for an extended period of time, so there was just a lot of anxiety, and still things are misunderstood by many people. As we continue now even two years right now into it, there are still some misunderstandings with it, a lot of mixed communication was sent out, but I do think the anxiety level that people had I think is something that we saw with many people in the community.

**CA101:** Yeah, just not knowing

**[00:50:00]**

caused a lot of anxiety and a lot of stress still do this day, right, but like I said before, just having access to tests, especially in the privacy or your own home, and it’s quick, and it’s readily available, and I can test my child before they go to school, I mean it’s a great tool to have. Then the other thing that we would hear from people too, when they would come to pick up testing, would be, “I got exposed to somebody who just tested positive, so I need to know,” so when we had folks testing the one day, and then we would tell them to test again three days later, and to have that available, I truly believe that we saved people’s lives providing these tests. We could have saved so many if we had them way before but we’ll take it now.

**INTERVIEWER:** Thank you, thank you. The next question is what measures, if any, were taken to ensure the needs of all county residents were equally considered throughout the program? I can repeat it. What measures, if any, were taken to make sure that the county residents’ needs were being considered equally throughout the distribution of the kits?

**CA101:** If I can start with that question, I think this is why the selected agency reached out to a lot of the CBOs, right, out in the community, and a lot of the schools, because we know where the challenges in our communities are, like we know where to go if we had to go like to Winton, an unincorporated community, and we know who to partner with if we had to go to Granada, because that's what we do, and so that's why they partnered with a lot of us to ensure that we did have the resources, we did have the capacity. The language capacity to ensure that families are able to communicate with us, obviously, so they partnered with organizations who speak Spanish, they partnered with organizations who speak Hmong, they partnered with organizations who speak Punjabi, [PH] Miseko, so they did a really good job of ensuring that we did capture all of the organizations that have the capacity to serve our community.

**INTERVIEWER:** Thank you, thank you.

**CA129:** And in the schools, we at the County Office serve kids county-wide, so made sure we distributed the test for every child in the county, and we sent home an automated phone call in English and Spanish, explaining why they were getting it, what they were getting, and leaving a phone number for them to call if they had additional questions or concerns.

**INTERVIEWER:** Thank you.

**CA97:** Can you briefly repeat that question?

**INTERVIEWER:** Yes, I can. What measures, if any, were taken to ensure the needs of all the county residents were equally considered throughout the project?

**CA97:** Oh, and again, you’re talking about by the project leads, what measures they took?

**INTERVIEWER:** Yes, in your organization and the project overall, as you were involved throughout the project, what measures did you take or your organization take to make sure that all the residents were equally considered when it came to I guess distribution of the kits?

**CA97:** I think most of that was also communication that we were receiving from the committees that we participated on, and in that we were getting lots of information that was being updated to us, where you had the highest transmission rates, positive tests. We were getting lots of data at that point, so I knew that there were certain areas that we needed to target. We also knew that there were certain areas of our communities that have less resources, less access, less transportation, so it was important for us to reach out to those individuals. As I had mentioned earlier, 101, many of them were very active in areas where patients that often have limited access or insurance programs, so you go directly to them where they’re at,

**[00:55:00]**

and this may be farmer markets where they’re going to come together, so it’s taking those to them, so this way we could make sure that there was equal and fair access to everybody, I think in regards to the entities, the health organizations like ours, we were trying to send out mass information out to people, whether it through our website, whether it through text messages out to people to let them know that there were these tests available, and trying to make sure we were able to distribute it where it definitely was needed. Like every community you go to, there are some individuals that understand how to navigate the healthcare system and there are people that don't, so how do you reach those people and how do you target those individuals, and that's really what we were trying to focus on.

**INTERVIEWER:** Thank you, thank you. Now thinking about how exposures to health and COVID-19 information in community, did this project help you identify any credible sources of information or additional credible sources of information, if any?

**CA97:** Did it help us? Could you repeat that again?

**INTERVIEWER:** Yes, I said thinking about how exposures to health and COVID-19 information in your community are identified, did this project help identify additional information?

**CA97:** I think the main thing is it just brought a lot of attention to the opportunity to do a rapid test, and I think for all the entities, the nonprofits that were involved, we were communicating and educating the community about this, so it definitely did bring more attention to COVID-19 and the circumstances that we were living in, so I just think it did provide us an opportunity to educate people, and why a rapid antigen test was so critical for us to be able to get that into their hands, but beyond that, I think to my experience and my work that I do, I could not say it was more than that. I think that the educational piece, when it came around COVID, has been a challenge throughout this whole time, because there’s been so many mixed messages that often the community had been very confused by it, so even when it came to rapid tests, you have some people that are on the other side that didn't want to accept COVID-19, refused the vaccine, refused the test, they felt there was no point in doing it in some cases. I’ve had many people tell me, they used the word that it’s a “scamdemic” versus “pandemic,” I’ve heard that often from people, that you’re doing a “scamdemic,” so a lot of mixed messages often.

**INTERVIEWER:** Thank you, thank you. Next question, unless the other participants have much to add for this one. How did your tasks for this project, compare to your normal activities with the organization that you are representing, change? Yeah, your tasks for this project compared to your normal activities, how would you compare them, if there are any differences?

**CA129:** Well, for me in education, I had not previously sent out medical testing kits like that, so we had to stop and think about how are we going to communicate it, how am I going to get it to the sites, because we had to make sure we’re storing it correctly, so it was a process, a system. It was new, a new process, a new system. So I sat down with my key leaders and we discussed how are we going to manage it, how are we going to make it happen, and took the time to make that happen.

**INTERVIEWER:** Thank you, thank you. How about participant 101?

**CA101:** Yeah, we are not an organization of direct service, like our heart for work is to train resident leaders to understand policy systems and environmental changes, and to create healthier environments, and so we are not a direct service agency, we haven't ever been a direct service agency. However, with all the inequities that COVID-19 brought out, it is why we chose to respond to the call, because when we were on lockdown, like we felt it was hopeless, right, like we’re supposed to be helping now instead of being locked in our houses. We locked ourselves in for like 60 days, and we were like, this is it, we’ve got to go out and we have to be part of the solution, because it is not helping anybody just being locked in our homes all nice and safe when people are still being exposed, where people still have to go to work, where people are not testing, because if they do test and they’re positive, they have to then stay home for 10 days and lose 10 days of work, so we really had to respond to the call to be part of the solution, and that included vaccine clinics, that included education, that included organizing education Zoom meetings to community residents, to our promotoras, to understand what was happening. We were doing those every other week for the first seven months of the pandemic. And then of course, again, just having access to COVID test kits was just another successful way of ensuring that people were staying safe in our community.

**INTERVIEWER:** Thank you, thank you, participant 101. Anything you’d like to add, anyone else?

**CA129:** I am ready to go back to normal though, I want to say that.

**INTERVIEWER:** I understand, I understand, I completely understand. The last question on this topic is is there anything else you would like to share related to this program? No? Okay, no problem. I mean I think you mentioned if you had more kits, more time, and so forth and so on, so nothing else related to that. Then are there any challenges – well, this could be relevant, right – are there any challenges you experienced specifically because of race, ethnicity, age, gender, sexual identity, linguistics, or geographical location in completing the task for this project?

**CA101:** No, but I did want to give one response to your previous question of adding something else, I don't think we’ve gotten results of the actual surveys that were attached to the test kits that we were asking people to participate in, so it would be nice just to know what the results of all of the surveys were, and if it could be broken down to our county or to our region, because I don't ever recall seeing anything in regards to the surveys that were attached to the test kits.

**INTERVIEWER:** Thank you, I’ll make sure to share that with the team, and I’m sure they will also probably share the findings eventually. I know some of the data that was collected was not part of the project itself, but more of the CDC data being collected, but thank you for bringing that up. I will also share it. We have now a few more questions related to another – I mean the same project but kind of switching gears a bit – but still related to what we’ve discussed, so this one is what encouraged you to agree to work on kit distribution, and I think we’ve all mentioned some responses related to that, so if there’s nothing else to add, I can just continue to move on to the next questions to make sure we ask all the questions, even if you’ve already answered them. Anything else to add to this one? No, okay. If you had to pinpoint one thing that had the biggest influence on your signing up for the project with your community or organization, what would you say influenced you to sign up?

**CA129:** Having test kits for every child that I served. I wouldn’t have wanted to participate if I couldn't have served all of my kids, so I was appreciative of that.

**INTERVIEWER:** Thank you, thank you. Anything else from the other participants? No, okay. The next one is how, if at all, was the

**[01:05:00]**

project’s team responsive to your specific community’s needs? It nothing else, I will move on.

**CA101:** Repeat the question one more time, please?

**INTERVIEWER:** How, if at all, was the project’s team responsive to your needs, the specific community’s needs?

**CA101:** The project’s team?

**INTERVIEWER:** Yes, the You + Me COVID Free project.

**CA101:** I guess just accessing the restocking of the test kits, I mean it would take us at least three days to get restocked on average, but they did what they could, obviously, because it’s capacity too, but overall, I think it was a good experience working with them.

**INTERVIEWER:** Thank you, and how, if at all, has working with the project impacted your confidence in talking about COVID, if any, if it helped in any way? Nothing. Would you participate in a project like this in the future? Why or why not? 0

**CA101:** Absolutely.

**INTERVIEWER:** Because?

**CA101:** Access.

**INTERVIEWER:** Access.

**CA101:** Yeah, we continue – that was somebody coming to pick up some tests, and then the phone rang, because it’s still an everyday thing for us. I know that we tried to reorder some boxes of test kits, and they cut us off, so apparently the Feds are slowing down the funding for access to test kits, so that scares me a little bit, but just knowing that we have access to test kits and that we can distribute them continues to help the community. I don't want to see ourselves back to where we are completely out of test kits, because this pandemic is not over. Until we have the majority of our population vaccinated, and not everybody in our community wants to get vaccinated, and so we have to respect that, but just having them in stock and being able to provide them to communities, you know, it’s still helping people.

**CA97:** Yeah, and I’m going to just add, the access word is important, but now looking at it from the perspective that it allowed us to create some equity when it came to distribution of these tests, because the cost factor for people to spend $25 or higher for a test, it can be challenging, so this project was so important to be able to create some equity when it came to getting tests into the hands of many people here that probably could not afford them, so they were not able to test. Then what was also mentioned is that we started seeing some of the supply becoming more difficult to access, and the healthcare provider where I’m at, we were even being challenged, and if we were challenged being a healthcare provider, and I am in contact with HRSA, which is the federal level or the state level, I have contacts, and even there, there were limited supplies, so this project was a great timing for us to get this in there, and I do think that when you look at the social determinants of health, we were able to get these tests out into the hands of people that probably couldn't afford them.

**INTERVIEWER:** Thank you, thank you, I completely understand.

**CA129:** And absolutely, education would participate again. Again, it’s access and equity, making sure we would have enough tests to distribute to every child in our system that we serve.

**INTERVIEWER:** Thank you, thank you. I have a couple of questions I skipped earlier, just to make sure we had enough time to cover the other questions, and then I’ll just mention them briefly. The first one is how do you think your county’s response for COVID compared to what was happening nationally, based on what you shared earlier?

**CA129:** Well, I can share with you, I had family in Texas, we had a much higher level of focus, concern, collaboration, attention, education to this issue

**[01:10:00]**

than my family members experienced in their home cities in Texas, so I was grateful and appreciative of what we were doing, and was sharing with them the information and the resources and the things we were doing here in our community.

**INTERVIEWER:** Thank you. Sorry, and the last one is where do you typically get your COVID-19 health-related news and updates from?

**CA129:** For education, it would be through the California Department of Education and the Department of Public Health. We are obligated in California to the Department of Public Health. Those are the three entities that guided how we responded and what we did and didn't do, and they continue to provide that information.

**INTERVIEWER:** Thank you, participant 129. 101?

**CA101:** We partner with a variety of folks that continue to help us keep up with up-to-date accurate information, because misinformation was a huge issue in our community when it came to COVID and vaccines and testing, so receiving accurate information was very important to us, so we partnered with UCSF, which is UC San Francisco, we partnered with UC Merced, and then of course our local Department of Public Health, California Department of Public Health. Then we also have like a community county-wide sort of education informational sharing that's organized by our Public Health Department, where a lot of the nonprofit and partners log on, on a monthly basis, and continue to share where we’re vaccinating, anything that's new that's happening in our community, so we continue to keep on talking and sharing resources whenever we can.

**INTERVIEWER:** Thank you, and I’m glad you mentioned the point about misinformation, because one of the questions is what makes a source credible, in your opinion?

**CA129:** For education, we couldn't judge whether it was credible, we had to follow what the mandates and the legal requirements were. We had a lot of people questioning California Department of Public Health or our local Public Health opinion and guidance – we had to follow it, and that's what we would tell everybody, and the same with California Department of Education, so they were our resources, and we had to have confidence in them. We would push and ask and present other opinions that were presented to us, and get them to speak upon it, and then we would try to share that information to our staff and to the public, those that were criticizing the guidance we were getting or claiming that it was not accurate. We were trying to continually vet the other side with those three sources, and then sharing the results of that.

**CA101:** Yeah, so in the Latino community, and you know, it is very important that the information comes from a trusted source, and we were very transparent with our network of promotoras from the very beginning. We at no point did we ever say that we’re the experts around COVID, because we were learning right alongside as they were, because every day things were changing on us, but we would jump on any training opportunity that was available, through Zoom or Facebook or anything that was available to us, because we had to continue learning and we still continue learning, but we were never the experts, but just knowing that they did have someone who speaks their language, someone who has a reputation in the community, that builds a lot of trust with community residents, especially the population that we were working with, which are immigrants, undocumented Latino communities that only speak Spanish, and so we were very privileged to be able to help and still continue to help to this day.

**CA129:** And if I could add one more comment after listening to her is that it was important to have the same message shared in every school district in the county,

**[01:15:01]**

so all the superintendents met regularly after the local Public Health call or in between, to make sure everybody was saying the same things to their staff and families, because there was a lot of misinformation out there, and if the schools weren’t going to be on the same page, it was going to make the situation worse, so they were really, really tightly connected with one another, and if they heard somebody said something slightly different, the county superintendent was calling and saying, “What’s going on? I thought we agreed on this.” So it was critical, because we had kids in elementary schools and in high schools, and if they’re getting different messages, it was just going to implode the whole process, which was already wrought with lots of challenges and different stories and misinformation.

**INTERVIEWER:** Thank you, thank you, participant 129 and 101 and participant 97, I have learned a lot from this discussion. I have never been to Merced, but I imagine what it looks like just from what you shared and hope to visit one day, and this concludes our focus group for today. I really appreciate you all for making the time and sharing your rich experience and your commitment to your community. I think that's definitely one thing I heard over and over from you all, even before COVID-19, that you are all committed to your community, you are committed to the work that you do, and I wish you all the best to continue to fight for the community that you are so passionate about, to get the resources and increase access to the community members. If you have any additional thoughts or opinions you would like to share with us, please you can do so now, or you can also feel free to email us as time goes by, but I’m really, really appreciative of what you have shared, and as someone who also works and cares for my community, I am originally from the Caribbean, but I know communities everywhere matter to us, so thank you.

**CA101:** We also want to thank, I know I want to thank Duke University, because when I heard that Duke University was bringing this program to Merced, I mean that was woah, that's prestigious, so thanks for putting us on the map!

**INTERVIEWER:** Our pleasure, our pleasure.

**CA1129:** Thank you!

**INTERVIEWER:** Thank you for responding to us. Well, Bola do you have anything to share before the participants, all of them leave?

**BOLA:** Oh, no, not really. I just want to say thank you all for your time, thank you for attending today’s session. It was a very insightful discussion, and we will also be sending out like a survey just to know your thoughts about how this focus group went, and then a confirmation of an email where your gift card would be sent to. But thank you so much for your time today.

**CA101:** Thank you.

**INTERVIEWER:** Bye-bye.

**CA129:** Thank you.

**CA101:** Good to see you, participant 129.

**CA129:** Yes, good to see you too. Take care.

**CA101:** Bye.

**CA129:** Bye.
