# Supplementary material for: Exploring barriers and facilitators of implementing an at-home SARS-CoV-2 antigen self-testing intervention: The Rapid Acceleration of Diagnostics—Underserved Populations (RADx-UP) initiatives
Source: PLoS One. 2023 Nov 16;18(11):e0294458. doi: 10.1371/journal.pone.0294458 (PMC10653400; doi:10.1371/journal.pone.0294458)
Supplement: S1 Dataset — (ZIP) [file pone.0294458.s002.zip › CA-Transcript (9.29.22).doc]

**FACILITATOR:** We're just going to kick off with some general questions. We'd just like to know what makes… My understanding is you're all from Merced. Correct? What makes Merced, or I guess, California more generally, a great place to live? What do you like about living there?

**INTERVIEWER:** Well, I can tell you right now the weather. I wouldn't want to be in Florida.

**FEMALE 3:** I think my neighbors.

**FEMALE 4:** I think Merced is a historically significant town that has growing pains, and I appreciate being able to be involved in the solution to the dilemmas that it faces today, but the weather sucks. 115 is not a good thing, according to me.

**INTERVIEWER:** I second that.

**FACILITATOR:** Anyone else? And you all are kind of anticipating the next question, which are some, what are some things that aren't so great about living in Merced?

**INTERVIEWER:** 115 degrees.

**FEMALE 4:** There is an attitude here that Merced sucks, and it doesn't seem to make any difference what happens with positive change. There are still people who are dug in to, they call it Merdead, and there'll be, on the city calendar, there'll be events every single day for children, families, individuals, whatever and people will still say there's nothing to do, and that attitude seems to be intractable. That is a drawback, and by the way, I do my work in Planada, which is a farm worker town, not Merced, and so, when I was distributing tests, it was in Planada.

**INTERVIEWER:** But in Merced County.

**FEMALE 4:** It's Merced County.

**FACILITATOR:** Yeah, okay. Yeah, yeah. Feel free to reflect on, you know, any of those areas that are relevant too.

**INTERVIEWER:** I think, for us, one of the complications is we have a love/hate relationship with UC Merced. We have a brand-new university, which is now a little over ten years old. So, it's not brand-new anymore, but we love the energy and the knowledge and the expertise that comes with it, but we hate the fact that the housing cost has gone up like crazy for our community members, and so, many of our low-income families are displaced, because UC students and UC see professors can pay more for things. It changed, also, the, there's almost an us versus them kind of feeling at times that's a little bit depressing. Well, depressing is a harsh word, but a little bit frustrating at times, because we should be working together, and so, I think that's, yeah. Yeah.

**FEMALE 4:** In Merced, since the university has developed, the needy are more needy than they hey used to be.

**FACILITATOR:** Yeah, interesting. Anyone else have anything else to add in terms of what's maybe kind of challenging or not so great about the community?

**INTERVIEWER:** Amelia, can I ask a backup question for just a second? I know most people on this call, but I do not know who's DA103. Yes. Can we?

**PARTICIPANT 1:** Hello. Hi, I'm [participant name]. I'm the Assistant Director of Case Management Services here in Merced for Central Valley Regional Center. We serve disabled individuals from birth through their lifespan. We actually serve six counties, and we have three offices, but I've been here, myself, 20 years. I'm very blessed to be part of this community. So, we serve over 24,000 individuals with all of our…

**INTERVIEWER:** And you do good work. You do important work.

**PARTICIPANT 1:** Oh my gosh! Thank you. I appreciate that. You made my day. Thank you.

**INTERVIEWER:** Yeah, we definitely need you. I just couldn't put a face with the organization. So, hi.

**PARTICIPANT 1:** Hi, thank you.

**FEMALE 3:** I'd like to say one thing that's on a negative side is the healthcare that's available in this town. It seems like, I myself, go out of town, you know, especially to the San Francisco Bay area or even, you know, Fresno/Modesto for healthcare, because we just, we're just very limited, and especially, I work with the blind and low vision here in Merced, and we're down to like two ophthalmologists or something like that. I mean, it's really gotten bad in this area for that.

**FEMALE 4:** Yes.

**PARTICIPANT 1:** I would have to mirror you, [participant name], in what you're saying, because even not just the individuals we serve, but for our employees, each year, we throttle between two, because they raise it $100 or $200, and so, although we serve six counties, Merced County, for our over 500 employees, is the highest premium for any of the six counties we serve, and for our children's resources here, our families have to drive to Valley Children's Hospital, and it's a big jaunt for individuals who live in Livingston and Blanco and Los Spanos, and they end up asking community members, can you give me a ride, and they charge them $50, and so, transportation, as Margaret and I have been involved in many transportation meetings throughout the years with our local transportation, is a challenge, because the places they need to get to for the medical doesn't have that route, because it's out of county, and so, I'm including transportation and in with that as well.

**FACILITATOR:** Yeah. Yeah, gotcha. That all sounds really complex to navigate, and perhaps, it’s a good segue into our next set of questions, which is around what you all felt like your local area did well in responding to COVID-19, specifically, and then what were some challenges or things that you wish had been done better?

**PARTICIPANT 1:** Can I real quick ask, because I was bumped out for a second there, did you, we were saying difficult things in the county. Were we able to express the positive things in our county?

**FACILITATOR:** Yes, that was the first question. So, if you would like to add anything there, please do.

**PARTICIPANT 1:** Yeah, I apologize. I was trying to get back in. You know, I think that, you know, just because I have the pleasure of working with so many counties, I can say that Merced County, what I love about us is our coordination and our collaboration. I mean, the other counties, we have round tables where we have APS there and CPS and mental health and, you know, alliance and our community partners, you know, from all the hospital, and we triage, but beyond triage, I mean, we'll have like 10-15 people at a table for monthly meetings to all try to see how our agencies could collaborate, and is that something that maybe they're developmentally disabled or mental health or they need, you know, the vision support services. We also do, like, through the educational system, transition council fairs, and I know that, again, I see Margaret, and I don't mean to be missing anyone, if there's somebody else on here I don't recognize, but a huge collaboration between the school systems and, you know, many of our providers that we do big events each year, and I think, because we are small, and we usually know our directors and our leadership, and we're on first-name basis, and there's a real benefit to that, and I've found that to be just enjoyable and getting to know each other and having those continued relationships, and it strengthens our ability and the service delivery model for the different populations we all serve and different age groups.

**FACILITATOR:** Yeah, that's great. Thank you for sharing that. Anything to add before we jump into the COVID specific questions? Yeah. So, what do you all think in terms of how your [INDISCERNIBLE] or the area in which you work responded to COVID-19? What do you think went well?

**FEMALE 4:** Public health really motivated the community and mobilized all of the nonprofits. We had vaccination clinics running right away. We had testing at all of the pharmacies within a few months. My own pharmacy was one of the first to offer testing. We reached out to the farm workers and people who were underserved, very deliberately, and were able to, I think, meet their needs. The little town of, the farm worker town of Planada, where I work, it's 75% vaccinated. The rest of Merced County is less than 50%, and it's because of the outreach that was provided to them.

**FACILITATOR:** Yeah, that's really awesome. What about others? What's your experience been?

**PARTICIPANT 1:** I can personally…

**FEMALE 4:** I think [INDISCERNIBLE], oh, go ahead.

**PARTICIPANT 1:** I can personally speak from the event that we had, you know, where we had the mobile vaccine, you know, to be able to give out the test kits and everything, that that was very positive. I can't remember how many thousand we did. I mean, we definitely had a traffic jam, and I'd have to revisit that for future events of that nature, because I was like thinking that our friendly police and sheriff's depart was kind of like, you can't do this. We had so many people, even after we kind of ran out, then people, you know, there was still some time, and so, we were out there directing them to the next site and the next location. There were multiple locations. I remember even driving downtown in our civic center down there, there were people, you know, out there all the way down the sidewalk, all the way around the block, accessing that, and so, I think, and the fairgrounds and the college, I mean, I just can't remember a site where there really wasn't, I mean, not that I'm trying to be negative, but I think some of the CVS or some of the pharmacies, that became a really difficult way to access things, because, especially because I work with disabled population, that they were very rigid, that they were like, you have to go through the site, the internet site and sign up, and I actually met with the managers there, asked for the manager, and I said, you know, not everyone has that ability. Like, the world seems to think like everyone's got a computer or has this ability to navigate these, and they're like, well, it's easy, and someone could do it. I'm like, no, it's not easy. I did it myself, and it's not that simple to get the times and dates and to do that, and I said, you know, ADA accessibility, I said, to this option, can we not have them come with their workers, and in person, set up a schedule or date, and they completely would not allow us at all, zero, zilch.

**FEMALE 4:** That was true of all of the big box chains, but the independent pharmacies, such as that Joaquin Drug, all took their patients by the hand and made sure they'd got through the process.

**INTERVIEWER:** Wow.

**PARTICIPANT 1:** Yeah. So, I didn't want to be negative on the whole thing, but I was mentioning that the larger ones, that was really unfortunate, because that was a huge barrier, but I wouldn't say all the large ones, because I believe, was it, there was another one, maybe, [INDISCERNIBLE], that the lady, the pharmacist there was just wonderful and took the personal phone call. So, once I realized that, she said, I'll help your families. So, she didn't make them go through the computer system, but it was the larger box ones, and so, they had lots of openings, but we couldn't access them, and they weren't open to the discussion, which was disappointing to myself, personally.

**INTERVIEWER:** Yeah, I just want to echo what has been said so far. I think the good part was that there was plenty of testing available. It was inundated, right, and so, that's really what we wanted to see happening. At the same time, for us in Winton, we did have a hard time getting our people vaccinated, because of the, well, we're not in the negative yet, so I'm going to wait for that later.

**PARTICIPANT 1:** I did the landslide though. Go for it.

**FACILITATOR:** Yeah, you can bundle if you'd like to respond to both questions.

**INTERVIEWER:** I'll wait.

**FEMALE 5:** Well, I think it's positive that the county did reach out to programs like yours that got the things to us to be able to distribute. I guess that's the best way to say it. I know that, you know, family members and things that live in other communities did not have the opportunity to be able to get free COVID test kits like we had here in our county. So, I think that was a real positive thing that we did may have resources available for us to distribute, you know, in our community.

**INTERVIEWER:** And I think, with that, the reality is that, having given it to the smaller non-profits was so powerful, because we are the people on the ground, right? Our community members know us. They know they can trust us. They know that even if they're undocumented, they will not face any issues around that, and so, I think that was a brilliant move, whoever orchestrated that whole thing.

**FEMALE 4:** The other advantage of doing it that way is the volunteers at nonprofits are community members. So, you go down to where Aunt Helen is and get your test, or Joe from down the street. They know who they are, and they feel safe and confident.

**FACILITATOR:** Yeah. Yeah, that sounds really powerful, and anything else to add in terms of good or bad?

**INTERVIEWER:** I just want to give out kudos to Janet, because I think Janet just worked her butt off. So, I have no clue how in the world she orchestrated this whole thing, but, you know, I mean I think we all were like texting and calling and going, okay, now what, now what, and stuff. So, she was just phenomenal in this.

**FACILITATOR:** And maybe you're also anticipating the next set of questions, which is around kind of your perceptions of, when your organization was contacted to participate in this AES COVID test project, and kind of how that interaction looked. So, can you tell us a little bit about what you thought about the initial communication, who reached out to you, and how that looked?

**FEMALE 4:** Honestly, I don't remember it.

**FEMALE 5:** Well, I think it's kind of what Barbara brought up about us networking and pretty close working together. I actually heard it from a couple of different people, because of the connections we make with different people in the community, and so, I think, like Barbara pointed out, that is a kind of a strength in our community, I think, because each of us have so little as far as resources go, we can't duplicate, because there's not enough out there to duplicate. So, we do rely on each other, and I think that's how I found out about it. We're involved with the area agency on aging. We're involved with some of the, you know, the church groups that were contacted and community groups. So, I was hearing it from even a couple of different areas that this project was out there and available and if we were willing to participate.

**INTERVIEWER:** I think it came through United Way, if I remember right, and so—

[OVERLAY]

**INTERVIEWER:** I think it was Janet who just knows everybody in town kind of just connected with all of us during that time.

**FEMALE 4:** Janet has been doing projects, social services type projects, for I think a million years, and in that way, has become acquainted with, well acquainted with the community organizations. Nobody else could have pulled this off the way she did.

**PARTICIPANT 1:** Right, Janet reached out to myself, and her and I had worked together. She used to be with Merced County, the bus. She was an employee of the bus and did the most dynamic, amazing work I've ever seen for our individuals with transportation training and unique routes, that we still don't have to this day, but we did when she was there, and then she was over with the county, with several different departments, and so, she would reach out to me. So, you know, you have that trust with somebody that's really part of your community, side by side. She's not a desk sitter. I mean, we were out in community events, handing out hats, you know, and other projects we worked on side by. She's a doer. She's a worker. She's a feet on the ground type of person, and that's what I do appreciate about all of our community partners, but yeah. If Janet were to retire, and you put somebody in, and they just said, hi, my name's Joe Jane. Will you do this? I'd be like, who are you. Like, is this a scam?

**FACILITATOR:** And can you all recall any immediate thoughts once you found out about the idea of participating in the program?

**FEMALE 5:** [INDISCERNIBLE 00:18:20] the test to give out. It was like, yeah, we all kind of jumped on it. I think Merced County, I mean, I was really surprised to hear Planada got so many people vaccinated, because for the longest time, it was hard to get out of the 30 percentiles as far as vaccinations were concerned. There was just a lot of people in the community didn't trust the vaccine, didn't believe in it, and we actually had a super high infection rate here. So, I think, when it seemed like when the tests were available, we had a lot of people coming and wanting the test, but they weren't willing to get vaccinated. They just kept wanting to do the test. So, it was kind of interesting dynamics in the community itself and their attitudes towards COVID-19. So, I think we knew, you know, in order to protect ourselves, we also wanted to make sure people were using the tests and getting out there instead of keep spreading it around.

**PARTICIPANT 1:** I remember that we had a, when Janet had contacted myself, we already had a naturally occurring vaccine clinic that we were doing in our back parking lot, and it was through the county, and the county had sent nurses. So it was, we already had a big event, and so, I was thinking, wow, what a great way to pair this, because we'd already done advertisement for the one in the back, for, you know, like you're saying Margaret, like we actually had the ability to compare the two. So, people that were actually coming through the front to get the free kits were like, we had point persons going, oh, are you here for your vaccine, or are you here for the kits, and they're like, oh, you're doing the vaccine, like, and we don't have to go through this electronic site. I'm like, no, if you want, come on in the back. We'll get your vaccine.

**[00:20:00]**

Then we'll pull you back through, and then you can drive out and get your kits. So, it was a one-stop-shop that just happened naturally, and I think that that was just amazing.

**INTERVIEWER:** I think initial thoughts for me was, okay, let's add one more thing to our plate, to be honest, right, um, because we were already doing emergency food at that point, and we are a small nonprofit, and so, my question was, how do I finance this, because I didn't only have volunteers. I also needed to have some staff supervising it at the different sites, and then recognizing that it took a lot of time, really, to make it happen, right, and while I thought it was really good and worthwhile, and so, we jumped on it, I was so grateful afterwards when Janet gave us a stipend to kind of offset the cost a little bit, because for us, as a tiny organization, and probably, Diane feels the same, anything that is extra is kind of like [INDISCERNIBLE] our little budget that we have. Right? So, I think my initial, like I said, my initial thought was, okay, how do we make this happen, but then also, recognizing there's a lot of pieces with that. Right? Even the transportation of getting the kids from Planada back to Merced, because there was a location where it was stored was like, okay, how many trips can I make with my little car, or who do I know that has a truck that we can, and all of those things. Right? So, there was just a lot of logistical stuff that was a little overwhelming for us as a small nonprofit, and yet, we so desperately wanted to help and make sure that people get the testing and then be encouraged to be vaccinated.

**FEMALE 4:** I thought it was absolutely essential. The community needed it, and I embraced it right from the beginning. I knew that most of the footwork was going to get done by me, and I was a little intimidated by that, but it turned out to be okay, and it was easy to recruit volunteers for this, because everybody recognized the need.

**PARTICIPANT 1:** I think the recruitment of volunteers came naturally for us, because Department of Developmental Services had mandated that all of our 21 regional centers across the state, all of our employees had to be vaccinated along with all of our vendors. So, with that being said, it was, there was a lot of feelings on that, because not everyone believed in the vaccine. So, then we became another tier that, okay, if you're not vaccinated through our legal journey, then you could do the testing. So, then they wanted to volunteer, because they're like, we can get test kits too. I'm like, yeah, you can get, you can go drive through your car and get the test kits too. So, they were really, it just was everyone, including our own staff. We intended to do it for the community, but that was an outcome that I did not anticipate, that the individuals volunteering really wanted them for themselves and their families too.

**FEMALE 4:** I am still getting free test kits now through their source through the state, and I'm giving out 3,000 a month via a food distribution. It's not too much work, because we're handing out food anyway. People are driving up, and we're putting them, so we're putting the test kits in bags with the food, with their canned goods, but there's still a demand in the community for this.

**INTERVIEWER:** And it's kicking up again, right? I mean, we have more people, once again. So, Diane, I want to find out where you get those kits from, because we need some more. So, we need to talk.

**FEMALE 4:** It’s by invitation from the California Department of Health. I'll send you the link, and you can see if it'll work for you.

**INTERVIEWER:** Can I just say, oh, I know Diane?

**FEMALE 4:** I don't know.

**INTERVIEWER:** And then your street cred is going to get me in?

**FEMALE 4:** The link didn't go to Planada in Action. It went to San Joaquin Drug.

**INTERVIEWER:** Okay.

**FEMALE 4:** But they won't send them to Sam Joaquin Drug, because they're a for-profit business. They send them to Planada in Action. So, I don't really know, but I'll send you the link, Monica.

**FACILITATOR:** Great, and I just had one more question around this piece, which is how, if at all, did the idea or process of working with other community groups, or a community group that brought together several community leaders, kind of this like community engagement model influence your decision to participate?

**INTERVIEWER:** That was a no-brainer.

**FEMALE 4:** Can you repeat that? It's a kind of complicated question.

**FACILITATOR:** Yes, sorry. Yeah. So, the question

**[00:25:00]**

is how did the process or idea of working with a community group that brought together other community groups to achieve the goals of the project influence your decision to [INDISCERNIBLE]? How did that idea feel to you?

**INTERVIEWER:** Okay.

**PARTICIPANT 1:** We do that naturally, though. To me, it feels like putting on the same pair of socks, I don't know.

**FEMALE 5:** Are you talking about our decision to get involved? Is that what you're wanting to ask?

**FACILITATOR:** Yes, yeah.

**FEMALE 5:** I guess I don't understand the question fully.

**FACILITATOR:** Yeah, I think it's around the idea of, you know, kind of what made you participate in the program. What was appealing to you about it?

**FEMALE 5:** Well, I think part of it is we're all involved in some kind of a nonprofit that's meeting a need in our communities, and so, anytime anything comes up that's going to meet the needs of the people we work with, we kind of jump on it. Even though we're dealing with people who are dealing with sight loss, when we heard there was a group giving out food, and we knew that some of our people were needy of food, and not just because of, also, because they were afraid to leave their house, and, you know, when you're blind, and you try to go to a grocery store, and they're telling you to go follow the arrows and keep so many feet away from somebody, and if they're wearing a mask or not, and you can't see that, it becomes very nerve-wracking to even leave your house sometimes. So, you know, that was kind of like, anytime we heard a need, when it came up for these COVID tests, there was an issue, because they weren't very accessible. I mean, even for myself, when I used it, it was like I had to use somebody with eyes to be able to say, is there a line. You know, is it coming out, until the accessible kits that came out a little bit ago, but because we took advantage of Free Matter for the Blind and we'd get people, we send out a notice to our mailing list, which is quite extensive and said, do you need kits. Let us know. You know, and we were able to send out kits using Free Matter to people, so they didn't have to leave their home to try to get them too, and I think being able to get it in different ways and formats that felt safe was important also.

**INTERVIEWER:** I think, for me, the importance of partnering with another agency had two, kind of, almost two or three different approaches. One was I knew Janet and trusted her. Secondly, I knew the organization that was heading it up, and that helped, and thirdly, it was really around one cause, and that was the COVID issue. Right? I think sometimes some issues get a little bit hijacked into political agenda things, which I don't necessarily always want to be part of, either direction, you know, whichever way we go. So, I think it had to do with knowing the organization, knowing the contact person, and having a trust relationship with them already, and then knowing who were some of the players that were participating in it as well. So, I think that kind of made it easier to make this decision. It definitely, definitely was working better to work through a local organization than working through Duke or anybody else that was the larger group, right, because there would've been so much red tape that I would've said, you know what, Forget it. So, I think anytime, I prefer partnering directly with local folks instead of the larger entities that has a lot more rigorous reporting or whatever. Right? Let somebody else do all the detailed things that need happen around that.

**FEMALE 4:** I agree with [participant name]. The integrity of the initiating organization was important, but the fact that we were a coalition of many nonprofits, shoot. I didn't even know that until I went to pick up tests and found other nonprofits were there. I operated pretty much in isolation from them. We didn't collaborate a lot with each other. United Way did all of the coordination, so that the sites weren't redundant and such like that.

**INTERVIEWER:** And it was such a fast turnaround. I mean, we went from, what, it was right around Thanksgiving to Christmas, we gave out, what, 200,000 kits or something. Right? So, it was like really, really hard work, fast work. We didn't even ever get together as a group of all of us. I mean, so I don't even know who was all part of it, honestly. We just knew, okay, we're covering this area, and Janet was coordinating who is covering which area, and we just did it.

**FACILITATOR:** Yeah, that makes sense. I just want to clarify, is Janet with United Way?

**FEMALE 4:** Yes, well, she was for this project.

**[00:30:00]**

United kind of project shifts. She's a project person. When an agency has a project they need spearheaded, they call Janet, and she will move between agencies, depending on who's got what going on. So, she was at United Way for this project, and then she went to the city, and I don't even know where she is right now.

**PARTICIPANT 1:** She actually had retired from the county. She had retired from the county, and so, when she contacted me, I'm like, Janet, what are you doing. Like, you're retired from the county, and she goes, I'm with United Way now, and I go, oh, cool.

**INTERVIEWER:** Yeah, she's one of those people that has nine lives or something. I mean, she's just a lovable person and really brings communities together.

**FACILITATOR:** Okay, the amazing Janet.

**FEMALE 4:** Yeah, uh-huh.

**PARTICIPANT 1:** It's Janet Casper Appling, Janet Casper Appling. I don't know if you need that for your recording, but we want to make sure.

**FACILITATOR:** Yeah, no, I think that, just to clarify, [INDISCERNIBLE] and I are, we're external evaluators. So, we actually have very little context about what actually happened on the ground in terms of specifics. So, any of that background is helpful. So, my next… Did anybody have anything else to add there around what kind of made you participate before we move on? Now, we just want to ask about what you thought about the Say Yes COVID test project in general. So, how, if at all, was the project responsive to the needs in your county related to COVID-19?

**FEMALE 4:** It was not. We don't have computer access everywhere, especially the people who are most in need, and a lot of what was required in order to comply with the project required computer access and internet access. The affordable connectivity program now has expanded that somewhat, but at that time, I was signing up people at the pharmacy for hours and doing their surveys and things like that. They couldn't comply, because they lacked the resources. Academics live in a world that’s full of internet, and everybody has technology. The real world isn't like that, and I think the assumption was made that it was.

**PARTICIPANT 1:** Yeah, that's exactly what I was pointing out earlier. It mirrors exactly our experience and just the lack of understanding, and they're like, well, they have free government phones. Well, I mean, you try to navigate some of this stuff on a small phone, and if you already, you know, you could have learning disabilities. You could be mildly intellectually disabled, I mean, different things that not everyone could do this. I mean, and this happens in all systems, the DMV, social security, everything, and so, the people creating these projects assume that we gave you access, and it should be great. So, I mirror what she's saying 100%. That's what I was trying to reference earlier, but she did it more, much better. Thank you.

**FEMALE 4:** Well, at that time I had a flip phone, not a smartphone. I could not tally my data on the fly, as I gave out tests. I had to do paper records and then sit down and put them in, and they were almost always late, and I mean, that's an example of the problem just on my side. On the client side, I know a lot of them never completed the actual study with the surveys, because they couldn't get online to do them. There was also a dearth of other language materials. The Spanish materials came late, and they were inconsistent. I mean, I'd get a box of Spanish and then none and then nothing but Spanish and no English, you know, it was inconsistent, and they weren't handed out by language.

**FACILITATOR:** Yeah, what about others? Similar experiences? Different?

**INTERVIEWER:** I totally…

**PARTICIPANT 1:** I just want to point out real quick, too, that we also serve a very long, large Hmong population here, huge here in here in Fresno and in Merced County. It's unique to us, and so, that's usually not in consideration. So, I think that we missed that population, unfortunately.

**[00:35:00]**

**INTERVIEWER:** Absolutely, I agree. Yeah, I agree with everything that has been said. You know, that even for us, we ended up doing paper copies and then went back and entered all the data into it, because it was just not possible to get it done on computers. Yeah.

**FACILITATOR:** Yeah, what about you, participant 136?

**FEMALE 5:** Again, a lot of the stuff was not accessible for the visually impaired and the blind. So, that was an issue. We had to, like, redo some of the directions into very large print, because those little cards that came with it didn't do it. We actually had, I don't know if you want to call it a clinic. We had some sessions on how do you use the test, because the paperwork that told you how to do it was also not accessible. So, there was a lot of issues around access for our people with using the kit itself was an issue on, you know, people would ask, well, can I use this if I'm blind? You know, well, no, you needed to have somebody sighted to be with you, and that also was a hindrance for the follow-up questions and the follow-up that was with it. So, that just, that was a negative part of all of this, you know, as far as that's concerned. Even the new accessible kits that have come out, you got to use an app with it. You know? So, it's like if you don't have that kind of technology or knowledge, it still is not going to be very accessible, and that's been an issue that even the blind organizations nationwide have had as a complaint about the whole thing that's been going on, especially as far as the test kits are concerned.

**INTERVIEWER:** And I think even, I think the importance of making it either a third-grade level reading or do more visuals, you know, because good intentions, right, we want to cover all of our Qs and Ts and whatever, but I think it makes it impossible for some of our folks to really follow, and I mean, for me, English is my second language, and so, even I at times have to go, what, say that again. What? So, I think making it simpler would be a very important part.

**FACILITATOR:** Sorry, wrestling a cat. Thank you for sharing that perspective. It sounds like you all kind of had similar challenges in terms of, or encountered similar challenges for helping your clients use the kits. Thinking specifically about some of the challenges you had in completing your part of the program, so, the tasks that you were asked to do, you've spoken a little bit about some of the data entry pieces and that being hard without a smartphone. Any other challenges or barriers that you encountered in terms of being able to complete what you, your part of the program and your tasks under the program?

**FEMALE 4:** Storage.

**FACILITATOR:** Can you say more?

**FEMALE 4:** Yeah, that was a real challenge. There had to be a space, and we didn't have a location. We don't have an office. So, I had to hustle around the community and find people who'd take boxes of COVID tests for me until I could get them out.

**INTERVIEWER:** I agree, yep.

**PARTICIPANT 1:** We had the storage. I think where our trouble was, was getting them into, there were bags, because once the drive through event gets going, you know, you get five or six bags or, I don't know. I'm still a little confused on how that went, because a certain amount of families was supposed to get a certain number, and another was supposed to get a certain number, and then we had all these bags, and, you know, we had a staff person literally all day, going and putting them in these bags, and so, I didn't, I don't, still, my takeaway is I know they were trying to give a certain number for certain families, but I don't think we really stuck to that, because, I mean, when cars are coming up, and you're going, I mean, you've got to move, and it's just like, here, take them. Here, take them, and so, I don't know how they tracked that, necessarily. I mean, at the end, obviously, we just counted the whole number that we provided, and some people were asking for some more, because they pick up for somebody else, because they didn't have a car, and they didn't have gas, and I found the volunteers that United Way brought were very understanding of that, but I think that there were some barriers for them doing that, because it was associated with a ZIP code and a number. So, I think that part of the process, especially because of the volume that we did, I think that that could be streamlined a little bit more, definitely, and they actually brought the mobile vehicle out for us, but the mobile vehicle had gotten broken into,

**[00:40:00]**

and so, we had a delay there, which, you know, was stressful, because people are coming, and then they want to park, and then other people are coming, and that's, you know, obviously, we couldn't predict that, and that happens, but I would be better prepared next time to know, like, how would we have people waiting that are already coming 20 or 30 minutes earlier. Do we have the capacity to do that? Not even because the van broke down. I mean, people were that desperate. We didn't have the space in the parking, probably because we had two events, but even if we didn't have the two events, we didn't realize that that was going to happen, and we didn't have a way to traffic control that, and then with us having, you know, the storage for us was on the backside, even though we had it. So, we had volunteers running from the backside, carrying all these bags to the front side, and then we had to recount more out, because we'd exceeded the number that we had, and we already had some there. So, it was a number game, and some things were colliding as far as, I think, safety, just overall safety of the volunteers and my volunteer staff too, that I would do differently next time.

**INTERVIEWER:** I think, so, for one, I don't ever want to have another pandemic just happen. Can I just say that? I'm done with that in my lifetime, but I think the other thing for us also was that we found out that some folks were actually getting, because there was only the ZIP code and the number of families. Right? So, families could go to numerous different sites and get kits and then actually sold them. So, especially, in one of our communities that happened, where people found out that people are desperate, and so, instead of telling them that they're free kits, they would just get their own free kits and then sell them, because people wanted to get tested. Right? So, so I think there has to be some way of, okay, how do we track this, but at the same time. we don't want to track it too detailed, because then people don't come at all. So, I don't know how that would work in the future, but again, I don't really want to have another pandemic. So, I'm good.

**FEMALE 5:** Well, I noticed we kind of dealt with that also at the very beginning, because we'd see cars pull up, and four people would get out of that one car, and they actually used to ask for their limit, and we're like, you guys are from the same family, you know, whatever, and then we kind of suspected some of that was happening. So, because we were really focusing on our population getting these kits, because they were having a hard time with other issues, you know, the barriers that were up there for the blind. So, we basically started limiting it to people that were, you know, on our list, so to speak, on our blind, you know, we were kind of wanting to make sure that the blind and visually impaired in our community were getting these kits. So, we started kind of limiting it to that, and we didn't get as many kits, maybe, as some of the other groups, it sounds like. We don't have a storage issue either. We did have plenty of space to keep it, but it was amazing how quickly the kits were going even then, you know, and then we were getting, because we did send out a notice to people, we were getting the phone calls saying, can you send it to us. Which that was…. So, I guess we kind of started controlling it a little bit more as far as who we were giving the kits to, because we were getting suspicious at the beginning of, like you were saying, people were grabbing them and selling them, because they were even having a hard time, you know, they weren't available in the stores. The shelves were empty, you know, and people were desperate to get the test kit. So, we kind of had dealt with that a little bit at the beginning and found a way to control it.

**FACILITATOR:** And what do you feel like helped you all accomplish the tasks that were asked of you? So, this could be anything about the program that you felt like facilitated your success in completing the tasks or things that you changed about the way you went about the work that you found helpful.

**PARTICIPANT 1:** I can say the biggest success, and we couldn't have done it without it, was the volunteers. I mean, you know, I don't know if they, I don't believe they, a couple were United Way employees, but I think that Janet rounded up other community partners that just chose to come out, that were retired individuals that volunteered, and they were just absolutely essential to the volume of people we had coming through. I mean, we had people, like, you know, different wings on traffic patrol that our staff did, but they had the volunteers going through getting the ZIP codes and, you know, getting the stuff out of… I definitely, I think that you need a large amount of volunteers, and that's helped the success of our event.

**FEMALE 5:** One thing that probably helped us too is we

**[00:45:00]**

also got connected with the state program that was giving out the masks and the hand sanitizers, and I'm trying to think. There were several things that we were able to give out at the same time. So, people who came in for a test kit, we were giving a mask, we were giving them out hand sanitizer, you know, other kinds of things also. So, they knew that they would be getting those also. So, that was kind of a help to it.

**INTERVIEWER:** And I think, for us, I think, in part, it was our staffing. I mean, I just have amazing people that hang out with me, and yeah. They just went beyond, and then again, volunteers, I think, you know. So, we're partnered a lot. So, we work specifically in a couple of neighborhoods that are under-resourced, and so, we're very, very focused on neighborhoods, but having volunteers there from the community, but then, also, I think, having food distributions going out at the same time was really beneficial, because it kind of brought two things together that were of necessity during that time for our communities.

**FACILITATOR:** Okay, what about others? Anything to add to what folks have talked about in terms of volunteers and being able to leverage some of your existing great folks that hang out with you?

**PARTICIPANT 1:** I honestly could say, even though there was that situation with the mobile van, that mobile van that they utilized, it's great for advertisement, because, just because you send out, you know, emails or these e-blast or messages like that, because we're on a busy road, we're on [PH] M Street, people driving by, I mean, it's just absolutely wonderful, and I know that we even had staff coming forward saying, can I get a picture with the van, and it became a real focal point of advertisement and just a real positive thing. So, I don't know who owns that, or how that came into play, but I mean, if you are in a busy location, you see that van, and you're driving by, then you can capture people right off the street that you wouldn't naturally. We had a banner, but you can't see the banner like you could the vehicle.

**FACILITATOR:** Right. Do you all feel like the project was successful at achieving its overall goal?

**FEMALE 4:** I don't know about the research goal, but we distributed a lot of dang tests, and it was successful in that way.

**INTERVIEWER:** Well, and I think, also, creating awareness for the need of the vaccine, you know, because in our communities, many of the folks, unlike Planada, somehow, many of the folks did not want to get vaccinated. So, one of our community members is in a very mixed neighborhood, and so, especially our African Americans were very hesitant, and even now, I still have staff members that are not vaccinated, and then, in winter, we also had community members that just said, no, you know what, we have been experimented on enough, right, and I think that goes back to the overall messaging around, the political messaging that's happening right now that is so detrimental, because we don't hear facts any longer. We hear interpretations of whatever the latest thing is, right, and it's about clickbait. It's not about truth and honesty any longer. So, I think it makes it very hard for our community members that don't know how to sift through all of the crap of the news right now and really finding good answers that protects their families, but also, keeps them healthy overall. So, I think, if this is one learning for all of us, I think it is ensuring that good messaging is going out, that is unbiased and focused and community-oriented, because yeah. I mean, you had the vaxxers and the anti-vaxxers. You had the, who cares about masks, and yes, double mask, and all of that, right, which makes it very difficult for folks who are already living in stressful life situations to have to make decisions on what's best for their families. So, I think good messaging is very important. I really appreciated the health department doing regular updates. That was helpful initially, because, so, Christine from the health department, every day, there were updates on saying, okay, this is how many people are sick. This is how many people are hospitalized, right, and so, I think it gave some real answers to stuff,

**[00:50:00]**

even though, still, some of my community members were convinced that those numbers are skewed, which were probably true, but making sure that we have good messaging going out, I think, is an important part.

**PARTICIPANT 1:** You know, I think, also, that what was concerning to our individuals is they would say the vaccines were free, but when you went there, they wanted your health insurance card, and so, that became like, well, why do they need my health insurance card? Like, am I going to get a bill? Is this not covered? Like, I can't afford this, and I'd be like, No. I went down there myself, and I think that was unclear as to why, if it's completely free, you know, why do you need that, and I think, when we did this distribution with a mask, you know, doing the ZIP code, thank goodness. Because what if we were asking them for their health insurance card? I mean, that would've built another, there was just a layer of distrust there. So, doing the ZXIP code was a blessing, because then people, you know, some people don't have health insurance. Then like you're asking for the card, and they can't get their vaccine. So, the ZIP code in that sense was very positive. I think that the success, like someone else had said earlier, of getting that out there was great, but the questionnaire afterwards, I would love to see that data, because I highly doubt that most of my individuals did that, just a guess on my part.

**FEMALE 4:** Just for your information, the reason for a health insurance card for vaccination is that the administrator can bill for an administration fee for giving the vaccine, not for the vaccine, and it is not required of anyone, but if they wish to surrender an insurance card, then the agency that's doing the administration can make a little money back for their services.

**PARTICIPANT 1:** I think, in some of our populations, where people were English is their second language, even though I tried to explain that, it's a complicated explanation for them, and it still built that fear almost like the INS in some other things. So, yeah. I recognized it, but to try to even explain that, I mean, it's kind of like earlier, someone had said that, for things to be maybe in third or fourth grade language. When you start getting in deeper explanations, you just feel like the government's controlling you and trying to number you, and I mean, there's all kinds of fears that go on, and I, honestly, if I walked in those shoes, I think I would probably have the same fears.

**INTERVIEWER:** Well, even at the testing sites, thinking about the backgrounds, right, on how difficult it was at times to just get basic testing done, right, the paperwork that needed to be filled out, and just, it was so important at that point, that people were getting tested and figured out where they're standing in all this. I think there could have been easier ways of even doing that, and not everybody trusted the home kits, honestly. Right? I mean, still, even now, I mean, we have, we're giving out home kits. We have some, and then they go to the doctor, and they have two different results, and so, then the question is, okay. Did they home kits not work any longer? Expiration is not, it hasn't expired, but it seems like that a number of folks that I have seen recently have done the home test and tested negative and then went to the doctor and tested positive. So, that, again, goes into that whole what's going on here. Either I am, or I'm not, right? I'm pregnant, or I'm not pregnant. I have COVID, or I don't have COVID.

**PARTICIPANT 1:** Well, and the expiration date, because we had several boxes, because cause we had a different set come from DDS, they did expire, and then what happened was we're like, we can't give these to families. Well, then, they had this little scan code on the boxes that said, now we've extended them. Now they're good for another nine months or so long. So, that built more distrust, like, okay you put an expiration date, but now, magically, they're all good for another year. I mean, that was very confusing. We didn't have that through this drive through, but I mean, they said that they were fine. The other thing was the pain factor. So, when the county was actually doing the one where the nose swab, and then people were going and telling people like, oh my god, it was so painful, like, I was like tears in my eyes and all of this stuff, and so, I myself was like, wow, I mean children and individuals, you know, our individuals that are autistic. Can you imagine an autistic individual like that already, you know, sensory touch issues, everything? And I was like just horror stories going on, and so, I myself had gone through the county and purposely went through the journey and the process, and in fact, it was true. I mean, she handed me at Kleenex. I was like, am I supposed to blow my nose? She goes, no that's because you're going to tear up, and I was like, wow, but then when I went to several other

**[00:55:00]**

testing ones that were done, it wasn't painful. They didn't go all the way. You know, I think some people got more zealous with that, and so, the more of our disabled that spoke to each other, then even if they had symptoms, they didn't want to get tested, because they were afraid of the pain, and so, I don't know, like, if some people weren't taught properly to administer and, other people thought you need to go full down the nasal cavity into your brain, or what happened there. I'm being facetious, but I'm not, because that really, we had people, especially the population I work with, that were afraid and then wouldn't go get tested, and that concerned me greatly, because then you're spreading it more.

**FEMALE 4:** Like a lot of things have to do with COVID, that has to do with the evolution of our scientific knowledge of what the virus was doing. The early tests were much more aggressive, and they become much less aggressive. The home kits are not at all painful, and can be so simple, because we understand more about the virus.

**INTERVIEWER:** Well, and I think I was going to say something similar to recognizing that we have never experienced a pandemic before, right? So, I recognize that the government had no clue what they were doing, and so, want to give them some grace in this, but I think consistent messaging is such an important part, and then, you know, as Maya Angelou would say, you know, you do what you know, and then when you know better, you do better. So, I think the same should be done in our scientific research and learning around all of that. Yeah.

**PARTICIPANT 1:** Oh, I agree wholeheartedly, and I also know that my own experience with COVID, with the people that had the last variant, because you had referenced earlier that it would be negative. That's because you had all the symptoms, and you actually didn't do a positive test until three or four days later. So, most of our staff, what happened is we needed multiple test kits, because even though we had the full symptoms, even though we were exposed and our family did, we'd have it for three or four days, continuously testing, but it wasn't until the fourth day that it would show on the test kit. So, we did the best we can, and praise God, you know, I mean, and ones that have lost people and those over that here that had the, you know, we tried. We all tried the best we could, and like you said, grace and love for everyone, for all of the efforts put forth.

**FEMALE 5:** Well, and I think it is actually understanding what those test kits test. They were testing your contagious level, so to speak, rather than whether you had it or not, because the only way you could really know if you had it or not was to get the, you know, the full, almost blood work, you know, on it, and I think that's the other part is why people is like, well, I was negative yesterday, and how come I'm positive today. You know, I've got the same symptoms. It was like, you probably weren't as contagious yet, yesterday as you are today. You know but understanding that those test kits were not whether you have it or not, but basically, your level of being contagious is what it was really showing with those home test kits. So, that's all that kind of information, as you're saying, was not out there for the general public, and it was a lot of confused information that was going out there, and that created mistrust too on those test kits.

**FEMALE 4:** Yeah. That information wasn't available to us either. Not only did patients not know, we didn't know, and so, I mean, we were all lost at that time.

**INTERVIEWER:** Yeah.

**FACILITATOR:** Right. So, I want to ask a follow-up question. You all are describing a lot of the reasons that people hesitated to get tested. What do you feel like were some of the reasons that people did pick up test kits? So, the folks that came to your events and picked up the kits, why do you think, what was their motivation?

**FEMALE 4:** [CROSSTALK 00:58:50], oh, go ahead.

**FEMALE 5:** No, I was just going to say, for our people, it was, being that they have to rely on public transportation, usually, to get around, if they thought they were positive, they didn't want to get on a public bus, public transportation to go get tested. So, they really, really wanted those home test kits, because, you know, they didn't want to expose other people too. They, you know, they had that conscientious part of, if I'm sick, I don't want to be spreading it. So, I want a home test kit. So, that was, a lot of our people wanted that, and then they had run into the part, I mean, we were actually showing them how to use, you know, cameras and stuff to, you know, how do you read these things, you know, and stuff, and we were having to talk people over the phone on how to use the test kit. So, that's, it was an interesting journey and still is with some of these test kits and on our population.

**FACILITATOR:** Sure.

**PARTICIPANT 1:** And there was also, twice, a period of time where you would get up to 80 hours if you were a COVID positive, and so, you know, along with what Margaret was saying is that, obviously, they didn't want to spread it, but also, I mean, at different

**[01:00:00]**

employments, unless you showed that you were positive, then you couldn't access that paid time off, and we had it, and then it actually, at one point, the first time they did it, it went retro. So, then we went back through that, but I know that that was utilized as a source of information for employers too, not just ourselves, many employers that needed that information to be equitable, I guess.

**INTERVIEWER:** And I think, too, one of the things was community members interacting with each other and learning from each other. Right? So, when Susie does it, and she's fine with it, and Jackie is doing it too, then maybe I should be doing it too, right, and take care of myself. So, I think the more positive messaging was going out among neighbors, the more people would be inclined to do this as well.

**PARTICIPANT 1:** That's why I didn't come back and tell anybody that I was crying after my nose swab. I was like, oh, it’s fine, you guys. It's fine.

**INTERVIEWER:** I had COVID early on, and I was negative for the first three tests, and I thought I was going to die, literally. I mean, I was like down for seven weeks, and on the fourth test, I finally tested positive. Right? So, yeah, it's such a crazy pandemic.

**FEMALE 4:** The message I found most effective for trying to get people to take tests was protect your family. We did this distribution at the holidays when people were gathering, and they really do care about their families. You know, don't put your parents at risk. Protect the grandparents, things like that. People are responsive to those kinds of messages.

**INTERVIEWER:** Well, especially in the Latino families, where it’s so much more common for people to live together multi-generationally. Right? So, I think that was a very vital message to give out at that point as well, yeah.

**FEMALE 5:** Yeah, we had a lot of people that would come in and ask for the test, because we're getting together with people, and before we get together, we want to, you know, we want to do the test. So, even if they didn't have symptoms.

**FACILITATOR:** So, thinking again about the tasks that you all were asked to do as part of the program, how did those compare to what you normally do in your work or at your organizations? How big of a difference or departure was it for you to take this on?

**FEMALE 5:** Well, I wish we could just do ZIP code. We don't, we have to get so much more information. So, if you were to compare what we have to gather with our normal clients that we work with versus this distribution, it was a lot easier to just have to get ZIP codes on people.

**FACILITATOR:** 58	What about others?

**FEMALE 4:** It seemed routine to me.

**INTERVIEWER:** What?

**FEMALE 4:** Yeah, I said it seemed routine to me. It was just another project. It wasn't harder or easier or… It was another project.

**PARTICIPANT 1:** It wasn't routine for us, because we're not direct service providers. We link with all the community partners, whether that's the food banks, the visually impaired, the deaf and hard of hearing, the rural community supporters out in Planada and Blanco whatever, everything. So, we're not typically the direct service providers. We coordinate and then, you know, send the information. So, it was out of our norm, for sure.

**INTERVIEWER:** Yeah. I think a lot of us experienced out of our norm with COVID, right, because like, we don't do mass food distribution. We don't do mass anything, because we're very focused on relationships, and so, we just had to pivot, because that was a necessity for our communities at that point. So, and glad we did it. You know, I don't regret a moment of it, but it was definitely not something that we would usually do.

**FACILITATOR:** And how hard was it for you all, those of you who said that it was a pivot, how hard was it for you to make that pivot, or what were some of the resources involved?

**INTERVIEWER:** We were able to get… Go ahead.

**PARTICIPANT 1:** I was going say that, for us, I don't think it was that difficult. I mean, because we roll our sleeves up, you know, at different times, you know, different projects, and everyone wants to do something more of the direct services in our particular agency. It's just that we don't want to go in where somebody's already naturally doing that, because we all have our roles, and so, we're really respectful of the roles of the direct service providers. So, everyone was actually very happy to get to be the, you know, the ground and to do that. Ours is a little bit different, because someone had mentioned earlier

**[01:05:00]**

about the financial aspect. Like, we didn't accept the money, and we were very happy if another agency could accept the money, the smaller agencies that don't have the same level of funding as us, and that's true of the $50 that you're offering today. So, if that can go donated somebody else, we'd happily do that, again, because it's just, we're more like mental health, and so, the smaller agencies, we don't want to take from their glory or their funding for what they do there, because we support them in their efforts.

**INTERVIEWER:** Thank you.

**PARTICIPANT 1:** You're welcome.

**INTERVIEWER:** And we should have known about that earlier. Diane and I would've knocked at your door. We know for next time. Can you repeat your question one more time?

**FACILITATOR:** Yeah. So, and I had a follow up for you, because I feel like you spoke to this maybe early on when we've been in the conversation, for those who said that it was quite a departure from your normal activities, and that it required some pivot, my question was how difficult that was, and then what level of resources were required to enable you to make that pivot?

**INTERVIEWER:** Good question. So, for us, we had extra staffing available. So, our community centers are, one of the community centers does a lot of afterschool programs, and of course, there was an afterschool program. So, we pivoted in that regard and created packages for the kids and had the kids pick up the packages, so they were not at the center, still, got the activities to do. We usually don't do mass food distribution, but because there was this huge need, especially for our senior citizens at that point. So, we did mass food distribution, but then conjoined it with the COVID kits and stuff. So, that kind of made that a little bit easier. I think it ended up being a financial. I don't know if we came ahead, honestly, financially, even with that stipend that we got, which again, for a small nonprofit is always a difficult thing, and I think, sometimes, government agencies don't realize that, when they give us these jobs to do, that there is a ton of work and a ton of administrative stuff that needs to happen that either takes us away from what we’re already supposed to be doing or just becomes a financial burden for us. Right? So, and again, I would do it in a heartbeat again if need to be. So, but just, I think there needs to be more of an awareness about it, and I think it would've helped, even my staff, knowing that there was going to be a little bit of extra compensation or something would've made it much easier for them in times when their own families were at risk. Right? So, I think recognizing that our staff members are all single parents, and so, they had to make a decision. Do we serve the community, or do we protect our children? Right? So, I think we just got to figure that out.

**FACILITATOR:** Yeah. Yeah, thank you for sharing that. Anyone else have anything to add on this point? Our next question is about communication within the program. How, if at all, could communication have been improved, or what were your impressions, overall, about how the communication worked in the project?

**INTERVIEWER:** Communication with Janet was awesome. Communication from Duke University was not. It was totally overwhelming.

**FEMALE 4:** Agreed. The university people were completely out of touch with what was going on.

**FACILITATOR:** Yeah, and they had different…

**INTERVIEWER:** Amen!

**FACILITATOR:** What are some examples of…? You said that you described it as overwhelming. Could you say a little bit about what that looked like, or what, specifically, was overwhelming, or if folks have anything else to add around that?

**PARTICIPANT 1:** I wanted to add that, because we had United Way, and we had United Way’s, volunteers and then we had our staff, I think that if we had a pre-meeting, you know, maybe half an hour before, we would have known our roles better, because everyone, with great intent, was trying to help each other, and there were some discussions that were occurring between all three different parties, because everyone was trying to jump in and help each other out, but we had some confusion, and we had some harsh words

**[01:10:00]**

at one point between their staff and my staff. So, I was running between the two and explaining it. I think we could have better planned and had a pre-meeting, because we just had so many wonderful people there that all wanted to jump in and help, but you definitely have to have identified roles, because if you don't have someone doing the traffic control or someone doing this, and then, you know, then we needed breaks. You know, people needed breaks, because we did it all day long, and then getting that communication that, you know, kind of that planning ahead of time, I think, would've really helped. In the end of the day, I mean, everyone hugs and everyone's thankful for it, but it's pretty, it was very stressful during the event though, to be honest, because of not having that pre-meeting.

**INTERVIEWER:** You hugged each other? Shame on you! No, I'm just kidding.

**PARTICIPANT 1:** Yeah, it's an interesting thing, because the population that I work with, you know, we tried the best to teach the elbow and the fist bump and everything, but, you know, most of our individuals are so isolated, and when they see us, like, we're really like family with them, and it's just, you know, the reality. I mean, I was pretty terrified myself, because I'm the provider for senior here in my own home, and, you know, I just, you know, I just, I couldn't stop myself, and I think a lot of us couldn’t. You know, we tried the best we could, but those people that really needed a hug, I mean, we did, and I wouldn't change it either, because I think I needed a hug too.

**INTERVIEWER:** Absolutely. I was just more being facetious. [INDISCERNIBLE]

**PARTICIPANT 1:** I know you were.

**INTERVIEWER:** Yeah.

**PARTICIPANT 1:** Yeah.

**FEMALE 5:** Well, when you talk about communication, are you talking about the distribution, what came with the kits? I think we were a little bit, at the beginning, unsure of exactly how and what was expected of us, and I'm not really sure where, you know, it was kind of like, here we've got these. Give them out. Get their zip codes, you know, but there was just that other step. Okay, wait a minute. Got these cards in here, now, what are we supposed to do with these? Oh, there's a QR code on here. You know, there was just a little bit of confusion, and then we had to like paddle this thing. Okay, how can we make this accessible and readable to our people? Because trying to get your phone to pick up a QR code that's even difficult, even if you know how to use your technology. So, there was a little bit of, you know, we had to do some adapting to what was given to us to begin with. So, I guess that's just part of it, which is very common. We're always having to back-paddle, but like I said, I think we were a little bit unsure about exactly are we doing what we're supposed to do, you know, because the, it was, we didn't have any event, like Barbara is talking about. Ours was like, okay, we have these test kits here now, and we can get them out to you and let them know about it, and they started coming in droves to get them, and so, that was a, yeah, it was a little overwhelming, you know, at first, but we kind of got our system down.

**FACILITATOR:** Yeah, and so, were you able to get responses from folks, I guess, either Duke or Janet, to help you clarify and get that system done, or was that something you all just figured out on your own?

**FEMALE 5:** Through Janet? We worked directly with Janet with all, most of what we did as far as communications are concerned.

**FACILITATOR:** Great.

**PARTICIPANT 1:** Us, Janet as well. Yeah, I don't know the Duke part of it. That didn't occur.

**FEMALE 5:** Yeah, I don't ever remember. I remember reading something that was probably connected to that, but all of our communications was through Janet, and then it was a little bit unsure, because people were supposed to get money for responding to letting, you know, the little, they're supposed to go somewhere to say, I did the test, these were my results, and then they were supposed to get some kind of card. I really don't know if anybody got anything.

**PARTICIPANT 1:** We didn't.

**FEMALE 5:** Yeah.

**FEMALE 3:** [INDISCERNIBLE] again, but nothing ever came.

**INTERVIEWER:** Yeah, which again, then goes back to people don't trust the government. Right? You promised me this. I followed through. You did not follow through.

**FACILITATOR:** Right. Yeah. Yeah, that's big. We're about at time. So, I'll have this be our last question, but feel free to share widely, but I just want to know if there's anything else that you all would like to share about the program, anything like we haven't already talked about, and specifically, any recommendations that you might have for something like this in the future?

**FEMALE 4:** The design team, the lead

**[01:15:00]**

team needs to have some community people advise them on the mechanics of how things will work, because they lack that insight.

**FACILITATOR:** So, in this case, that was Duke or the academic connection?

**FEMALE 4:** Yeah, their team.

**FACILITATOR:** Yeah. Got it.

**INTERVIEWER:** Well, and even understanding, for me, I'm a big picture person. How does Duke University get involved with all of that, right? There was a whole other thing where I'm like, I don't get it. Is this just one of those science experiments that you guys are doing? How does this all work together, right? There was no explanation on why Duke instead of Stanford, here in California, or any other California-based organization or university was doing this, and then shipping, like the timing on the shipping, even, was off, because it came from who knows where, probably China, but, you know. So, I think having more community input on those things would be helpful.

**FACILITATOR:** Yeah, I see some folks nodding in agreement. Anything you want to add on to that or any separate thoughts you want to share? Would you all…?

**INTERVIEWER:** I need to ask a totally ignorant question here.

**FACILITATOR:** Go right ahead.

**INTERVIEWER:** [INDISCERNIBLE], where are you from?

**FEMALE 6:** I'm Nigerian.

**INTERVIEWER:** Okay, thank you, because I've been puzzling the whole time, and I'm like, okay, I'm going to just be bold and ask the question. Thank you.

**FEMALE 6:** No problem.

**FACILITATOR:** You said English was your second language. Where are you from?

**INTERVIEWER:** Germany, but I learned English in Bluefield, West Virginia, if that makes any difference at all.

**FACILITATOR:** Wow. Interesting.

**FEMALE 5:** I recognize that accent. My parents are from Germany. So, I was like, oh.

**INTERVIEWER:** Is it a good thing or a bad thing? Because I hear some people, like, they hear the German accent, they think of their grandmothers who are really, really strict. You know, Germans are like type A personality to the fault, and so…

**FEMALE 5:** No, it makes me want to talk German because I [INDISCERNIBLE].

**INTERVIEWER:** Oh, good [GERMAN 01:17:27].

**FEMALE 5:** Yeah. [GERRMAN 01:17:31]. No, it's just, you know, it's kind of, you know, my background in linguistics, you know, it's, you hear a language, your brain just wants to, you know, you hear that accent kind of thing. That's why I brought that up, but anyway.

**INTERVIEWER:** Yeah.

**FACILITATOR:** So, one final question, since we have at one minute, and it can be yes or no, or you could say more, but would you all participate in a program like this again?

**FEMALE 5:** Yes.

**INTERVIEWER:** Yes.

**FEMALE 4:** Yes.

**INTERVIEWER:** I don't think that [INDISCERNIBLE] heard you.

**FACILITATOR:** Okay. So, the very final question is would you participate in a program like this again?

**FEMALE 3:** You bet you. Our community needed it.

**FEMALE 5:** Yeah.

**FACILITATOR:** Hoping for no more pandemics, but…

**INTERVIEWER:** Yes.

**FEMALE 5:** Well, I'm a little worried about the fall and the winter coming up, and I'm hoping people will actually take some of the precautions they took for COVID to prevent big flus. That is, you know, we're supposed to get a bad flu this season, they're predicting and things, and they said, actually, we haven't had a lot of flu the last two years, because people were protecting against COVID. So, maybe we learned something out of this, that, you know, to take some of the precautions, but of course, there won't be the testing for it, but I do think the testing made people aware of, like we were saying earlier, you know, you're going to meet with elderly parents, people with maybe some kind of, like we had, you know, cancer, maybe going through different kinds of health or things, and you didn't want to expose somebody to another disease. So, they tested ahead of time. So, just making people more aware of what they pass around was, you know, that was a good, positive thing of this.

**FACILITATOR:** Yeah, that's true. Well, I think we can end with that. I really thank you all for this rich discussion, and it was really great to hear about your experience. I know [INDISCERNIBLE] might have some steps or instructions in terms of us passing out the gift cards. [INDISCERNIBLE], do you want to jump in?

**FEMALE 6:** Yes. Thank you, everybody, for your very insightful discussion about the CS COVID test. Yeah, this was really helpful. So, the next step would be, you'll be getting like an evaluation survey just to get your thoughts on how the focus group went, and then just a confirmation of your email and name to send the gift card. So, but that will be after. Yeah, that will be after today, but you should get some, like, thoughts page on how the focus group went and then confirmation of your email and name for the gift card. Thank you, all once again.

**INTERVIEWER:** And it was for and $120,000 each?

**FEMALE 6:** No, I'm afraid not. Yeah, just a thank you for your time, just something small.

**PARTICIPANT 1:** [INDISCERNIBLE], would you like me to complete that and then make a notation that I wouldn't be receiving that, and would that be sufficient for you?

**FEMALE 6:** Okay, I can note that and…

**PARTICIPANT 1:** But if you need me to complete the feedback, I can do that, and then if there's somewhere to note it, I'm not sure.

**FEMALE 6:** Okay. Yeah, you can note it, and then I will let the team know, the person sending out the gift card, know that, and yeah. Thank you, and that's [INDISCERNIBLE].

**PARTICIPANT 1:** Thank you. Yeah, we have a company policy that we don't accept gifts, even through this, so it's just part of our company. No disrespect to your beautiful gift.

**FEMALE 6:** No problem. Thank you.

**FEMALE 5:** Can't use it as a prize at one of your auctions or anything?

**PARTICIPANT 1:** Yeah, I thought of that too, and then I don't know. Then if I do it for a few, then everyone else, you know, even if you try to buy coffee, it just gets complicated. So, I'm sure that there's lots of people that are going to partake in this. So, thank you.

**FACILITATOR:** Okay. Thank you, everybody. Enjoy the rest of your afternoon.

**INTERVIEWER:** Yeah, you too. Bye.

**FACILITATOR:** Bye.

**PARTICIPANT 1:** Thank you, bye.

**FEMALE 3:** Thank you.

**FEMALE 6:** Bye.
