# Supplementary material for: Exploring barriers and facilitators of implementing an at-home SARS-CoV-2 antigen self-testing intervention: The Rapid Acceleration of Diagnostics—Underserved Populations (RADx-UP) initiatives
Source: PLoS One. 2023 Nov 16;18(11):e0294458. doi: 10.1371/journal.pone.0294458 (PMC10653400; doi:10.1371/journal.pone.0294458)
Supplement: S1 Dataset — (ZIP) [file pone.0294458.s002.zip › FG 1.03_Focus_Group_Guide_March 2022 Final.doc]

**Focus Group Procedures and Discussion Guide**

**Purpose: The purpose of this focus group is to evaluate the experience of community organization leaders or staff that were *recruited to participate in the SYCT project*, either as a community resource, distribution site, or other project-related community engagement activity.**

**Consent Process**

Consent forms for focus group participants are completed in advance by all those seeking to participate. Below is a summary of the information in the consent form that focus group organizers and facilitators will use to make sure participants understand the information in the consent form.

*Thank you for agreeing to participate. Duke, UNC, NCCU and the SAY YES! COVID TEST team are very interested to hear your valuable opinion on your experiences with the SYCT project.*

- *The purpose of this project is to learn how best to partner with communities and community organizations to engage with COVID-19-related health and health promotion efforts.*
- *The information you give us is completely confidential, and we will not associate your name with anything you say in the focus group.*
- *We would like to tape/record the focus groups so that we can make sure to capture the thoughts, opinions, and ideas we hear from the group. No names will be attached to the focus groups and the recordings will be destroyed as soon as they are transcribed.*
- *Due to the virtual format, we will assign you a number that will be used in place of your name during the web-based focus group (e.g., Participant 1).*
- *You may refuse to answer any question or withdraw from the project at any time.*
- *We understand how important it is that this information is kept private and confidential. We will ask participants to respect each other’s confidentiality.*
- *If you have any questions now or after you have completed the questionnaire, you can always contact a project team member like me, or you can call the SYCT project team leaders whose names and phone numbers are on this form.*
- *Please sign the adjoining consent form if you agree to participate.*

**Focus Group Questions:**

***Let’s start the discussion by talking about what makes [Insert State] a good place to live.***

1. What are some things that you enjoy about living here in North Carolina?
2. What are some things that aren’t so good about living here in North Carolina?

***Now I want you to think about how your county responded to COVID-19.***

3. Thinking now about your area’s local response to COVID. Were you satisfied with this response to COVID?
   a. If yes, why were you satisfied?
      i. What did the County do well?
   b. If no, what more would you have liked to see from the County’s response?
4. How do you think that your County’s COVID response compared to what was happening nationally?
   a. What made it similar? (Probes: Good communication, ease to accessing care, others)
   b. What made it different? (Probes: Level of communication, perceptions of safety policies)

***We are also interested in learning more about how you typically get your health and COVID-related information?***

5. Where do you typically get your COVID-19 related health news and updates from? *(Probes: Friends, Family, News outlets or materials, online sources, social media, etc.)*
6. What makes a source credible in your opinion?

***Now I want you to think about your perceptions when your organization was contacted by someone about the SYCT project.***

7. What did you think about that initial communication?
8. Can you recall any immediate thoughts? (Probes: Positive, Negative, Neutral)
9. How, if at all, did the process or idea of working with a community group that brought together several community leaders influence your decision to participate in any way?

***Now I want you to think about the SYCT project in general.***

10. How, if at all, was this project responsive to the needs in your county related to COVID-19?
11. What are some of the challenges or barriers, if any, that you experienced during this program?
12. What, if anything, helped you accomplish the tasks that were asked of you?
13. What do you think was the overall goal of the project?
    a. In your opinion was it successful?
    b. Why or why not?
14. What do you think was the biggest factor driving county residents to pick up COVID-19 testing kits?
15. What do you think was the biggest reason that county residents were interested in knowing their COVID-19 status? Probes: safeguard family, occupation, other?
16. What measures, if any, were taken to ensure the needs of all county residents were equally considered in the SYCT efforts?
17. Thinking now about your exposures to health and COVID information in your community, did this project help you identify credible sources of information?
    a. If yes, how?
    b. If no, why?
18. How did your tasks for this project compare to your normal activities with the organization that you are representing?
19. How could the communication have been better?
20. Is there anything else that you would like to share related to this program?
21. Are there challenges or barriers that you experienced that were specific to your race/ethnicity, age, gender, sexual identity, linguistics/language, or geographical location in completing your tasks or working with this project?

***Finally, let’s switch gears to talk about your experience.***

22. What encouraged you to agree to work on kit distribution?
23. If you had to pinpoint one thing that had the biggest influence on your signing up for SYCT with your community organization, what would you say influenced you to sign-up?
24. How, if at all, was the SYCT team responsive to your specific community’s needs?
25. How, if at all, has working with the SYCT project impacted your confidence in talking about COVID?
26. Would you participate in a project like this in the future? Why or why not?

*Probes for Discussion:*

- *Language/Translation services*
- *Needed information*
- *Transportation and other barriers*
- *Variety in community organizations*
- *Feeling respected/ heard*
- *Satisfaction*
- *Trust*
- *Bias*

That concludes our focus group. Thank you so much for coming and sharing your thoughts and opinions with us. If you have additional information that you did not get to say in the focus group, please feel free to write it on this evaluation form.
