# Supplementary material for: Exploring barriers and facilitators of implementing an at-home SARS-CoV-2 antigen self-testing intervention: The Rapid Acceleration of Diagnostics—Underserved Populations (RADx-UP) initiatives
Source: PLoS One. 2023 Nov 16;18(11):e0294458. doi: 10.1371/journal.pone.0294458 (PMC10653400; doi:10.1371/journal.pone.0294458)
Supplement: S1 Dataset — (ZIP) [file pone.0294458.s002.zip › NCFG-Transcript (8.25.22).txt [Christina].doc]

**AMELIA:** So, first we’re going to start with just some general questions. For starters, what are some of the things you enjoy about living in North Carolina? So, I know no one wants to be the first to talk, but Participant 36, you’re off mute. So, do you want to kick us off?

**PARTICIPANT 36:** I know, just for me, I was born and raised here in North Carolina and one of the things I like is just the friendliness. The experiences of all four seasons, the fall, the winter, and summer and spring. And the wages versus the cost of living as well. That’s about it.

**PARTICIPANT 18:** Good morning.

**AMELIA:** Morning.

**PARTICIPANT 18:** I’m going to preface this. I had eye surgery about a week-and-a-half ago and my eyes tear, so if you see me with my, like blotting, I’m not crying about this. I’m not upset.

**AMELIA:** Okay, good to know.

**PARTICIPANT 18:** Like my dog is fine, no one broke up with me. I just, my eyes are tearing a lot so, I just wanted to preface that because I didn’t want anybody to think I was having a breakdown today and need to be worried about me. My eyes are kind of crazy. Anyway, my favorite thing is I’m very active and so, I like where I am. I have mountains, I have beach. I’m not in a big city but I have access relatively easy for cultural things and I just like to do a lot of things. So, for me living here -- I’m from Virginia and where I lived in Virginia didn’t have as good of access as I have here. So, I like that a few hours I can be in the mountains, a few hours I can be at the beach, or I can be in a -- drive for a theater show one night and come back the same night. So, for me I just, I love the weather’s nice. I just love, I like the variety that North Carolina has so, I’ve been very happy here.

**AMELIA:** Great, and what about you, Participant 33?

**PARTICIPANT 33:** I’m not very good with Zoom. But I love where I live. I live in the small town of Winterville and so, the small town living I enjoy that. I enjoy being the proximity to places like Raleigh and other places, but I don’t want to live there. I like the small town. I love the climate that we have here. We do have distinct seasons and I just love the people and the fact that I feel safe where I stay at. That’s one thing, I really feel safe. And I can be active in the community because it is a smaller community, and I can get to know people in larger cities still.

**AMELIA:** Mm hmm, yeah. Yeah. And what would you say are some things that aren’t so good about living in North Carolina?

**PARTICIPANT 36:** Aren’t so good?

**PARTICIPANT 18:** I would say I have young kids in the school system and I’m very concerned about our teachers and our, I think -- I can’t compare it to other states because I haven’t lived in other states with a family, but I know that North Carolina oftentimes is kind of cited in terms of our ranking and things for what we’re doing for our teachers and I’m concerned about the mass exodus that we’ve seen and the amount of just openings and things in the school system and the fact that our teachers aren’t feeling very supported. I think it’s a very tough situation for them and I think working in higher ed and seeing a lot of students be excited about wanting to teach and then when they get out realizing that they’re not getting the support. I know other states have problems, too, but I feel like education is a big thing on the North Carolina burner and certainly it’s an issue for me because I want our teachers to feel like they’re valued and want to stay in their profession. And I think especially the last few years we’re really seeing a mass exodus. So, I think for me that’s a concern statewide, is just making sure that that doesn’t become -- I think it’s in a crisis mode right now and it’s something to be concerned about for our teachers and our kids.

**AMELIA:** Mm hmm.

**PARTICIPANT 33:** And I think that North Carolina doesn’t give us, we don’t get the national recognition that we deserve as such a great place to live. And some of the,

**[00:05:00]**

some of the legislative things and things like that I feel like we are looked down upon, more than we are recognized for the strides that we are making.

**AMELIA:** Mm hmm, mm hmm.

**PARTICIPANT 36:** I know for me living in the eastern part of North Carolina -- I’m in Winterville as well -- the economic growth. I think I’ve seen this part of our state go more from a manufacturing base to more of a service based. And so, I would say the lack of more manufacturing and the lack of opportunities for people that’s not really going to college into those specific areas like nursing or doctors or dental and things like that.

**AMELIA:** Mm hmm. Yeah, those are all really great examples. I thank you for sharing that. We’re going to focus in a little bit about Covid 19 specifically. So, thinking about your areas local response to Covid were you satisfied with the response and how things went?

**PARTICIPANT 33:** It took a while for the people in my, that I was, the groups that I was working with to really see the relevance of the tests. And by the time they caught onto what they were getting the project was almost over. But we were able to distribute quite a few in the southern part of the county but like I said, some of them they didn’t, if you asked them did they want, no, but then later on they came back, do you have any more? That kind of thing.

**AMELIA:** Mm hmm.

**PARTICIPANT 36:** Yeah. I think the education around Covid itself and the impact, knowing our communities a lot of times the lack of education makes people more reluctant to participate. We did participate in a lot of Covid kits distribution, and it gave us an opportunity to kind of educate before we passed out the kits and things like that. The only other disappointing to me is the receptiveness of wearing masks [INDISCERNIBLE] and the pushback of wearing masks to prevent this from spreading was kind of disappointing as well.

**AMELIA:** Mm hmm, mm hmm. Yeah, and I think -- oh, go ahead, Participant 18, if you want to jump in.

**PARTICIPANT 18:** No, it’s okay. It’s okay, you can go ahead.

**AMELIA:** No, I was just going to build on what you all are speaking on and ask what you feel like your county did well specifically and what were maybe some areas for improvement? I feel like you’ve highlighted education and masking maybe as some potential areas where things didn’t go so well.

**PARTICIPANT 18:** I think for us, I think it was hard because I think the first year, kind of with the pandemic everybody was very supportive and everybody was kind of from a healthcare perspective people were, we were healthcare heroes, and everybody was happy for the fact that we had testing and happy for the fact that we were sharing information. We all kind of felt like the second year it turned and it was more, people felt like it was, it was kind of being thrust upon them and I know for me, I’m in a university setting, we did have some mandatory things. And I think the second year it felt like people pushed back a lot harder on us and it felt like it was things we were doing to the in a penalizing way. And certainly, we didn’t feel that way. We felt like the more testing we had available the more we could have adequate surveillance of what was happening in our immediate area. And I think for us it felt very different, the last part of it. It felt almost like people were adversarial to testing and they were not really, not really happy about the fact that we were, we kept pushing stuff out. People kind of were, I think they had some fatigue with it and kind of wanted us to, everybody wanted it to go away but it wasn’t going away and I think we just got a lot of pushback. Especially parental pushback and that kind of filtered down through the students. The parents would kind of be angry about things and it was just a different feel to us. Like I said it felt supportive and we were all in this together and then as the vaccine came out and, in the beginning, it was kind of supportive and then for us it felt like it kind of turned a little bit. Almost like we were the bad guys. So, I think from a healthcare perspective we felt almost like we were adversaries

**[00:10:00]**

instead of working with the community. And so, that was kind of difficult for us just because we didn’t feel any different. We were just trying to keep everybody safe, but we felt like the public mood, particularly masking and things like that felt like it kind of made a turn to where it wasn’t as supportive for us.

**AMELIA:** Mm hmm, mm hmm. Yeah. Anything else to add around that from the rest of the group? I guess I’m just wondering too, how much you all feel like -- and this kind of leads into my next question. If the kind of challenges that you’re describing were related to the way that your county specifically responded to Covid versus kind of what was going on nationally?

**PARTICIPANT 18:** I mean, I thought we did great here and it sounds like other people are in my same area. So, compared to what I was hearing from friends in other states, we had a lot of testing sites open. It seemed like the vaccine; I think our town did great rolling out at the convention center and having these mass events and I really thought in terms of I would see people on social media post that they couldn’t get appointments and they couldn’t find vaccine sites and --

**PARTICIPANT 36:** Mm hmm, yeah.

**PARTICIPANT 18:** -- I feel like we had a lot of opportunities in Pitt County. I feel like we had a lot of sites open. Our drive through site was -- it was long waits, but you could get tested I felt like compared to some other places relatively easy. And then like I said when the vaccine came, I felt like Pitt County did a really good job of making it as available and easy. I heard nothing but good things. We didn’t participate in the convention center vaccine efforts; we were doing our own on campus, but it seems like people had very positive feedback about most things. So, I thought we actually had a fairly robust response.

**PARTICIPANT 36:** Yep, I tend to agree as well. I think we were a lot better than a lot of the other states so, I would agree with the availability of the various testing sites, the distribution of the Covid testing kits and things like that. I think we were above the norm.

**PARTICIPANT 33:** Mm hmm, and I agree also.

**AMELIA:** Okay, that’s great. I think we’re also interested in learning a little bit about how you typically get your Covid-related information. So, where do you typically get Covid-19 related health news or updates from?

**PARTICIPANT 36:** I know for me I do a lot of research on my own. Just really actively going out to read the various articles around Covid. I also have two daughters that’s nurse practitioners and they’re pretty much in tune with what Covid is and explaining that as well. So, those are, those are my sources there. I don’t rely a lot on the news.

**AMELIA:** Mm hmm.

**PARTICIPANT 36:** So, I mostly try to read it from articles and other health professionals.

**AMELIA:** Mm hmm. Yeah, the article that you typically read are you finding those online or how do you come about those?

**PARTICIPANT 36:** Mostly, mostly online, yeah.

**PARTICIPANT 33:** Well, I found that we had almost daily updates from the Governor’s office on television and I tried to stay abreast of them and the information that was being passed out in those broadcasts. Of course, newspapers. I read newspapers of course, and I receive just direct emails now. With offers for Covid kits and that kind of a thing, but those daily updates from the Governor’s office and the news on television. I did keep up with a lot of the things that kept me updated as to what was happening in the area as far as the Covid numbers and where to get tests. Where to get vaccinations? How to get it and things like that.

**AMELIA:** Mm hmm.

**PARTICIPANT 36:** I’d also like to add too, the Pitt County health director made himself available. I was part of a couple groups that we actually invited him in, and he came in to answer questions and really

**[00:15:00]**

shared a lot of information as well.

**PARTICIPANT 18:** I read a lot online. I read a lot. I’m on Twitter a lot and kind of follow a lot of med Twitter related articles and things like that. We meet as a group. We have a -- it’s changed names quite a few times, but we essentially have like an infection control committee here at the university and obviously it ramped up quite a bit and became more of like a pandemic group. But we share stuff in that group. Somebody finds something we kind of keep a lot of things going in that group. Also, we get regular updates from Dr. Silvernail and from DHHS and certainly CDC and things like that. So, kind of lots of different sources. Some of them more professional chain and then personally. I read a lot of stuff off-work and we just try to share things that we read and that we think are applicable. We get a lot from our governing organization, American College Health, and they put out a lot. They do a lot of advocacy so; they have a lot of physician papers and they put out a lot of guidance. Typically, when CDC updates they help update for higher ed so that we have kind of a formal body that shares for all of the system institutions and things. We also meet with the UNC schools pretty often. We have a director’s group that all of the 17 UNC state schools, we meet pretty much weekly and chat between health directors and folks that are in administration just to make sure what’s happening in other universities across the state we’re all -- if we need advice from another school or just to kind of make sure we’re all doing similar things.

**AMELIA:** Mm hmm, yeah, that makes sense. And you all have spoken on this a little bit in your responses already, but in your opinion, what makes a source credible?

**PARTICIPANT 33:** I guess the data is one thing.

**AMELIA:** Mm hmm.

**PARTICIPANT 33:** And of course, if they are officials speaking about it. I notice someone mentioned Dr. Silvernail, of course DHHS, the Governor’s office, those kinds of things. That would make it credible to me.

**AMELIA:** What about others?

**PARTICIPANT 36:** Yeah, that’s a pretty tough, pretty tough question. That’s why I did rely on a lot of news sources because you do get the conflict and opinions. And I tend to agree with what the previous speaker said, speaking of people in positions of knowledge like the Pitt County health director, the Governor and things like that, so. All the professionals.

**AMELIA:** Mm hmm.

**PARTICIPANT 18:** For us it’s kind of our partners and what sources our fellow partners are looking to. Like I mentioned kind of our governing bodies and kind of the information that they share out and that they use. Things that are peer-reviewed, things that are -- we look at a lot of the data from other schools that are of similar size and similar makeup and where we are geographically. And that helps guide us because that’s actual peer institution information and so, that’s usually very credible to us because students typically in similar types of academic situations we can kind of compare information because we tend to see trends. So, I think that, making sure it’s peer, making sure it’s scientifically based and endorsed by the folks above us that help us make our decisions.

**AMELIA:** Mm hmm, mm hmm. Yeah, that all makes a lot of sense. Now I’d like for you to think a little bit about your perceptions or how you felt when your organization was contacted by someone about the SYCTP specifically. What did you think about that initial communication or what was that like for you?

**PARTICIPANT 33:** I was excited to be part of the project. I really was, because this was something that was going on in our community and I felt like this would be a positive thing to do and to have in your household so, that you could easily test. I knew that it took a long time in those lines to be tested and so, I was really excited about being able to do something --

**[00:20:00]**

in the battle against Covid to help people.

**AMELIA:** Mm hmm. We may be having a little bit of trouble hearing you, Participant 36. It’s cutting out a little bit.

**PARTICIPANT 36:** [PH] Amelia, can you still hear me?

**AMELIA:** Yes, now we can.

**PARTICIPANT 36:** Hello?

**AMELIA:** Yeah, we can, we can still hear you a little bit.

**PARTICIPANT 36:** Pardon me?

**AMELIA:** I said we can still hear you.

**PARTICIPANT 36:** Okay. [INDISCERNIBLE] being a part of the distribution and communication, is that right? I missed that part of your question, I’m sorry.

**AMELIA:** Yeah, how did you -- what was your initial reaction to the idea of participating in the project?

**PARTICIPANT 36:** Yeah, I was excited. [INDISCERNIBLE].

**AMELIA:** Okay, we’re losing you a little bit.

**PARTICIPANT 36:** [INDISCERNIBLE], so, I was excited to have been a part of it.

**AMELIA:** Great, so, excited, I heard? What about, what about others?

**PARTICIPANT 18:** Yeah, we were, we were excited. We were glad to be able to participate and have the testing available and yeah, it was great. It was great to have that and to be able to offer that.

**AMELIA:** Great. And how was it all, or I guess what was your reaction to the idea of working with a community group that brought together several other community leaders? How did that kind of influence your decision to participate in the project?

**PARTICIPANT 33:** I felt that it was going to be a great opportunity for our community to have those kits at home, and so, it was a great opportunity to work with others. We were all working towards a common goal and we got to know each other. We got to know some of them, and I worked with them. Some of them I already knew but others I welcomed the opportunity to do something in Pitt County to fight Covid.

**PARTICIPANT 18:** Yeah, I wasn’t really involved in the initial, I was just basically told because I ran the testing clinics. I was basically told we were getting them and to distribute them but there wasn’t any negative for us. We had been trying to increase access and increase availability and really ramp up and really at that point encourage people to be testing. Any way we could get that to our community we were in favor of, so. I wish we’d had them earlier and had lots more of them honestly, and still had them. I wish we still had them.

**AMELIA:** Mm hmm. We’ve actually been hearing that quite a bit from folks, so.

**PARTICIPANT 33:** And I guess my experience was a little different because I was coordinating it for Winterville, Ayden and Grifton. So, I had to reach out and touch base with the churches and the ministers and the civic organizations and ask them if they wanted them. Ask them how many they wanted and that kind of a thing. And I had to deal with some people wanting more than they were distributing and doing things like that. I even worked with Ayden Housing Authority. They got like a hundred at one time. And then I worked with a church that got 100 and they only distributed 50. And I had to go back and get those. So, I got to reach out and touch base with some community organizations which meant that they were glad to get it, to get the kits and the members were taking them.

**AMELIA:** Mm hmm, yeah. I see that you came off mute.

**[00:25:00]**

Did you want to add anything?

**PARTICIPANT 36:** There’s not a lot more I can add. It was just good to be a part of a common effort to make sure that the distribution went well. So, I don’t have a lot to add to that.

**AMELIA:** Okay, great. So, yeah, our next set of questions are just diving into your experience with the project in general. So, how, if at all, was the project responsive to the needs in your county related to Covid-19?

**PARTICIPANT 33:** Repeat your question, please?

**AMELIA:** Yeah, so, the question is how, if at all, was the project responsive to your county’s needs related to Covid-19? So, do you feel like the project addressed an important need?

**PARTICIPANT 33:** I feel like the project was help with what we needed at that time. People were not, as mentioned before, the lines at the testing sites were very long and by having the kit at home you could test immediately. What, 15 minutes? Whereas when you went over to the site, which I did go over and get tested, you’re talking about an hour, an hour-and-a-half to go through. To get through. And so, at that time we needed something to rapidly tell people if they had Covid. Or to even if they say well, I take the test and it comes up positive I’ll go to the site, too. But it gave them that kind of information, which was needed. It was most definitely needed at that time, because we needed to diagnose it so we would know how to isolate and do the things we needed to do so we wouldn’t be part of the spread.

**AMELIA:** Mm hmm, mm hmm. Yeah. What about others?

**PARTICIPANT 36:** Just to clarify, just to clarify your question, Amelia, was it around the responsiveness of the effort? Is that what you’re trying to get to?

**AMELIA:** Yeah, so, I think the question is essentially like did the, was the program useful for what the biggest need was at the time in your county?

**PARTICIPANT 36:** Okay, yeah. I think it was pretty useful. I think with anything, the initial startup of anything may be a little restrictive. You experience long lines and things like that, but I think over time as the process improved, I think it met the needs of our community pretty well.

**AMELIA:** Good, good. Participant 18?

**PARTICIPANT 18:** Yeah, I think similar. Like I said we could have given out a lot more of them and we could have -- at that point our processes for kind of accepting home tests were not what they are now. And so, we utilized and where it was kind of towards a break in the semester and so, we were able to let students use them less for, like they couldn’t use them for university requirements but it was nice for them to have them and have so many so, that if they did wake up and they weren’t feeling well or if they had an exposure they had ample ability to kind of screen themselves before hopefully participating in things that you wouldn’t want to participate in. And so, for us it was great. Like I said we would love to have lots more of them. So, I definitely think it was positive for folks to have access.

**AMELIA:** And what are still the challenges or barriers, if any, that you all experienced during the program?

**PARTICIPANT 33:** Well, I couldn’t get enough tests. At several times I had requests that I had a hard -- well, eventually I was able to juggle around and get some but distribution of the tests I felt like that was, that was the biggest hurdle that I had. Getting it not from me to the person or the company or whatever agency, it was getting it from the, I guess, I don’t know, I say warehouse.

**AMELIA:** Mm hmm.

**PARTICIPANT 33:** But wherever they were stored at. Finding out where they were and then getting them from there. Setting up a time to go and get them. I think we changed the process a couple of times. One thing we changed leadership. It was, I won’t say changed leadership, but another person was put in place

**[00:30:00]**

to administer it and that person, and the person before when I got involved in the program were two different people and they did things different ways. And I had a hard time just getting in line with what they were doing.

**AMELIA:** Mm hmm, so, I want to make sure I’m understanding what you’re describing. Was it more so the transition between the two leaders that caused issues getting the tests or was it that one person handled things in a way that wasn’t so great and then it changed?

**PARTICIPANT 33:** It was the transition more that did it. The transition and the methods for distribution. Because at first it was like okay, I was told even at my initial, to get my initial group of tests I had to send someone at them and while they were on the way the place to go changed. The actual place to get them from changed. And then after that it was like okay, where do I go to get some more? That kind of thing and it changed several times.

**AMELIA:** Mm hmm, yeah, that does seem challenging. What about others? Any similar experiences or different types of challenges?

**PARTICIPANT 18:** Ours was just we could have given out a lot more. I mean, ours was pretty simple so, we didn’t really have any challenges. We were already right here and holding events and stuff, so, distribution wasn’t an issue for us. It was just once people realized what they were and what was available, it took a little bit to kind of -- because again home tests at that point weren’t quite what they are now, but once people figured out that they were available and especially the amount that were in the, in each test kit, we could have given out lots more. And like I said, still could. I still get people asking for them.

**AMELIA:** Mm hmm. Yeah, and what about you, Participant 36?

**PARTICIPANT 36:** I think my experience was the same as the two previous ones. I think a lot of the coordination I think locally may have been the issue just really trying to get things coordinated on how we would distribute them. But after those things were resolved I think everything else was well.

**AMELIA:** Mm hmm, yeah, and in terms of coordination with your organization and your experience what were some of the issues that you encountered?

**PARTICIPANT 36:** One of the biggest things we did initially, we had the distribution coming to several different people within our community. And what we did was we had, we changed it to have it come into one distribution. One person, Churches Outreach Network, so, all those kits came in there and then the local churches, the local community would go there and pick up their supplies and then distribute them to the community.

**AMELIA:** Mm hmm.

**PARTICIPANT 36:** But before that they was coming in, they was coming in from different avenues. But I think having that one central distribution for our area, it helped us to, it made it more efficient.

**AMELIA:** Yeah, so, centralizing, streamlining things?

**PARTICIPANT 36:** Yeah.

**AMELIA:** What were some other things that maybe helped you accomplish the tasks that you were asked to do under Covid? So, another way to say that is what were some things that kind of facilitated the success of the program?

**PARTICIPANT 33:** Well, they actually made kits available at community events. There were tables set up and that actually helped a lot. I think that everybody that participated had already relationships with other groups. Like I said the ministers and I in the Winterville/Ayden/Grifton area, we had people to call up and we could tell them we got these kits. Do you want some of them? How many you want? That helped out. The people that we already knew and the other civic groups we knew, that helped out a lot and just the concern in the community about Covid was good enough to open the door. When you say okay, I got Covid kits you can use at home. That opened the door to a lot of people as to the convenience of that kit. Those kinds of things added to our ease in distribution

**[00:35:00]**

of kits in the area.

**AMELIA:** Mm hmm.

**PARTICIPANT 18:** I don’t really have -- I mean, we had the testing kits and people grabbed them. I don’t really know the [INDISCERNIBLE] much more to say. People were excited to have them. Once they figured out what they were they [INDISCERNIBLE].

**PARTICIPANT 36:** Yeah.

**PARTICIPANT 18:** So, I think it was a pretty simplistic thing to offer and people were just happy to have the access. And that they were free. Students love free things. Everybody loves free things but students really, students really love free things.

**AMELIA:** Mm hmm, right.

**PARTICIPANT 36:** Yeah. Yep.

**PARTICIPANT 33:** And the experience that I had was that one, the churches here in Winterville -- I live in [INDISCERNIBLE] -- that we gave, I called and they said okay, we’ll take 25 -- which was a box -- 25 kits. The lady that actually came to the church and picked up, came to my church I belong to and picked them up, do you know a couple months later she died of Covid.

**AMELIA:** Mm.

**PARTICIPANT 33:** Died of Covid. But that’s how relevant and significant it got to be to know if you got Covid in the neighborhood.

**PARTICIPANT 36:** Wow.

**AMELIA:** Yeah, wow. Anything else there? I saw you nodding your head, Participant 36 along with what others shared.

**PARTICIPANT 36:** Yeah, I don’t have anything to add there.

**AMELIA:** Great. So, what do you all think was the overall goal of the project? Or I guess if you had to say it in your own words how would you describe it?

**PARTICIPANT 36:** I was, I was --

**PARTICIPANT 33:** I thought it was -- go on.

**PARTICIPANT 36:** I’m sorry. You can go on, Miss.

**PARTICIPANT 33:** Well, I thought it was overall early identification of people with Covid, which was a preventive measure to keep it from spreading so fast. That if we identified them then they could go, people that had Covid they could go in isolation. My granddaughter and my daughter both had kids. They, my granddaughter started feeling bad; my daughter could immediately test her. She didn’t have to go through the long line and she found out she had Covid. So, therefore she started measures to isolate her and of course I isolated myself because I was around them a lot and I didn’t know if I had it, even though I was tested and the tests came out negative.

**PARTICIPANT 36:** Yep. And I tend to agree with that comment. I think it would be more around, the goal was overall education around Covid, early detection of Covid and then you go into the prevention of Covid. So, those are the three things that I think I walked away with the goal being.

**AMELIA:** Mm hmm, mm hmm. Anything to add?

**PARTICIPANT 18:** I’d say for us just that breaking down the barriers of there was a lot of testing available but if you didn’t have transportation or if --

**PARTICIPANT 36:** Yes.

**PARTICIPANT 18:** -- if there wasn’t, if it just didn’t -- even though there were lots of opportunities like we talked about before -- we recognized there’s still a lot of barriers and I know for us in our situation our big thing was just trying to keep the other folks safe around you. So, people having a test kit, I mean, it’s great that you can, if you wake up you can check before you get involved din things and I think that was what we needed from the get-go I think universally. I think the ability to, if you don’t feel well, to immediately check and even though it’s not perfect we know that having that access could have made a big difference if we had had it across the board. I mean, I think it’s great that we had it with these kits and having that many kits in a, in a, I mean tests in a box that’s really a great thing. Exposure-wise or symptom-wise, it gave people a really fast check and helped them be more responsible for screening themselves versus having to -- and then for us we had lots of testing available but there’s still a lot of barriers. I mean, people still had to get to us. It had to be during the hours that we were available. You know, some people work or they have class schedules that they couldn’t come when we had testing or they didn’t have transportation to get to a Vidant test site, or whatever the case may be. And so, I just think it was great for the more testing and the more surveillance,

**[00:40:00]**

I think it was just, I think it was beneficial. I think from the get-go it would have been nice if, I mean, it’s nice that we have free tests available now and you can request them by mail and things like that. But I think had we had that from the get-go and people could check or people like us with kids. They’re always sniffling and sick and it’s just so great to have that easy access in your home that you can do really quick. I just think the availability of the testing was a key factor in slowing things down and we just didn’t have that to begin with.

**AMELIA:** Mm hmm, mm hmm. Yeah, so, my next question is kind of building on what you were speaking on in terms of how, if you all felt like the program was successful in meeting its goals. It sounds like, Participant 18, that you’re saying that from your perspective it was. What about others?

**PARTICIPANT 33:** Yes.

**PARTICIPANT 36:** Yep.

**PARTICIPANT 33:** I feel like it was very effective.

**AMELIA:** Okay. And I guess why or how do you feel like it, like what made it the most effective?

**PARTICIPANT 33:** Well, the things that she said as far as availability. The fact that you could do it right there at your home. It was easy to do, too. It wasn’t real technical and it allowed you to isolate quicker. And isolating we were slowing the spread and that was the goal of everything that we did during Covid was to slow the spread of it and the severity of it.

**AMELIA:** Mm hmm, mm hmm.

**PARTICIPANT 36:** Yeah, I tend to agree. I think just some of the testimonies you hear about I tested myself and I found I had Covid and I isolated myself. But just hearing those stories from all the people I think is a great indication that the program was pretty successful.

**AMELIA:** Mm hmm, mm hmm. Yeah. Yeah, that makes sense. And again, you all have spoken on this a little bit so, the next question is what do you think were the biggest factors driving county residents to pick up Covid testing kits? What I’ve heard so far is it was easy, it was free. Anything else that you feel like really caused people to pick up the kits?

**PARTICIPANT 33:** People dying close to them from Covid.

**PARTICIPANT 36:** Yeah.

**PARTICIPANT 33:** That really made it serious business. I don’t know, I don’t have any numbers to back it up, but I know it’s not many of us who are not aware of someone who died from Covid during the high spread, the times when we had a lot of people in it and everything.

**PARTICIPANT 36:** Yeah, I tend to agree. Fear. Fear had a lot to do with it. Not really understanding it had a lot to do with it.

**AMELIA:** Mm hmm. Yeah, and when you say not really understanding it do you mean, I guess can you say a little bit more about that?

**PARTICIPANT 36:** Well, I think the fact that some people would get Covid and some people -- some people would get Covid and be okay and some people would get Covid and not be okay. And not really understanding the difference. And I know there was education around underlying conditions to kind of give us some insight, but the fact that not understanding or making that connection I think it really did get people to say okay, I want to test myself. If I can catch this early, I want to, I want to do that.

**AMELIA:** Mm hmm. Right, right, that makes sense. It kind of felt unpredictable.

**PARTICIPANT 36:** Yeah.

**PARTICIPANT 33:** And that, and that not understanding it is like my daughter and my granddaughter -- my granddaughter got Covid. I think she got it from a driver’s ed class. But my daughter got it. Now, I was around my granddaughter, but I didn’t get it.

**PARTICIPANT 36:** Yep. Yep.

**PARTICIPANT 33:** I had another -- my daughter has two girls. The other daughter was right there in the house with them. She went into isolation with them, and she never got Covid. And you know, then of course we have that term asymptomatic. So, I can’t say I didn’t have it. I don’t know if I had it. Those kinds of things, the unknowns and people not catching it and some people catching it and you being in the same place with someone, and they have it and then you don’t have it. That kind of a thing led us to question marks. What’s going on here? What’s the difference? So, those are the kinds of things that I agree with --

**[00:45:00]**

I say Williams, because it say it on your shirt. I agree with him about we didn’t know.

**PARTICIPANT 36:** Yeah.

**AMELIA:** Mm hmm. What about the student population? Folks in the academic setting?

**PARTICIPANT 18:** In terms of what?

**AMELIA:** What made people pick up the kits? What do you think was the biggest factor that led them to want to use the kits?

**PARTICIPANT 18:** I think convenience. I think that they would come and get tested when they had to but I think there were a lot of situation where they knew for instance maybe they were going to be around somebody that, maybe someone that they knew was more at risk and they, it wasn’t time for like one of our mandatory tests but they -- a lot of people were taking good responsibility of I want to make sure that I’m okay before I go and see someone or before I do something. And so, I think for them it was convenience of we know that there’s testing available but there’s a lot of times in between those that we want to check, or we don’t feel good, or we want to, we want to just make sure. And so, I think for them it was just the convenience of being able to do it at home versus having to come to a testing site and then wait for the results and that kind of thing. So, I think for us it was, it was pure convenience. And I think too just the having them as a backup. Having them as an, if I need it, or if it’s at night and I feel sick or if it’s on the weekend and I feel sick and I don’t want to wait for a testing site to open. Because we had a lot of anxiety too, around it, especially being in communal environments. People were pretty cognizant that they had roommates, or they had sorority sisters, or they had other people that they had intermittent contact with. So, we’re only open like regular office hours so, students know if they started to feel bad in the evening, waiting until the next day until they could get to an official test site and then still waiting probably another 24 hours. We did have some, we have some 4-Plex, like rapid PCRs that we can do, but students want it right now. And so, if at 9:00, at 9:00 they start feeling bad they want to test at 9:00. So, I think for us, and even us adults and staff members that used the test too, I think it was purely convenience and being able to do it in the comfort of your own home versus having to -- even though they were available -- having to find an official testing location.

**AMELIA:** Mm hmm. Yeah, and so, you all have talked about people picking up tests or feeling like they wanted to test because they knew they had been exposed to Covid or because they started feeling bad. Did you notice any other reasons that people were using the tests? So, let me give you an example. Like people in a certain occupation that maybe felt like they needed to test an additional amount or if people lived with family members who had preexisting conditions? Anything like that?

**PARTICIPANT 36:** I will add that my mom, she’s 84-years old and during the height of the pandemic we were very, very careful about testing before we went to see her. So, I think like if I know if I’m going around elderly people I think -- I know for my mom we was very adamant about people testing before they went to visit. And also just my daughters being in the healthcare field their need to test -- they tested a little more than I think the average person was. So, I’m thinking based on, based on who you’re around and your area of work would drive you to test a little more.

**AMELIA:** Mm hmm. Mm hmm.

**PARTICIPANT 33:** Not wanting to be part of a group that spread. You don’t want to be the contact for people to get Covid. One of our councilmembers, his wife didn’t go anywhere and he was out some. But there was a lady who came to see them and she worked with people that had Covid and his wife got Covid. He got Covid. His wife died.

**PARTICIPANT 36:** Wow. Wow.

**PARTICIPANT 33:** So, this lady walks around now with that guilt on her.

**[00:50:00]**

She feels responsible. And we don’t want -- we didn’t -- we don’t -- not that she, she couldn’t help it, you know. I know she couldn’t but what I’m saying is that she blames herself for that and we don’t, we didn’t want to be, those of us didn’t want to be part of it so, we were testing to make sure when we went around people that we weren’t the point of contact for them.

**AMELIA:** Mm hmm.

**PARTICIPANT 18:** We had some specific groups that used some of the kits that were higher risk or groups that were not physically here. There’s a lot of university activities that we go off-site for or that, like our theater students for example, there are certain areas that we’re at higher risk because they couldn’t mask. Some of our musical students that weren’t able to have some of those precautions. We saw some students in those areas have more of an interest because they knew they were in a higher-risk environment. I don’t know that we had any professors, per se, but I do know we had some students. I know I had some theater students that particularly in the theater world they weren’t masking when they were getting ready for productions and stuff. And a lot of actually the theater, the governing bodies of actors and theater, they had some mandates that people that were in a production tested and so, we were able to allow the students to use because it was not practical for them to come to test at a test site. I think they had to test three times a week or something like that and they were able to use antigen tests for that. So, I know we had a few students that used them when they were kind of in the throes of their production for that short period knowing that they were at risk. So, they would use them as kind of more -- and we talked to them a lot about the limitations of an antigen test but they were using them kind of in a serial fashion and so, thinking about the other people in that production and how they’re not able to take, they weren’t able to mask or have social distancing in those types of situations. So, we did have a few, a few groups like that where we were able to utilize those tests as kind of a safety measure for them.

**AMELIA:** Mm hmm. Mm hmm.

**PARTICIPANT 18:** And then we also, we do a camp for a couple of days with incoming students and we have a provider go, but we don’t have a lab or anything off-site because it’s at a rural camp. And we were able to take a test kit there just in case anybody at the camp got sick. It was, it was kind of a nice way for us to make sure we didn’t have any issues but it was also nice to have because we can’t take our lab with us and we couldn’t take those types of tests. But it was nice to have that as a potential if someone at the camp developed symptoms, they had the ability to kind of test them and certainly isolate. I mean, we would isolate them anyway if they had symptoms, but to have more information. So, we were able to use it in that capacity that I remember.

**AMELIA:** Mm hmm. Yeah, those were good examples. What measures, if any, do you all feel like were taken to ensure that the needs of all county residents were equally considered in designing the program? Or executing the program? Oh, [INDISCERNIBLE] muted. Sorry.

**PARTICIPANT 33:** Excuse me. The use of media to -- the advertising that was done. The television, the radio. It was in the churches; it was on signs. Everywhere you saw there was information for people to get free tests for Covid. And even now you still see the signs. We see the signs. I been to events where they pass out free kits. It was like the agency had them and they were passing them out. I think that that’s been done very effectively.

**AMELIA:** Mm hmm. Mm hmm. So, making sure that we used a variety of media, that it was targeted. What else? What are other people’s thoughts on this?

**PARTICIPANT 36:** Can’t think of anything else to add to that one. I think there was a good use of media, all forms of media, to get the word out, so, I don’t have a lot to add there.

**PARTICIPANT 18:** Yeah, and I’m not -- I’m kind of in a bubble here so, I’m not exactly sure from the broader community perspective. I’m not sure I really have any input on that.

**[00:55:00]**

It was kind of targeted for us here, so.

**AMELIA:** I guess put a different way, do you all feel like there were any groups of people for whom the program didn’t work or wasn’t as accessible?

**PARTICIPANT 36:** I think the only thing I would say there, I think where the involvement of the different communities, the different groups within the community, I think having that in place allowed the communication and access to certain communities. I think if those people or those groups were not in place, I think you would have had a group of people that would have been left out of that whole loop. I know one, I know one of the things that we did was try to reach out into the Mexican community. There’s a group here called Mexi-Can that we reached out to, to make sure that there was resources and things were there. So, I think by having groups within the community already there it made it easier for that distribution. If those groups were not there, I think we would have had a part of our population that was, that wouldn’t have gotten access.

**AMELIA:** Mm hmm. And thinking about your exposure to Covid-19 information or the exposure to Covid-19 information in your community, do you feel like this project helped you or your organization identify credible sources of information?

**PARTICIPANT 33:** Yes, because so much of it used some of that same data that came from the health department, came from the state. It reinforced the guidelines to governors. The Governor was asking for us to follow, to try to stop the spread of Covid. So, we had that and it also helped us -- people would ask you for other things like masks during that time. Sanitizer, that kind of thing. I got to know about some agencies and resources that I did not know existed in Pitt County because of working with the testing, distribution of testing kits in the county.

**AMELIA:** Mm hmm. And so, building off that question a little bit, how did these tasks or the tasks that you were asked to do under the SYCTP, how did they compare to your normal activities that you normally do in your work?

**PARTICIPANT 33:** Well, I’m a government official so, I’m elected to represent the citizens and so, pretty much I’m out and about in the community and I serve, I won’t say I serve as a, I hate to say a resource, but I do get calls about the various things in the community. And people during that time after the word got out that I had kits, they started calling me for kits, and they didn’t mind it. And my information, my contact information was out there. It was public, and so, pretty much it kind of aligned with what I do I might as well say from day-to-day because when you’re an elected official your job does not stop with your meeting at 9:00. It’s your everyday life and people have access to contact information, your contact information, so, it’s easy to get in touch with you.

**AMELIA:** Mm hmm, mm hmm. Yeah, what about others? I thought I saw Participant 36 talking, but you were muted.

**PARTICIPANT 36:** Could you repeat the last part of your question? I missed some of that, Amelia. Could you just repeat it for me?

**AMELIA:** Yeah, definitely. So, the question is how the tasks, your tasks, under the projects and what you were asked to do aligned with what you normally do at your organization? So, how new was this for you?

**PARTICIPANT 36:** Okay, yeah. Pretty much like the previous speaker mentioned. Just being involved in the community already providing information and things like that. It fit in perfect with the, with the Covid. It was, we were already in the community. We were already involved, so, it was a natural fit to use

**[01:00:00]**

that same infrastructure to do the community with the Covid kits and the communication around Covid. So, it fit perfectly with what we were already doing.

**AMELIA:** Mm hmm. Mm hmm.

**PARTICIPANT 18:** Same for me. I mean, I do public health and education first events about all kinds of health issues. So, I was thrown into the Covid testing arm when we had to basically shut down all of our regular operations and just go to a Covid, we were basically just a Covid command center for the university. So, everything we normally did shut down and we just divided up testing, vaccines, tracing, tracking. And so, I was thrown into Covid testing but in terms of what the program, patient education, health promotion, healthy behaviors, preventative. All those public health tenets, that’s what I do every day. So, it was just a matter of it was Covid for this, sometimes it’s chlamydia, sometimes it’s HIV or Prep or Narcan.

**PARTICIPANT 36:** Mm hmm, yeah.

**PARTICIPANT 18:** The subject for me changes based on what’s happening but the mode of delivery and how we address and kind of tackle health education it doesn’t necessarily change. So, for me it was just a different thing to educate people on from a health perspective.

**AMELIA:** Mm hmm. Okay. So, we’re going to switch a little bit to talking about your personal experience with the program. So, what would you say encouraged you to agree to work on kit distribution?

**PARTICIPANT 18:** I didn’t have a choice, so, I don’t know. I don’t know that I had any say in it. I mean, I certainly would have done it had they asked but I was running Covid testing so, it was like hey. I basically just got this information, and my boss said hey, we’re getting all these test kits and we get to promote at-home testing, so, I certainly would have agreed to do it and would have been glad to be able to break down those barriers, but I didn’t have any choice in the matter.

**AMELIA:** Yeah, what about others?

**PARTICIPANT 36:** I’ll just say for me, I’m just an advocate. Personally, I love, I just love speaking for people that can’t speak. I love connecting with people that may not be connected, so, that’s what drove me. I think if I wasn’t part of an organization, I still would have volunteered to say hey, how can I help to take this to people that may not get access to it?

**AMELIA:** Mm hmm.

**PARTICIPANT 36:** So, just personal, it’s just personal motivation and personal satisfaction just being a part of that. Making sure that people are okay.

**AMELIA:** Mm hmm.

**PARTICIPANT 33:** And I think it’s the same thing. That desire in us to be of service to people and to help build our communities. That’s what it’s all about and we wanted to help and this was a way to help with what was going on. To educate and provide a service to the citizens, to the people that we live around.

**PARTICIPANT 36:** Mm hmm.

**AMELIA:** Mm hmm, mm hmm, yeah. Yeah, that’s great. And what would you say if you had to pinpoint one thing that had the biggest influence on your organization participating in the program? What do you feel like influenced your organization to sign up?

**PARTICIPANT 33:** I’d say the severity of the problem. The severity that we were seeing all around us like I said before. We all know someone who died of Covid.

**PARTICIPANT 36:** Yeah. Yeah.

**PARTICIPANT 33:** And the fact that it was affecting our community. Our children were out of school. We had people not working. Things like that were going on in our community, and our work force was being affected by Covid because we had in the Town Hall there, we had people out and we couldn’t, certain things had to be changed, the way we did things. And also, the way, the different things in the offices. The shields and things like that. So, the severity of the problem was major.

**AMELIA:** Mm hmm. Mm hmm.

**PARTICIPANT 18:** I think, for us, we were, we were willing to grab onto anything that we thought would help and so, obviously we had a vaccination piece and a testing and masking and a social distancing. And I think as the previous speaker mentioned

**[01:05:00]**

it was just such a large problem that we had to really --

**PARTICIPANT 36:** Yeah.

**PARTICIPANT 18:** -- anything we felt like would help in accessibility to this resource was obviously absolutely. We will grab onto anything that will help keep cases low and keep people out of the hospital.

**PARTICIPANT 33:** Yes, mm hmm.

**PARTICIPANT 18:** Help us [INDISCERNIBLE] with the healthcare system and really just help to keep the community safer. So, I think for us it was a no-brainer. It was, it was just absolutely. There’s no doubt [INDISCERNIBLE] making this more accessible for people and that’s just one component of trying to stop the spread of this. And so, it was an easy agreement for us to participate.

**AMELIA:** Mm hmm, mm hmm.

**PARTICIPANT 36:** Yeah, same for me. Schools shut down, jobs impacted. It really required all hands on deck so, it was a very, it was very easy decision to make.

**AMELIA:** Yeah, that makes a lot of sense. And how, if at all, do you feel like the SYCT team, so, the folks working in the program, were responsive to your needs in implementing the program or others working in your community to implement the program?

**PARTICIPANT 33:** I was able to contact them and talk with them about any problems that I had, and it seemed like they understood some things, anyway. I’m not as good with computers and Zoom-ing and all the different types of programs that some of the other people were -- and I still have some difficulty with some of them. And they were willing to do the format that I knew how to do. So, I could stay communicated. So, I appreciate that.

**PARTICIPANT 36:** Yeah, yeah. I tend to agree with the previous caller. I know using different forms of ways to communicate, everybody doesn’t have access to computers. Any everybody maybe by just having, just ensuring that they responded in various ways that told me that they were, they were really trying to be responsive.

**AMELIA:** Mm hmm.

**PARTICIPANT 33:** Yes.

**PARTICIPANT 36:** And really connect with people no matter where they are or if they didn’t have computers or anything like that. So, I think they were pretty responsive.

**PARTICIPANT 18:** I personally didn’t have any contact. I don’t know if someone above me did in our team, but I didn’t have any reason to reach out or have any issues, so I don’t really have any feedback for it.

**AMELIA:** Okay. For those who did communicate with the team, are there ways that communication could have been better?

**PARTICIPANT 33:** I really feel that if the coordinators -- I can’t remember the titles -- had been in place to begin with, it would have cut down on some of the difficulty. I didn’t have, it wasn’t, it wasn’t extreme but there was some difficulty once we switched leadership. And so, I really feel that if the leadership had been in place at the beginning things -- the little minor things that I did run into -- they wouldn’t have happened.

**AMELIA:** Mm hmm. So, you spoke on that a little bit earlier but it sounds like maybe in addition to the leadership transition there were maybe at a later time some coordinators that were involved that weren’t involved initially? Am I hearing that right?

**PARTICIPANT 33:** Yes. But to get, I guess it’s like, it’s like a school, a new school getting ready to open. They hired a principal and the principal works about a year before the school opens. They don’t hire the teachers and then the principal, they hire the principal first and it goes down.

**AMELIA:** Mm hmm. Yeah, any other, anything else to add around that from other folks?

**PARTICIPANT 36:** I don’t have anything else to add to that one. I think she captured it pretty well.

**AMELIA:** Mm hmm, mm hmm. yeah. How would you, would you all participate in a program like this in the future? If there was a similar effort moving forward, would you consider participating?

**[01:10:00]**

**PARTICIPANT 33:** I would.

**PARTICIPANT 18:** Mm hmm.

**PARTICIPANT 36:** Yeah. I would as well. I can say at the core who I am is just really being an advocate in anything, any opportunity to do that, I would be willing to participate. So, the answer would be yes.

**PARTICIPANT 33:** Yes.

**AMELIA:** Mm hmm.

**PARTICIPANT 18:** We’re always looking to increase access to preventative and health related things certainly. Yeah, we’d love it. I still get calls all the time about if we have them.

**PARTICIPANT 36:** Yeah. Yeah.

**PARTICIPANT 18:** So, if there’s any that are out there or anybody has any monkeypox kits, too. We’ll take them.

**[OVERLAY]**

**PARTICIPANT 18:** Chlamydia, anything.

**AMELIA:** Yeah, so I guess anticipating that question. What do you feel like makes you -- you all said you would participate again, but what are the primary reasons that would motivate you? So, you mentioned advocacy and it just being part of your kind of core values. What do others think?

**PARTICIPANT 33:** Same thing.

**PARTICIPANT 18:** Increasing access. The more access people have and the less barriers they have to get to things the more successful you’re going to be. So, anything that helps broaden that access and gets things to people instead of having people have to seek things out is good. I mean, we find that with any health issue we have if the more we can get it to people through access the better we are. So, breaking those barriers down and making it less difficult for people to access things is our big thing.

**AMELIA:** Mm hmm. And is there anything that you, anything else you all want to share about the program that you haven’t already shared? Or any recommendations moving forward for programs like this in the future?

**PARTICIPANT 33:** I would like to see more programs like this where it increases the access, too. Whether it’s medical treatment or whatever, I like these kinds of programs because we do have people that don’t, can’t get to the site. I know that we feel that with transportation we should, and I think the easier we make it the better we can overcome pandemics and other kinds of health issues, situations that may occur in the future.

**PARTICIPANT 36:** Yep. Yep.

**AMELIA:** Mm hmm.

**PARTICIPANT 36:** Like the previous speaker said I think it’s important going forward that we maintain the infrastructure that we’ve already gotten in place. Keep those things in place because in the future there’ll be other things that will happen so, you don’t want to have to, have to go back and rebuild those things because that takes time. So, I think in the meantime even though there may not be a pandemic it’s very important those infrastructures are maintained, those communications are maintained. Those lines are always connected so they’re in place when we need them.

**PARTICIPANT 33:** Yes.

**AMELIA:** Mm hmm, yeah. Yeah. Anything else? Any words of wisdom for future program planners?

**PARTICIPANT 36:** I think it was a good response. Given the enormity of the issue I think the response was good. I think there some learning, some takeaways from that but I think overall once we got systems in place, we really prevented a lot of people from dying.

**PARTICIPANT 33:** Yes, most definitely.

**AMELIA:** Yeah, and you all previously spoke about some of the challenges around the distribution and kind of streamlining that. Are there other systems or pieces that you felt like were helpful once they were eventually up and running?

**PARTICIPANT 36:** I can’t, uh uh, I can’t think of anything else.

**AMELIA:** Okay.

**PARTICIPANT 33:** We talked about the media campaign.

**AMELIA:** Mm hmm, right.

**PARTICIPANT 33:** And everything. We talked about that, and it was good because

**[01:15:00]**

it was on social media, it was on television, it was on radio, it was in the newspaper. People were talking about it. It was, it was something that hit all kinds of avenues and so, I think that that was good as far as trying to get something out in a quick manner to help combat our slow spread of any kind of disease or whatever we have that’s going on in our nation.

**AMELIA:** Mm hmm, mm hmm. Yeah, that makes a lot of sense. And last question in our final four minutes. Do you feel like you encountered any challenges or barriers in implementing the program that were specific or related to your identity? So, your race, ethnicity, age, gender or sexual identity, language or where you were based?

**PARTICIPANT 33:** I don’t think the light is on with everybody about the severity of Covid. Later on, we had people in our community that I can put my hands on and I talked about that died of it. I don’t think that some people at the time that I was distributing the kits really, really felt that it was as serious as it, as it became to them.

**AMELIA:** Mm hmm.

**PARTICIPANT 33:** I don’t think that that was, that was there. And I think that, I don’t know, you tell somebody -- you tell them, you tell them, you tell them -- until they experience it that’s when they believe it. And we had people in our community that actually, you know, of course it got serious after a while. But at first, oh, I’m not going to take the shot. You know, that kind of an attitude. And rumors that were spread.

**PARTICIPANT 36:** Yep.

**PARTICIPANT 33:** I actually had to deal with one in my family. My youngest, my youngest daughter, works at Caswell in the Covid unit and someone told her that if she took the shot she would be, it would affect her fertility. And I had to get her to understand that if you’re fertile and you have a child and you get Covid, you’re going to die and leave the child. Do you want me to raise it? And that quickly [INDISCERNIBLE].

**PARTICIPANT 36:** Wow.

**PARTICIPANT 33:** I’m who I am and what I am, and I’m old school. Anyway, it was enough to motivate her to get the shots, so, that was good.

**PARTICIPANT 36:** Yeah. The only thing I would add to that is I really believe if you -- I know in our area we didn’t have the existing infrastructure in place, those existing groups in place I think we would have had some areas that wouldn’t have gotten the information to make decisions and things like that. So, I just, I just think that based on the past we had those systems in place so, that we could get that information, get the kits out to all areas within the community.

**AMELIA:** Mm hmm.

**PARTICIPANT 36:** And I think that would be one of my recommendations is really to continue to strengthen that.

**PARTICIPANT 33:** Okay.

**PARTICIPANT 36:** Because it won’t end with this. It will always occur, so, let our learnings be to make sure we have those things in place so, that we don’t leave anyone out. That we can reach everybody within our community.

**AMELIA:** Mm hmm. Mm hmm. Yeah, like someone else brought up, monkeypox and all.

**PARTICIPANT 36:** Yeah. Yes.

**AMELIA:** On to the next. Okay. Well, we’re about at our 90 minutes. Anything else to add that we haven’t talked about or that you’d like to share before we close out? Okay. Well, I really appreciate this conversation. It’s really hopeful for us and great to hear all of your perspectives and experiences with the program. So, that concludes our focus group. Thanks for coming and sharing your --

**[01:19:25]**
