# Supplementary material for: Exploring barriers and facilitators of implementing an at-home SARS-CoV-2 antigen self-testing intervention: The Rapid Acceleration of Diagnostics—Underserved Populations (RADx-UP) initiatives
Source: PLoS One. 2023 Nov 16;18(11):e0294458. doi: 10.1371/journal.pone.0294458 (PMC10653400; doi:10.1371/journal.pone.0294458)
Supplement: S1 Dataset — (ZIP) [file pone.0294458.s002.zip › NCFG-Transcription (9.21.22).doc]

**FACILITATOR:** Thank you, alright. I think your names will be changed to IDs very soon, so when I refer to you, I’ll be using your participant IDs. I know right now, your names are showing, but we’ll change that moving forward, and I think Interviewer will help with changing your names to a participant ID.

**INTERVIEWER:** Yes, so with that, we don't have participant IDs for some of you. Are you guys comfortable with your names being on the screens? Are you guys okay with that?

**PARTICIPANT 1:** Yes.

**PARTICIPANT 2:** Yes.

**INTERVIEWER:** Okay, thank you.

**FACILITATOR:** Okay, thank you very much. So let’s start with this session by talking about what makes your state or city a good place to live. What are some things that you enjoy about living where you live? I know you are all in the communities and very involved, so what do you enjoy about your communities or enjoy in that area?

**PARTICIPANT 2:** Well, we have access to a medical school, a recreation, a community college, a university, so that is very, very helpful to improve our quality of life, I think. Healthcare, and we have some employers that are providing income, livable income, yeah.

**FACILITATOR:** Thank you, thank you, and you live where?

**PARTICIPANT 2:** Pitt County, Greenville, North Carolina.

**FACILITATOR:** I thought so, just wanted to hear it. Anyone else, please, free to add.

**PARTICIPANT 1:** I would like to add – hello, can you hear me?

**FACILITATOR:** Yes, yes, please, go ahead.

**PARTICIPANT 1:** Okay, this is Participant 1. In addition to all the things that Ms. Huggins stated, I agree with all of those, and also I think we are the largest county in eastern North Carolina, so we are sort of looked up to by those on the outside, however some people think we’re the sleeping giant, but we have some awesome communities that surround Pitt County, and they utilize a lot of the services, and having worked in eastern North Carolina as a career, we have a lot of sprints in those smaller counties, and they do sort of look up to Greenville, but I’ve seen both sides of the picture, and I think that it’s good to know that we can work well together with our outerlying counties and provide them some services that they don't necessarily have as well. I think our education system here is doing a good job, however always room for improvement, and we are one of the largest systems in eastern North Carolina, and there is a lot of access to all the awesome opportunities we have, a Pitt-Greenville Convention Center that provides great services to our community, and so that's the end of my list, so Ann has told most of them that I thought of.

**FACILITATOR:** Don't forget the airport.

**PARTICIPANT 2:** Yeah, I was just getting ready to say, I forgot the airport in our community.

**PARTICIPANT 1:** Yes, um-hum, and the rural aspect of it, I like the rural urban parts, because I grew up in the country, back up in the woods, and today I could relate to all of it.

**FACILITATOR:** Wonderful, wonderful, thank you very much for that rich response. The next question is what are some things that are not so good about where you live, that you think, at least? If everything is good, [INDISCERNIBLE]? You don't need to make things up, if you are very happy and you don't think there’s nothing that's not so good, that's fine.

**[00:05:00]**

**PARTICIPANT 2:** Well, as Mil just said, there’s always room for improvement in our law enforcement. There’s room for improvement even in the education system, in the school system, we do have some schools that are failing, so there’s always room for improvement. I think we have a lot of groups that work well together, so I think partnership is a strength of Pitt County, being able to form partnerships.

**FACILITATOR:** Thank you, thank you. Now I want you to think about how your county responded to COVID-19, thinking about your local response to COVID. Were you satisfied with this response to COVID-19?

**PARTICIPANT 2:** Well, being – I don't want to be – somebody else say something.

**FACILITATOR:** It’s okay, I will follow up with them. You can go ahead and then I’ll go there.

**PARTICIPANT 2:** Oh well then, one of the commissioners on the County Board of Commissioners, and of course the Health Department falls under the County Commissioners, we were able to ask the Health Director to set up testing sites across the county, as well as giving the vaccinations too, and as we distributed the home test kits, that was very acceptable across the county, and I think we did a great job in covering our county with the home testing kits, as I said, the Health Department, the hospital and all, so we had a good partner. The partnership there was good, I think.

**FACILITATOR:** Thank you, thank you. Anyone else?

**PARTICIPANT 2:** You muted Mildred.

**PARTICIPANT 1:** In addition to that, we do have 10 municipalities in Pitt County, so we were able to organize and make sure all our entire country was covered, and we had good partners from the faith-based community to public housing, the educational system, the city hall, lots of lay organizations, the black and brown communities worked, the Latino, the Black community, and others worked with us. And so we really did do I think a [INDISCERNIBLE]’s job in a short time, connecting all of those elements, and it was sort of a unique part of it because we were able to connect with our elected leaders who were supposed to be the other policymakers of those towns, and so we really targeted them and we think they did a good job working with the lay communities as well as professionals and lay people, and that helped, and I think we have some friends forever out there. We worked with the veterans’ groups. There was a whole long list of people that we connected, even the food trucks connected in, so we tried to make it really true local, and it seemed to have been good.

**FACILITATOR:** Thank you, thank you. We’ll call on Participant 3, do you have anything to share? You’re muted, Participant 3.

**PARTICIPANT 1:** Unmute, Participant 3.

**PARTICIPANT 3:** I’m working on it, alright. Good afternoon, to everyone. Having worked at my church with Participant 5, Mildred, and all the others that worked at Holly Hill Original Free Will Baptist Church

**[00:10:00]**

in Belvoir, and having had the opportunity to pick up the first batch of vaccines that arrived at the post office and deliver them at the church, at our Life Center, then being one of those that were responsible for helping to store and make sure that the vaccines were – I mean the test kits, I’ve worked with vaccines all my life – but the test kits were readily available and separated out, and when people came to pick up their products, then it was a simple process. We already had them counted out and waiting for them to come pick them up and deliver to their various areas. I thought this program in Pitt County really went over well, because a lot of people were involved and a lot of people did an awesome job of making sure that everything worked out fine, so logistically at our church, we were able to make sure that our products were readily available when people came to pick them up to go back to their particular areas.

**FACILITATOR:** Thank you very much, thank you very much. Anyone else, James, would you like to say anything?

**PARTICIPANT 5:** Yes. Our church is located in the rural area of the county, what we call north of the river, and we were a warehouse where we used to distribute test kits to other municipalities that would come and get test kits and go back to their communities and distribute them, and so we were in an area where we could assist other agencies in north of the river, and one important factor that I like about it is that it originally was planned to be in Greenville, but we included the rural area of Pitt County, and Mildred and others spoke very highly about that, because we have so many people that were not going to be reached because of their location in the county, but by all of us working together, city and county, it went over beautifully in Pitt County.

**FACILITATOR:** Thank you very much. Participant 4? You are muted.

**PARTICIPANT 4:** Can you hear me now?

**FACILITATOR:** Yes.

**PARTICIPANT 4:** I’m sorry about that. I’m the Commander of American Legion Post 160 in Greenville, North Carolina, and we were so glad to participate in this program because of location, and we have veterans that not only come from Pitt County but outlying counties, so we were acting as a distribution point, and we tried to concentrate on like breakfast, lunchtime, and dinner for people to pick them up, and we also had some of our veterans that would take them to the southern part of Pitt County in the Grifton and Ayden area because they lived there and they had churches there that they had some of the kits there, so we did that, and not only did we do that. Afterwards in April of last year, we had a medical or health fair, we teamed with Vidant Medical Center, and not only did we have 66 vaccinations, but we participated in a health screening like for high blood pressure, cancer, and those type things, so all in all, I think we did a great job and we worked with all of the people that went before me, really good job.

**FACILITATOR:** Thank you, thank you very much. Very rich responses. How do you think that your county’s COVID response compared to what was happening nationally? What were some differences or similarities? Can we start with [INDISCERNIBLE]?

**PARTICIPANT 3:** [INDISCERNIBLE] Pastor of Holly Hill, also Chairman of the Pitt County Board of Education. I would say that the people that we were working with here in Pitt County really worked very well together. What I heard from others, they did not have the collaboration that we have here in Pitt County, which was a plus for us that we had organizations that stepped up to the plate and people that sacrificed their time to come and participate in this. Our participation was great, and I think that's what stood out the most with us,

**[00:15:00]**

that we had people willing to come together for one common goal, and that was to get the kits into people’s hands.

**FACILITATOR:** Thank you, thank you.

**PARTICIPANT 1:** And I think wasn’t North Carolina, we were the pilot program for the home testing kit, I believe, weren’t we?

**INTERVIEWER:** Right, yeah.

**PARTICIPANT 1:** Yeah, so I think we did a great job in piloting the program across the county, yes.

**PARTICIPANT 2:** And across the nation, it was the country to do it, [INDISCERNIBLE].

**FACILITATOR:** Yes, we’ll get to the project, but this question is just about the general response before let’s say this COVID project came into your county, like the response the county had before the project, how was that response similar or different to what was happening in the country before this COVID project came into the picture?

**PARTICIPANT 2:** Well, as I said, I think partnerships were important because – oh, are you asking about testing or not testing?

**FACILITATOR:** No, no, I’m asking about in general, like the COVID response, how did Department of Health or organizations respond to the pandemic in general in your county, that response, how does that compare to what was happening nationally, based on when you were looking at the news, did you see some similarities between what was going on in your community and other places in the U.S., or some differences?

**PARTICIPANT 2:** Well, some similarities, but Pitt County kind of led, I think we kind of took the lead on some things like mask requirement and shutting down and all of that, where some of the others were still open, so I think maybe we got ahead of some of it, but I think that was important. Of course, we caught some flack from those other people about shutting down, because they wanted to talk about the economy, so they wanted to keep the stores and all that open, when we said we need a shutdown and then require masks. Yeah, some of that we kind of took the lead on, I think, for North Carolina.

**FACILITATOR:** Thank you. Now I want you to think about your perceptions when your organization was contacted by someone about the Say Yes COVID-19 project, the Say Yes COVID test project. What did you think about that initial communication when you were first contacted for the Say Yes COVID test project?

**PARTICIPANT 1:** Well, I was the contact for Pitt County, I got a call from a friend of mine out of Raleigh that was working with Al Richmond, who is with the Community-Campus Partnerships, and he met with me and said that he had written this grant through National Institutes of Health, and he wanted to know if I could pull together a focus group so he could tell us that Pitt County was very high in incidence of COVID-19 in the wastewater, and that he had an opportunity to help us with that by using those kits here in Pitt County, or they could go somewhere else to do it, and of course, we were concerned, especially at my church, my pastor was very concerned about his members and the surrounding communities and everything, so I called him and so many people on this same call, because I wanted a group of people that I felt were real, that could really represent many aspects of this community to be in the focus group, and he wanted it done like yesterday, but he called us, I’m like wait a minute, and so anyway, but fortunately, we were able to pull together – because I think they asked for at least five people, and I always believe in going a little step further, because sometimes people do drop out – so I think I organized nine people,

**[00:20:00]**

and we still had one to drop out, so we had a really good mixed group of faithful people in our community from all walks of life, representing many different organizations, from faith-based to the veterans groups to our leaders or the whole county, the County Commission, and also we had some lay and professional civic type clubs that were engaged as well, so we tried to really mix it up and I felt that this would be a good group. I contacted them, and they all agreed, and so the rest is history.

**FACILITATOR:** Thank you very much for that rich response, Participant 1. What I would like to ask you to share a bit more was your perception of when your friend contacted you, what did you think about the way she communicated to you about the project itself? What were your thoughts? Were you positive, negative, or anything, neutral?

**PARTICIPANT 1:** It was positive because I felt that this gentleman, I had met out somewhere in my network working throughout the state with various organizations, and I couldn't remember at the time where it was, and I knew of his work in the state, and I trusted his [INDISCERNIBLE], and I even trust more the person that referred him to us, so I felt like this would be a good opportunity for us to be engaged, and when he explained to us that this is something to help your community, and we need people that we can count on if you all would like to do good, so I felt confident that we had a real friend, a professional that knew what he was doing and that would lead us in a good direction, so I was positive, I was excited, and it gave us something to do to truly help the most vulnerable populations in the nation, really, because we were struggling like everybody else.

**FACILITATOR:** Thank you, thank you. Now, since you say you contacted the other members on this call, I’ll ask then maybe Participant 4, what did you think of the communication you received from Mildred?

**PARTICIPANT 4:** Well, I was elated, because being a veteran, we are very much community focused, not only when we was in the military, but when we came back, we love working in the community and doing things for the community, and this covered two of our pillars. We have four pillars of things we do in the community. It covers like for instance pillar four was children and youth, but it covers immunization, and it covered the test kits. Pillar one was for veteran affairs and rehabilitation, which cover our volunteer and outgoing into the community servicing groups, so we were very elated.

**FACILITATOR:** Thank you, thank you, and what did you think about the communication itself? What did Ms. Mildred say to you, or Ms. Council say to you?

**PARTICIPANT 4:** Well, first she wanted to see were we interested, and she knew that we had a reputation of getting out and getting the job done, so it was a good go, go-go-go.

**FACILITATOR:** Thank you, and anyone else? Participant 3, what did you think of the communication?

**PARTICIPANT 3:** Well, having spent 27 years in vaccine-preventable diseases, for me it was a no-brainer. I mean I covered as many as 25 of the 100 counties in North Carolina at one time, did it with vaccines, and so when you have a situation like this wherein you were testing people to see what their status was, it made nothing but sense to me to get out there and push it as hard as possible, and that's pretty much what we did, and if you look at the people that you’re looking at on the screen right now, all of us have rich backgrounds in public work, every one of us, from law enforcement, social workers, public health, so for me, it was the way to go because I was looking at protecting individuals from a disease that we did not know how devastating it was going to be, so for me, it was awesome, and the information that I was getting from none other than Mildred – and I got Betty every single day on a regular basis – so you know, it worked well with me.

**[00:25:01]**

**FACILITATOR:** Thank you, thank you, I appreciate your answers. The next question is related to what you already shared, but I have to ask you, and maybe someone else can respond. The question is how, if at all, did the process or idea of working with a community group that brought together several community leaders influence your decision to participate in any way? I’ll start with you, Ann, since you didn't get a chance to respond to the previous question, but it’s a chance to do so.

**PARTICIPANT 2:** Well first, when Mildred called me to tell me that she had this friend that had this program that he wanted to bring to Pitt County, of course I had questions and I said, “Okay, how are the people going to know how to test? How are they going to get where the results are going?” Oh, I just had all kinds of questions, because I am used to people coming up with these programs and they are not always in the best interest of us as African Americans, but her confidence was in the person that was bringing the information, so I was willing to listen. So in doing that, now the other part of the question, I forgot now what you wanted me to answer.

**FACILITATOR:** Oh, the question is how did the idea of working with other community leaders influence your decision to participate?

**PARTICIPANT 2:** Oh, naturally, yes, it did, because as Mildred said, she reached across the county and invited people to listen to this presentation and get involved, so that's Mildred. She is going to do that, and I will do the same thing. If an idea or something comes to me, I am going to reach out to other people that I know who are like minds and let them hear, present it to them, see what they think about it, bounce it off of them, so yes, I was all for working with these groups and getting it done.

**FACILITATOR:** Thank you, thank you. How about you, James?

**PARTICIPANT 5:** Yes, I would say that the idea of working with people across the county – Mildred, being her pastor, I could not say no, she was going to involve me anyway, but the thing was everybody that participated did not just participate protecting their own interests or their own areas. It was a group thing, and my thing was to see people across the county come together, because it started out initially they tested Greenville water, and you know that a lot of people work in Greenville, travel to Greenville, and they were testing the water, and they were not including the rural areas, but when Mildred said we are going to include the rural areas, no one said it’s going to just be here, but it was actually all over, and that's one of the things that I was impressed with, that everybody had an opportunity to get free test kits, and especially those in the rural areas.

**FACILITATOR:** Thank you, thank you, so anyone else would like to add a response before the next question?

**PARTICIPANT 2:** Let me add to what Participant 5 just said, he reminded me when he said at first it was going to be tested just in Greenville.

**FACILITATOR:** Before you do that, because the question is about the community leaders, is it related to community leaders or the rural?

**PARTICIPANT 2:** The rural, because it was kind of comical when they said we’re just going to test the water, I mean the sewage water in Greenville, so we said, “Well, why not the county too?” Well, the answer was that people at some point in the county was going to come --

**[00:30:00]**

**PARTICIPANT 5:** -- to Greenville.

**PARTICIPANT 2:** [INDISCERNIBLE] to be in Greenville.

**PARTICIPANT 1:** Because they have to come here to work, to play, get groceries, use our medical systems, and all of that, so they were all responsible for the COVID [INDISCERNIBLE].

**PARTICIPANT 2:** That was the answer, but then Mildred didn't take that for the answer, she wanted the whole county to get those kits.

**FACILITATOR:** That's the way a great leader should do it, should want everyone in the community to benefit. Thank you.

**PARTICIPANT 5:** And sometimes, after just trying to pull leaders like that together, some are very protective and they protect their own areas, but I would say these leaders actually came together for the cause of all people.

**FACILITATOR:** Thank you very much, James, that's very important.

**PARTICIPANT 5:** You too.

**FACILITATOR:** It’s good to have selfless leaders like those who were involved in the project. The next question is how, if at all, was the Say Yes COVID test project responsive to the needs in your county related to COVID-19? And I am sure you have a lot more to share, as you already started doing that in the beginning, about how the project was able to help address all the needs in the community, so please share. Maybe we’ll start with you, Participant 3.

**PARTICIPANT 3:** Well, again, I would say that with all of this being new to everyone, and being a pilot county in the entire country, one of two, there was some apprehension, and I think Ms. Huggins mentioned a minute ago, how are we going to do this with these test kits, and how are we going to do that with these test kits, and the negativism that would come from it, but I think the process, and the process is that before the kits were ever really introduced to the overall public, we got a working knowledge of how to do the testing and the best way to present it, and so when it was done, it was done very professionally and very precise, and tried to remove all the fears, that that test was not going to jump out and bite you, it was going to protect you once you were able to go in and actually do those tests, so we were able to feed them the best possible knowledge in the best possible way, in the shortest time possible.

**FACILITATOR:** Thank you, thank you. Anyone else? How did the Say Yes COVID test project respond to the needs, if at all, in your community?

**PARTICIPANT 4:** Well, I concur with Participant 3. He came to our American Legion building, and as we were passing out the kits, we gave them instructions on how to use the kits, and believe it or not, the general public said they were very elated because they never knew the American Legion being anything other than party, so it was a win-win situation where they saw us in a different light, and now it did help us pick up on our recruitment, we have found new membership, so it was a win-win situation.

**FACILITATOR:** Thank you, thank you. Anyone else before the next question?

**PARTICIPANT 5:** I will say at first, it was kind of slow starting because people were apprehensive about it, and how to use the test kit, but once they got to rolling, it went very well.

**FACILITATOR:** Thank you, thank you, and I think what some of you have mentioned relates to the next question, which is what are some of the challenges or barriers that you experienced with the Say Yes COVID test project, and I think earlier, one of the issues was the trust, the lack of trust in the project in the beginning, but what were some of the challenges or barriers you faced besides maybe being slow to start, as James just mentioned?

**PARTICIPANT 1:** Well, one of the things for me is that because our target, Al told us that it’s for everybody, but usually when a project of this magnitude comes in a community, usually the most marginalized people are left out, and that we want you all to push hard

**[00:35:00]**

to make sure that the marginalized individuals, the black and brown community, get access to these kits, and anyone else that want them, so we all went to trying to attract everybody. Well, I went to my nail salon, and the guy looked at me like, “Well, what is this, what is this” and everything, and so I told him what it was, and of course his wife wanted it, but now here I am spending my money with him every so often, but I didn't feel that he trusted me, and later on when the test kits ran out, you could not find them, even the drugstores didn't have them. I went in one day, he said, “Ms. Council, Ms. Council, Ms. Council, tell me, I need some kits, I need some kits,” and I looked at him, he was really hot, he said, “Oh, I know, Ms. Council, I know, I know, you brought me some and I didn't take them.” I said, “You want some now?” I was straight up with him. He said, “Please. Ms. Council, please, please, please. I need some because I think some of my nail techs got COVID, I just got to test them.” I said, “Well, I cannot help you, sir, right now because the program is over.” “But don't you have one?” I said, “I do, I have my personal kit.” “Well, could you bring it back and share it with me?” I said, “Well, how can I do that when the kit only has a tube of solution and a test thing,” I said, “That's like your toothbrush.” “Well, Ms. Council, just please bring it by and just pour some in a --,” I mean he was furious, and so I said to him --

**FACILITATOR:** [INDISCERNIBLE]

**PARTICIPANT 1:** So, we went to barber shops, beauty shops, distributing them. I said, “Well, maybe some of my fellow beauty shops and all of them,” so I thought of one that had gotten quite a few, because they have a really big population of customers, so I called them, couldn't get no answer, so when I called the smallest salon way out in the county, she said, “Ms. Council, I have three left, and come get mine,” and so I did, and we gave it to him, and he was like, “I apologize, Ms. Council, I apologize that I didn't do what I could have done back, because you brought some for everybody.” I said, “Well, that's what happens, we are out here trying to help everybody.” So that was a real interesting story that I experienced, and I said, “Right down the street at the cleaners,” this was an Asian nail tech place, I said, “The cleaners accepted them, they wanted some for everybody at the cleaners.” I think they had nine staff members, and so that was one of the issues I faced, trying to help everybody, but at the end of the day, they learned that we were there for everybody.

**FACILITATOR:** So, the problem you had was not having enough kits to give to people during the program?

**PARTICIPANT 1:** No, the problem was that he didn't trust me for the kits. I guess he thought, you know, he didn't trust me at first, wouldn't take any, and his wife took one anyway, and of course they used it later, but later on when there were no kits left, we could hardly find any because the program had ended, he then comes to me for help.

**FACILITATOR:** So, the problem during the program, before the program ended, was that he didn't trust you at first, that was the problem.

**PARTICIPANT 1:** That's right, that was the problem.

**FACILITATOR:** That was the problem, okay, perfect. Anyone else? Participant 3, any problems you faced during the program, not after it ended but during the program?

**PARTICIPANT 3:** No, not really, because my main function was to make sure that the ktis were distributed according to the needs of the areas that our church served north of the river, and just really staying up with the number of kits that we had so that they were distributed fairly, so not really. I did not have any issues from my point of view, from my standpoint.

**FACILITATOR:** Okay, no problems.

**PARTICIPANT 5:** I’ll say this. In the beginning, we did have a delivery issue on the way that they were being delivered, and then once we got that straight, the deliveries came better, because some were being dropped off at the church, then there were some we were looking for that were being dropped off at the post office, so we had to go into Greenville, get them from the post office, go back out into the community, but once we addressed how they were being delivered, then they were being delivered in a proper manner.

**FACILITATOR:** And the issue with delivery was that they were not being delivered in the same location or they were not being delivered on time or what was the issue?

**PARTICIPANT 5:** They were being delivered, some were being dropped off at the church,

**[00:40:00]**

what we experienced, and then there were others that were dropped off at the post office, and we were not aware that they were at the post office.

**FACILITATOR:** Okay, so at the post office, so not knowing, and then you fixed that.

**PARTICIPANT 2:** Yeah, FedEx would give you a time of the delivery, and it could be 2 o’clock in the morning, you know.

**FACILITATOR:** That could be a problem for anyone.

**PARTICIPANT 1:** That could be a problem, okay. Nobody was going to be at my church at 2 o’clock in the morning, but when I went to my church, the boxes were stacked up there at the door.

**FACILITATOR:** Ah, thank you, thank you.

**PARTICIPANT 2:** And the way that was resolved, we had a gentleman that worked with Churches United, I think it’s called, across our county, and they have a warehouse, so they regrouped and used that as the main distribution center, however the individual churches could pick some up and still deliver to their closest individuals or organizations that needed them.

**FACILITATOR:** Thank you, thank you. Now the next question, if no one else has something to add for the challenge question, is what helped all of you to accomplish the goals of the project, of distributing kits to so many people? What helped? You mentioned the trust issue to begin with, and then eventually the distribution, the delivery as an issue, but what were some of the things that worked to help you make the project a success?

**PARTICIPANT 3:** Social media.

**FACILITATOR:** A committee?

**PARTICIPANT 3:** No, social media. We used Facebook a lot, and we would show people coming to get kits and encouraging people to come out and get kits, so social media helped a lot in getting the word out, where the locations were and when they could pick them up.

**FACILITATOR:** Social media, ah, thank you, thank you.

**PARTICIPANT 1:** And we even went on to some of the social media outlets to advertise the kits. We used our local black radio station. We have a group out of Wilmington that the paper reaches this area, and so they interviewed some of our members to ask those same kinds of questions and find out how we were reaching out with this brand new project in the area, so that was good as well, and then there was a lot of marketing on billboard signs throughout the community, and of course they used the newspapers some, so we had all kinds of ways of reaching out. They even used some of us on the posters so that people would know who were helping to get the job done, and I think that was effective because they knew you and they felt a little more comfortable, if they didn't at first. And we had some great organizations like the National Council of Negro Women, that really just took charge. They just said one day that wherever the need is, just send us, we’ll go anywhere in Pitt County you need for us to have a popup, giveaway, and they were just awesome ladies to help do that, the black social workers, the alumni association groups, HBCU Coalition, they helped, the sororities, fraternities, they got engaged, so we really tried to cover as many different entities as we could, and that was effective.

**FACILITATOR:** Thank you.

**PARTICIPANT 2:** Also, Mildred, the Committee, Al and Betty, we would have general meetings or whatever to keep in contact in that way too.

**PARTICIPANT 1:** Oh yeah, we had a good supporting network, that's true, I forgot, that was really good. They really kept us on task, and we could ask them any questions that we wanted to and everything, so that was really good to have a team that was sort of overseeing the project, because we were brand new at this, we had never done anything like this before, and so they were very, very effective.

**FACILITATOR:** Thank you. Anyone else?

**PARTICIPANT 4:** Yeah, Participant 4 [INDISCERNIBLE] again.

**[00:45:00]**

We have a working relationship with a couple of more organizations, like for instance Department of Public Safety, we work with them, and they helped us get the word out, and the [INDISCERNIBLE] Black Men in eastern North Carolina helped us get the word out too.

**FACILITATOR:** Thank you, thank you.

**PARTICIPANT 1:** And the food trucks were the most interesting one, I thought, because one day I was like desperate in getting – I set my own personal goal, even though I was like community organizer of the project, advocate, and all of us on this call, for the most part, were spokespersons for the project, and one day I was at a grocery store and I saw this food truck and people lined up, so I went up and got some permission from the operator of that food truck to see if we could give these kits to their customers, and they said, “Oh yes.” Then later on, the lady called me and said, “We’re getting ready to have a food truck celebration in Ayden, North Carolina, and could you come and be there,” because what this food truck did, every year they have a customer appreciation, and then they invite the other food trucks in the area to join in with them, and so they said it would be in the park for about two hours, two to three hours, they said, and “if you all could come and help give these to our customers that we are appreciating, that would be helpful.” So we went to a food truck celebration in Ayden, and that was very unique and different, and they provided free food for everybody, and their other neighboring food truck operators joined in to support this one food truck group, so that was different.

**FACILITATOR:** Thank you.

**PARTICIPANT 1:** And then out of that, one of the food trucks was a pastor of a church there in Ayden, so she asked me to come during their food giveaway to just be there when the people pick up their food, because they serve the community like twice a week, and so I showed up for that, so that was different, and I got a chance that time to see a lot of poor people that I never experienced before, because when they talk about it, it’s always people of color that are very poor, but that particular day I saw a lot of poor white people that I had not seen that was needing food, and so it was a new experience for me personally that day to see all these real, real poor people coming that had very little and needed help just to eat.

**FACILITATOR:** Thank you, thank you. I understand your experience can be very shocking sometimes with different people in need that you may not think are in need. So now before I move to the next question, are there anything else that helped to accomplish the objectives for the project that you didn't mention that you want to share now? Anyone else?

**PARTICIPANT 5:** Those that came and volunteered to help us distribute, they were very excited about it. Some of them took it like it was a job. Some are retired, they weren’t doing anything, and they came out and they helped tremendously. The volunteers that showed up were great, they really helped us out, to have people to man command what we were doing, those [PH] spaces.

**FACILITATOR:** Thank you, thank you. Anything else? It’s very important to know what helped you to be so successful.

**PARTICIPANT 3:** Well, on what Participant 5 was just saying, once we set up a schedule to distribute these kits, and using social media, as has been mentioned before, then the individuals were able to come, they knew that we were going to be there, as he just said, volunteering, so they knew exactly when to come, how long we were going to be open, and we would always have plenty of kits available so they could just drive up, get the number of kits that they needed, and keep right on moving, and it worked very, very well, and I think that was done throughout the county.

**FACILITATOR:** Thank you, thank you.

**PARTICIPANT 1:** Let me go back to the very beginning, when we first got the kits and started putting the kits out in the county, I encountered a few medical people, African American I will say, that were

**[00:50:00]**

hesitant about participating in this, and so I had to say, “Well, our Health Director is supporting this,” and this was before the Health Director came out with their announcement about the home testing kits, because when we were first approached, we were not supposed to – we were not to say anything at first until the announcement got made, supposedly been coming from the President, but yes, there was some hesitation from some of the medical people about the home testing kits, and I had to say, “Well, our Health Director is supporting this.”

**FACILITATOR:** So this sounds like it was a challenge, right?

**PARTICIPANT 1:** Yes.

**FACILITATOR:** Not what was helping to make it successful.

**PARTICIPANT 1:** Yes.

**FACILITATOR:** Okay, so that was another challenge you faced.

**PARTICIPANT 1:** Yeah, yeah.

**FACILITATOR:** Thank you. Now the next question is what do you think was the biggest factor driving county residents to pick up COVID-19 testing kits? We can start with James.

**PARTICIPANT 5:** What was the question again?

**FACILITATOR:** What was the biggest factor, what was the biggest reason that made people in the community come get the kits?

**PARTICIPANT 5:** I think when families started having gatherings or going to different things, and families were getting COVID two to three at a time, and people decided then that they needed those test kits when so many people started getting COVID.

**FACILITATOR:** Thank you, thank you. Participant 4?

**PARTICIPANT 4:** Well, once they became educated, we educated them or helped the people who educated them, then they knew it was legitimate, and they did come in very well then, and like you said, it’s been mentioned before, once they found out our hours of operation, it was good, because we tried to operate around the time they went to work, lunchtime, and the time they came home.

**FACILITATOR:** Okay, thank you, thank you. Participant 3?

**PARTICIPANT 3:** I would concur with both the last two statements, and that is when people really began to contract the COVID disease, COVID-19, people realized that their saving grace was to be able to be tested, and those kits began to run off the shelves.

**FACILITATOR:** Thank you, thank you. Mildred?

**PARTICIPANT 1:** Well, I ditto all that's been stated.

**FACILITATOR:** Okay, perfect, perfect. Ann?

**PARTICIPANT 2:** I do want to share one story with you, like Mildred had a story a while ago, I forgot this one.

**FACILITATOR:** Oh, before you continue, is it about this question, because we have a lot more questions that we need to get to?

**PARTICIPANT 2:** It’s about distribution of the kits, and the validity or being accepted, but you can go on to your next question. I’ll hold it, if you want me to.

**FACILITATOR:** Oh no, go ahead. I just wanted to make sure it’s related to what was making people come to collect the kits. As long as it’s related to that, I will be happy to hear.

**PARTICIPANT 2:** Well, I got a call, because my church was a distribution center, and our church number was listed as a place to pick up, so I got a call on a Saturday morning, I think it was Saturday morning or Saturday afternoon, one, from this doctor with the [INDISCERNIBLE], and she was one of the doctors with the communicable disease division, and she called and said that she wanted to get a kit. I said, “Well, we’re not open,” and she said, “Well, I’m going to a gathering, and I want to test before I go to this gathering,” so I said, “Well, alright, I will meet you at the church and give you a kit.” So, I met her at the church, and she said, “Can I give you some money for it?” I said, “No, you can’t give me any money for it, it’s free,” and she said, “Well, can I give a donation to your church?” I said, “Well, I’ll take the donation for my church.”

**FACILITATOR:** Definitely. Thank you, thank you. So, you said she wanted the kit because she wanted to test before going to this gathering, I think somebody mentioned that that was another reason people wanted the kits.

**PARTICIPANT 2:** Yeah, I mean but this was a doctor, okay, this was a doctor.

**FACILITATOR:** Yeah, well, doctors, I think we all want to get tested. Maybe [INDISCERNIBLE].

**PARTICIPANT 2:** Yeah, I mean a medical doctor, [INDISCERNIBLE], so she wanted to test before she went to that gathering.

**FACILITATOR:** And one could argue that the medical doctor needs it more, because they are probably interacting with a lot of patients and can get COVID, so she didn't want to maybe go and give it to other people, so even more important in a way – not important because she was a doctor, but because of the risk that she had being in the frontline, but thank you. So, the next question was what do you think was the biggest reasons – oh, I think it’s really related – that county residents were interested in knowing their COVID-19 status, so it’s related to what you all already shared, and if you have anything else to add, you can, but if not, it’s okay.

**PARTICIPANT 2:** Say it again?

**FACILITATOR:** Oh, what do you think was the reason that people wanted to know their status, and I think the story you shared is related to this question, like why do you think that doctor wanted to know her status for COVID-19? Was it because she wanted to be safeguarding family, occupation, her occupation as a medical doctor?

**PARTICIPANT 2:** Exactly.

**FACILITATOR:** Any other reasons you think?

**PARTICIPANT 2:** Because family members were dying, and friends were dying.

**PARTICIPANT 1:** Ann had a brother and sister to die within a week apart at your church, didn't you?

**PARTICIPANT 2:** Um-hum, yes.

**FACILITATOR:** Wow, so you think they wanted to know so that they wouldn't die or give it to someone else, because knowing people are dying may be definitely very devastating, but it may actually create some people who do not want to test, so just to be more specific, was it because they wanted to protect family members or protect themselves? What were some of the discussions?

**PARTICIPANT 1:** I think both, I think both, um-hum.

**PARTICIPANT 2:** I agree.

**PARTICIPANT 5:** I think too, for the African American community, some of them were being in the thought that it was like a [INDISCERNIBLE] thing, and they were hesitant about it, and once they found out that this was legit, that we were really trying to help people safeguard themselves from COVID by giving them free test kits, they got over that barrier.

**PARTICIPANT 2:** Um-hum, and something else that was brought to my attention when I was speaking about it to I’m going to say the very knowledgeable and very learned people, along the same lines as Bishop just said, he wanted to know, okay, people come into our communities and do these tests, do these results, and we never hear the results or the final outcome of it. That was a very concern that was brought to my attention. Mildred, I think you heard that too?

**PARTICIPANT 1:** Oh yes, I did, definitely, and that's the part that sort of impressed me, because this team that's just sitting here today, and there are several others that helped too, we felt empowered that we were helping lead the process as a team, and that helped make our jobs a little easier, and that they knew that they could trust us to do this work.

**FACILITATOR:** Thank you, thank you, that's very helpful to know. It is a lot of work, and dealing with people, I can understand it’s not easy. The next question is related to what you mentioned earlier in the focus group, and the question is what measures, if any, were taken to ensure the needs of all county residents were equally considered for the Say Yes COVID Test Project,

**[01:00:01]**

and just to say what I heard earlier was that I believe one person said, Mildred said that she wants everyone to be tested, to have access to those kits, or to be included, so that's one of the measures, I believe, right, that Mildred spoke out and advocated in the beginning for the project. Were there any other measures, or you can elaborate a bit more about that statement you made earlier?

**PARTICIPANT 1:** When we took the test to all – we went to like Walmart shopping center, grocery stores, where the people were, anybody that wanted one could pick up one, and like I said, some of our support groups, those organizations and all, they were out there asking everyone that came by that exhibit table to please take a kit, so it was everybody that they were shooting for, however we wanted to make sure that the most marginalized communities were not left out, and so we just went everywhere that we thought people showed up – industry, banks called. In Ayden one day, I was over in Ayden, and the Southern Bank called, they wanted to know if they could have one for all their staff members, and they were a predominantly white bank, and so we took them, and the grocery stores, you name it, those kits was going out all different directions and everything, but by the time that we had gotten into that phase of it, we had really made sure that the most marginalized community had an opportunity to be engaged too, because we wanted to make sure they were safe, that we didn't discriminate against anybody.

**FACILITATOR:** Thank you, thank you.

**PARTICIPANT 5:** We especially made sure that the Hispanic population was also given an opportunity to get test kits.

**PARTICIPANT 1:** Um-hum, and they were on the team too, the Hispanic people were part of it, and what they did, they wanted kits when they would have their little festivals and things going on, and so the kits were distributed when they would be having social gatherings and all of that, and then they even asked for support to bring the people out, so there was a component of folk working directly with them who spoke Spanish and all of that, so they were right in with what was going on. They were connected.

**FACILITATOR:** So inclusive, inclusion of different groups.

**PARTICIPANT 1:** Oh yes.

**FACILITATOR:** For distribution, and having multiple, you know, going to multiple places to distribute and trying to reach everybody was the measure.

**PARTICIPANT 1:** Yes, that's what we did, exactly, um-hum.

**FACILITATOR:** Thank you, thank you. Very, very good work, very good work. Now thinking about your exposures to health and COVID information in your community, did the Say Yes COVID Test Project help you identify credible sources of information that you were not yet aware of before the project?

**PARTICIPANT 1:** Not for me, it did not totally, because I am a retired public health professional, so I worked 32 years with the State of North Carolina, and I worked with every aspect of public health. In our offices we had doctors, nurses, therapists, social workers, all kinds of folk, and we were tied into a state-wide network, and we met periodically with the families, maternal and child health, infant mortality, [INDISCERNIBLE], you name it, every aspect of health which still today exists with the North Carolina Department of Health and Human Services, so we were exposed to all of that, and so I wasn’t as surprised with that because I sort of knew how that system worked, and was having all of these different – like for instance with the American Legion, one day we just went on a tour to see what was happening across Pitt County, because we wanted every aspect of Pitt County to showcase. That one day, we started out in Fountain with the Mayor, and she was giving out kits like at little a drive-through. We left there, came into Greenville, went to Koinonia Church, that's one of the largest churches in the city. Her church was having not just kits,

**[01:05:00]**

but they had medicine for people, they were giving out our tests, they had insurance companies, anything that families needed, food, they were doing it all. Then we left there, went on over the American Legion building. They were having screening of all different types, and in the process of going there, we stopped by Holly Hill, of course, and Holly Hill was having a big to-do that day. They had brought in the vaccination piece by this point, so they had vaccinations going on, so that day, that was a huge, magnificent, service delivery for people just to come up, it was like a big health fair, and actually the American Legion I think that particular day, there was competition between them and Koinonia Church, because they had a whole lot of stuff going on. They even had the economic group out of Raleigh showed up, I’m trying to think of the name of them, so they was coming there to give services to the people, anything --

**PARTICIPANT 5:** Let me help you, [INDISCERNIBLE] from Raleigh, and [INDISCERNIBLE 01:06:11] from North Carolina, yeah.

**PARTICIPANT 1:** Okay, so they were there, and they were just excited. People wanted to take our kits to them, so “Wait a minute, these are supposed to be for Pitt County,” so anyway, we had a showcase that day of services in Pitt County, North Carolina, and there were other days that same kinds of things went on too, so it definitely exposed us to the people, to what you can get, because people were struggling, and even the food people, like the group out of [PH] Koinonia brought in food to give away, helping people here in Pitt County, so all kinds of resources were showing itself that had not before because people needed it.

**FACILITATOR:** Ah, thank you, thank you. Now the next question is how did your tasks, or the activities that you were engaged for the Say Yes COVID Test Project, compare to the normal activities you used to do in your organization that you are part of, church and other organizations that were mentioned? The things you were doing for the Say Yes COVID Test Project, were they different or similar to what you were already doing before?

**PARTICIPANT 2:** I think it enhanced and took us to a new level of supporting our community, that's how I looked at it, and I was excited to see some things happening through Pitt County that I had not seen before, and I think because of COVID-19, the pandemic was the real deal, and to help people stay alive, number one, stay healthy was what the goal of this was, but then to see the additional things that people needed, you know, food, clothing, and other kinds of basic needs that we needed was also enhanced by bringing all the people together, and I think it excited people, it gave them something to do, because things shut down. Just to get out was good for some of us.

**FACILITATOR:** Yeah, yeah, so the activities you were doing for the project became better because of the [INDISCERNIBLE]. What you were doing for the project enhanced what you would usually do in your community, your involvement and so on. You had more things to do because of the project, and I understand that. Thank you, thank you.

**PARTICIPANT 5:** Yes, [INDISCERNIBLE], when it comes to the Hispanic population, a large number of the Hispanics in Pitt County are north of the river. It has enhanced our working relationship with them to a point where now they have done it three times, the Hispanic consulate came here to help them get passports, birth certificates, and other things that they needed across the state. The first time we did it, it was probably about 500 people, and the next time it was even that same number, and they’re coming again in October to do the same thing, and so what we learned was that the Hispanic population felt comfortable coming here to our location to do that,

**[01:10:00]**

and so we are working with the consulate out of Raleigh and other groups here in Pitt County to make sure that this location is available to them for people to come from all across the state to get their IDs, to get their passports, and even get birth certificates for the Hispanic population, whereas before we didn't have that contact until after this situation with COVID test kits.

**FACILITATOR:** Thank you, thank you. Again, the activities you did for the project helped to improve your relationships with community members in the community, that's very good. Anything else, Participant 3?

**PARTICIPANT 3:** I would just simply echo what was just said, because it definitely was a bridge between the different individuals, and as the Bishop said, the trust is definitely there because they are very comfortable coming to our facility.

**FACILITATOR:** So the activities for the project helped to increase the trust that the community members had before?

**PARTICIPANT 3:** That is correct, yes, yes.

**FACILITATOR:** Okay, we’ll take that. Now the next question is how do you think the communication could have been better between the Say Yes COVID Test team out of Raleigh and the UNC team, with you and other people who were involved in terms of how they were communicating with you all about the project? How could it be better, or could have been better, if any, if at all?

**PARTICIPANT 5:** Well, I’m just speaking for me, I don't think it could have been any better, because Mildred and others that have been involved have been on point, and even those from Raleigh – what’s her name? We made her a honorary mother here.

**PARTICIPANT 1:** Betty, Betty [INDISCERNIBLE].

**PARTICIPANT 5:** Betty, yeah, because of the information they shared and how we worked together. I don't think it could have been any better.

**FACILITATOR:** Thank you, thank you. Well, if it couldn’t have been any better then I’ll ask the next question. Is there anything else that you would like to share related to the Say Yes COVID Test Project?

**PARTICIPANT 2:** I wish that it could have lasted a little longer, because when the drugstores ran out of the test kits, people were really in a limbo, like wow, now we need to be existing with this, we need to start all over, because people really became serious, and then the other part of it, the cost of those test kits, they were getting ours – we had 25 in the kit – free, and the drugstores were charging, what $25 for three or four? Was it $25, Bishop?

**PARTICIPANT 5:** It was about $25 for two, I think.

**PARTICIPANT 2:** Yeah, it was ridiculous, so they were really abusing the system, I thought, but we wished that we could have more to give out at the really needed time after we had gotten through the project.

**FACILITATOR:** Thank you, thank you. I completely understand. It’s a big change to go from having so many free kits, and then all of that stops.

**PARTICIPANT 2:** And then I would like to say, with the ones that the federal government has been sending into homes, I liked – I really just probably am being biased – I liked ours better because I got used to using ours, and then we had to start using theirs, and we had to learn a whole new process. I like the kits of 25 that [INDISCERNIBLE], two in a pack of five.

**FACILITATOR:** I know, I know, it’s a big difference.

**PARTICIPANT 2:** It is, yes.

**FACILITATOR:** Anyone else would like to share anything? Alright. One question, I do have a few remaining questions, but the next one is are there any challenges or barriers that you experienced with carrying out the project that was specific to your race, age, gender, or your geographical location? I think some of it was mentioned, some of the challenges were really about trust and maybe not people not understanding, but do you think any challenges you faced were related because of your race, gender, or age, sexual identity, language? Okay,

**[01:15:00]**

no problems, nothing. Now we have the last few questions, the next one is what encouraged you to agree to work on kit distribution? You all already answered this, but I have to ask it, if you don't mind, you know, saying the same thing you said.

**PARTICIPANT 5:** Participant 1.

**FACILITATOR:** Oh, I like that, very straightforward.

**PARTICIPANT 5:** I didn't have any choice, I live in the same house, so I had to be involved.

**FACILITATOR:** So you didn't have any choice because of what?

**PARTICIPANT 5:** I had to be involved, I didn't have any choice, we live in the same house.

**FACILITATOR:** Aahh.

**FACILITATOR:** Oh yes.

**PARTICIPANT 3:** She volunteered her pastor.

**PARTICIPANT 5:** Yeah.

**FACILITATOR:** So Mildred, Mildred, you are the reason a lot of people agreed to work on kit distribution.

**PARTICIPANT 1:** Oh wow, well, that's very special. Well, I was just passionate about things to help other people, and I know I worry them to death but you know, but they did join something that they felt that I was right, for a change.

**PARTICIPANT 5:** Well, I think we all saw the opportunity to be of help, to be a servant to the entire community, and there is nothing wrong with volunteering.

**FACILITATOR:** Yes, wonderful, wonderful. Thank you, thank you. Now the next one is if you had to pinpoint one thing that had the biggest influence on your signing up with your community or organization – okay, we already answered this, Mildred – and then seeing the opportunity the assist in the community. How, if at all, was the Say Yes COVID Test team responsive to your needs or the community’s needs, so during the project, were there any additional needs that you needed the team from Raleigh or Chapel Hill to respond to for your community?

**PARTICIPANT 1:** Well, they were very helpful in helping us when we would do a site or setup, if we needed a table – I mean not a table – but spreads or signs, I’m talking about the marketing material. They were very helpful in providing marketing material for us.

**FACILITATOR:** Okay, perfect, perfect. Anything else? Okay. The next one is --

**PARTICIPANT 2:** Actually, I missed that question, I’m sorry.

**FACILITATOR:** Oh, the question was how did the team for the Say Yes COVID Test Project in Raleigh or Chapel Hill respond to the needs that you or your community had during the project?

**PARTICIPANT 2:** I think in an excellent way, because whatever we told them we needed or we were concerned about, they really tried to address it, and so that was good and I enjoyed that part of it, because they asked us that this is your community, tell us what you think they need, and we’re here helping your community, we are with the community, that's the part I liked about it. They made us feel that, starting out with Al and then the team expanded, and [INDISCERNIBLE] that she had to meet every morning with this team, and she would share what we were experiencing in the field, and so she was like our advocate, and she did an amazing job, I thought, of telling exactly what we were experiencing, because if I heard something positive or negative, I would share it with her, and then she would take it from there and present it in a way that we could get results, positive results on it, so she was I think excellent at that.

**FACILITATOR:** Thank you, thank you. The last two, one is how, if at all, has working with the project helped improve your confidence or impacted your confidence in talking about COVID-19?

**PARTICIPANT 1:** A great deal, I learned a whole lot about it, and being a retired public health professional, it has enhanced a lot of my knowledge and I guess my little skillset, whatever that is,

**[01:20:00]**

so I’ll leave it at that.

**FACILITATOR:** Thank you.

**PARTICIPANT 5:** This is Participant 5 again, what I learned through this whole process was not only kept here in Pitt County, but being the Annual Bishop and General Bishop, a lot of the information that I learned was [INDISCERNIBLE] congregations throughout the state.

**FACILITATOR:** Thank you, thank you.

**PARTICIPANT 1:** And I too shared it, because I actually every network that I am a part of, I engaged them in some way, and they helped in all kinds of ways of getting the information out, and I know that they couldn't do something for every single person, that was just impossible, so we had this amazing team of volunteers, that I just wish that I could have given something to everybody that did great work, you know, a little certificate or something, but they did have two celebratory events for us at the country club, and all the people from Duke Chapel Hill came down to be with us, and they honored our work and told us the amount of work and things that we did was real important, because we didn't realize we had done all that, but that's what they said, so we were really sort of shocked. We were just being ourselves, really, and so they honored the work, and so that was good for the people always, you know, there’s nothing wrong with getting a little [PH] stroke, even though we didn't do it for that. We did it because we wanted to make sure that Pitt County citizens didn't get COVID, or if they got it, that they would be well and could have a more productive life.

**FACILITATOR:** Thank you, thank you. Alright, if there’s no more response, then the last question is would you participate in a project like this in the future? Why or why not?

**PARTICIPANT 5:** Yes, because I love serving the community.

**PARTICIPANT 3:** Yes, yes, yes, yes.

**FACILITATOR:** Yes, yes, yes, yes, yes, yes.

**PARTICIPANT 5:** Yeah, give me the same team.

**PARTICIPANT 2:** The same team, oh my goodness, I love it.

**FACILITATOR:** Why would you participate?

**PARTICIPANT 3:** Well, as the saying, an ounce of prevention is worth a pound of cure, so any time you can pass out a kit and have you self-test yourself to prevent a disease is well worth the effort, in my opinion.

**PARTICIPANT 5:** And also it was a need in the community that was met at a certain time.

**PARTICIPANT 1:** I have a picture of my little – I think he was five years old at that time, when we were distributing the test kit – I covered his neighborhood, and his aunt and I, he was pulling his wagon with home test kits in it.

**PARTICIPANT 2:** So they engaged the little people.

**PARTICIPANT 1:** Yes.

**PARTICIPANT 2:** And I did that too with my 4H Club, I have a 4H Youth Club, and so we made sure that all our families, 4H families had access to the kit too, and the kids helped get them to their homes. One of the disappointments, there was a federal housing near where I live, and so we had asked the lady could they come over and set up in the park and let people come over and pick up the kits, and this was going to be their community service project for that month, because we couldn’t meet in person, and we’re still meeting on Zoom, so they were so disappointed that we weren’t allowed to go to this federal housing and set up and pass out kits, but they were able to get them to their home, and the 4H agent, interestingly enough, called me one day and said, “I understand that you all have these kits and your 4Hers are passing them out, could you bring me one for my personal use,” and I said, “Of course,” and we did, and then in the city of Greenville,

**[01:25:00]**

the Fire Department and Police Department had kits, I mean we had these kits everywhere, you know, anybody that wanted them, so we covered a large portion of our county, and for those that wanted them, we really did, and we are just proud of having had that opportunity, and we would love to do something like that in the future, just to help save a life, definitely, and enhance our community overall.

**FACILITATOR:** Wow, I could not think of a better way to conclude our focus group. Thank you very, very much. I have to say, I really appreciated finally meeting Mildred and her team, I’ve heard so much about you all, and it’s a real honor to meet you and hear it from you directly, your impact and involvement in your community, so I’ll pass it now over to Interviewer who will – [end of audio]
