# Supplementary material for: Exploring barriers and facilitators of implementing an at-home SARS-CoV-2 antigen self-testing intervention: The Rapid Acceleration of Diagnostics—Underserved Populations (RADx-UP) initiatives
Source: PLoS One. 2023 Nov 16;18(11):e0294458. doi: 10.1371/journal.pone.0294458 (PMC10653400; doi:10.1371/journal.pone.0294458)
Supplement: S1 Dataset — (ZIP) [file pone.0294458.s002.zip › PID3-Transcript (08.16.22).doc]

**INTERVIEWER:** Okay, perfect. And if it’s okay with you then I’d like to begin now.

**PARTICIPANT:** Okay.

**INTERVIEWER:** And my first question is -- we’ll begin some questions about your role at the participating organization so, we’ll start with the first question which is; what is the primary mission for your organization that you were part of [INDISCERNIBLE]?

**PARTICIPANT:** Okay. As I said, I’m retired from the health department. I do have a non-profit that I founded before I quit the job, but I enjoy because it allows me to interact with the community. I serve on many Boards in the community. Mental health boards and Department of Social Services Substance Abuse, so, it’s almost like the whole gamut. So, it gives me an opportunity to get to know the community. The mission of my organization is pretty much focused on women and girls. I want to make a difference in their lives, so, anytime I have an opportunity to do something in the community and bring it back to my organization then I’m all for that. And it kind of keeps me abreast of what’s going on because I serve on many Boards and commissions in the community.

**INTERVIEWER:** Wonderful, thank you. And I think you mentioned you founded an organization and I’d like you to share a little bit of your position at your organization that you referred to?

**PARTICIPANT:** So, I’m the President of the organization. We are real big on advocacy and so, like I said it’s a non-profit. I have a 501(c) with this organization, so, it allows me to move quietly in the community to again assist the people that I work with, with the focus on women and girls and families in general.

**INTERVIEWER:** Thank you. And next I have a question about the organization and the Say Yes Corporate Project? In your opinion what made you or your organization a good partner for the Say Yes Corporate Project?

**PARTICIPANT:** Okay, first let me say that I didn’t go willingly into this project. I was asked to represent my church, and it was on a Sunday, Easter Sunday. And I said well okay, let me just go and get the materials, so I did. And I was all in as I found out about what was going on with this project. So, I just stayed, and I enjoy and I was able to get kits out to many people. People always know I’m a resource person so, they were calling me left and right. I was so tired of those kits I did not know what to do because everybody wanted kits. But I thought the project was really good, if I’m answering your question because again, it touched the people. The smaller group of people rather than larger like the corporations and people like that. It was right at the grassroot level and I was real interested in that so, it was easy for me to get involved once I found out what it was all about. So, that’s pretty much how I came to this project. I was standing in for another person at my church. Because what Mildred and that group above us was trying to do was to look at different municipalities and get them involved as a way of reaching the people. And so, the representative in my area was our county -- he was the town council -- I come from a small community and he was the representative and he was out of town because he was a nurse and he was doing nurse duty --

**[00:05:00]**

as a traveling nurse, so, I filled in to go get the kits and to take them back to my church and things of that nature. And so, it was just an easy thing for me to do.

**INTERVIEWER:** Thank you. Thank you very much for that answer. I can understand your interest in the project as you mentioned, giving that it was focused on reaching the people and what you mentioned about your organization also in your role in the community. And related to that question I have another question that is, how do you feel about the Say Yes Covid Test Project’s ability to address your county’s Covid-19 needs?

**PARTICIPANT:** I think it was extremely successful because as I said we were at the grassroot level and most of the people, and I would probably say 100 percent of the people that were involved in this project, they had connections. And so, when they had the kits and so forth, because they were trustworthy people it made it easier for the community members to buy into what we were doing. Because you know, the first, when this project -- or should I say when Covid-19 came out, especially the vaccine, people did get very, very concerned and I knew some of the background because I was brought up in that area of how many times Blacks was used as guinea pigs and so forth when something new, research or whatever, was coming out. But because of the people that were involved with this project, they were trustworthy people and other people in the community would, they could relate and they could have confidence in the project, the vaccine, et cetera, because of the people that was saying okay, this is okay. This is not going to harm you; it’s here to help you and that kind of thing. So, I think that made it easier by having people that was closer to the people, and people that looked like them as well. That is important as well, when you’re looking at trying to get people to do something. People want to see somebody that look like them. They tend to have a little bit more trust. Not saying that other people aren’t trustworthy but with this especially it was something new. It was something different, although like I had the polio vaccine and it never dawned on my because I have my mark on my arm, but of course as time went on you don’t have to go that route to get that vaccine. But I’m saying that by having someone that looked like them involved I think it made it really easier for people in the community to trust and then do like they supposed to do.

**INTERVIEWER:** Thank you. Thank you. And just to confirm, you do feel you would say that the Say Yes Covid’s project was able to address the county’s need overall, but the role of the people involved made a big difference in that? That’s what I’m hearing.

**PARTICIPANT:** Yeah. I would say that in terms of the whole county by the people that was involved with the project, we were able to help the county as a whole. That means it was less people -- it still was a lot of people going to the emergency room and being hospitalized, but I believe it would have been more. So, with East Carolina Vidant Hospital and their public health center, all of those working with these community people it just made it really, really well. Like I said you could get your shot; so many different ways that you could get the vaccine. That made it really well for the, for the community so, I think the partnership with what the lay people were doing, that collaboration with the professional people, I think it really impact the community in a very positive way.

**INTERVIEWER:** Thank you. Thank you. I’m going to ask some additional questions now related to the Say Yes Covid Test part program,

**[00:10:00]**

as you’ve already shared some very useful information. So, now the next question is how, if you can describe how you were initially contacted about this program? I know you touched on this earlier, but if you could share again how you were contacted? By who, and yeah?

**PARTICIPANT:** Okay, and as I said, Mildred Council, she was the one that asked me to come. I think probably everybody in Pitt County has my phone number. And she knew that I went to a particular church and they were trying to get many of the churches involved and she could not reach the councilperson as I had described. So, she called me and she asked me if I could come over to Holly Hill Church and get some kits for St. John, and so, I said okay. I was a little reluctant because I just don’t know how to say no. So, I said well okay, I’ll come over and get them and I thought it was just going to be a one-time thing. But for some reason I was talking to the other people there and I saw how useful and how beneficial it was to the community. So, I bought in right from that point on, so, I was not the first person to represent my church. But I guess you call me a substitute. I went over, picked up the kits and everything and from then on everybody was calling me. They really didn’t even know the councilperson anyway. So, it was easier for me to deliver kits and I think, I can’t remember now how many that I give out but it was like way up there. I think it was almost, it was about 800 or so? Yeah, 800 to 1000 kits.

**INTERVIEWER:** Wow.

**PARTICIPANT:** So, it was a lot.

**INTERVIEWER:** Oh, wow.

**PARTICIPANT:** But everybody kept calling, calling, calling. They wanted them. They saw the value in it, so, that was how it was.

**INTERVIEWER:** Perfect. Wow, that’s definitely a lot of kits, but glad you were able to assist with distributing them. Now, with regard to, with the communication with Mildred, I would like to know in your opinion if you think this was the best communication approach for getting you involved or someone involved [INDISCERNIBLE]?

**PARTICIPANT:** I probably would think so, you know, because --

**INTERVIEWER:** [INDISCERNIBLE].

**PARTICIPANT:** Yeah, I think, I think so. As I said I’m real busy and sometimes you’ve got to pick and choose the things that you can do and not do. But like I said this was one, this project was one that was real important. So, yeah, I think by number one, I knew her anyway. We worked together on many things so, again, it’s all coming down to trust and relationship. So, I had that with her, so, when she called it was kind of hard to say no. And then when I saw what they were doing, I said yes. So, yeah, I think that was a good way to go.

**INTERVIEWER:** Perfect, perfect. Thank you. The next question relates to some of the things you’ve shared already, but just for formality if you can, maybe you can elaborate some more. What made you or your organization decide to participate in this project?

**PARTICIPANT:** Okay, so, I work with the whole community. I do not discriminate, but I do tend to work more with the African American community. So, again that made it easier because they did not have the information and information, good information that you can share is really important. So, it was easy again for me as well people that were working with me to buy into this project. To want to do something because it benefitted the community. It benefitted the African American community because they were really dying from Covid, so -- and if they weren’t dying, they were hospitalized and they were in the hospital for long periods of time. So, anything that I and my group could do was really important. So, we did. We embraced it.

**INTERVIEWER:** Thank you. Thank you for not only answering this question but [INDISCERNIBLE].

**[00:15:00]**

Now related to this question what were you asked to do specifically for this project when Mildred reached out to you?

**PARTICIPANT:** Well, to be quite honest I was connected to more than one way to this project. Yeah, Mildred had reached out to me and it was for my church, but then there’s another organization that I was involved with and they really stuck with this project all throughout, and that was the National Council for Negro Women. So, I’m a member of that organization and so, between -- I think Mildred is a member as well -- but you see many time people have more than one connection and so, it makes it even more important to get involved with these activities. But the National Council for Negro Women, they set up booths, they gave out kits. They passed out the educational material and things like that. So, I don’t even know if I’m answering your question but it was more than one way that I got involved, so, it kind of cross-linked with me.

**INTERVIEWER:** Thank you, and this is very helpful because the next question is how close were these activities for the project related to your task for your role in the community?

**PARTICIPANT:** Okay, say that --

**INTERVIEWER:** Should I repeat it?

**PARTICIPANT:** Mm hmm, say that again? I’m not quite sure --

**INTERVIEWER:** Oh, yes. How -- yeah, so the activities you were [INDISCERNIBLE] were those activities to [INDISCERNIBLE] before the Say Yes Covid Test project came into the community?

**PARTICIPANT:** I believe --

**INTERVIEWER:** How similar or related were those activities?

**PARTICIPANT:** Well, it all to me came together at the same time, if I’m answering your question. If I heard it correctly. Like I said, the National Council of Negro Women, they had decided they were going to buy into this project; they were going to help out. That was on their level, and then like I said, because they were asking churches to get involved through the elected official. And it was ironic here in Pitt County. I believe there was an African American either mayor, councilperson, et cetera, in each one of the towns, so, it just made it real easy for everybody to get the information and we started right out doing it on a regular basis. It wasn’t like we were doing it on a monthly basis or every two or three -- it started out and it grew strong and it just lasted the whole time. So, when there was an event, we would show up and we would pass out kits and encourage through the educational material for people to get their shots and everything. So, that’s how it went and it was almost like it was hand-in-hand with Mildred being the lead person for us here, and I believe that National -- the Black Social Workers. It was a couple other organizations but with them and with the National Council for Negro Women it was just easy to pull all those groups together. It was almost like a true collaboration where everybody saw it as a task or as something they could do and it wasn’t where it was really draining, but it was a need and everybody was able to do their part. I don’t know again if I answered it the way you wanted but that’s how I thought.

**INTERVIEWER:** Thank you. No, thank you. There are no right, there are no right or wrong ways to answer the questions, so, thank you. The next question is about what, about the resources you required to complete this task for the Say Yes Covid Project by you or your organization? And I can repeat it just to make sure you understand. It’s really just if you can share what are some of the resources

**[00:20:00]**

you required during your time in the project to help carry out the tasks that was needed for the Say Yes Covid Project?

**PARTICIPANT:** Well, if I could answer it like this. The kits were at a certain location. All I had to do -- and I know everybody else probably followed the same process -- you call and say hey, I need 20 kits, for example. And so, then you would go by and you would pick them up, and what was really great you’d just, you know, with your car you didn’t have to get out, you didn’t have to touch another person or whatever. The people at the site was able to put the kits and everything in your vehicle and then you could go ahead and leave. The other thing that was good about resources was during this pandemic foot shortage happened and a lot of people did not have a lot of food. So, an organization that really helped with food, they were able to give food boxes while we were giving out the kits and that kind of thing. So again, that collaboration, that working together, it just made it so easy for us and as we got the kits, we did get the literature as well. So, we combined all those resources together to help the people in the community and as I said, they were able to set up appointments to get the shot. And then there was a couple places where I believe you didn’t need an appointment. You could just go and get an -- in your car -- and you could get your shots that way. So, that was very helpful. So, I think the resources, the way the whole county did it I think was real helpful to the average person. They could just drive up in a car, they can get the shots that way, or like with me, I got Moderna. I got it from the local health department and I told a lot of people to get it that way when they would call and ask where they could get it. So, that was a place that I suggested because they had a real easy system to put you down for your shot and you can go on about your business. That was another resource I thought that was handled very, very well. Vidant had a really excellent program. They did the Pfizer and they opened up the convention center and people were able to book their appointments so, that went very smoothly as well. And then they also had the drive-by, so, you stayed right in your car and you got your shot that way. So, I think all-in-all it was just really good resources. But I think early on to be able to get those food boxes, people really appreciated that. To get their shot, to get a food box and go on about their business. So, that made it really, really easy.

**INTERVIEWER:** Thank you. Just to clarify were the food boxes being distributed with, at the same time, when you were distributing some of the Say Yes Covid Test kits, or was it just for the vaccine?

**PARTICIPANT:** Sometimes. Sometimes. It just depended what kind of activity it was and what you could do. So, it was always to try to do just as much for people as possible. Sometimes, like we did the test kits over at Piggly Wiggly. You know you couldn’t have no food boxes there because they sell food, but as people was going into the grocery store, we would tell them about the vaccine and we had two nurses over there who could give them the vaccine right then and there. So, that was a good thing. So, it was just all kind of ways. You either had the test strip and then you could go somewhere and get your appointment to get the shots. It was just a lot of different avenues and I think they all were good here in Pitt County. But the nurses --

**INTERVIEWER:** Thank you. Thank you.

**PARTICIPANT:** Uh huh. The nurses in the different sites, they were able to give people the vaccine and that was, it was pretty simple. It was pretty easy. Of course they kept the record and gave out the card. We all got our card, but by-and-large it was an easy, to me, it was a very easy process.

**[00:25:00]**

**INTERVIEWER:** Thank you. Thank you for the information about the other strategies that you engaged in to help with the distribution of the kits and I’m curious to know now, based on this next question, during that time with the project, did you have any questions for the project team at all during your collaboration with let’s say Mildred or any of the staff that you reached out to when you were collecting the kits? Or just maybe any of the Say Yes Covid team members, did you have any questions for them?

**PARTICIPANT:** No, I thought everything was pretty self-explanatory. I didn’t have any questions, but I felt that if I, if I did then I could get those questions answered. Another thing, and I can’t say necessarily if it’s unique to Pitt County or not, but our health director was involved. Of course you know what the health department was doing anyway, but in terms of information and getting that information out to the public he was very active in that. We have another organization called Pitt Partners for Health and it is made up with probably 50 or 60 different organizations. So, now you got people that are able to go back to their prospective agencies and so forth and give out good information. But the health director kept us informed about the status of what was happening with Covid, and he still does with Pitt Partners for Health. And they sent out news releases so again, the public would know what was going on and I believe that helped to dispel a lot of the fears perhaps that maybe people would have had, had that not happened. But the idea that information was free-flowing and it wasn’t like they were hiding anything, I think that made people feel a lot better as well. But yeah, he did a lot and also with the county commissioners, he was reporting to them about what was going on with Covid and that kind of thing. So, again it was a comprehensive approach. I guess I still go back to that, giving out the masks, the kits and all of that stuff. Getting the education and being able to work with different layers of people. And like I said, I didn’t really have any specific question. I probably wouldn’t have known what to even ask for but as people talked and shared the information, you know you could always then ask additional questions. So, it was fine as far as I’m concerned and I kind of knew where we were going and we went there.

**INTERVIEWER:** That’s very true. You were able to distribute a lot of kits as you mentioned, and it sounded like there was a lot of other information in your county to help address any questions if you had any. Going now to the county level, do you think the project, specifically the Say Yes Covid Test Project addressed a need, a county need related to Covid-19 health concerns? I know you touched upon this a bit earlier, but what do you think may have been addressed by this project more specifically?

**PARTICIPANT:** Hmm. I don’t know if I really know, but I do know that everybody in our county was working together. It wasn’t where they were pulling against each other. Everybody had the main goal to make the county really safe, so, I know that at the county level they were making sure the information got out and like I said with the appointments and things of that nature. Made certain that people knew where to get the kits and that kind of thing. So, it flowed very well, and I also would like to say that even on our county commissioner,

**[00:30:00]**

we had three African American individuals that serve on the county commission level. I’m not saying that other people wouldn’t have done what they needed to do but again it goes back to that level and trust and identity. So, they were there and many times they were picking up kits, they were distributing kits to people and so forth. So again, it was just a real community event and a community affair, and I think that was really good. Again, if people see people that look like them -- and I’m not saying all the time because I have doctors that I have a lot of confidence in and they are not all African Americans or minority doctors, but I’m saying that it helps. But we just work together, like I said Vidant Hospital, that collaboration with the health department and the community organizations. It worked really, really well and I think through Pitt Partners for Health by having all these groups coming together -- we meet like on a monthly basis -- you already had trust and you already had awareness of what each person was doing even before Covid. So, I think that also helped. It wasn’t where we had to go out and reinvent a wheel, but now if you ask somebody maybe at a different level, they might did invent some wheels, I don’t know. But what I saw I thought it was in a way of speaking kind of seamless.

**INTERVIEWER:** Thank you. Thank you. Based on what you saw, what were the needs in your county related to Covid-19?

**PARTICIPANT:** What were the needs? Well, I’ll tell you, there was one that was an issue prior to Covid, and I think it did get better during Covid and I think also as Covid is kind of stabilizing, and that was housing. We had a lot of homeless population here in Pitt County and some people -- and I don’t mean to generalize or stereotype but some people kind of really like to be outside and so forth. And then there are others who really needed places to stay and didn’t have those places. And I know that we have a shelter here, but the way it’s funded is that you get up in the morning and you have to leave, and then you come back in the afternoon around 5:00, and then you can sleep there through the night. And I don’t know where they got the money from or anything like that but they were able to let people stay at the shelter all day. They were able to get hotel accommodations for people that needed that so, that they would be on the inside. And I think some of those they were really, really important because people had somewhere to go and they were able to have better hygiene and different things of that nature. And so, I think that was a problem, like I said, prior to Covid and then during Covid that’s how it was addressed. Now I think they’re going back to the way they used to be but I believe the city of Greenville had a grant that people that did not have a place to stay they could support them through helping individuals find a place to stay and also paying the deposit for a place and that kind of thing. So, those are the kind of things that I saw that was extremely helpful. Now the other things were the kids that were at home, some of them didn’t have internet. Now that was a real issue for a lot of kids, but the city of Greenville, they have free wi-fi. You just kind of get it all over the city. That’s what they had, so, you could go out to say, for example, this one park in the Black neighborhood. What is the name of it? Anyway, it’s a park over there. All you have to do is sit out there on your picnic table and you can get wi-fi. I’m not saying it’s the ideal situation, but I have seen people park cars and get it that way. Of course if you go in some of the stores

**[00:35:00]**

you can get the wi-fi. So, our children were able to keep up with their homework and many times teachers were reaching out to them constantly. I tell you, I commend those teachers because I hear that there is a lot of shortage of teachers. They probably had burned themselves out for all that they were doing during that Covid experience. So, I think housing and I think the internet were two of the things that I remember. And there probably was some other stuff. You know, as you get further away from a situation you tend to forget. But those were the two things that I know that made a real impact in this community.

**INTERVIEWER:** Thank you. Beautiful.

**PARTICIPANT:** And of course the other was the food boxes. You know, being able to help people, because there’s some people that don’t qualify for food stamps or whatever, the EBT card. So, having many of the churches, they got real involved with doing food boxes. So, one church is doing it on Thursday, another one is on Friday, another one is middle of the week, Saturday. And so, people were able close to where they live to get food. So, I guess I would say food, housing and then internet.

**INTERVIEWER:** Thank you. Thank you. Now, related to the community that you shared about your community involvement and what do you think are some of the biggest barriers to Covid-19 testing in the community? Would be during the time of Covid and your involvement in the Say Yes Covid Test Project, what do you think were some of the biggest barriers?

**PARTICIPANT:** Well, I think the biggest barrier was some loud-mouth people, many time on TV and many times in the communities said they were not going to get the shot. It wasn’t that there was a barrier to going somewhere and get it. They were afraid; they had a lot of suspicion about the shot. But I know that if people --

**INTERVIEWER:** Sorry, the question is about Covid-19 testing? Sorry.

**PARTICIPANT:** So yeah, that was what I was getting ready to say. First you got to get them there so you can test, right? But what I was getting ready to say was that they also had opportunities. If people didn’t have transportation, they could get transportation to go to a site to be tested and so forth. But like I said if I saw a barrier, it might would be the misunderstanding that it was important and that it was going to be true. Once you get a suspicion in your mind that somebody is going to do something to you, they don’t want to be tested. But there was money available for people that did not have transportation, they could go to a site to get tested or whatever. So, to me it really wasn’t much of an issue but for other people it could be.

**INTERVIEWER:** Thank you, thank you. And based on what you just described, do you think that the Say Yes Covid Test Project responded well to getting tested for Covid-19?

**PARTICIPANT:** I think so, because some of the places when they had events -- now, like I said, I did a lot of distributing tests to people one-on-one or whatever, but many times they were set up sites and many times they would be at the grocery store and places where people were constantly frequenting. So, therefore they were able to come get kits and do what they needed to do and I thought that was good. I thought that was real strategic, that where people were hanging out, that’s where you would find folks distributing those kits. Those were kind of what I would call the events and then others were like on a more personal level passing them out to people when they call needing the kits.

**INTERVIEWER:** Thank you. Thank you. And at the end of the Say Yes Covid Test Project, how close did your typical activities match

**[00:40:00]**

what you were asked to do for the project in the beginning? If you remember your initial conversation with Mildred and other people who spoke to you about the Say Yes Covid Test Project, how close do you think your activities toward the end of that project were to what you were told in the beginning?

**PARTICIPANT:** I don’t know that I can really answer that. I know that at first, I think it was, it was to get people to use the kit and all of that kind of stuff. So, it was something that was new. By the end of the project people knew about them. They valued what was going on and they wanted them; if they didn’t want them for themselves, they were getting them for other people that they knew could use them. So, the people themselves were identifying folks. So, I think at the beginning it was more, it was something new. It was something different and it had to grow and I believe that’s what happened. It grew and I believe when people got the kit they valued it anyway, but I’m just saying I think it started out one way and by the end people saw the value in it and they wanted to help not only their family but a neighbor or extended family. If that’s what you were asking me.

**INTERVIEWER:** Yes, thank you. That’s very helpful to know. And related to that question, did you find the task that you were asked to do by the Say Yes Covid Test Project difficult?

**PARTICIPANT:** No, it wasn’t difficult. Like I said it was not on my agenda to do. That was not what I was going to be involved with. But as I said once I picked up the picks I just got involved. It was something that was necessary, something that needed to be done, and so, then I did it. But that was not what I had set out to do. So, I think that --

**INTERVIEWER:** Thank you. Thank you. [INDISCERNIBLE] appreciate it. Oh, go ahead. Please continue.

**PARTICIPANT:** Well, that was pretty much what I was saying, that many times things, you do something in the beginning. It’s just like -- let me see now. If someone says hey, sample this cake, you just take a little piece and then all of a sudden you say oh, this cake is very good, so you want a bigger piece and so forth. Now of course that’s not the same thing so, when I started, I was just doing a favor for someone and then all of a sudden, I saw the value of what we were doing, so, I was all into that. So, that was really good. If that is answering your question.

**INTERVIEWER:** Thank you. Thank you. Now, we’re coming toward the end of the interview so, I just have a couple more questions. And the next one is what are some reasons individuals in your community ordered the test kits from you. I know you touched on this earlier, but it’s one of the last few questions. If you can reshare and re-elaborate what you think some of the reasons were?

**PARTICIPANT:** I think people wanted to be safe. They wanted to make certain that people around them weren’t sick and so, as I said, once the project started people saw the value of it themselves because they wanted to keep their families safe. And so, by the kits being so close to them in terms of you could call someone and get a kit; you could go somewhere, and you can get your kit it just made all the difference.

**INTERVIEWER:** Thank you. Thank you, I appreciate that. And the next question is related to any recommendations that you have for the Say Yes Covid Test Project to improve

**[00:45:00]**

their approach overall?

**PARTICIPANT:** See, I think it worked well. Now, I’m sure there might have been some hiccups and that kind of thing, but I think the way it was handled in Pitt County, I think it was really good. I think perhaps in the beginning maybe there was not a lot of information that it was going to happen. This was going to be a tool and maybe they didn’t need to know that. But as people saw how they could easily get the kits and so forth, it just made life real easy so, people reached out to individuals. And the good thing about this project, it was done close to people. People knew folks that they could get a kit from. It wasn’t like they had to go through some bureaucracy or something like that in order to get a kit. So, it just made it easier all the way around so, they knew someone. It was somebody that lived in their neighborhood or not too many streets from them. It was an organization that they were aware of, and so it just made it easier for them to get kits. But I think maybe at first just not having the information might have been an issue but as soon as the kits was available and they started to get out people saw the value for their own family and that’s why they did. They welcomed those kits.

**INTERVIEWER:** Thank you. What you just shared is related to [INDISCERNIBLE] question about people ordering the kits online or in person. I don’t know if you were aware about the online option, but were you aware of the online option as well?

**PARTICIPANT:** Not at first. Not at first, I wasn’t. But like I said even when people were asking me about kits, I was able to go to the source, to Holly Hill Church and get kits. You know, make the phone call that said I’m going to need so many kits or whatever. And if they were running low they said well, we’ll have them by two days from now, something like that. So, it really wasn’t a problem in terms of getting the kits and so forth. But I didn’t know at first that you could order them, but then that was a good option, too. But the way the mail run, but I think they came back in a reasonable time. I don’t know, but I was always aware of where you could get the kits, so, it wasn’t an issue.

**INTERVIEWER:** And when you did find out about the online option, that people could order it themselves or to collect it from you, which method do you think was preferred, the online option or the ability to call you and just?

**PARTICIPANT:** Picking them up.

**INTERVIEWER:** Picking them up [INDISCERNIBLE].

**PARTICIPANT:** Yeah, people liked that. It’s just something about immediate, if you want to say immediate gratification or whatever. And even if you told them look, I won’t have them until Friday they were okay with that. So, yeah, I think being able to pick them up was better than ordering them online.

**INTERVIEWER:** Thank you. Thank you. And for the last question I would like to just ask you what would you like to see happen in your community and the health of your community?

**PARTICIPANT:** Mm, so, what are you talking about? Are you talking about Covid or just in general?

**INTERVIEWER:** In general.

**PARTICIPANT:** Well, as I’d say -- and I could be prejudiced because I’ve worked in the healthcare profession. Just recently they opened up another clinic where people can go on Sunday -- I think it’s two Sundays a month -- to the health department as a matter of fact, for free checkups and that kind of thing for diabetes and that particular issue. They always had some places, but it seemed like people are opening, well should I say the medical profession is opening up a few more places. So, I think that’s really good because people need healthcare. They need that a lot and there are some people

**[00:50:00]**

and I know with Affordable Healthcare and some of those kind of options, there are still people who are falling through the cracks. And the emergency department is still overwhelmed because not enough maybe primary care providers because people don’t have the money. So, I would like to see something like that happen for the community to help out with that. But I think we’ve done a great job in terms of being outside, getting exercise and different things of that nature. We have done that. So, in all of that I think we’ve done a pretty good job, but like I said, I could be prejudiced. I don’t know.

**FEMALE 1:** [PH 00:50:56] Interviewer, can you still hear us? Okay, I’m not sure if he can hear us anymore. It looks like his microphones gone, so maybe he got off. But that was the last question anyway. So, that you for participating. Oh, there he is.

**INTERVIEWER:** Hello, I apologize. I think the internet disconnected on me.

**PARTICIPANT:** Okay. But I think I answered the question, I guess.

**INTERVIEWER:** Yes, you did. Yes, you did. I was listening as it cut off but I heard a lot of the answer before it was disrupted. But I really appreciate you making the time to share your responses for all these questions that we had and appreciate all your efforts for the project overall and we thank you for your time again. And you will see your gift card soon enough, I believe. So, if you have any questions for us, you can [INDISCERNIBLE] us or ask any questions if you have any.

**FEMALE 1:** I just need to confirm your email. What is the email you would like us to use for your gift card?

**PARTICIPANT:** It’s [Participant name].

**FEMALE 1:** Okay.

**PARTICIPANT:** [participant email address]

**FEMALE 1:** Perfect. Okay, well, thank you so much for participating today.

**PARTICIPANT:** Thank you. This was rather interesting because it had me to go back. You know, it’s been a while and so, I had to kind of go back and see and remember things. So, it was good. I appreciate it.

**FEMALE 1:** Good, we’re glad you enjoyed it. Thank you so much.

**PARTICIPANT:** Okay. You all take it easy.
