# Supplementary material for: Exploring barriers and facilitators of implementing an at-home SARS-CoV-2 antigen self-testing intervention: The Rapid Acceleration of Diagnostics—Underserved Populations (RADx-UP) initiatives
Source: PLoS One. 2023 Nov 16;18(11):e0294458. doi: 10.1371/journal.pone.0294458 (PMC10653400; doi:10.1371/journal.pone.0294458)
Supplement: S1 Dataset — (ZIP) [file pone.0294458.s002.zip › PID6 transcript_08_03_2022.doc]

**INTERVIEWER:** Okay. We’re recording now. If it’s okay with you, we can go ahead and get started. So, I’m going to begin with some questions about your role at the, at your organization or the organization that participated in the project. So, what’s the primary mission for your organization?

**PARTICIPANT:** Resource, connections, feeding, kind of a little bit of everything in that. But basically, the resource and feeding to administrate, with everything else just added on in the ministry.

**INTERVIEWER:** Okay, and what’s your position at that organization?

**PARTICIPANT:** I’m the founder and president of Churches Outreach Network in the interfaith clergy.

**INTERVIEWER:** Okay. And then next we just have a couple questions about your organization and the Say Yes Covid Test Project?

**PARTICIPANT:** Mm hmm.

**INTERVIEWER:** So, in your opinion what made you or your organization a good partner for the Say Yes Covid Test Project?

**PARTICIPANT:** What made us a good partner, you ask? The question?

**INTERVIEWER:** Yes.

**PARTICIPANT:** Well, I guess I think what ended up happening, that they reached out towards me because we also have a disaster recovery inside Pitt County, and that was something that was put together when Matthew came in 2016. So, we get anywhere between three to four tractor-trailers still now that comes into our community all year round with products and stuff like that. So, I think when they reached out to me knowing that we had a warehouse; we’re already kind of doing that since 2016.

**INTERVIEWER:** Mm hmm, yeah, that makes sense. And how do you feel about the Say Yes Covid Test Projects ability to address your county’s Covid-19 needs?

**PARTICIPANT:** To me it was very good. Thought out on having it. I think it happened around Easter time when, that year, was it ’19, I believe? When they first done it. The problem that I kind of seen is that they, that’s something that should have been continued going on. I mean, if people was being tested before they went to their job or babysitter or whatever they went over the course of the day, or school, they would have known they was positive before they went and I think that we could have stopped the spread a lot more better than what we did. I think we had a good pilot test. It was good and we should have continued having that going on. I think that, that should have been something that people should be able to test their selves. It was just like if a person has HIV, they do not want to go to the clinic to find out if they have HIV so, they was able to test their selves at home before they go out. So, I’m really sad that it was a very small project that ended so quickly, but then realized that the President actually had Covid kits go out, but it was after the horse left the barn. Closed the barn.

**INTERVIEWER:** Right, right. That makes sense. I’m just going to ask a couple more detailed questions about the Say Yes Covid Test Program and your experience. So, can you describe how you were initially contacted about the program?

**PARTICIPANT:** Yeah. Mildred Council knows me very well. I’ve known her since I been here for 17. So, she reached out to me, and if she ever reach out because they know that Churches Outreach Network, because we do feeding, we do senior citizen food programs. We have Toys for Tots for 14 counties. We work a lot in the community, so, we’re more of a community advocate for the communities and everybody knows to reach out towards me as well, as we have a television broadcast, and we have an Eblast database that goes over 10,000 people. So, we really can send out the information towards the community.

**INTERVIEWER:** Okay. And in your opinion was this the best communication approach?

**PARTICIPANT:** Yes, it was. I mean, I like how they kind of put together, which they started with little teams, and some people didn’t know; didn’t know what they was doing, but then I know those who was doing, we really kind of connected and made it happen. It was just the only issue that was that they thought that Amazon was in the area. I could have told them that Amazon wasn’t in this area and which, we took the issue of getting the stuff from the post office. The post office would not deliver it, so, we got a U-Haul truck and we asked Two Men and a Truck. They help me with Toys for Tots all year round. So, they went there, those guys, and they brought it all to the building.

**INTERVIEWER:** Mm hmm. And in general what are your thoughts about how communication worked across the project?

**PARTICIPANT:** The communication was good. I think that the continuing meetings was really good. They was very committed and consistent of it. They value a time and to me that is very important. And was getting things done. That’s the key thing. No one sat around watching grass grow, and they had good communication. They continued on time, had meetings, email you. If they didn’t see you responding to email, they called you. So, that’s what I call -- at a dealership, that’s what they do at a dealership. If you don’t call them back, they call you.

**INTERVIEWER:** Right, right, right. Yeah, that makes sense. And what made you or your organization decide to participate in the project initially?

**PARTICIPANT:** You said why did we decide to participate?

**INTERVIEWER:** Yeah.

**PARTICIPANT:** Because we do everything. Our ministry is more of helping in the community. We help. We’re a community that helps. There’s many parts of the Churches Outreach Network. We work with men and women who comes out from jail. We work with people who are homeless. We help pay people’s rents, light bills. We’ll community network. We have feeding programs. We just believe in helping, and we have disaster sites in different locations. We’re just a network of folks that come together and help in the community.

**INTERVIEWER:** And what were you all asked to do as part of this project?

**PARTICIPANT:** At the warehouse where – my place was the location where everything came in and distributed out to the people. So, those -- they had -- I had folks who have to pick up so it made it even easier. When they was getting the Covid we was also giving them hand sanitizers and masks. I mean, we was able to gibe that that and cleaning supplies all at the same time. That’s at the warehouse. So, my job was to, they would send me who was coming to pick up, how many boxes to give them and we did a log, of course. A log of who took what; had them sign it and kept a record and submit the records to them on that. So, that’s what we was more of, the warehouse facility where everything came to the warehouse. It was one location at where everything could come.

**INTERVIEWER:** Mm hmm. And based on what you just described in terms of what you all did under the project, how close were those activities to your regular tasks or what you usually do as an organization?

**PARTICIPANT:** Well, you know, I’m retired for now 26 years. 23 years, I’m sorry. I worked 26 years there, so being retired I mean, I do this all day long. We have folks that help me a lot in the ministry, so, I’m truly blessed on that. So, we have folks that help us, but it’s just a matter, it’s the same as doing anything else. We was already in the warehouse giving out PPE stuff. We’re already there, it’s just a matter of having more items in there for somebody to pick up. It was nothing to throw you off track. Like a child, if you’re already watching your child, bringing one more child to the house won’t make a difference, you know.

**INTERVIEWER:** Right, and you described previously that you all had the warehouse and that you had experience doing like distribution-type projects related to other disasters?

**PARTICIPANT:** Yeah, we actually -- I’m sorry, I didn’t mean to cut you off.

**INTERVIEWER:** No, I was just, I just want to confirm that I’m understanding that correctly?

**PARTICIPANT:** Yeah, we get, we get stuff that comes down. We do have a disaster recovery team there, so, we get stuff that comes down to our community to help with that. And also, as I work for the corporation of PepsiCo, I mean, I have pretty good tides with many folks. So, sometimes I get a call up to Joe Gibbs has helped me with paying for Feed the Children. We have four tractor trailers go to different areas so, sometimes it’s not just in my area. I have 14 counties that I work with, so, with that being said, that’s what we do. We got a team of people that work with us, a total of about 70 volunteers.

**INTERVIEWER:** Hmm, hmm. I’m sorry, it was 70 volunteers to run the --

**PARTICIPANT:** We’re doing for the 14 counties.

**INTERVIEWER:** Mm, mm, okay.

**PARTICIPANT:** Everything we do between the feeding and the 14 counties and Toys for Tots. And we have many things that we do. I mean, I would long, I would be all day telling you what we do.

**INTERVIEWER:** And so, this, this next question kind of builds on that, but what resources did you require to complete the tasks that you all took on under the project?

**PARTICIPANT:** What resources that we was request to do?

**INTERVIEWER:** Yeah, what resources did you need to make that happen?

**PARTICIPANT:** We already have a database, and we already had a website that is in existence, so, it didn’t take much of having to add that on the website. Also send out the Eblasts. We have a, we use Constant Contact for which we have over 10,000 folks have subscribed on there. So, it don’t take long to put that out there when they send that out.

**[00:10:00]**

So, it wasn’t, it wasn’t too difficult when you already the tools already in place. We already do a disaster. When we done with disaster we worked with FEMA and then when the stuff came down, they helped with funds and we also got case workers, which we actually contract Catholic charity. We contract them to do that work, so, this was just a -- easy way to say it was just a easy donut in the bag. Wasn’t too much to do in that. I mean, we already was doing large quantity of stuff already.

**INTERVIEWER:** Mm hmm, mm hmm. And what resources did you require to complete these tasks from the Say Yes Covid Test team?

**PARTICIPANT:** All that was required is when they sent the information it was a form online because we were going to try to do everything where people had to go all crazy. So, we had an app as well. They could do it online. They put the requests online and they either would just come pick it up at the warehouse. They know they needed five or six boxes. They know what they needed, and we also sent a copy to the person that was over me. And so, she had a copy of who picked up what day and we did a daily log on that. So, it wasn’t too much of a requirement. And then when they was inside the warehouse, we’d also let them know we had some PPE stuff and they could have some of that stuff as well and then we’d just add that on another sheet of paper on stuff they can have as well.

**INTERVIEWER:** Hmm, okay. And throughout the project did you have any questions for the project team during your collaboration with them?

**PARTICIPANT:** Nah, the only issue, the only problem that I had and I think it was just a mix-up of the delivery. That was, that was the biggest issue which, you know, they thought Amazon and we got emails that they going to deliver at -- the post office is going to deliver at this time and we had people that was there which they never delivered. We went to the post office; they had all the boxes sitting in the back room. I mean, all the years I been here I never been to the back of the post office here. So, they had all the boxes there and we made arrangements to get it brought over from the warehouse -- I mean, from their facility to our warehouse. That was the only issue I really had was getting the boxes. Once we had that the post office would call me on my cell, so if we got more boxes no problem. We’d send somebody over there to go get them. And we did a count of each box as we received from them and we did a report on that. How many boxes have we received that day. So, we did a daily report.

**INTERVIEWER:** Mm, mm.

**PARTICIPANT:** And that was required.

**INTERVIEWER:** Yeah, and just so I understand. So, the initial confusion was that they wanted to deliver them via Amazon, but Amazon doesn’t deliver to your area, or?

**PARTICIPANT:** Yeah, right. And where we’re at they deliver in Raleigh but going down towards where we at, there’s no Amazon here. There’s not an Amazon store, but everything is in Raleigh which is where the airline is at. So, they thought that Amazon could deliver here but Amazon delivered to the post office and the post office had to deliver to us, which the post office didn’t.

**INTERVIEWER:** Hmm.

**PARTICIPANT:** So, that was the confusement (sic).

**INTERVIEWER:** Okay. And how responsive were the project team members to your questions?

**PARTICIPANT:** Oh, they was really -- they answered my questions. They jumped on it right away. They didn’t allow too much grass to grow on in. And they couldn’t get me by phone they texted me, let me know this was taking place and then they emailed me they just got off the phone with somebody. Somebody, this person, whatever meetings they was having as soon as that meeting was over, I got more than one person contacting me letting me know what was happening. So, I was updated on what’s happening.

**INTERVIEWER:** Okay. And if it’s different than I guess responses to your questions, how responsive were they to your needs in general over the course of the project?

**PARTICIPANT:** Well, the needs, the building we already had and I think that anything -- I don’t know what we had too much of a need from. Actually that we was just being a service to them when the boxes would come and who they’d want to pick up. Sometimes they had made special requests. This person come from here -- they ran out. And we was already at the building, I mean, no problem, just let me know she’s coming or he’s coming and we gave them what you told me to give her. We follow authorities.

**INTERVIEWER:** Okay. Yeah, that makes sense. Do you think that the project addressed a county need really to Covid 19 health concerns?

**PARTICIPANT:** I’d say that -- let me make sure I heard the question. Was it more of a need because of the pandemic?

**INTERVIEWER:** Yeah, yeah, I guess the question is do you feel like the program ultimately addressed --

**[00:15:00]**

-- whatever you see as your county’s major need around Covid 19?

**PARTICIPANT:** I say they addressed it, yes. I say that, I would say that what ended up happening that, because of the Covid kits, Yes kits came out, that was the beginning. It was what we needed at the very beginning if anyone got Covid. They would be able to test their self at home and they children and that’s what we needed. It avoid people from running to the site. If you know you had it, if your employees say well, if you have it stay home or some employees said well, you go to a site and go get it. Because we didn’t have a lot of sites open at all at that time. So, whatever site was available at least for employee reasons, then you go there. But they had some long lines, some very long lines. Those lines was long, you know, for those folks when they finally got those sites open. So, to me, that was what you needed and continued needing it. I think that that’s, if Covid’s going to be around which I think now they’re saying it’s like maybe a common cold or a flu right now, but still you can test yourself. You know, you want to test yourself. There’s no difference in testing yourself if you got a fever with a thermometer. It’s the same thing. Test yourself. If you got, if you’re running a bad fever take Tylenol, you know. But test yourself. So, I think that’s something that should continue giving it. Well, I don’t know that it would be feasible to give them because those things are very expensive. You know, I don’t think it’s feasible to give it away but I would hope that they would have found some money, Covid money, to help subsidize that. Well hey, if you low income and help give those out and work with some kind of stipend so the families can receive it who couldn’t afford it. But they not cheap, so I mean, that’s why I wanted to help if they would have done, but obviously they didn’t do it that way.

**INTERVIEWER:** And in addition to what you just talked about, what were the needs that you saw in your county related to Covid 19?

**PARTICIPANT:** Well, first two things. One was the people that have education about what was going on. That’s number one. So, we tried to give them education on that where we did, we had the doctors, we did webinars. We had webinars on Zoom where they could get that. But that was the first thing. The second thing is making sure that people have it and how they get it. It was an issue of transportation but we took care of that by working with agencies and the agencies would give it to the people. So, you see the Housing Authority. They was able to get 30 boxes and they hand delivered those to those folks who had it. Well, not have it, had a need for it. So, that’s kind of where we eat up the transportation. We only have seven buses in this whole town and they stop going at 6:00 and they don’t run on Sunday. So, we don’t have the transit. It’s a very -- this area is really terrible on transportation, so, we don’t have that. And to wait for a bus, you know, it could be an hour or so. So, that’s what I would say on that.

**INTERVIEWER:** Mm, mm hmm. Hmm. And what do you think are the biggest barriers to Covid 19 testing?

**PARTICIPANT:** I don’t think that it was the barrier of -- first I would say that it was understanding what it was about. But I would say that the test kits was something that was very simple. I mean, it was self-explanatory, had a little video. Had everything. One of the things that we did was test, do it on ourself and show how easy it is to do it. I don’t see any issue on that. I don’t see anything that will put a barrier over that, but I would then, I would leave again to keep on standing on that, that they should continue having that going on. But when they pulled that and people started getting more Covid and they didn’t know, it just, it was just like a wildfire in the woods somewhere, you know?

**INTERVIEWER:** Right, right. So, for the time that it was up and going do you think that the Covid, the Say Yes Covid Test Project responded well to the barriers that you addressed, or that you noted to Covid 19 testing?

**PARTICIPANT:** I say yes, it responded well. I also would say that unless we didn’t have many people with Covid that -- well, actually if they have it, they wasn’t spreading it because they wasn’t going out. And as soon as the Covid kit tests was gone that’s when all the fire came out. People didn’t know and they was going all over but at least if people know that they had it, or whatever symptoms that they may have, they was able to swab they self and test they self. And test the family so, that made it easier. But once it was removed that’s, then the numbers start climbing.

**INTERVIEWER:** Mm hmm.

**PARTICIPANT:** So, I believe that it prevented the spread but now that they have pulled it away the spread became.

**[00:20:00]**

**INTERVIEWER:** Mm hmm, mm hmm. That makes sense. Switching gears a little bit, I guess. At the end of the project, so, after everything was wrapped up, how close did you feel like your typical activities matched to what you were asked to do at the beginning of the project?

**PARTICIPANT:** Well, it matched. I think that some people had different tasks than I had. My task was to maintain the warehouse and make sure the products and then the follow-up. That was kind of easy. You know, make sure it was there and giving it out. Some people had to go out and do, have test sites and things like that. That wasn’t something I had to do. My main place was to have it coming in, to follow when they sent Amazon, follow-up with that, follow-up with the post office and make sure it was in the warehouse and build a report.

**INTERVIEWER:** Okay, and you’ve already commented on this a little bit, but did you find these tasks to be difficult?

**PARTICIPANT:** Very simple. It was like A, B, C. Very, very simple. Very simple. Easy and it didn’t even have a little video but very simple. I mean, to do that. It’s very self-explanatory.

**INTERVIEWER:** Okay. And what do you feel like are some of the reasons that individuals in your community ordered the test kits?

**PARTICIPANT:** Well, I know that they had -- it was really a big thing for folks who was travelling outside of the state because I think the airlines had put some kind of restriction as well. I know that they wanted it. I know when we didn’t have no more it was really a big disappointment. When you’re out and when you don’t have no more and the site was probably still up where we had kits you can get, boxes, and we didn’t have more to give nobody. It was like looking for water in the desert. We was done.

**INTERVIEWER:** Mm hmm. Mm hmm. And what do you think are some of the biggest reasons that people in your county picked up a testing kit?

**PARTICIPANT:** I would say that they know there was a possibility they could get Covid or may have Covid or maybe spreading Covid. I mean, that is something, I mean, it was really all the news and the numbers going up. It put alert on everybody that you may have Covid. Then employees, a lot of people, you know, things started changing. Employees started allowing people to work from their homes. A lot of people that I know who had rental places, they lost a lot of tenants. People started to work at home and they haven’t came back. They just find it easier to work at home. And it just made a big change. I mean, there was events we was doing every, once a month. We stopped that in ’19. We haven’t had that yet, you know, having all the clergy, having all the people coming with resources. At least have food for them to eat. So, we haven’t had that at all, so, everybody jumped on Zoom and that’s where it been ever since. But I believe that it made it really good for them to know that they could get tested, and to test them self before they went out instead of going to a place and the person said you got Covid. You know, that’s like -- you know, you can walk around. You don’t want them to tell you, you got Covid, but if you got Covid you probably shut your mouth up and stay home. You know, and don’t go nowhere. If you didn’t have part-time employee or tell your employee I don’t feel well. You know, I mean, whatever you want to tell your employee.

**INTERVIEWER:** Mm hmm.

**PARTICIPANT:** But yeah, it was really good to have when they can test them self at home.

**INTERVIEWER:** Yeah.

**PARTICIPANT:** Whether they had it or not, or people coming to their house when they have it. It was really good. It was like an American Express Card, don’t leave home without it.

**INTERVIEWER:** Wow. Wow. And in your community was it an option for people to order the testing kits using the online ordering option?

**PARTICIPANT:** I believe for those who was, [INDISCERNIBLE] for those who have internet experience and they was able to do that. But we have a lot of folks here who does not have that experience and how to do it. So, I do a lot of babysitting and that’s what we do. So many folks do not know how to read or write, so, we had to work with that. So, they know they can get a kit. They get a kit; it was going to be bring to them and they had people to help them with it. So, it was explanatory how you do it, so, I think there was a lot of folks who might be in different areas, different I guess let’s say price bracket, they know how to do that stuff. They doing it. Some folks, some folks, not a lot of folks know how to do that. It all depends. Those who is in a six-figure home, you know.

**INTERVIEWER:** Yeah, so, our next question was around which you thought, which method you thought people preferred for ordering the kit?

**[00:25:00]**

So, online or not online? So, I don’t know if you have anything to add besides what you just replied.

**PARTICIPANT:** I will say that the -- well, first I will say the online would have been good for people that -- let me just explain it. Me and my wife have a difficult issue on paying the bills. I go to pay the bills every month on the 25th with a check. I go to all the people and write checks and go give it to them. You know, all of them, everybody. My storage, everybody gets the check. My wife likes doing it online and said to me I do not like mailing checks. And I thought there was something wrong with me, but I have another pastor who’s 82 years old and he said the same thing. So, I felt good talking with him when I said that his wife likes to do what my wife likes to do. So, I think you need to have both but for me, I think it’s easier. I feel that it’s something to talk with a person than do it online and hoping it come in the mailbox and it’s more than giving them the kit and they’re picking up and with some you have conversations. I believe a personal touch is always better than a preaching word, so, I like the idea of being able to give it to them. And them being able to have it and you being able to talk to them. Maybe some of them may be going through or may need help with something else, and that to me means a lot.

**INTERVIEWER:** Mm hmm.

**PARTICIPANT:** Me personally. But I don’t want to speak for those who don’t like talking to bodies. My wife is not a person that likes to go on or be on a microphone or anything. She’s not a public speaker. So, I won’t push nobody up there who don’t like to do that. Some people just -- my daughter’s the same way. She wanted to be at Zaxby’s but she want to order it on the app instead of walk into the store. Like driving it in there, she want to order it on the app and now it come to the car. Like to me, that make no sense. Let’s go in the store. I’ll go in the store and get my food, you know? I’m sorry, I don’t know. I just like to say hi to people.

**INTERVIEWER:** Right.

**PARTICIPANT:** [INDISCERNIBLE].

**INTERVIEWER:** Yeah, yeah. So, it sounds like both options [INDISCERNIBLE 00:27:06] preferences.

**PARTICIPANT:** Yeah.

**INTERVIEWER:** And do you have any recommendations for the Say Yes Covid Test Project to improve their approach?

**PARTICIPANT:** Can you repeat that?

**INTERVIEWER:** Do you have any recommendations for the project to improve their approach?

**PARTICIPANT:** I think, I think that they did a fantastic job with, to me, which the communication and everything I would say they did a fantastic job with that. I think the only thing that would probably be is, this is what my recommendation with anybody. You need to have someone running the warehouse and you have to have everybody in place and everything. Where’s the stuff going at if there’s, you know, we have to have a warehouse where it should be placed in one house. I think if you have it all over the place and that was some confusement (sic), because I had to call Dr. [PH] John, the Director from the hospital, I had to call him and see, his was coming on a Saturday. And he goes, runs there with a key to open up the place and it never came and he’s calling. And that’s what they said. So, you should have, my recommendation is having a person that can run a warehouse and when the place coming, then continue. What you had, they had the second and third part already set up but they didn’t have the first part where let it go to one location, and one location we know we have an inventory of how many that came there, how many is left, how many went out. So, that’s what I would recommend. But number two they had. They had the communication. They had people sending emails. They had the people setting up the, setting up the tents. Because all the tents went there as well to the warehouse. So, you had to have one location. Don’t rely on UPS. Or who else? Federal Express or the post office but have one location. They could have just shipped everything on one tractor-trailer. Would have been done.

**INTERVIEWER:** Mm hmm.

**PARTICIPANT:** And so, I think they, I think they went to spending more money and more aggravation. They spent a lot of money in shipping that they could have avoided.

**INTERVIEWER:** Mm, mm hmm, mm hmm.

**PARTICIPANT:** That’s my opinion.

**INTERVIEWER:** That makes sense. And then lastly, just want to get your thoughts on a couple kind of big picture questions as we wrap up. So, what would you like to see happen with your community and their health moving forward?

**PARTICIPANT:** Well, first I’m really involved with the health in our community. We have a program called No Excuse Healthy Living Alliance. Very important on that on health. And I’m really involved with the health part, even with those suicide prevention trainings and I’m involved with that. With Living Works. I did a training with them; have another one this year working with them. I had a meeting with the person from Blue Cross Blue Shield. I’m really working on their expansion care for Carolina. I been to legislation with them to close that --

**[00:30:00]**

-- gap so they could make sure they get their healthcare. So, when it comes to health and the need for the community I truly, truly believe that we need to connect them with it. Some don’t know how to get it. We have a broadcast television station that we have resources that come on there that speak to them on what is available, what’s for free. We let them know all the free food. I do believe that food has got to be very nutrition food. I’m really focused on that because I believe your medicine is in your food. I think that you eat the right type of food instead of eating all this other stuff out here that’s really making them sick and stop popping pills. You eat, you are what you eat. You know, I mean, get some walnuts for the brain, apricots. Eat that, that’s good food that is really good for you. And just leave all that stuff outside. I think you are what you eat so, when it comes to resources and health and diabetes, leave the soda alone. Drink regular water. [INDISCERNIBLE] gave me a health tip. Anyway, I believe that health is very important. And it’s really important to let the community know about what is available and help them get it because if they don’t have transportation, they can’t get it and make it easy for them and serve them. Service is the rent we pay for the space we take up on earth. Serve them. Serve them. Help them.

**INTERVIEWER:** Mm hmm. Mm hmm. And do you have any additional questions or comments? Anything that you want to share with us today that we haven’t already talked about?

**PARTICIPANT:** Well, I mean the only thing I would say that I really hope that it is too late now and the horse has left the barn. I wish they would have continued going. I wish that the President and Congress and all of them would have voted to continue to have that, to continue helping these families. They still need to have these Covid kits at the home and if mailing would have been an easier way to send it out then fine, but you still need to have people be able to get it. You don’t want people selling it because, you know, you put things in the mailbox and you get confused. You got to keep where this thing went at. It should be where you could go register, your docs could give you a kit. There’s got to be something they should have done better. And I just think that we started out good and we just went sour. Just let it go, and it’s kind of hard to regain what we started.

**INTERVIEWER:** Mm hmm, mm hmm, yeah.

**PARTICIPANT:** That’s all I say, yeah. I just wish they would have kept it going. They should have kept that going. The President, they should get involved with that and how it was done. They should hit every state with that but they decided to do it at the very end. You know, I mean, the child already got the infection, you know. It was already spreading. It was going too wild. He should have put the fire out when it was just in one little room. Now the whole house is on fire. What do you want me to do? Then I have a house on fire. Let me start there.

**INTERVIEWER:** Well, thank you for your responses and your time today. This has been really informative for us and really been great talking to you, [participant name]. So, I think --

**PARTICIPANT:** No problem.

**[…]**

**BOLA YUSUF:** Okay. All right, thank you very much for your time.

**PARTICIPANT:** I thank you all. I thank you all.

**INTERVIEWER:** Yeah, have a good rest of your day.

**[00:35:00]**

**PARTICIPANT:** You too. [INDISCERNIBLE]. Take care now. So long.
