# Supplementary material for: Exploring barriers and facilitators of implementing an at-home SARS-CoV-2 antigen self-testing intervention: The Rapid Acceleration of Diagnostics—Underserved Populations (RADx-UP) initiatives
Source: PLoS One. 2023 Nov 16;18(11):e0294458. doi: 10.1371/journal.pone.0294458 (PMC10653400; doi:10.1371/journal.pone.0294458)
Supplement: S1 Dataset — (ZIP) [file pone.0294458.s002.zip › P'sID(9)-Transcript (08.17.22).doc]

**INTERVIEWER:** So, if it’s okay with you, we’ll go ahead and get started. We’re just going to be giving out some questions about your role at your organization, or the organization that participated in the Say Yes COVID test project. So, what’s the primary mission of your organization?

**PARTICIPANT:** That I work in, or the organization that did the –

**INTERVIEWER:** The organization that did the project.

**PARTICIPANT:** Well, I utilize my practice as a facility, so I guess I’m referring to my practice. I am a pediatrician, and I’ve been in practice now since the early 90s, and I see children from age 0-18 years, so we were involved because, of course, we see a lot of clients with COVID, and I was fairly interested in getting more aware of the tests so I could better explain and encourage my patients to participate in the screening process.

**INTERVIEWER:** Okay, and you’ve just spoken to this somewhat, and what’s your position at that organization?

**PARTICIPANT:** I’m an owner and I’m a primary care provider.

**INTERVIEWER:** Okay, next we have some questions about your organization’s participation in the project. So, in your opinion, what made you or your organization a good partner for the Say Yes Covid test project?

**PARTICIPANT:** Well, primarily because of where we are located, and this project started out with an emphasis on reaching those patients who are not necessarily the first to seek care, and we are an urban practice, so we have a lot of people that we could share information with.

**INTERVIEWER:** Okay. And how did you feel about the Say Yes COVID test project’s ability to address your county’s COVID-19 need?

**PARTICIPANT:** Well, I think it was a very good project in terms of disseminating tests, but it came at a point where people didn’t realize what was going on. And I think in the advertising, there was a clear disconnect between the test and actually being vaccinated or given medications against COVID, because so many people were resistant to even taking the test because they were so afraid that they had something to do with this death and disease, and they didn’t make that connection that this was a screening test. They thought that by accepting this, they would, in part, be agreeing to some kind of vaccination or whatever regarding COVID, and they would get afraid.

**INTERVIEWER:** So, I just want to make sure I’m understanding. It sounds like the folks’, the barrier was that folks didn’t understand that what was being provided was a test kit vs., like, a treatment or a vaccination.

**PARTICIPANT:** Exactly.

**INTERVIEWER:** Okay, that’s interesting. We have a couple questions now about your experience, your activities under the program. Can you describe how you were initially contacted about the program?

**PARTICIPANT:** Yes. There was a young lady, I wish I could recall her name, or lady who had once lived in the Chattanooga area, and this area was selected as a site by the people who had control of where these kids would go, and because she knew me personally, I was invited to participate, because she was reaching out to community leaders and people that she thought could penetrate the community, and I was selected on that basis, on my practice.

**INTERVIEWER:** And do you remember what organization she was with?

**PARTICIPANT:** I cannot recall. I cannot recall at all.

**INTERVIEWER:** Okay, and what was kind of the mode of communication, I guess, for you? Was it, like, an email or a phone call or –

**PARTICIPANT:** It was an email followed up with a phone call.

**[00:05:00]**

**INTERVIEWER:** Okay, and in your opinion, was this the best communication approach, or what are your thoughts about how that communication looked?

**PARTICIPANT:** Well, I actually, think it was appropriate, because I think you can’t just send an email for those kind of things. You really have to reach out to the person personally. So that was an email describing what it was, but I had a personal call from the person who was interested in participating.

**INTERVIEWER:** Yeah, that makes sense. What made you or your organization decide to participate?

**PARTICIPANT:** Well, as a provider in the community, I’m one of the frontline people who have to know everything about these things. So, when it was introduced, I knew that this was something that would be big for COVID in terms of the ability to screen. So, because of the position that I held and because of what I do for a living, I thought it would be very pertinent and significant that I participate.

**INTERVIEWER:** Okay, and what were you asked to do for this project initially?

**PARTICIPANT:** Okay, we were asked to be one of the sites for holding the kits for distribution. So, we were asked, our facility, to utilize our facility, that was essentially it.

**INTERVIEWER:** Okay. And based on what you just described, so serving as a distribution site, how close were these activities to your regular tasks or your regular functions as your organization?

**PARTICIPANT:** How close were they?

**INTERVIEWER:** Yeah, so I guess the question is, like, was this something that was really new to you all, were your tasks over the project similar to things that your organization does routinely?

**PARTICIPANT:** Well, it actually did have a lot of similarities, because we were talking about COVID. I actually recall, we had a table out front that we had boxes there and were encouraging people to get a box to take home. So, we spent time during my discussions with the patients, talking about the kit, and then that kit was provided on their way out, and again, as I said, there was a lot of resistance because people were just fearful of COVID, and they wanted nothing to do with anything regarding COVID. But I was able to integrate it into what I actually did every day, because I had to talk about COVID, so the fact that I had the kits there, I was able to promote it as a screening tool.

**INTERVIEWER:** That makes sense. And have you all had prior experience, I guess, COVID is a brand-new thing for everybody, but with doing these sorts of distributions, or kind of public health initiatives in the past, in your practice?

**PARTICIPANT:** I’m trying to understand what you’re asking. You’re asking if I have had to do outreach to the community or the patients that I serve to discuss a particular – because I could see this being there, similar to maybe HPV vaccines that were fairly new for adolescents, so we had to be the frontliners on promoting that, so we could do that, and then for flu immunization, that is also, especially in the black community there, that is a vaccine that they are not willing to take, so on the frontline for that. So, this pretty much fell in line with a lot of things that I do every day.

**INTERVIEWER:** Yeah, that’s exactly what I was asking about. And can you just name what resources you all required to be able to complete the tasks that you were asked to do under the project?

**PARTICIPANT:** What resources did we –

**INTERVIEWER:** Yeah, so like, what sorts of, kind of like, investments did you all need to make to be able to be successful under the project, or what things did you need to put in place, those sorts of things?

**PARTICIPANT:** The time, because that required a lot. That actually – it took more time, explaining the test than it would on a [INDISCERNIBLE] visit, so we,

**[00:10:00]**

and we needed staff on the way out, because I had those boxes there to encourage patients to take the box and participate, and therefore, I had to train the staff to say certain things and to really understand what those kits were about, and of course, we have to explain the procedure. We had to understand how the test was perform and essentially talk to the people about the simplicity of it, because people were very resistant. So, it required a lot of time and training of staff members to support this particular effort for me. And then I had a key person, and she actually will be participating in the interviews too, who understood the program as she was essential as my liaison, because I could not do it primarily by myself, so my office manager was very involved in securing the kits. She provided the space for storage, and she was the one that arranged to have those kits in place so that all I would have to do is discuss the kits, and as I said, I had to discuss the procedure itself and convince people to pick up a kit.

**INTERVIEWER:** Mm-hm. So, in addition to time and training for staff, and then this one person definitely had a large role in doing a lot of the logistics, anything else that it took to make the project happen on your end? Anything you needed specifically from the Say Yes COVID test team?

**PARTICIPANT:** Actually, because the communication was so well organized with my office manager, because she had a personal relationship with the person who was responsible for introducing the program to the community, my needs were pretty much satisfied, but that key person had direct contact with the organizers of the program, and that was very helpful to me.

**INTERVIEWER:** Okay, great. So, you’re anticipating our next question a little bit, which is, do you have any questions for the project team during your collaboration with them?

**PARTICIPANT:** They were really answered, I can say that. I did have questions, but… Well, I can’t remember any specific questions, but all of the questions, they were mostly with securing the kits and how many should we give to one household. Those questions were just easily answered, because we had access to the organizers of the program, but it was more or less things of that nature.

**INTERVIEWER:** Yeah, that makes sense. And so, our next question is, how responsive the team was to your questions. It sounds like what you’re describing is that they were very responsive.

**PARTICIPANT:** Very responsive. They really were.

**INTERVIEWER:** Okay, and if it’s different, how responsive were they to your needs? So, if you, say, needed additional resources or something more intensive than just clarification?

**PARTICIPANT:** They were very responsive, because what we did, we did more outreach, that was just from that side, but I organized two organizations outside of my office, and we actually set up a site on, for instance, and I attended this particular one, was in front of a soul food restaurant. So they had to give us a tent, and we had a table, and we actually distributed the kits as people were walking in and out, so I had a team, it was an organization called Delta Sigma Theta, I invited them to do it as a project, so we did one there, and we also did one at a church, but they were having a planned activity anyway, so we set up that site there, and we distributed the boxes. Because one of the main goals we were told was to get these kits to people in the inner city, those people who didn’t necessarily participate in the process for lots of different reasons. So we made that effort, and we did a big outreach. We actually had a third event at my office where we had another organization distributing the kits. So we organized three other sites to distribute these kits, and of course, we had to train all of the people and describe the process to them, because none of them, of course, had not done the

**[00:15:00]**

actual testing themselves, so they were unaware. But we were very successful, particularly at the soul food restaurant. That was a great event. Great event.

**INTERVIEWER:** And did you have any questions for the team around those pieces of your tasks, the training and the organizing with the other groups?

**PARTICIPANT:** It was pretty self-explanatory once we read the directions, and they were very clear. We were, everything just fell into place.

**INTERVIEWER:** And had your practice been involved in activities like that prior to this project, like working with other organizations to hold events outside of your site?

**PARTICIPANT:** Oh yes, because we actually participate in major health fairs, I’ve just not finished one, where we’ve had up to 4,000 people there. And as a matter of fact, we had the Health Department involved with this major health fair, and they gave away 1,000 of the kits. That health fair had been the last, well the time before that, we were discussing COVID and the kits, but it was more virtual, but on the ground, we actually distributed those kits, so my practice has definitely been involved, because this is a major minority health fair that we’ve been doing for 22 years, and we’ve set up booths, and we’ve distributed information to people when we get vendors in other health fields, and even social programs, mental health, we are a part of organizing, at least the one that does it in our office, we organized this event, so we’re very [INDISCERNIBLE] to doing that.

**INTERVIEWER:** Okay, yeah, that makes a lot of sense to me. Do you think, ultimately, the project ended up addressing your county’s needs, or a need in your county around COVID-19?

**PARTICIPANT:** It did, but I tell you, it came, when the kits were first there, the marketing probably could have been improved. I thought maybe it was enough before we actually began to distribute the kits, people were not informed enough. And what’s enough? I don’t know, because people were so fearful of the disease itself until they just didn’t want to be involved in anything, so it would have been a very difficult thing to do to make everybody comfortable with the process, and that would have made it easier, because I tell you what happened, once we got rid of the kits, people understood, and I had so many calls to my office trying to get the kits. Yeah, so all of the efforts worked, but it did not make people comfortable, because it was on that set period of time that we were doing this project, so when the project ended, it was right at the end, people became comfortable with what we were trying to get, so I think marketing was the biggest factor in this, and I don’t know, because I’m not in that area, how it could have been done better, but maybe news, radio spots, news programs, so we missed the prime time with getting people more engaged, but they did come to appreciate it, and that was very helpful as people reflected back on what had been done.

**INTERVIEWER:** So, what do you think led people to eventually be more comfortable with it? Or what do you think were the biggest factors that made them ultimately feel comfortable?

**PARTICIPANT:** That actually, those who had those kits in their homes and were able to do it and understand that they have a positive result, and then they understood that they didn’t have to rush out to the doctor’s office and get them done and stand in the long lines, so they were able -- and then too, people weren’t doing that towards the end, they became much more comfortable with the tests, and because we had established sites all over Chattanooga where people were standing in long lines to get the tests done, so then it became very significant to mesh all together, gosh, these are test kits that we can do at home, and if we find that we’re positive, we can call in, because they were getting off time from work, and of course, a lot of them still had to go for verification, because people will always abuse the system, but that was very helpful, because people were so fearful of COVID during those days, that if they found out they had it,

**[00:20:00]**

They didn’t want their family members to contract it, so it became very much useful information to them to have.

**INTERVIEWER:** Yeah, that makes sense. And aside from the need that was addressed by this program, what do you see as the needs in your community related to COVID-19?

**PARTICIPANT:** What other needs?

**INTERVIEWER:** Yeah, so this program addressed at-home testing, but are there other needs, or what do you see as the biggest need or barrier to folks being safe from COVID-19?

**PARTICIPANT:** Well, the biggest barrier is, it’s become so politicized. It is unreal, it is very much unreal, because I tell you, what happens in my practice, because I am a firm believer in immunizations, because that’s what I do, and I have seen the effectiveness of it, but I can clearly tell you the people who will accept those vaccines, and we have a lot of minorities that still are very fearful, but we have all those who, and I’ll have to say it, of Republican persuasions, walk in and they get angry with me when I tell them, all the time, and they say, Dr. Hubbard, you’re repeating yourself! I said, yes, but that’s part of my job. I am going to say it, because it’s an ongoing problem. That is our biggest barrier. We have people who will not respect being safe, they will come out with COVID, and they will not wear a mask, and I don’t know how we will get any of this to mesh anymore, because people are very divided for political reasons, that’s one thing, and then others don’t understand, well some are divided for political reasons, and then you have this group that are fearful that they are being tested on, and this is some scary thing for them, so altogether, I have to come into the office on some days, and I ask almost every patient, have they been vaccinated, will you consider it, and some days, I get maybe 70%, other days, 50%, and it is a wide range, and because of how this has been presented and people were given choices in light of this being a public health crisis, I don’t know how you’ll ever blend it all together, because unless it’s made mandatory, get a grip on it, at that rate, it has been wonderful that the hospitals required it and a lot of the employees, that way, and even one private prep school here required all of their students to take the vaccine, and that was a major thing, because you have a lot of people politically not wanting to take those vaccines, but they stood their ground, and because they did, we had a large group of the parents began to become immunized, because they know, they wanted their kids, but even they had to back off and carve out a group that they had to allow to come and not be, so I don’t know how we can solve that unless we start from the top. How do we do that? I don’t know. But that is the major barrier. It really is, because I am in this daily. I talk about COVID every day at work, and I know how difficult it is out here. But it is useful now, these home kits. They really are.

**INTERVIEWER:** Yeah, that’s great. So yeah, our next question is what you see is the biggest barrier you see to COVID-19 testing specifically.

**PARTICIPANT:** Well, supply, because we’re not going to have enough to do that. I had someone call me just this morning on the test they did, and I told them, I think perfecting those tests and them being in adequate supply, but once charges are required, that’s going to decrease usage, because I had one friend who was told that it would cost him $200 to go out, he had symptoms, but he’s not going to get tested or go out, he was yelling.

**[00:25:00]**

So that is, and they will spread it, and I tell people all the time, their little kids are going to spread it, and we even at this last health fair had it set up where we gave incentives to everybody who got a COVID test, and we had maybe 800 people and 200 ended up getting it, so that was significant. But it is a problem, because all the fear and politically. But the kits, I think, if we continue to perfect the kits, and they are made at very low cost, for free, they will be utilized. People appreciate the home kits now. They really do.

**INTERVIEWER:** Yeah. So, our next question is whether or not, or to what extent you thought that the Say Yes COVID test project responded well to the barriers that you’ve covered around testing?

**PARTICIPANT:** One thing, ultimately, it was a positive effect, it did have a positive effect on the community, because you had to do these different things, and in retrospect, it’s really appreciated. Not at the time, but people can reflect on it, and they understand how significant it is. And it’s the very thing that people want. This was introducing that concept. Now everybody wants that in the home, so it was very successful. Yes. I have mine stacked downstairs, and because of the position I was in, and I have children in different places that benefitted from me being in my position, because they all got a box to screen the children, and everybody has had COVID, except for a couple of them out of my family.

**INTERVIEWER:** Yeah, so what do you think specifically about the project helped it overcome some of the barriers and make it easy for people to use?

**PARTICIPANT:** Say that – ask that question again?

**INTERVIEWER:** Yeah, so I guess, what aspects of the project, the way that the kits were distributed or provided to folks made it easy for them to use, the COVID test, or helped overcome some of the barriers they were experiencing to testing?

**PARTICIPANT:** I think most people became more knowledgeable of it, it really was easier, well once more people became more knowledgeable, they became more comfortable, and they sought the test. They understand the value that’s being determined, they understood they were valuable.

**INTERVIEWER:** Yeah, that makes a lot of sense. And changing gears a little bit, at the end of the project, so once you all were all done, how close did you feel like your typical activities matched to what you were asked to do for the Say Yes COVID test project, so I think this question is asking about, we had talked up front about how the project was initially described to you, but is that kind of how things ended up going, or did you all make changes over the course of the project?

**PARTICIPANT:** I’m trying to think, because when we first started the project, we did not think it was very useful, because people were so resistant, and it required so much of our time to encourage the patients to take the kits, but I think towards the end, with all of the news programs and people actually seeing so many of their close relatives becoming very ill and even dying from the disease, that the attitudes towards the kits, so I would think that at the end of the project, the attitudes towards home testing really did become significant in the lives of everybody who participated. I’m struggling with how to say it, because there was something early on that we said,

**[00:30:00]**

oh, this is just so wasteful, we thought that because so many people would take those kits, and we really had to shove them and make them accept them, but towards the end, we had everybody calling for additional ones, so the acceptance overall, I don’t know if I’m answering your question, but the acceptance of those kits were definitely, made more, where the people accepted the kits more after the program, I guess I have to say about that.

**INTERVIEWER:** And did you all end up changing your approach or how you were going about it once you noticed that people were really resistant, or it just kind of happened over time?

**PARTICIPANT:** Well I tell you being in a practice, how we changed ours, if they took those tests at home, we were able to protect ourselves more, and they were positive, we wouldn’t have allowed them to come to the office, because what we did, anyone with the symptoms had to be tested for COVID in the car, and if they were found to be positive, we would not allow them to come into the office. So, it saved time with a lot of people, because they knew they could not come to the office if they were positive, and if they came in with sick visits, they were going to be tested out in the car, so this saved them from having to come to the office and get the test. So, we were able to do more effective screenings, because the only ones who would do the tests, would do PCRs would be if they had the symptoms, and they tested negatively with the test kits. So, our practice has changed. We were able to keep those people out of circulation more effectively if they tested positive at home.

**INTERVIEWER:** And in general, how difficult did you find that your activities or your tasks for the Say Yes COVID test project?

**PARTICIPANT:** They were pretty difficult, because it took so much time convincing people to take it. When we first started giving those kits, it truly was a problem getting people to accept them, and it was because of a lack of education. They weren’t comfortable with what those kits really were, and they just weren’t going to let you hand them something, as if they were taking the kits, they would introduce COVID into their homes, and they just would not take it. There was just, that much fear was there.

**INTERVIEWER:** And did you end up doing anything differently to try and convince them than what you thought you would do at the beginning?

**PARTICIPANT:** No.

**INTERVIEWER:** Sounds like you guys were doing all you could.

**PARTICIPANT:** I know we were! We were! We were out there, yes, we were. I don’t think we did anything differently, because we were focused on getting the kits in the hands of those that the program targeted.

**INTERVIEWER:** Yeah, it’s really challenging. [INDISCERNIBLE] amazing what you were able to accomplish. So, what do you think were some reasons individuals in your community ultimately took the test kits?

**PARTICIPANT:** Because they were comfortable with us as providers, and when you have people who trust you with their medical care, they did it because we told them that it was a safe thing to do. And then, too, later on, they continued to hear in the news, test kits, test kits, and then some employees would accept them having positive tests at home and would allow them to stay home with pay. That first year, because we were given the grants where we could afford that, but once that stop, of course, we could not pay attention to it.

**INTERVIEWER:** Right. And do people in your community have the option to order test kits online through the program?

**PARTICIPANT:** I don’t think, through the program, no, it was after the program. It may have been towards the end of the program, I’m just, I know they could not initially, but maybe towards the end, they were given that option.

**[00:35:00]**

**INTERVIEWER:** Do you have a sense if people preferred online ordering vs. picking it up in person, or if people in your community maybe preferred one option vs. the other?

**PARTICIPANT:** Now it depends. Those who are used to and comfortable with a computer, that is not a problem, but for a large percentage of the population, it needs to be convenient, and they just pick it up. So, you definitely need both avenues, because most of my friends, they would much prefer just doing it online and not going out, and young people that are very versed with technology, that’s just not an issue. For others, they will need them at a facility, and at a convenient place: the pharmacy, the grocery store, and the doctor’s office. Things like that have been the choice places.

**INTERVIEWER:** Do you have any recommendations for the Say Yes COVID test project to improve their approach moving forward?

**PARTICIPANT:** Well, the advertising, just doing that thoroughly, and really establishing positive relationships with two places where a large group of these people, I always say patients, but people would meet, and that would be health departments, grocery stores, the pharmacies, letting them take the lead role, especially those places in terms of having people there or promotions there to encourage participation. Because it does work.

**INTERVIEWER:** Right, right. So, you spoke on that earlier, the challenges with marketing or advertising around this project. It sounds like you think there’s room for improvement, both in terms of how early it started, and then also where the advertisements were placed, am I understanding that correctly?

**PARTICIPANT:** That is very true, because I think before we received our kits to distribute it, it may have been one or two weeks before the event, and that was a big barrier there, because we had to talk about it too much. We needed more advertising, showing the procedure on TV, because it’s very simple, demonstrating that, really discussing the purpose of the kits and how convenient and how they would identify index cases in these people, not go out into the community at least for 10 days, then.

**INTERVIEWER:** That makes sense. Anything else to add there? We just have a couple more questions for you.

**PARTICIPANT:** No, because the fact that they were free, and it was very positive. I’m really happy that we were able to do that for as long as we did, and then giving people breaks from their jobs, because we were giving those incentives to pay for those physicians, I don’t have anything else to add.

**INTERVIEWER:** Okay, so just two kind of wrap-up questions, moving forward, what would you like to see happen with your community and their health? What are your biggest goals or hopes for your community and their health?

**PARTICIPANT:** Is this surrounding COVID?

**INTERVIEWER:** Not necessarily.

**PARTICIPANT:** Well, I tell you, I think that community providers need to be more supportive, because we see more of the high risk populations, the people who don’t necessarily understand the importance of appointments, it is very tiring on our system, and I have a group, in practices, we have all kinds of patients, and if you serve a certain group, providers need to be, struggling with the extra help, as I said, during this project, it required staff time to do the promotions and to do the education and teaching.

**[00:40:00]**

We just have to provide more education, I guess, overall, and really make it tangible where people can go up and get services readily from those providers, and that’s often hard to cover, because people themselves are not necessarily stable, and they want these immediate, and they don’t like the plan, so to accommodate that, so it’s very difficult, because I do believe people already are not made to be as responsible as they should be, so I’m putting another layer on it, but just providing adequate services or support to those people who are willing to participate in these projects, and we should be able to get those grants to help us really do the programs effectively and not allow it to tie into our time with the patients, because I would have really enjoyed someone for a brief period of time coming in and talk about it, being able to educate the patients, and we would sell it big time with that time, even to tell how the testing should be done, and that could be with anything that we’re trying to get big medical issues over to a population, we really have to give the practitioners who are the frontliners with the key or the target population, the support that they need to educate and to provide those services.

**INTERVIEWER:** Yeah, that makes a lot of sense. Do you have any additional questions or comments for us? Anything that you want to share that we haven’t talked about already?

**PARTICIPANT:** Well, will this be ongoing, or it’s going to take another –

**INTERVIEWER:** You mean the program?

**PARTICIPANT:** Yes, the program.

**INTERVIEWER:** I, actually, don’t know the answer to that. So, we’re an external evaluation group, so not necessarily the folks in charge of making that decision. I think that’s something pending we would all like to see if folks found it helpful, but hopefully you sharing your experience will help us make those decisions.

**PARTICIPANT:** Okay, thank you.

**INTERVIEWER:** Yeah, thank you. And I’m going to turn it over to Christina. I think she has some logistics for the gift card.

**PARTICIPANT:** Okay, thank you. This is the fun part!

**CHRISTINA CARILLI:** Yeah, definitely is. So, I just want to confirm the email that you would like the gift card to be sent to. Is it the same one that you signed up with?

**PARTICIPANT:** Yes.

**CHRISTINA CARILLI:** Okay, perfect. So, we have that. Perfect. So, thank you so much.

**PARTICIPANT:** Thank you!

**INTERVIEWER:** Yeah, thank you [participant name]. Have a good rest of your day.

**PARTICIPANT:** Thank you.
